# Supplementary figures and images for: Thy1 transgenic mice expressing the red fluorescent calcium indicator jRGECO1a for neuronal population imaging in vivo
Source: PLoS One. 2018 Oct 11;13(10):e0205444. doi: 10.1371/journal.pone.0205444 (PMC6181368; doi:10.1371/journal.pone.0205444)

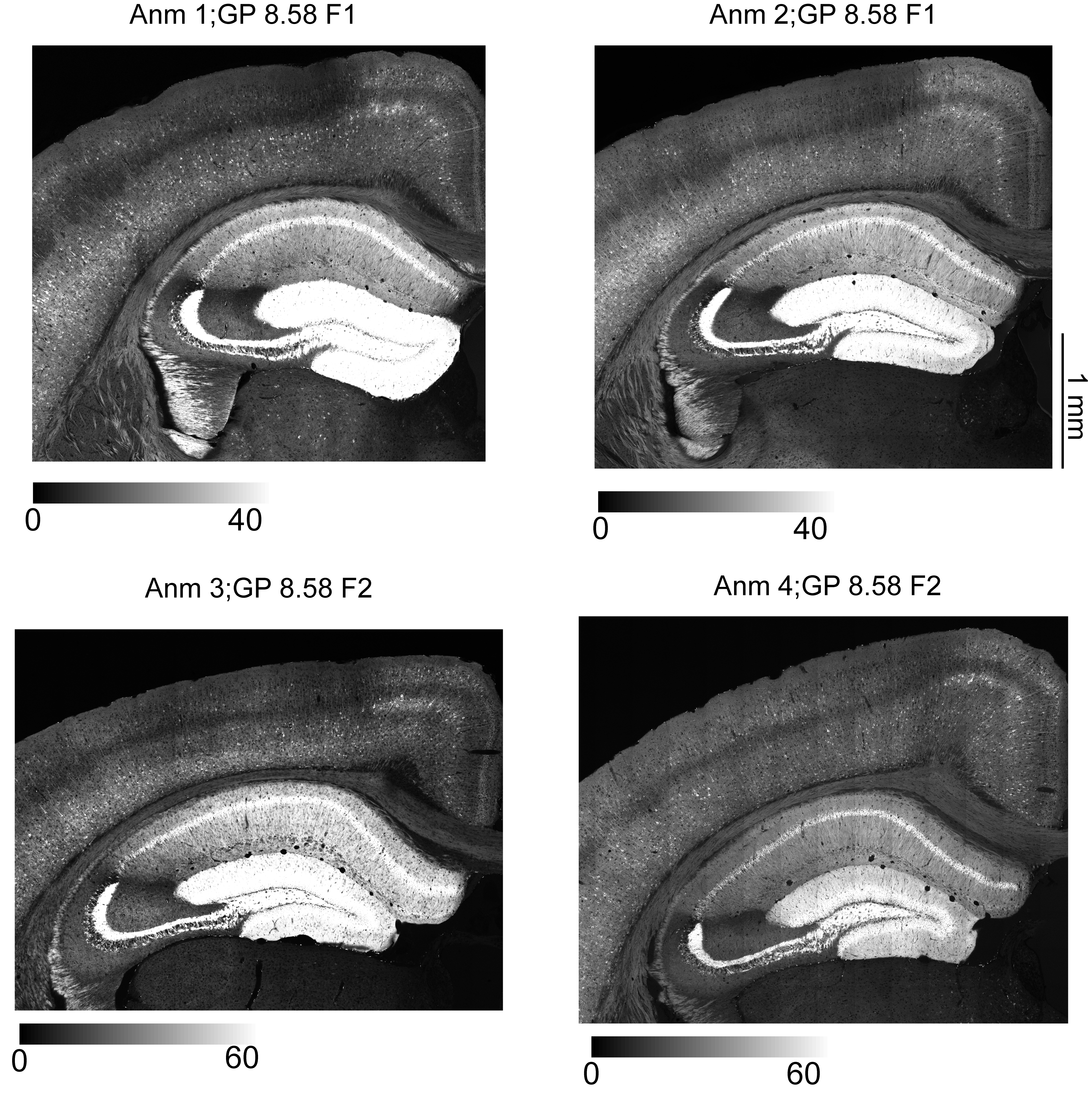

Supplement: S1 Fig — Confocal images of coronal sections from four different GP8.58 mice, taken from the first and second generations of this line (F1 and F2 respectively), show similar labeling patterns. (TIF) [file pone.0205444.s001.tif]

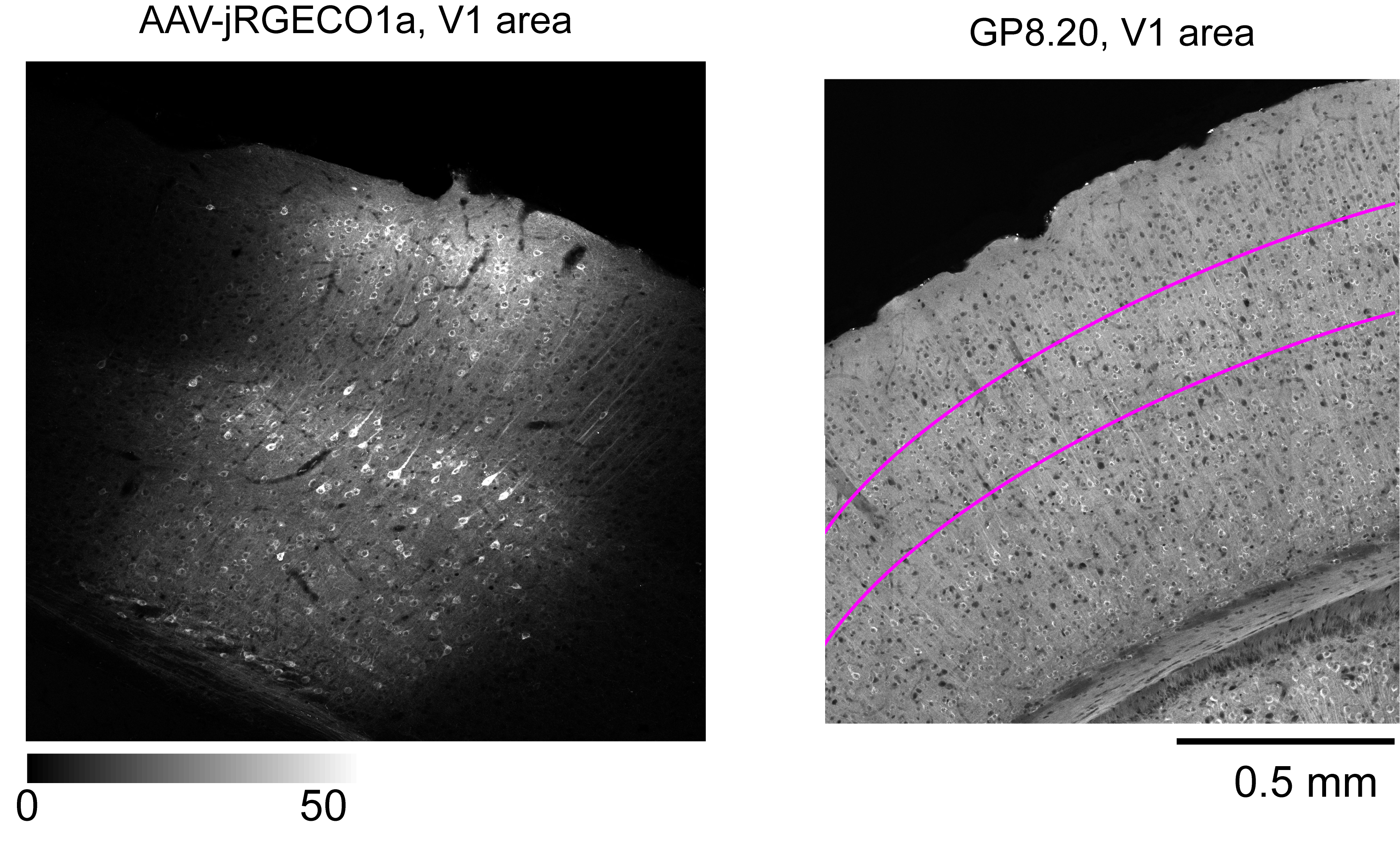

Supplement: S2 Fig — Typical labeling patterns of AAV-mediated expression (left) and Thy1-transgenic expression (GP8.20, right) are shown. AAV-mediated expression is localized around the injection site and exhibits large variability in fluorescence protein expression level. The Thy1-transgenic expression is generally lower than what achieved with AAV but is more uniform and stable over longer times. Magenta lines indicate the location of labeled L4 cells. (TIF) [file pone.0205444.s002.tif]

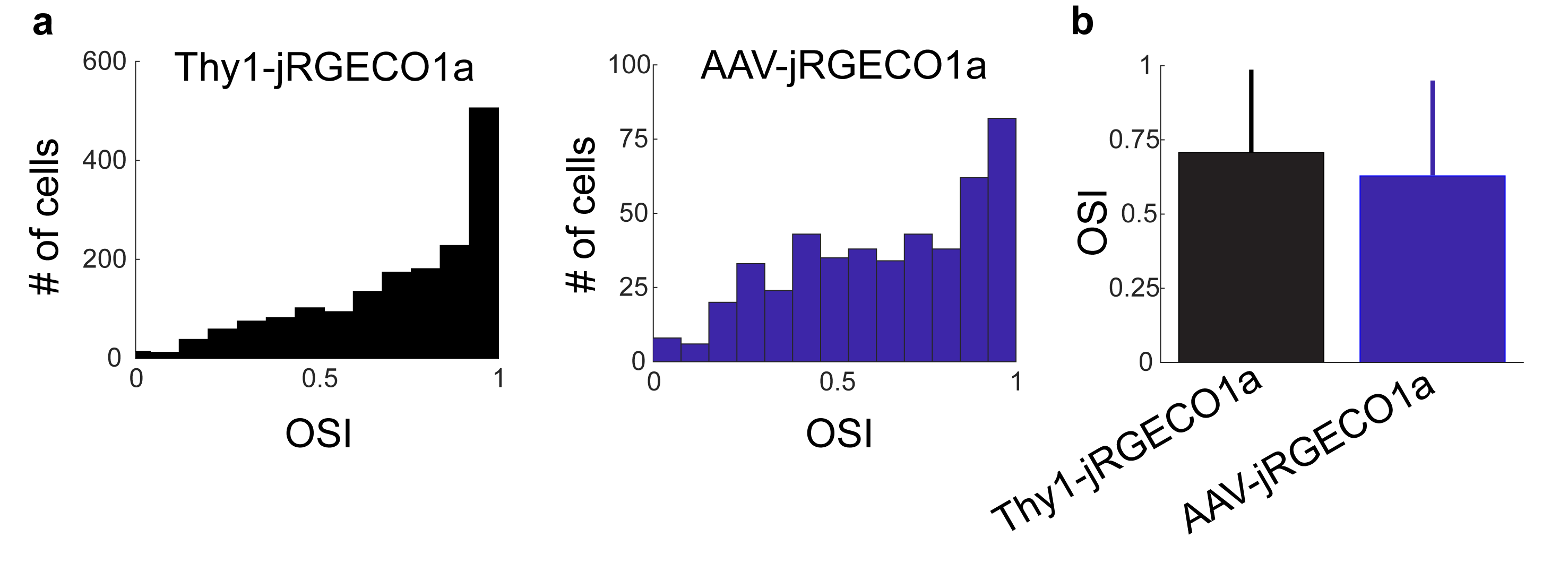

Supplement: S3 Fig — OSI indicates the tuning level of a cell to a specific orientation of a drifting grating stimulus. The similar distribution of OSI values for Thy1-jRGECO1a and AAV-jRGECO1a, showed in panel a, and summarized in panel b, indicates that the tuning of the measured cells was not disturbed (Thy1-jRGECO1a, 1725 cells from 8 mice; AAV-jRGECO1a, 472 cells from 4 mice). (TIF) [file pone.0205444.s003.tif]

# GP 8.5

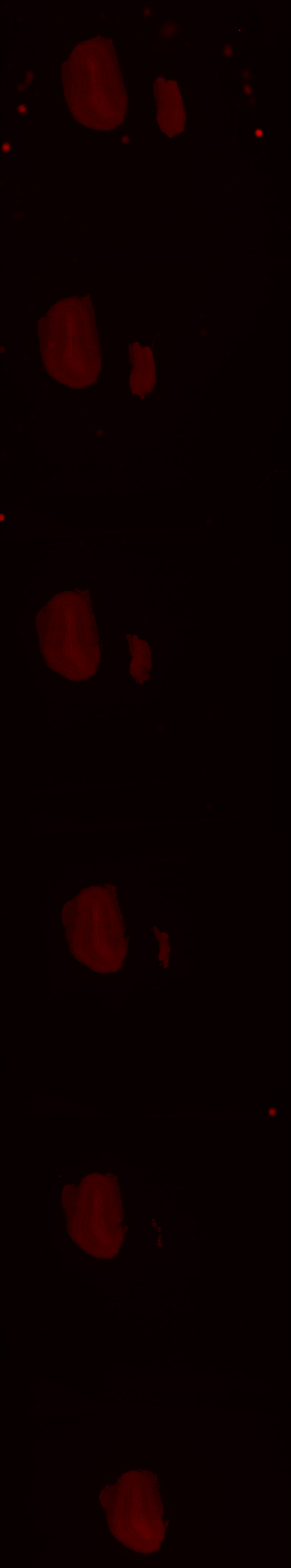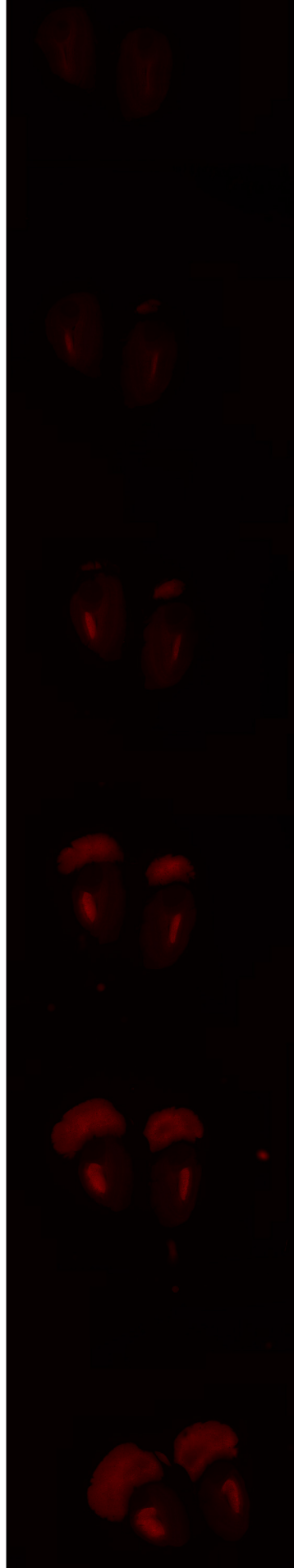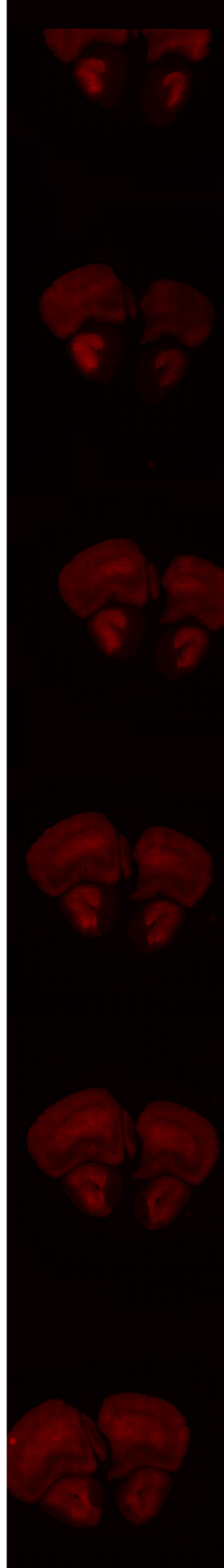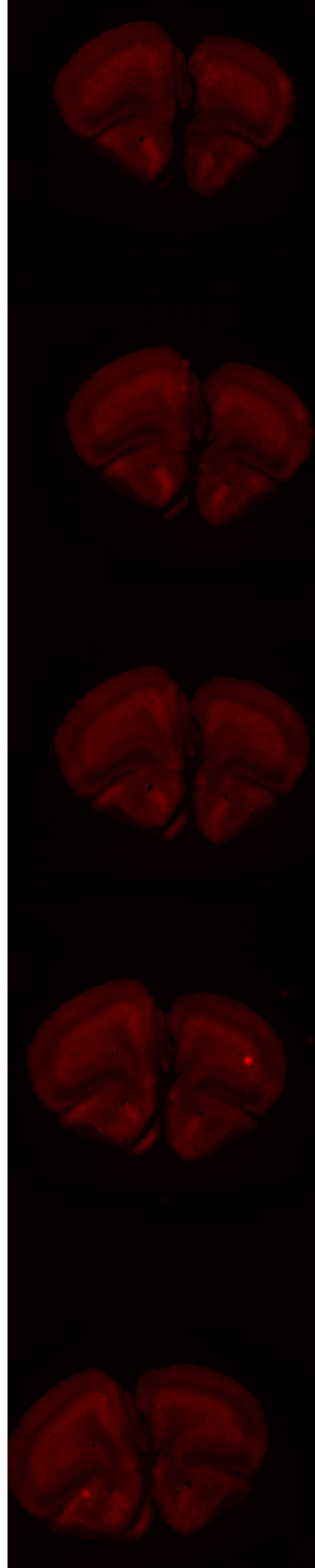

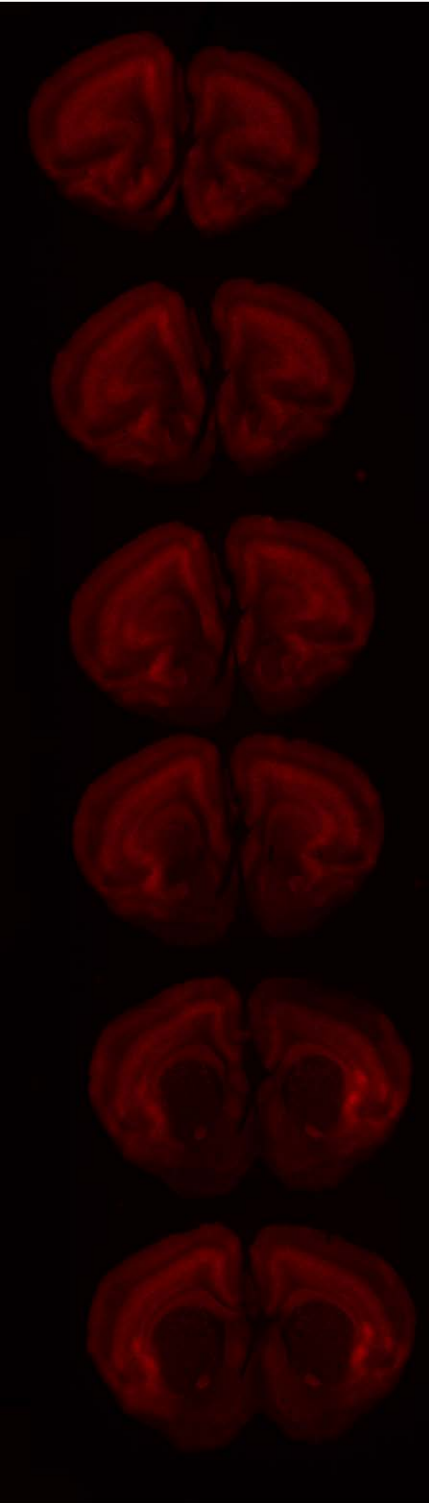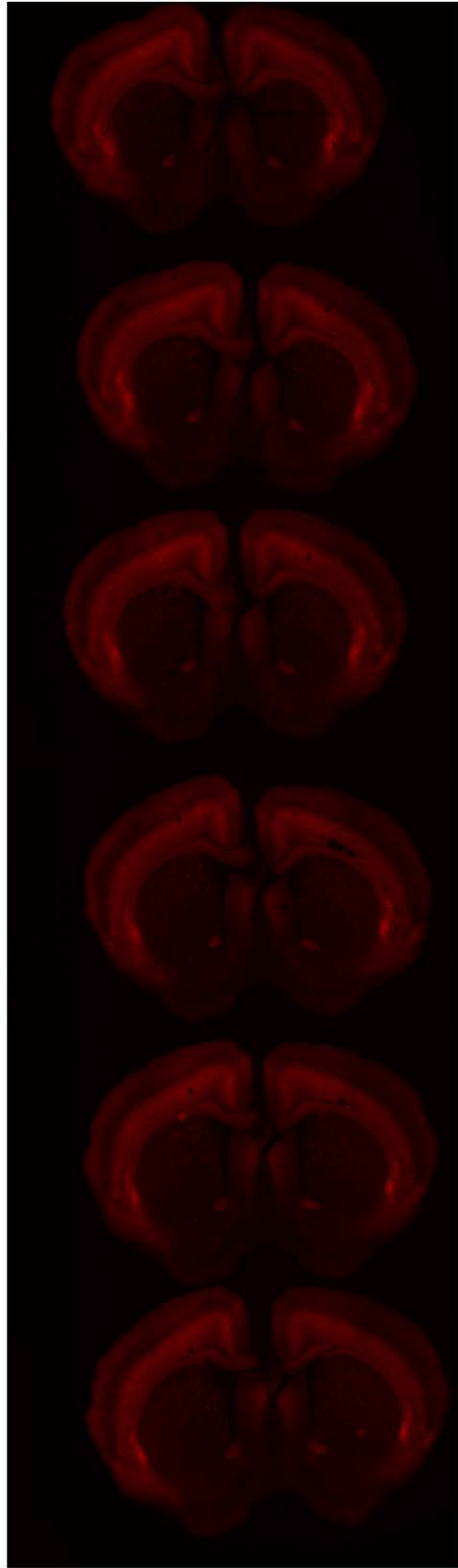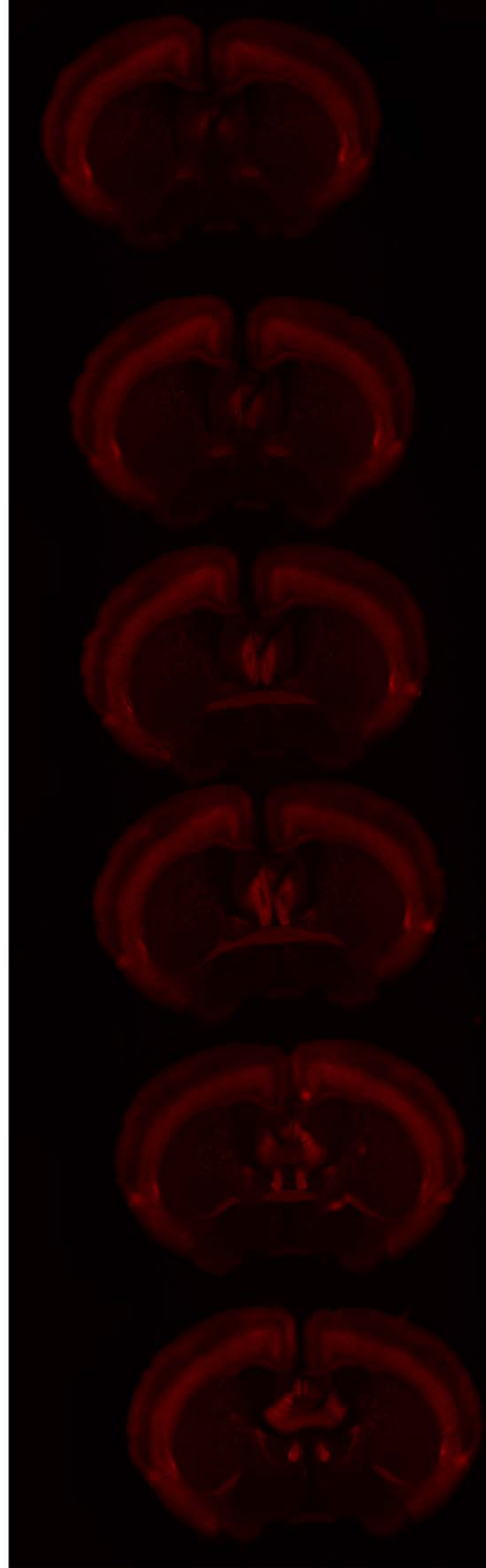

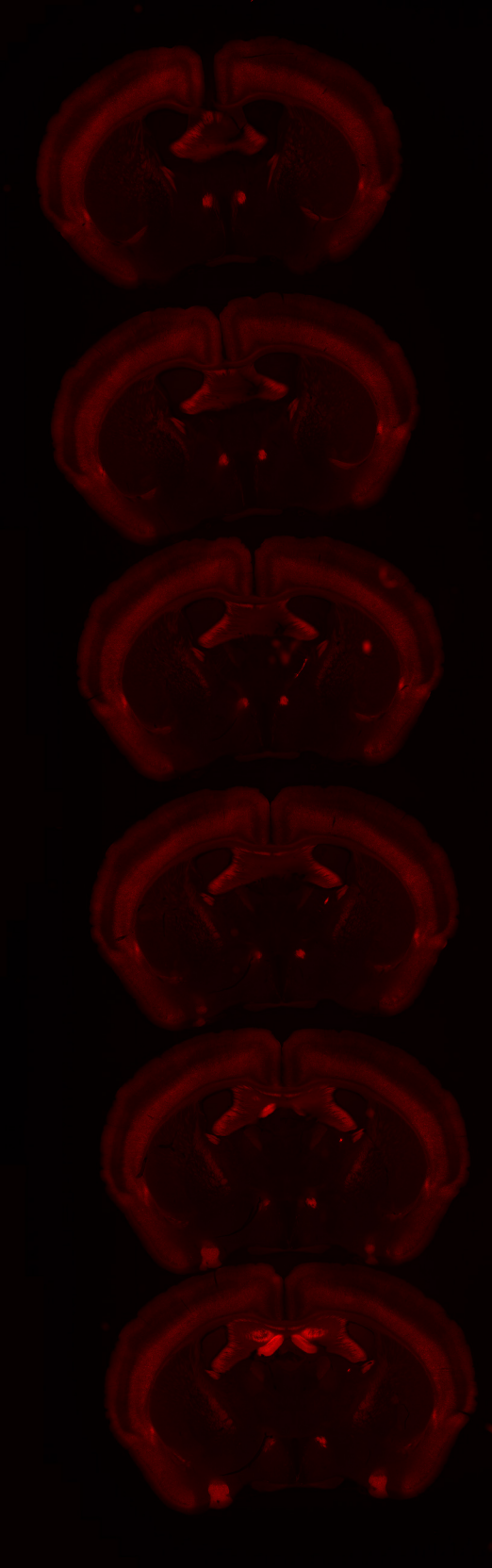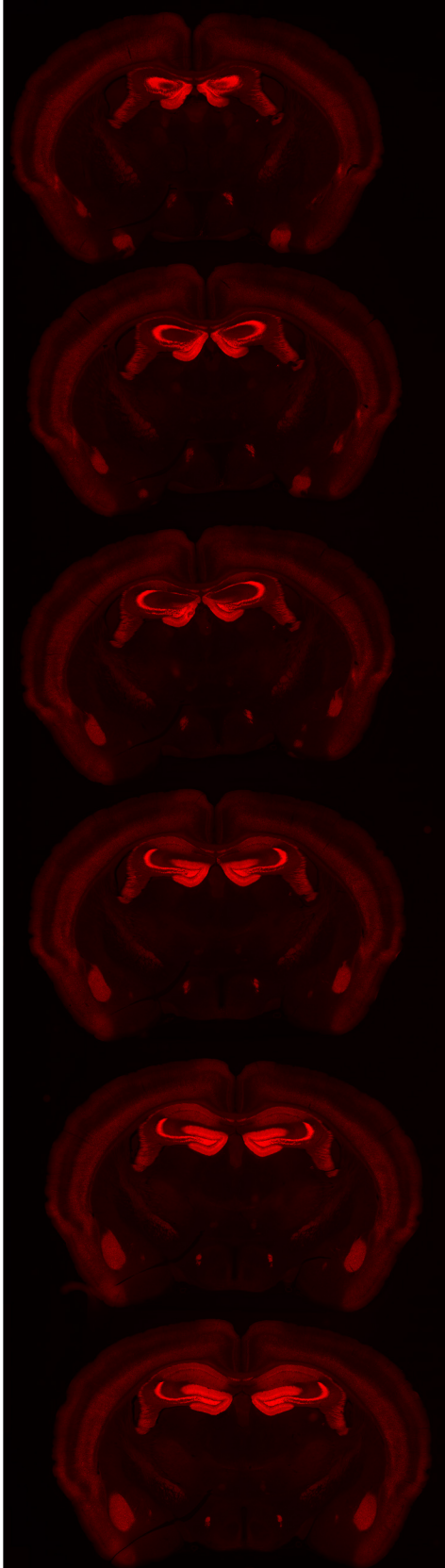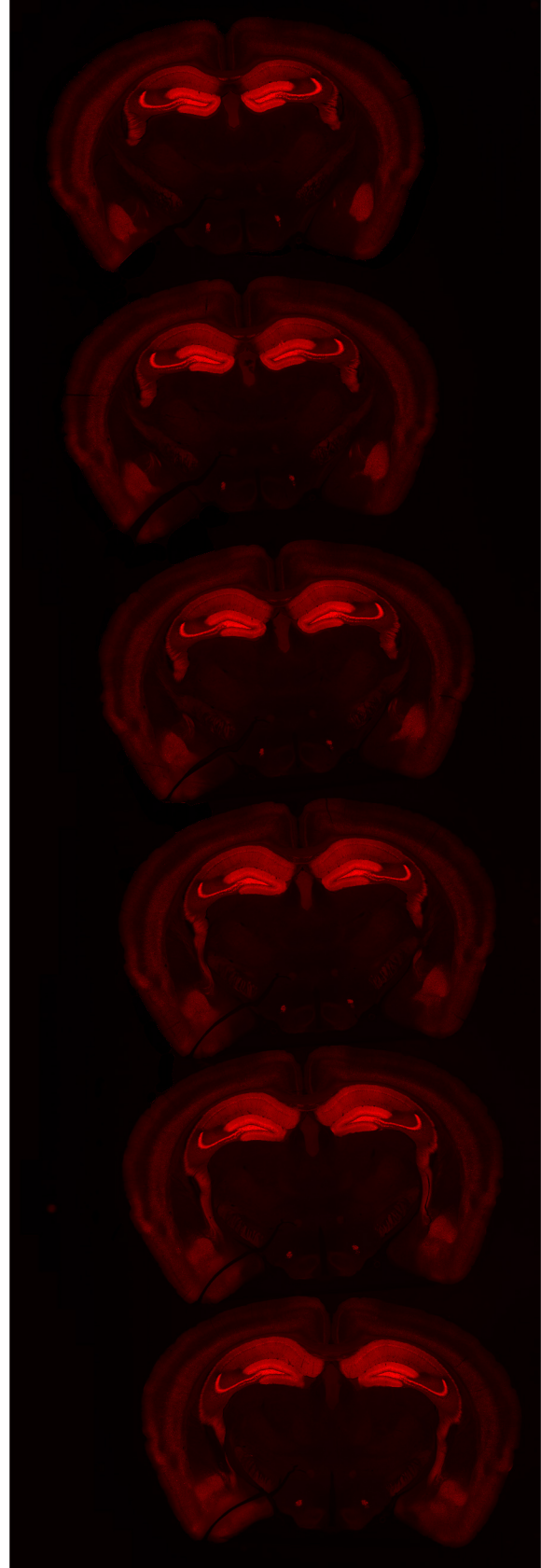

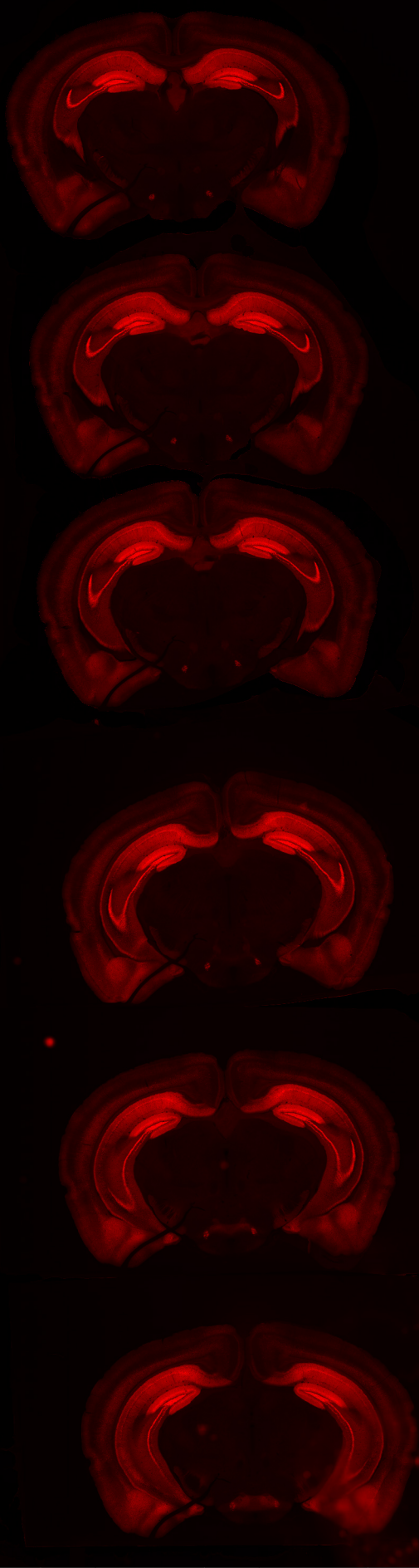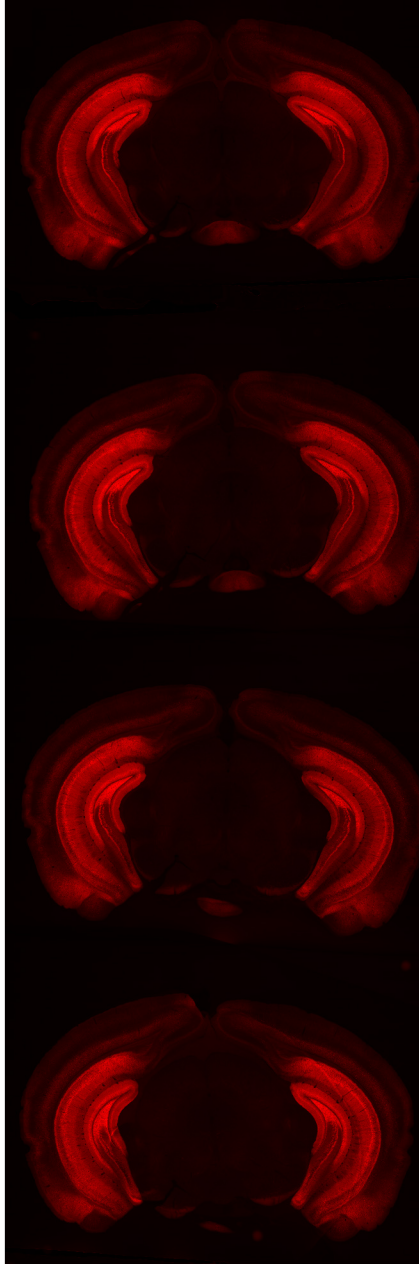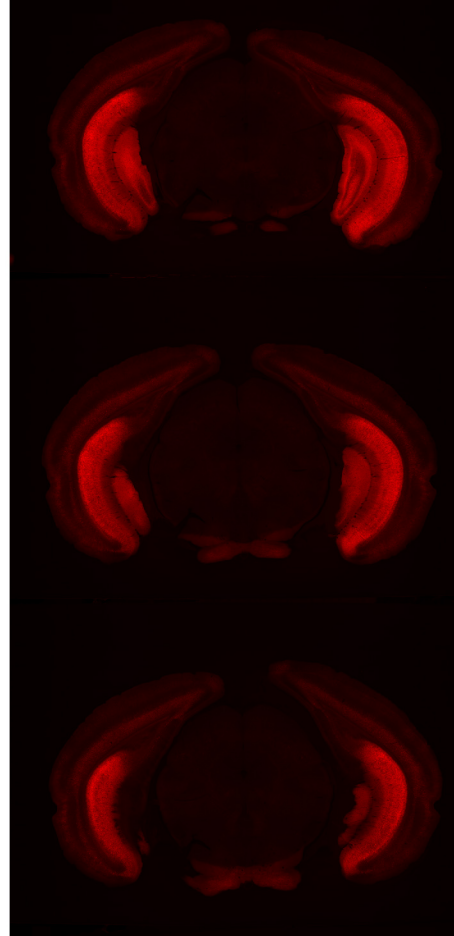

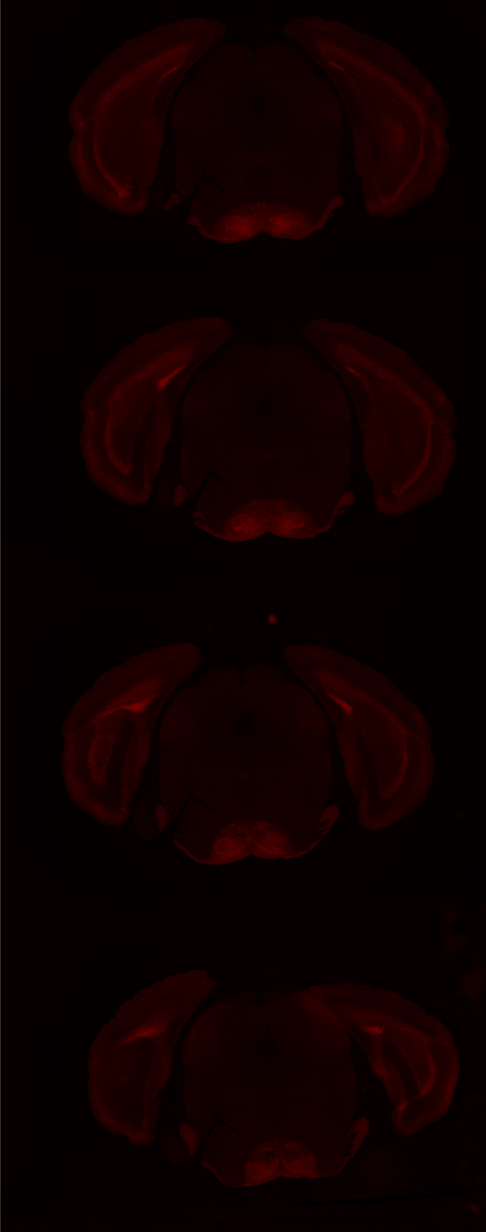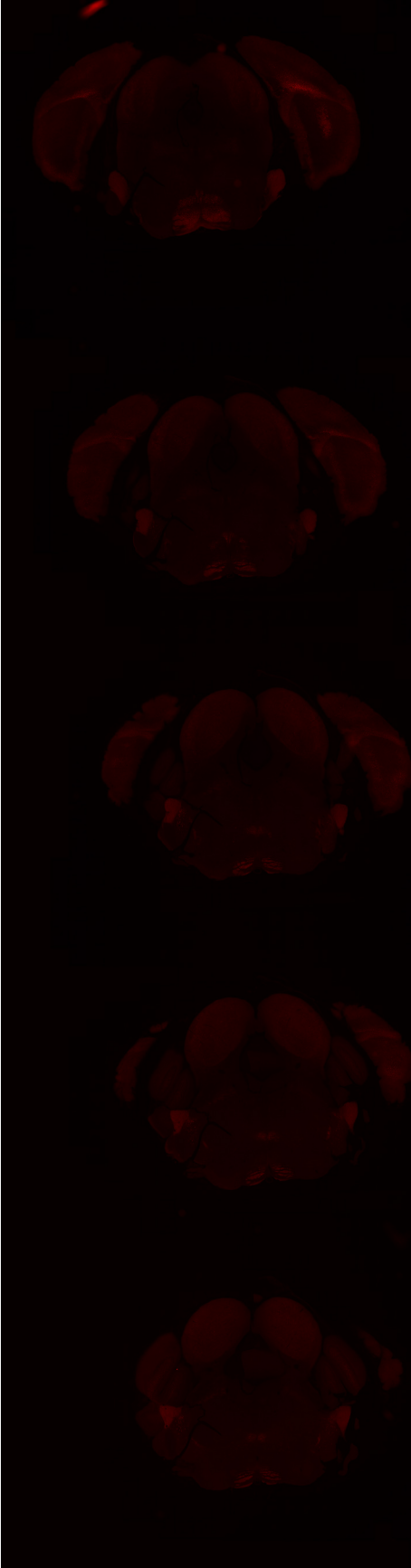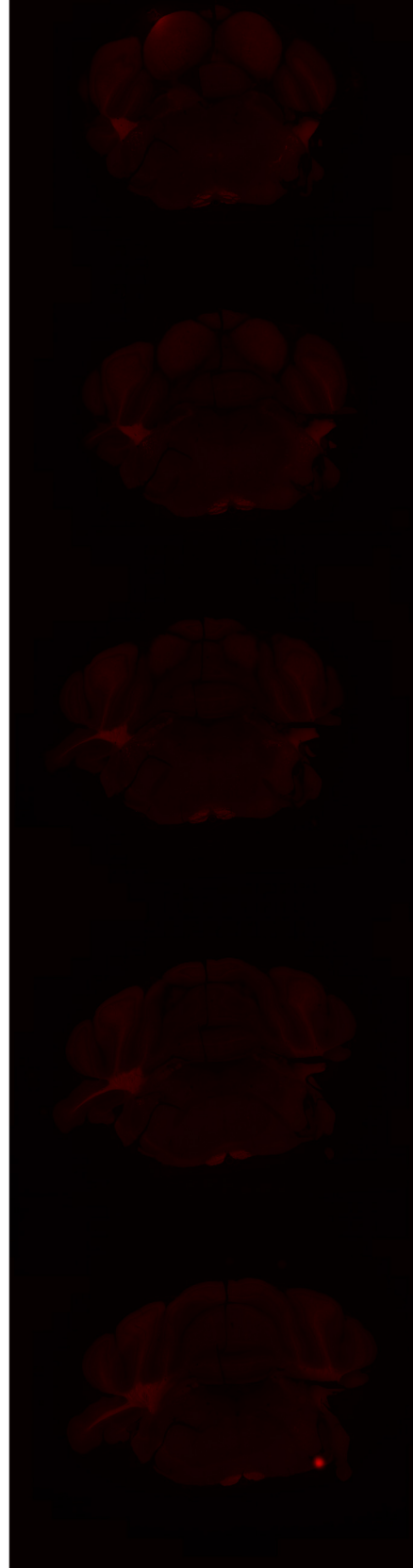

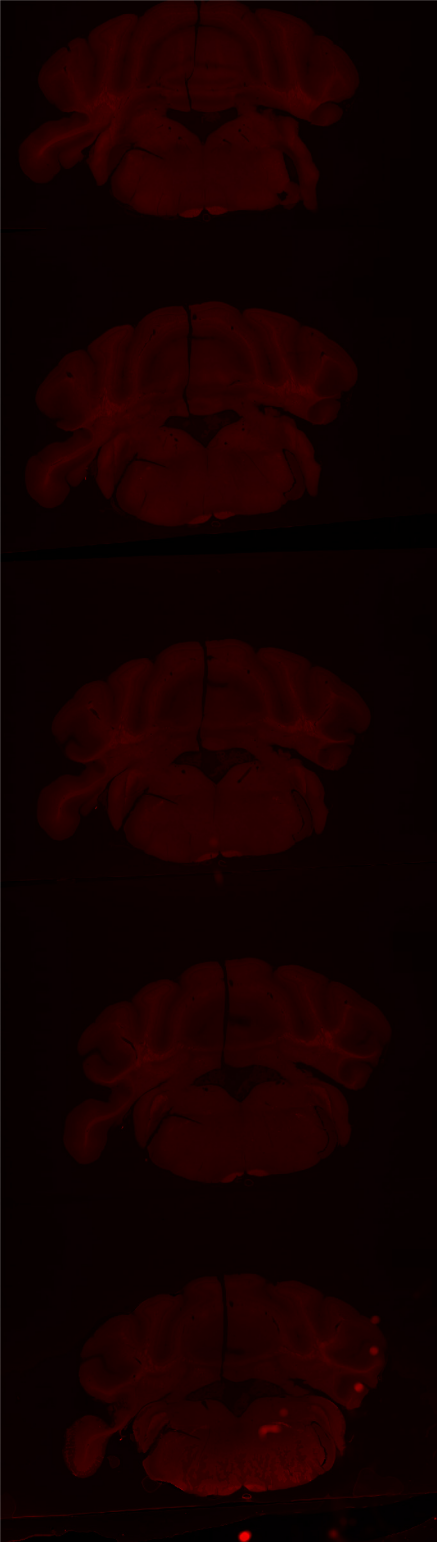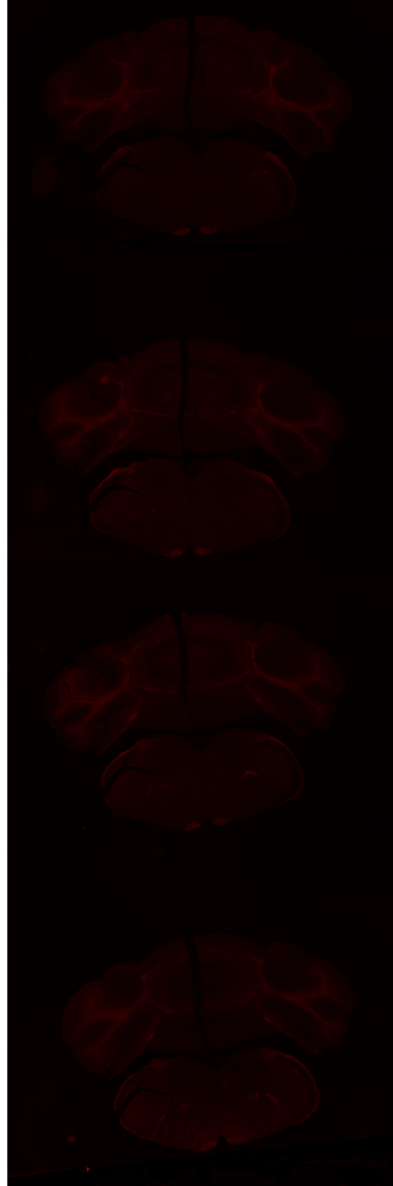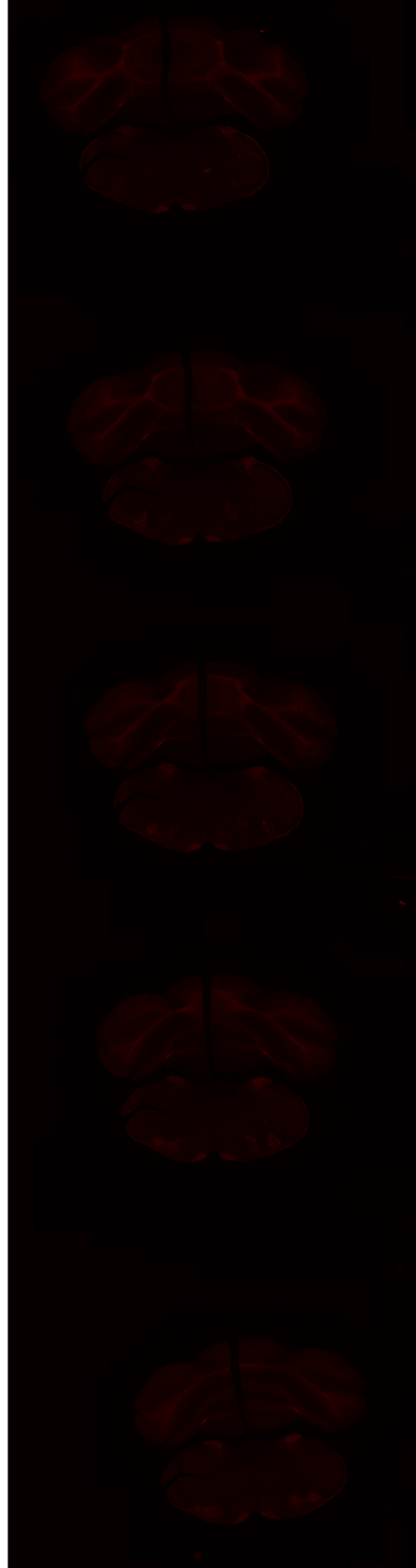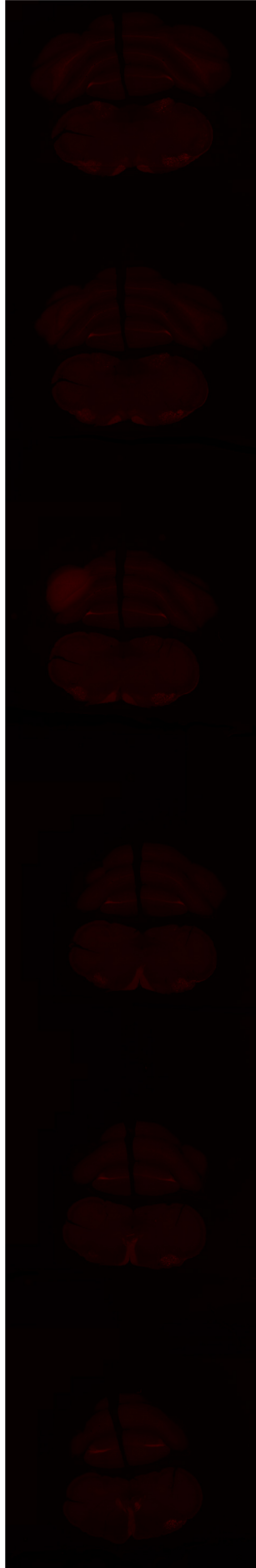

# GP 8.7

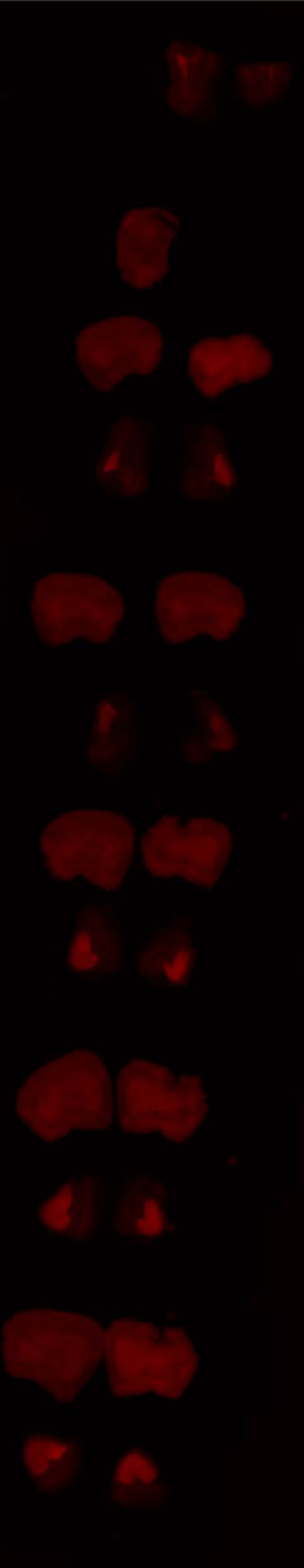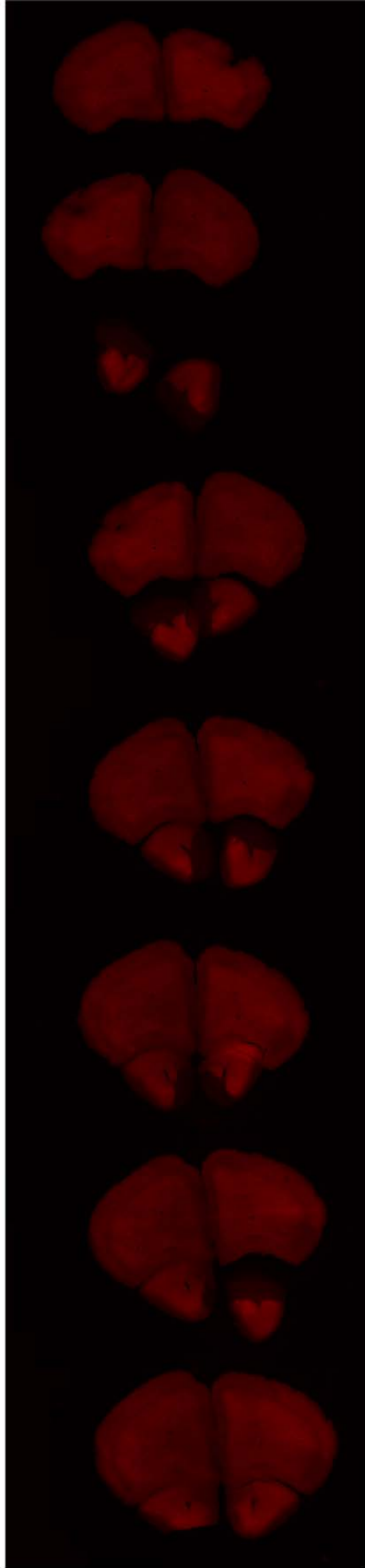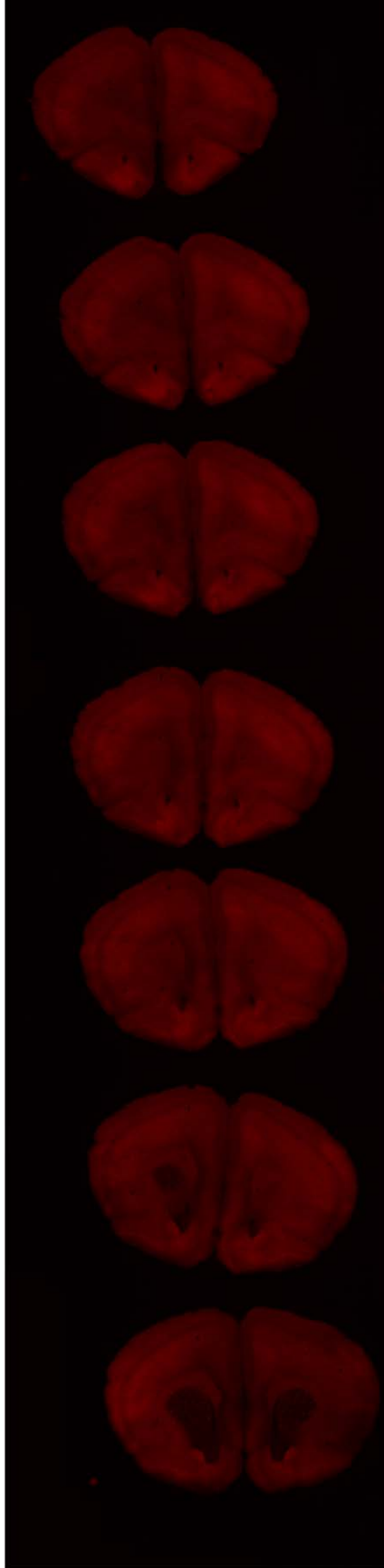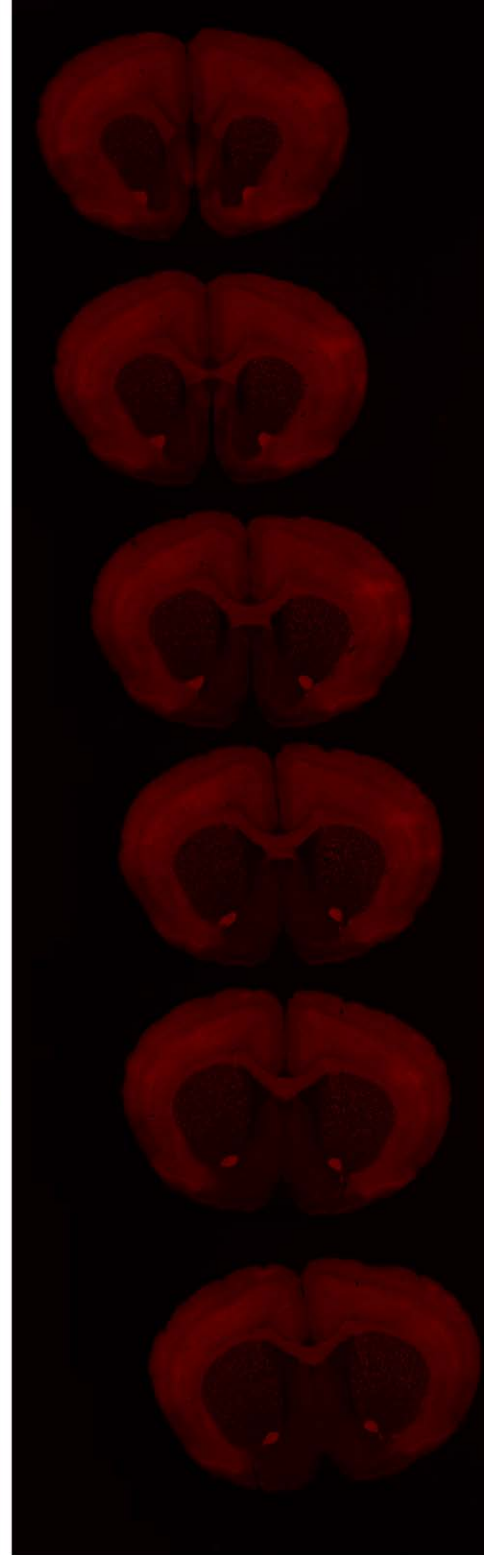

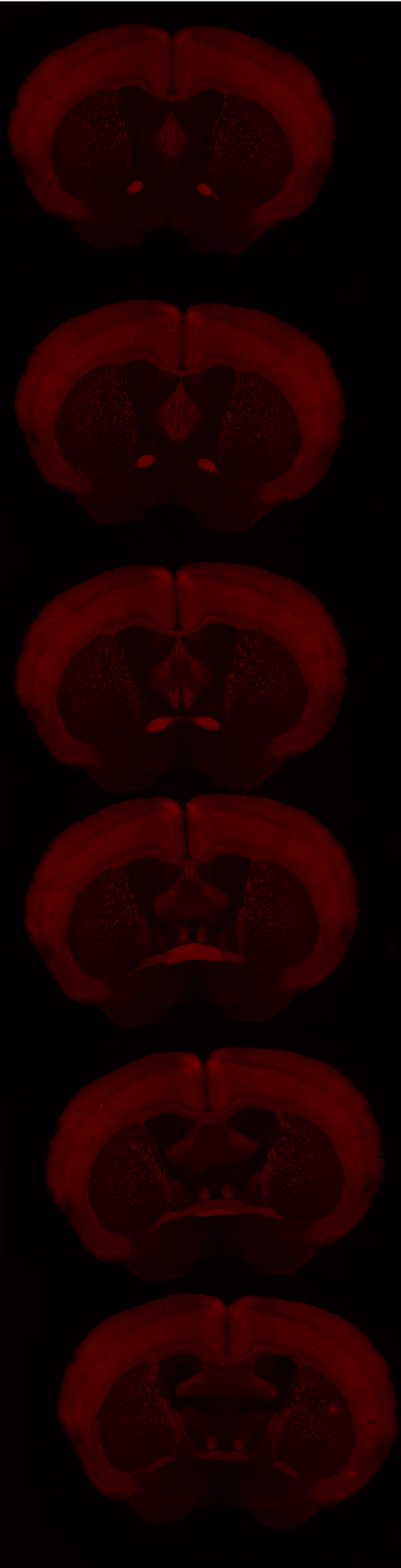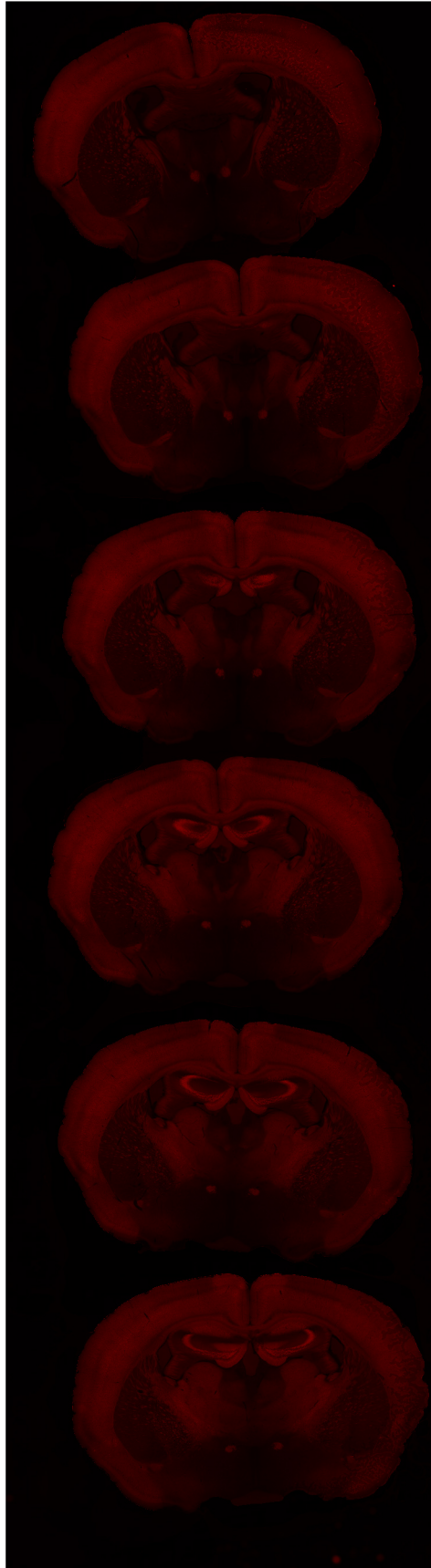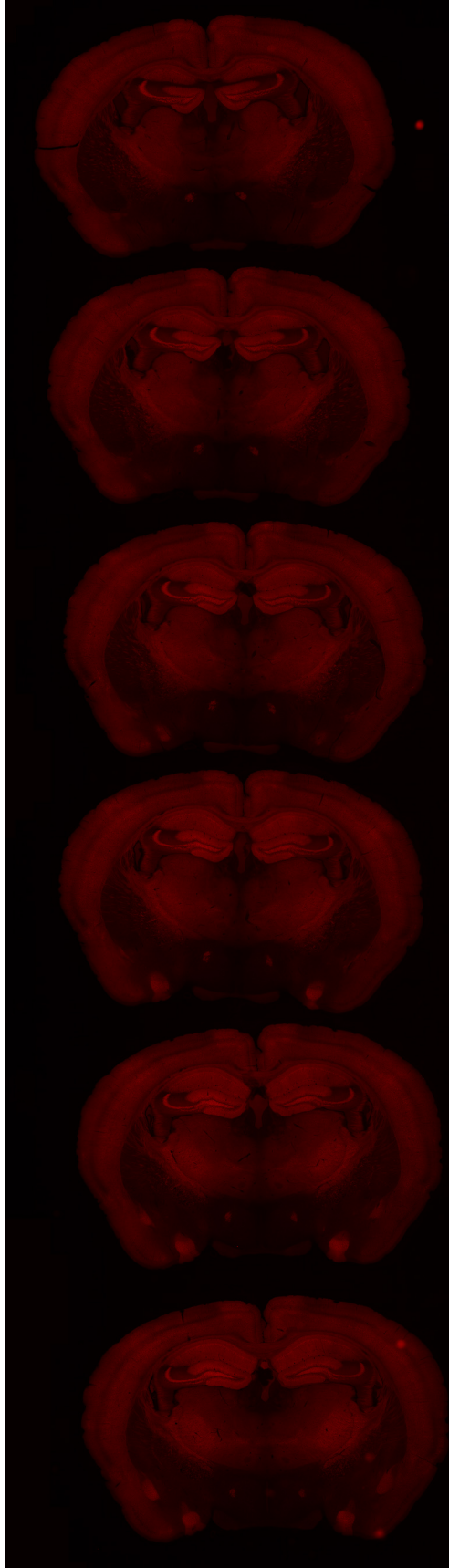

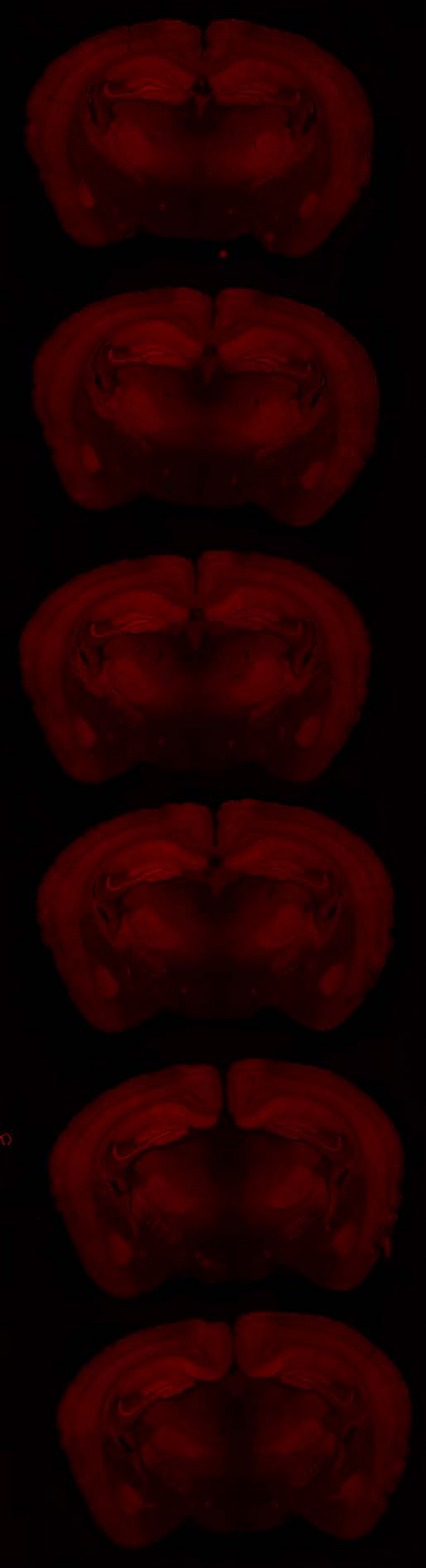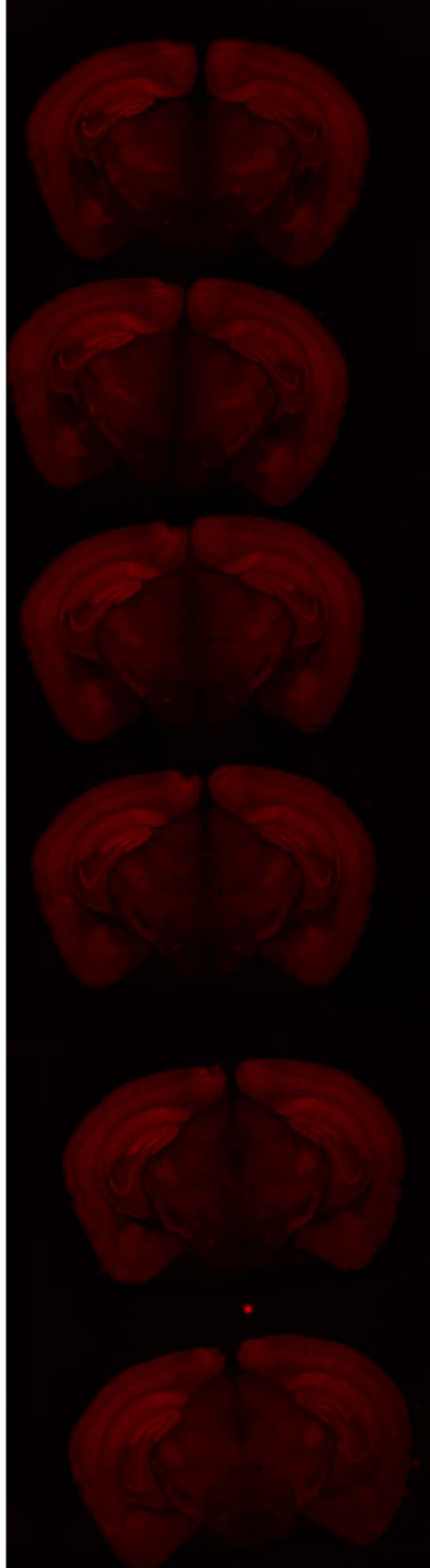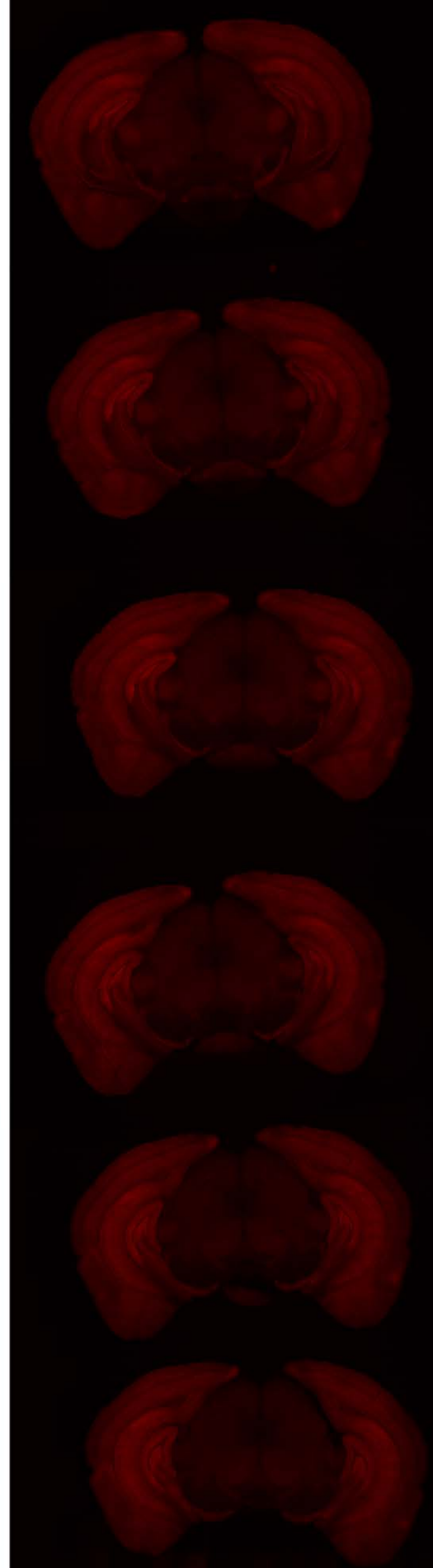

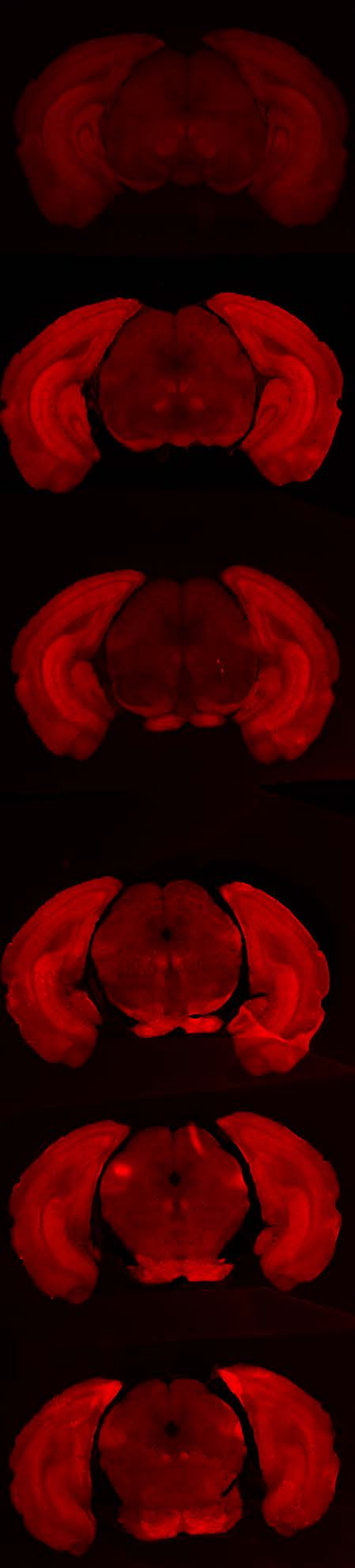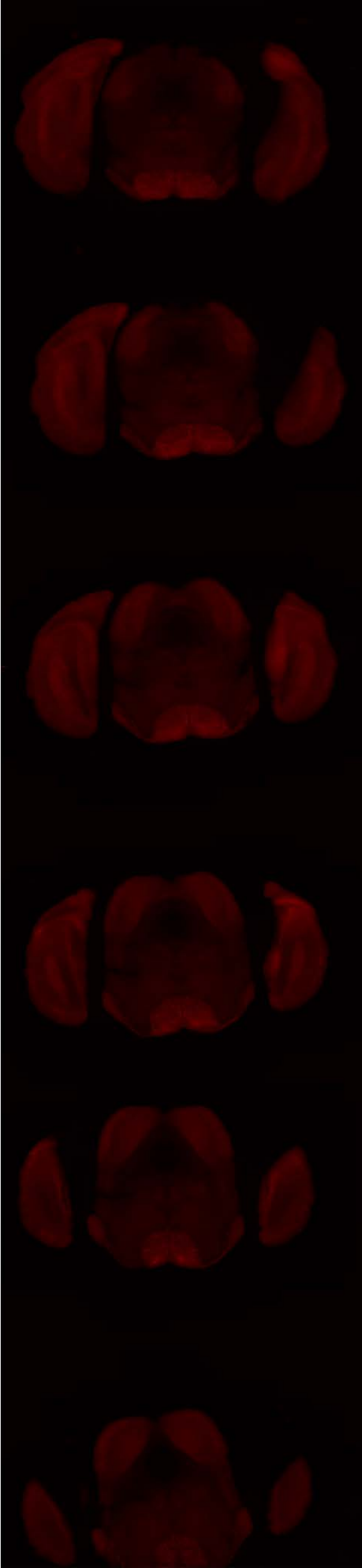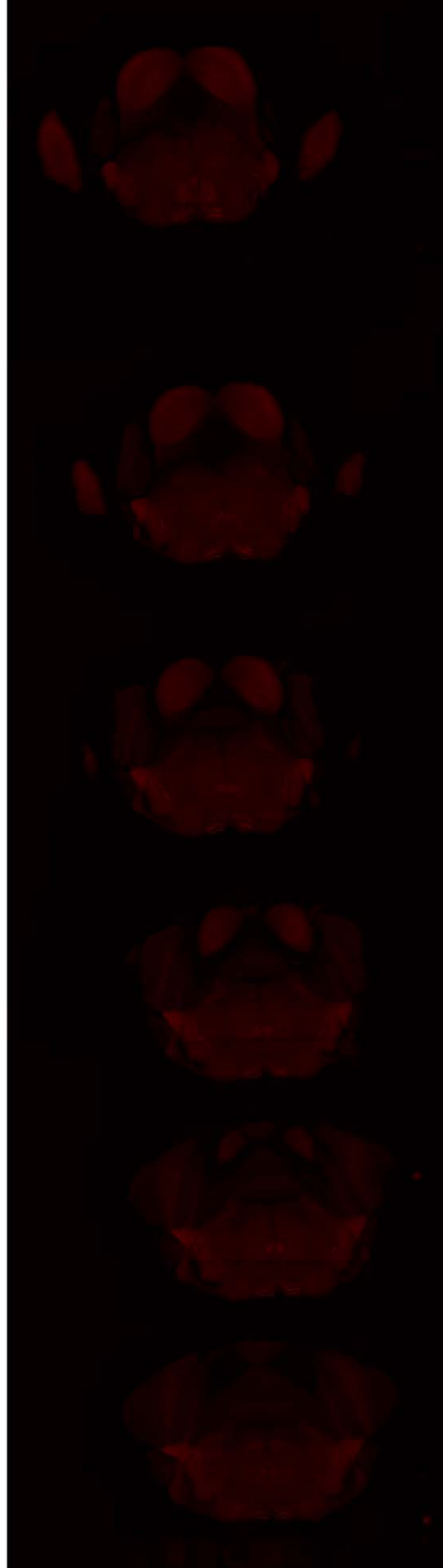

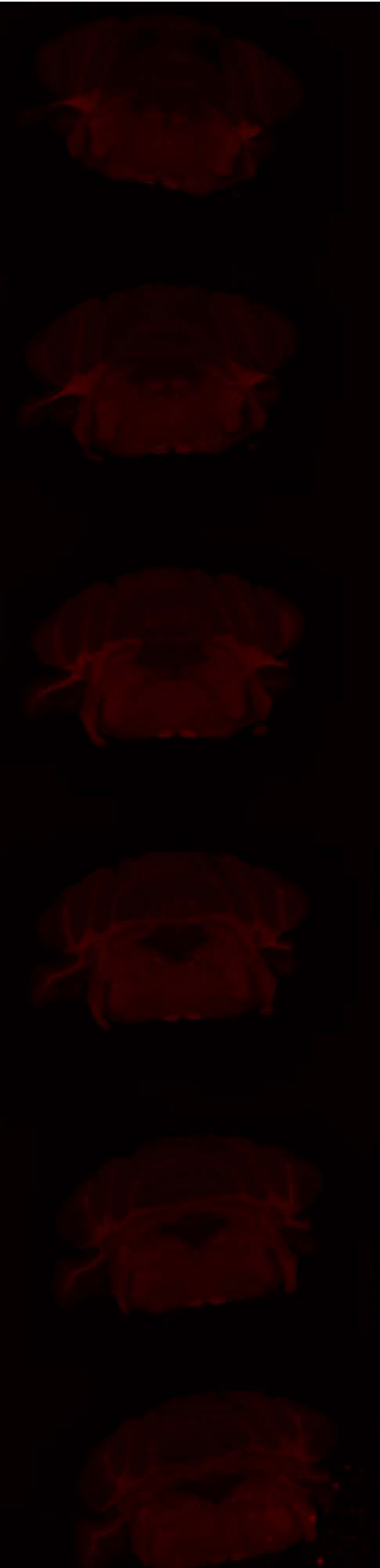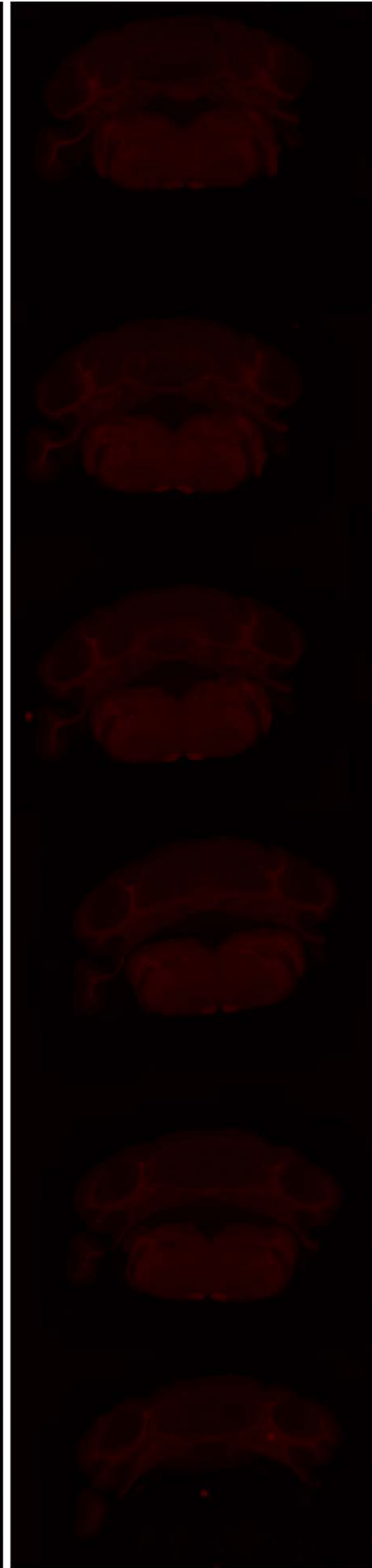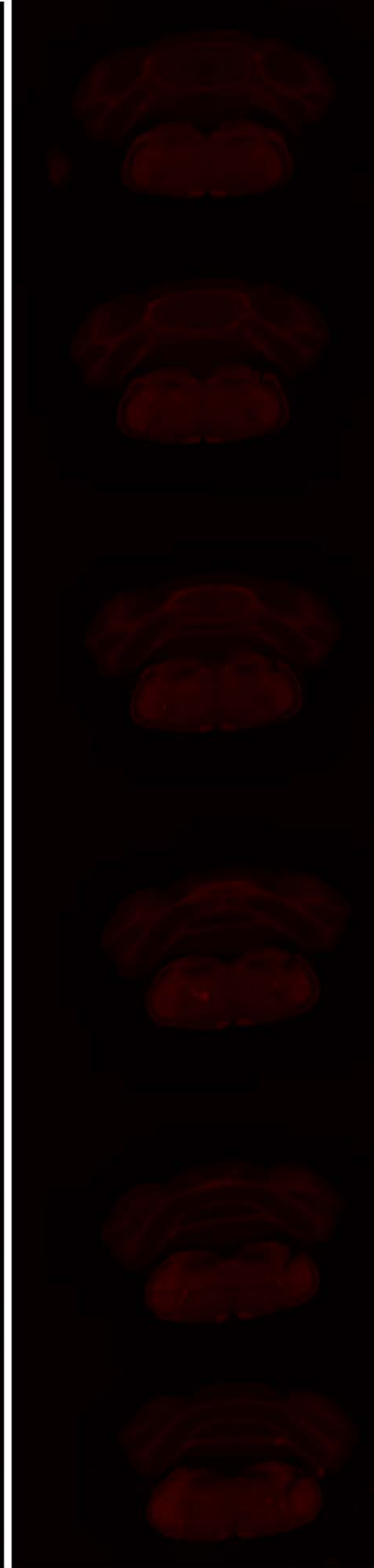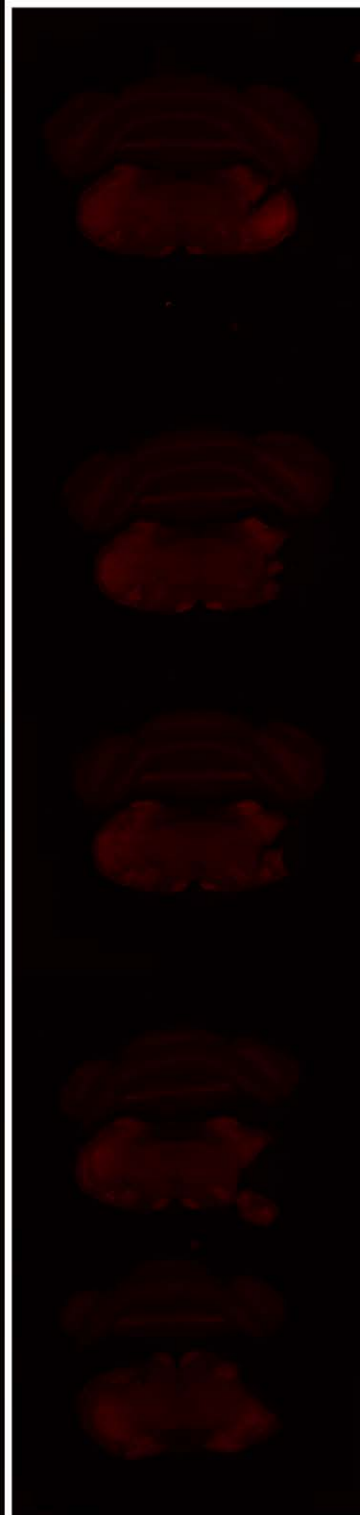

# GP 8.8

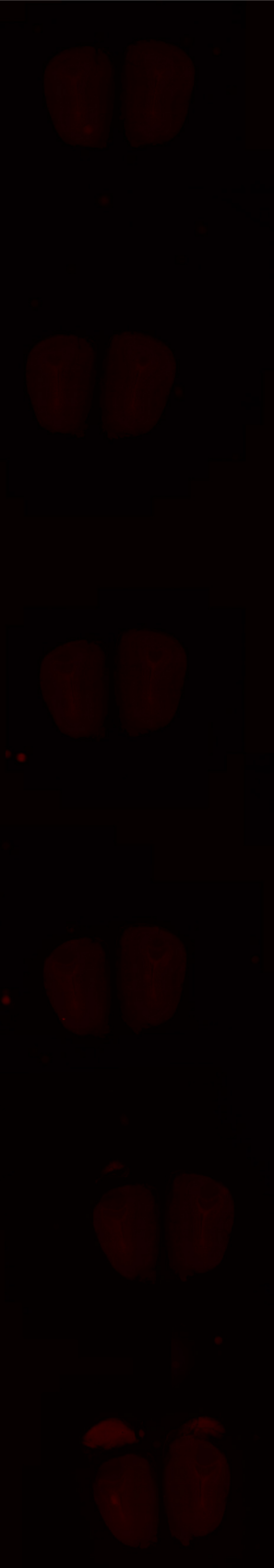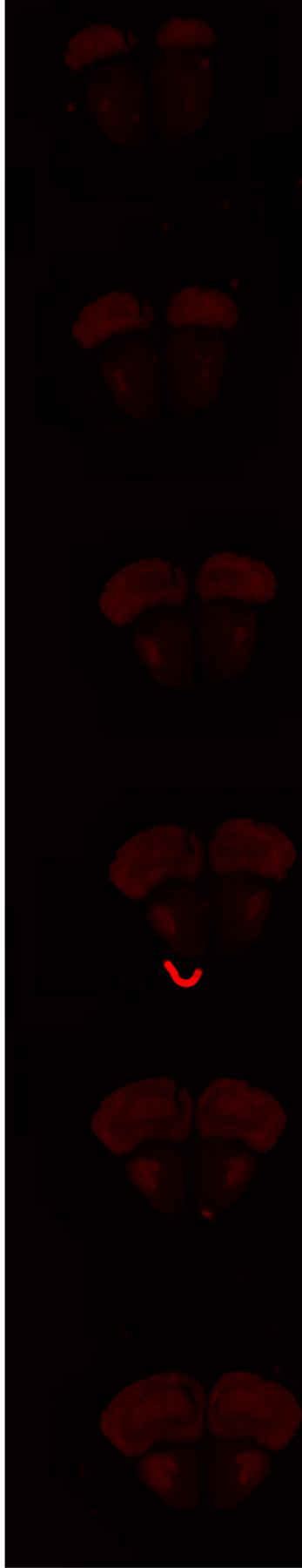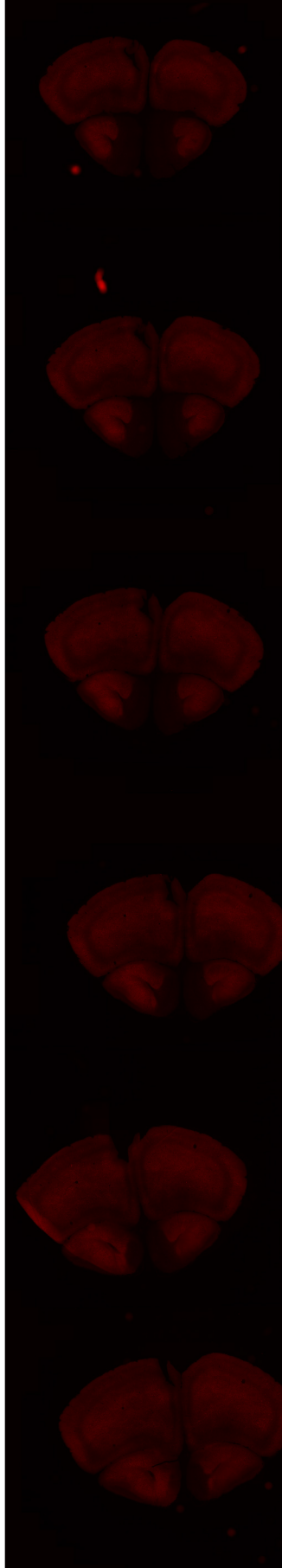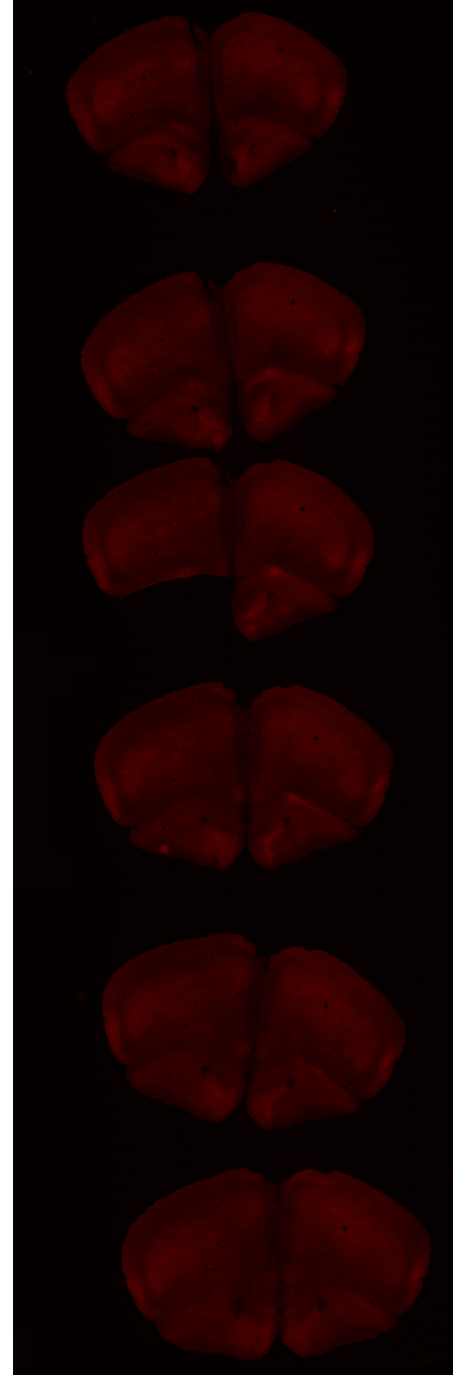

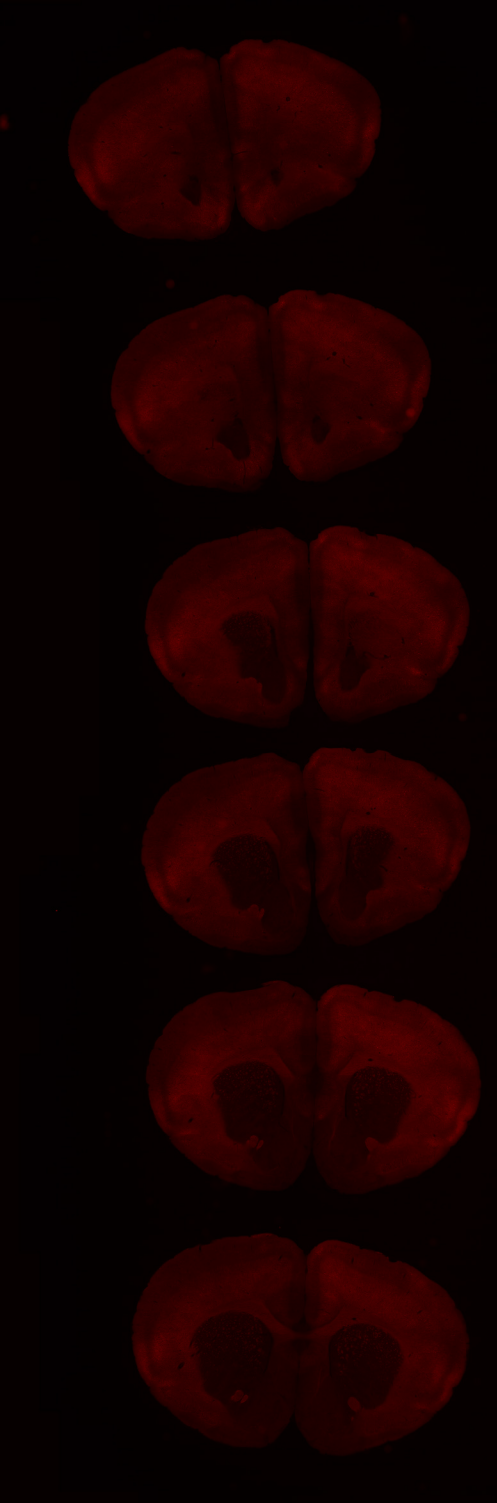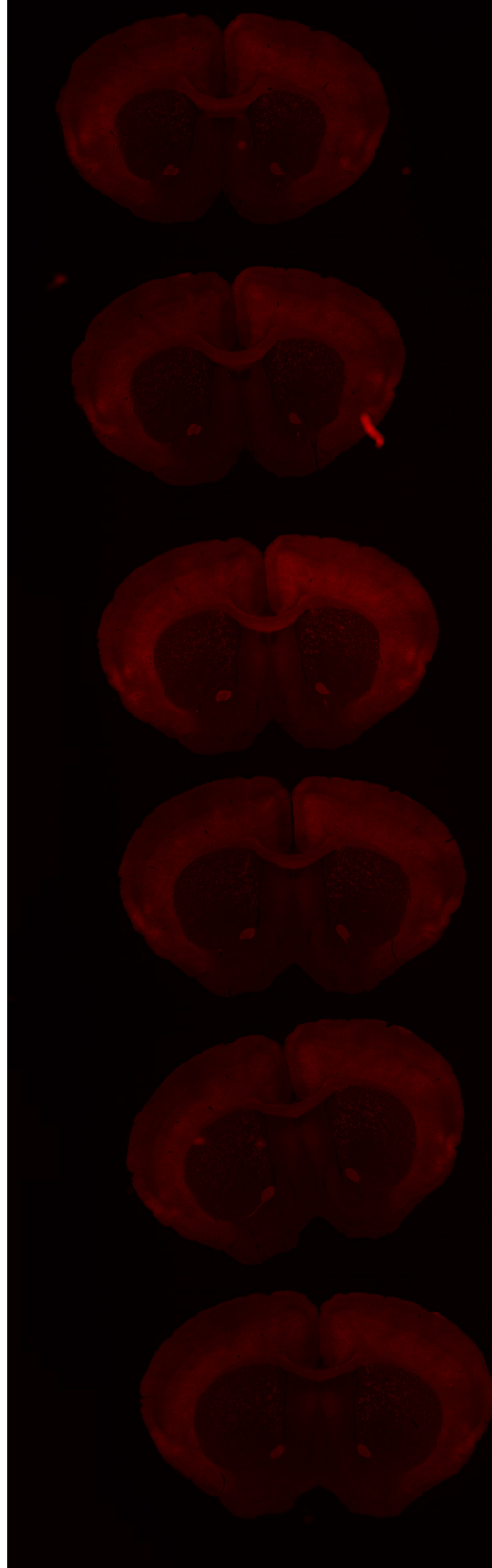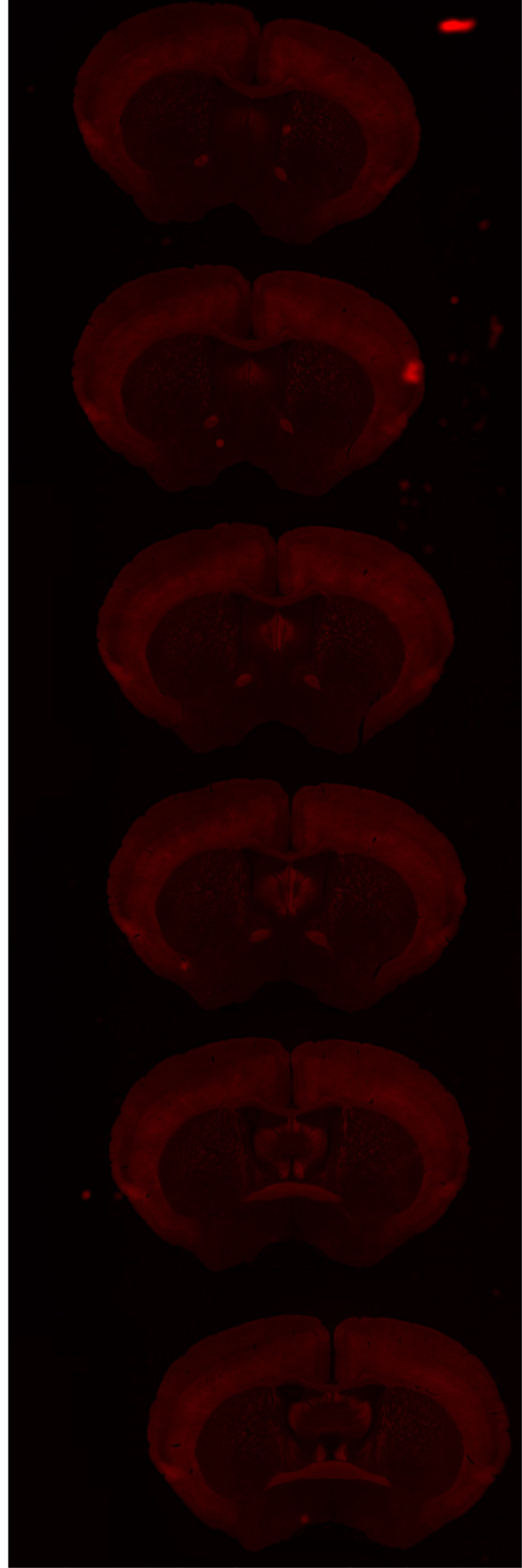

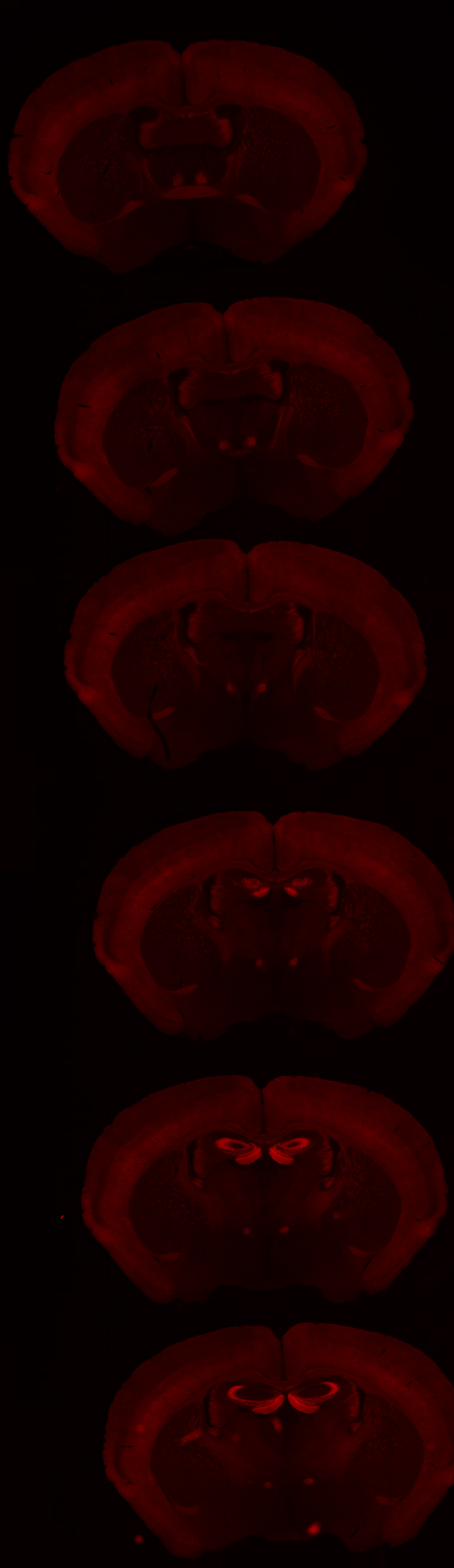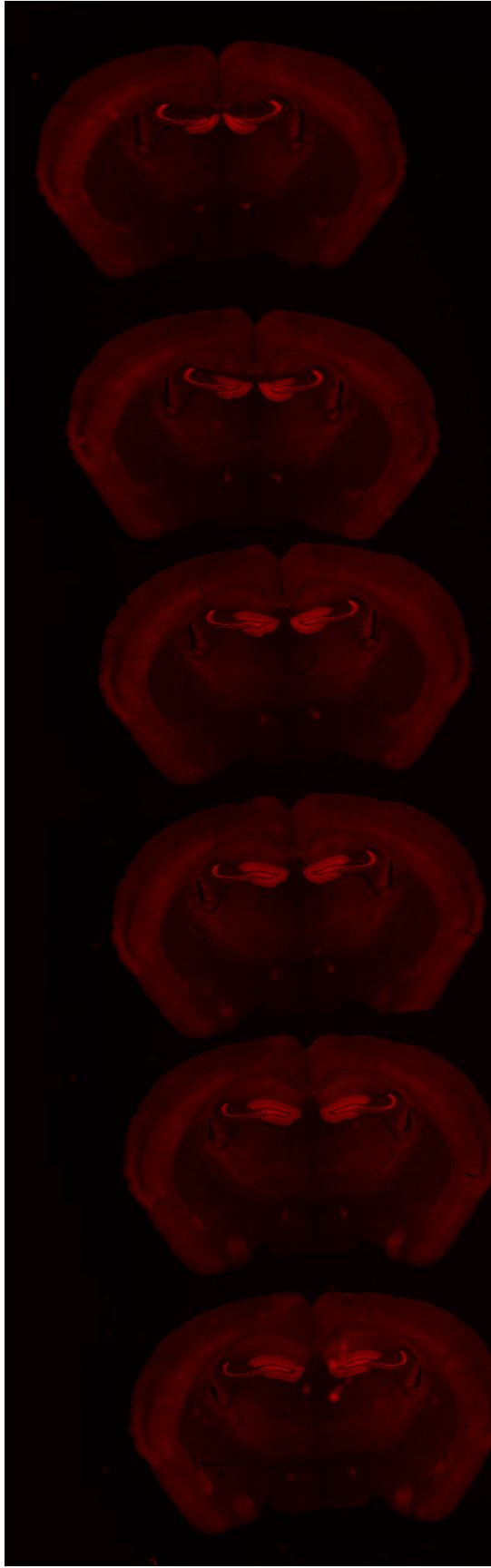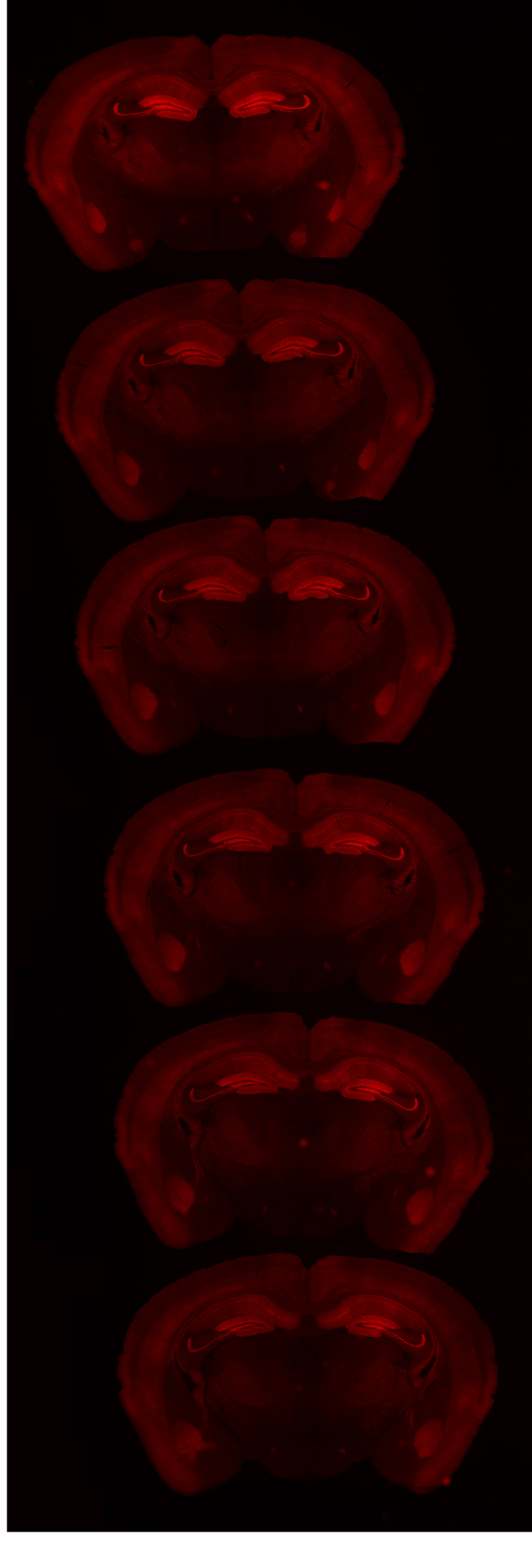

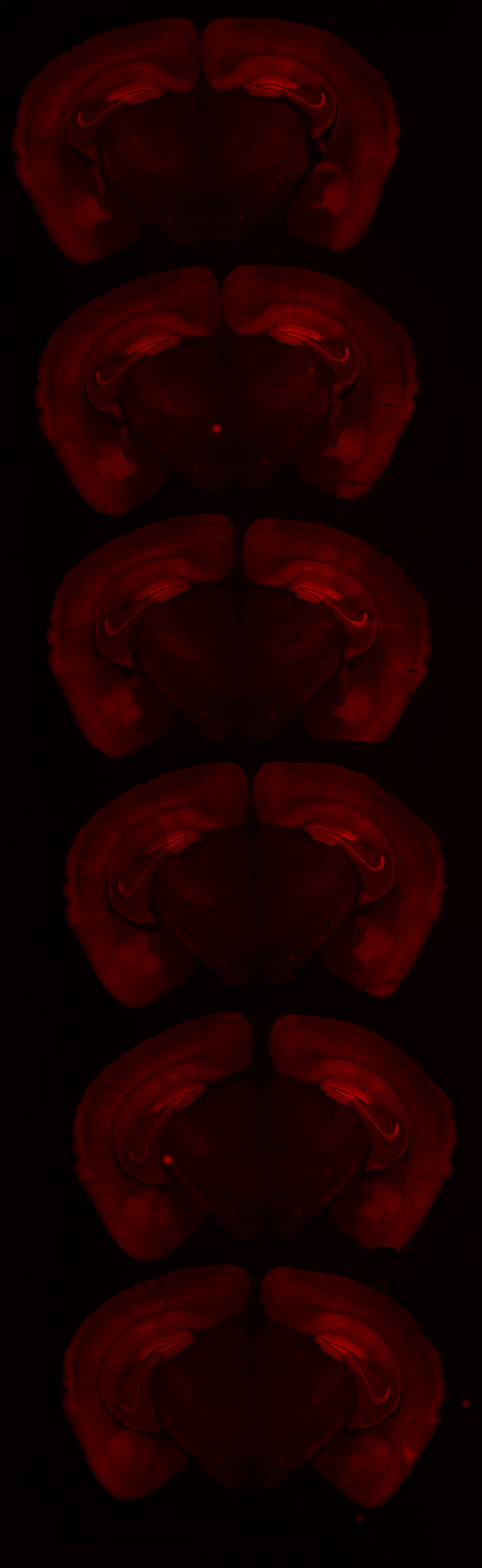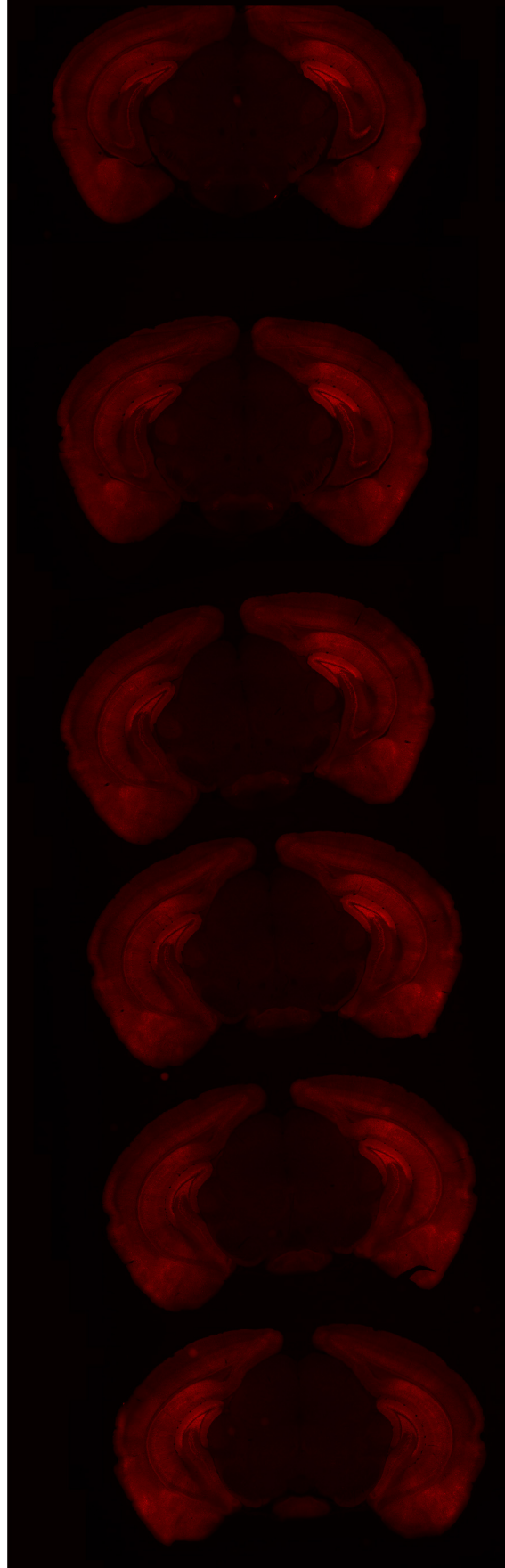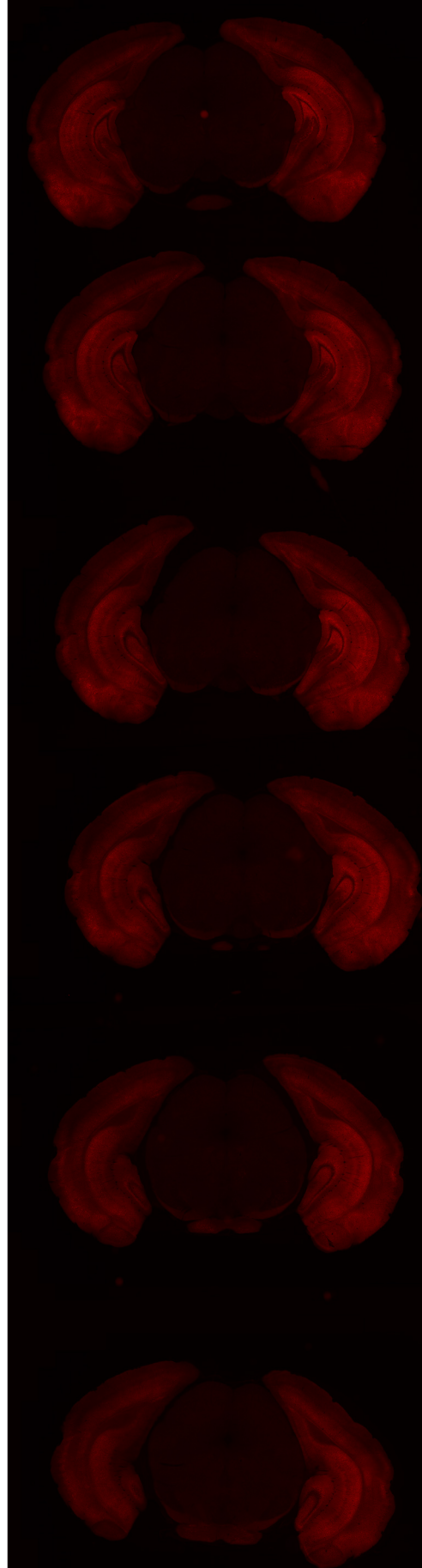

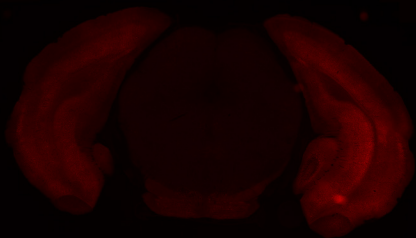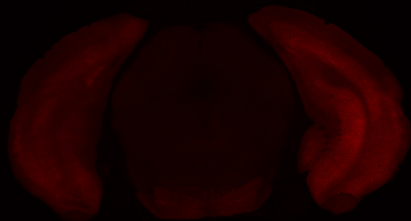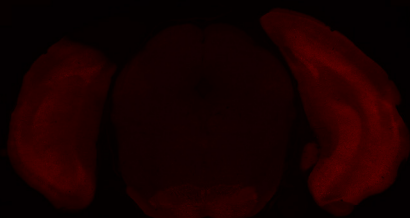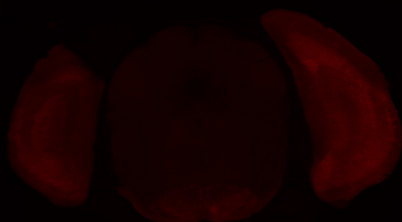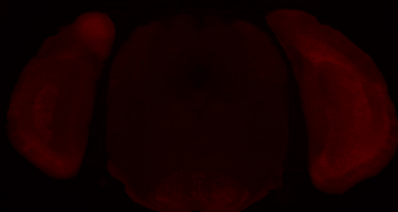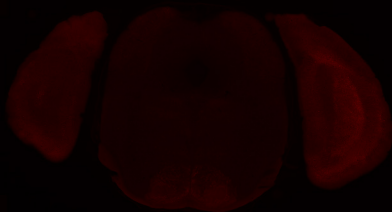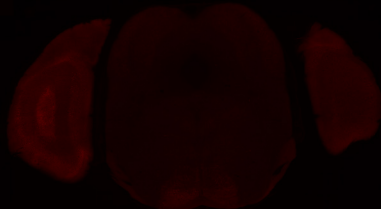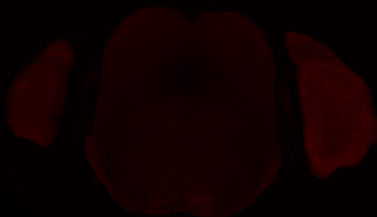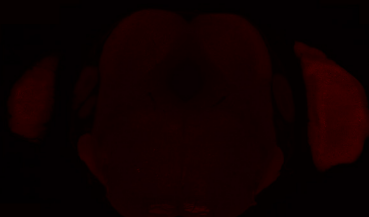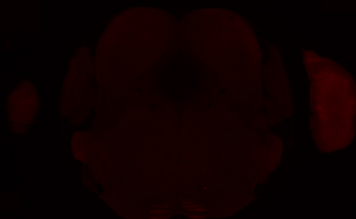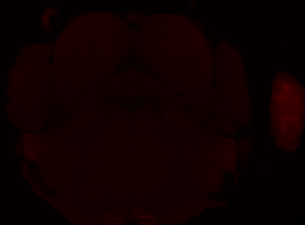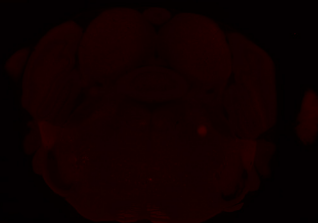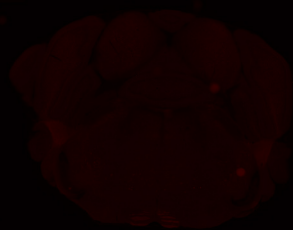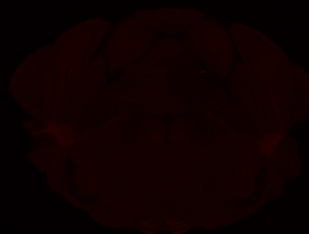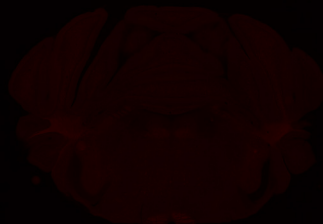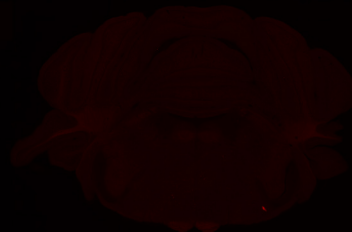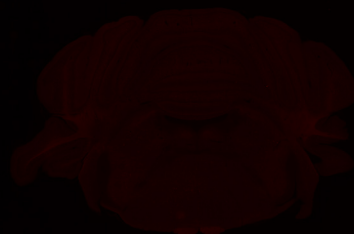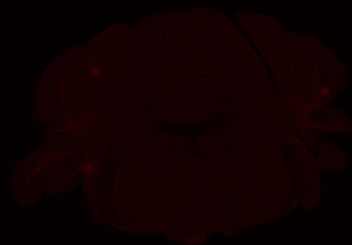

# GP 8.20

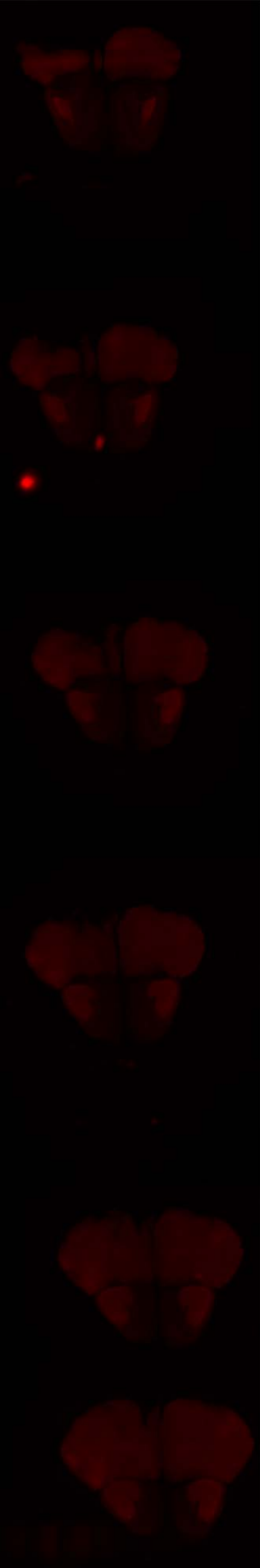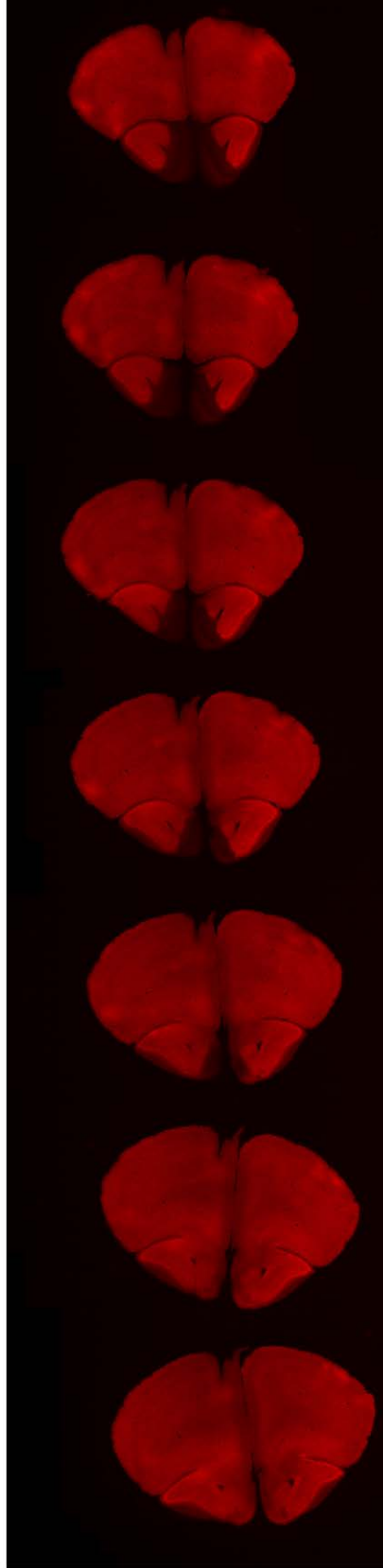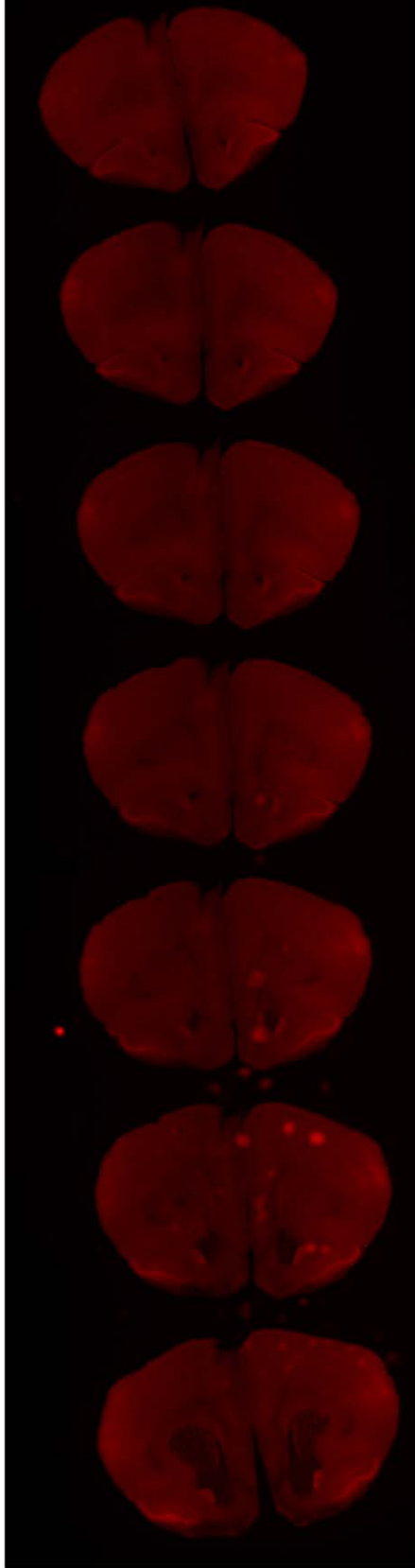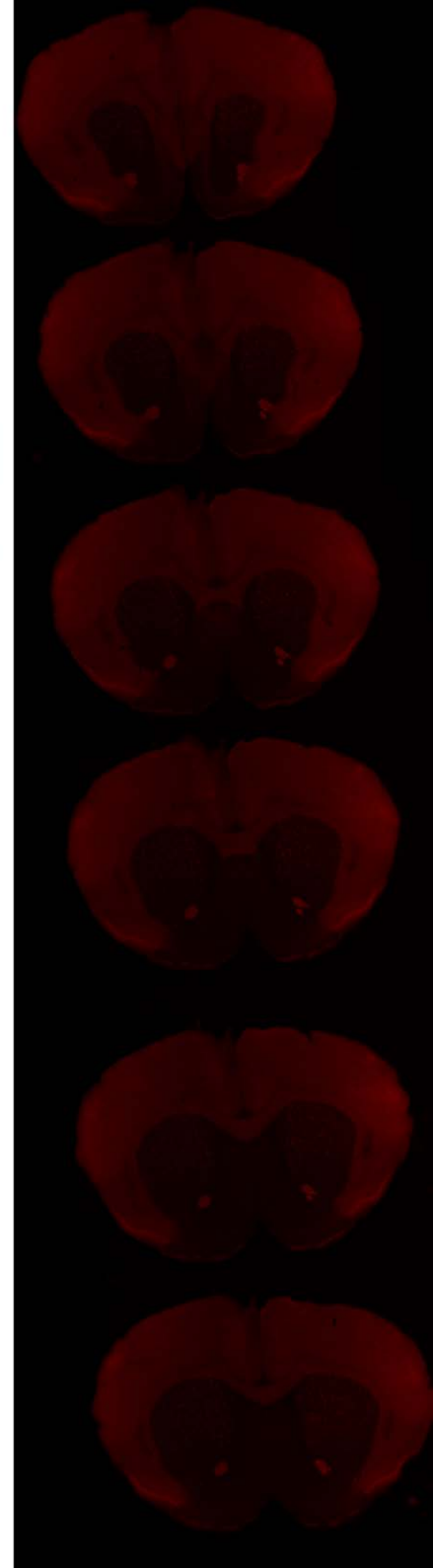

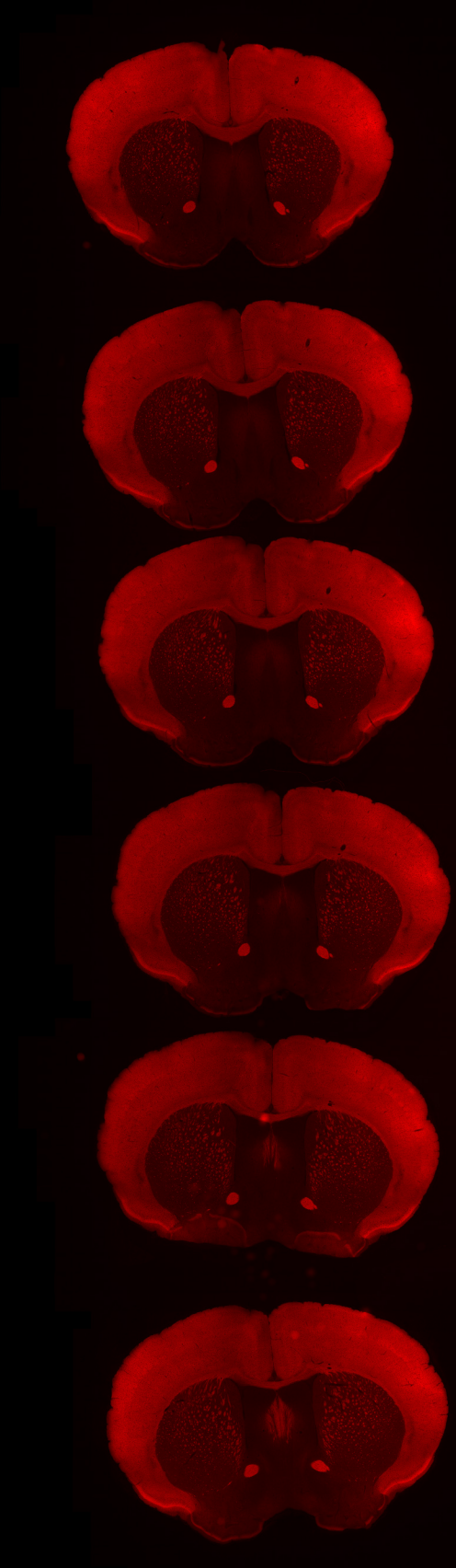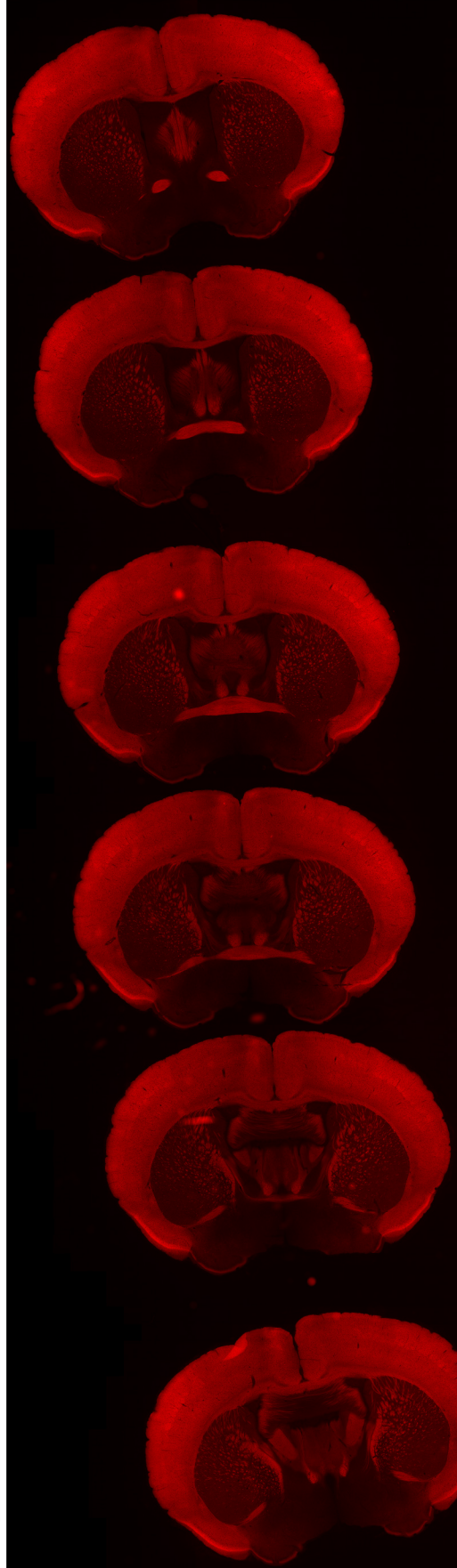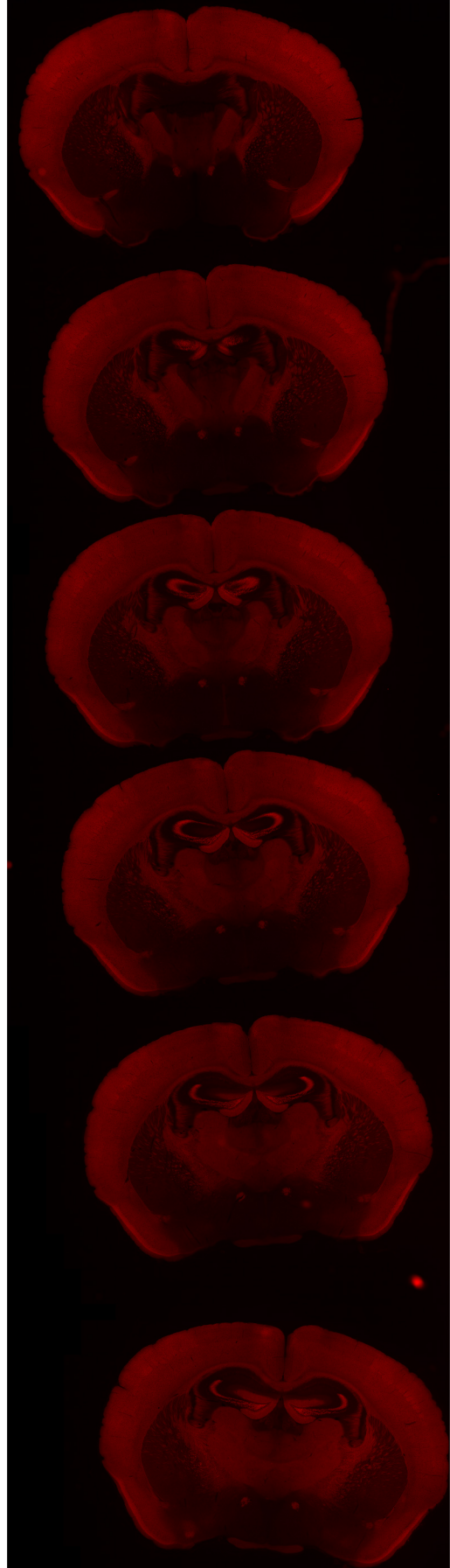

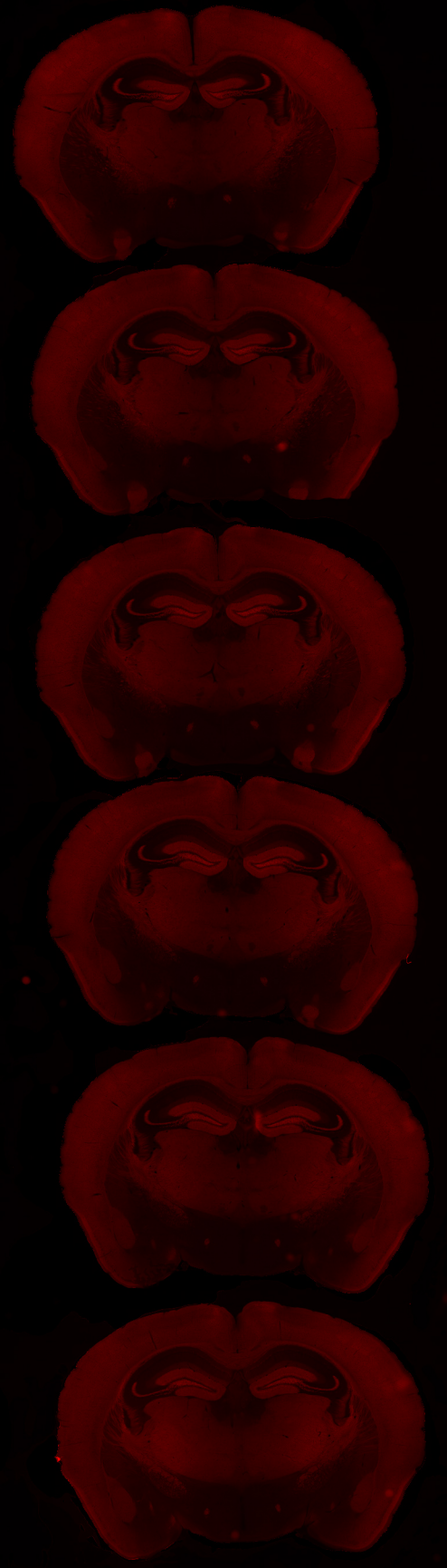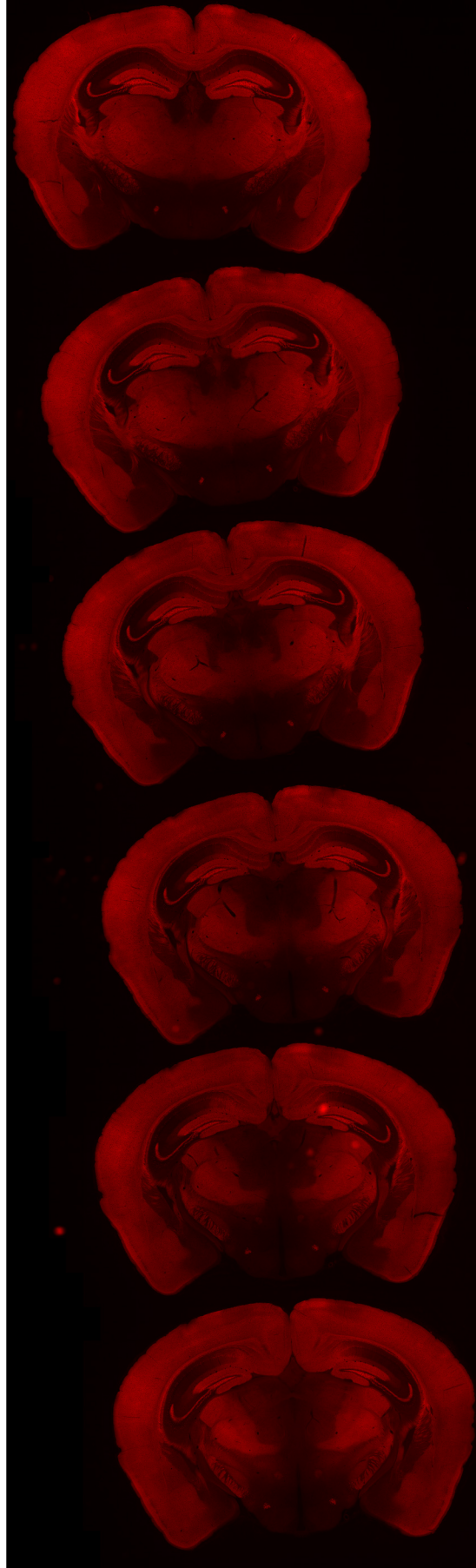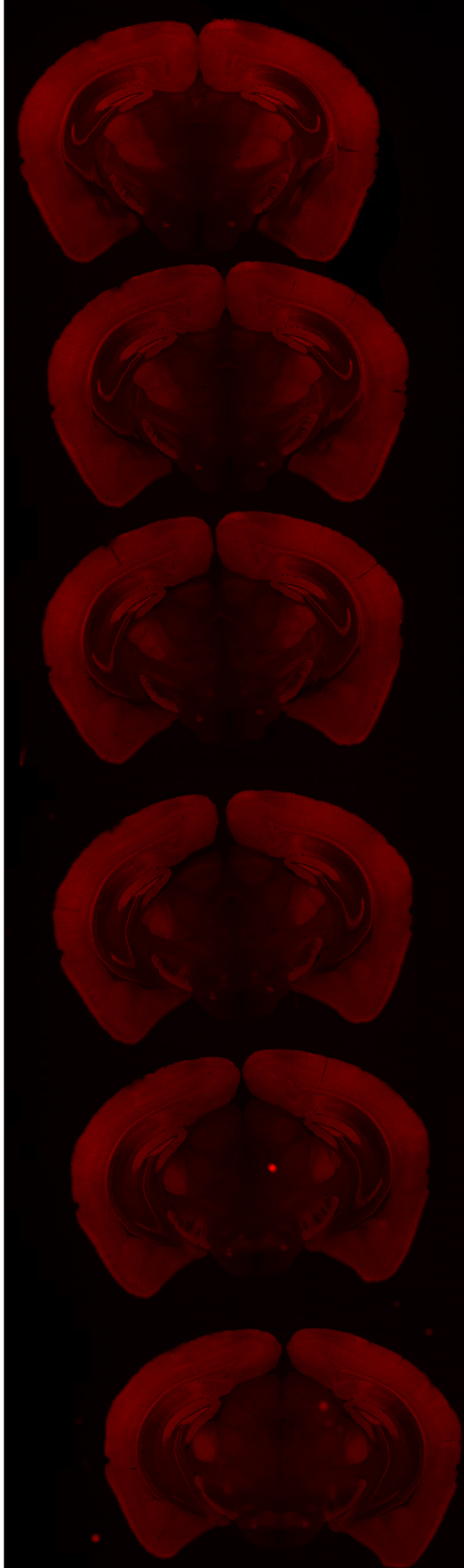

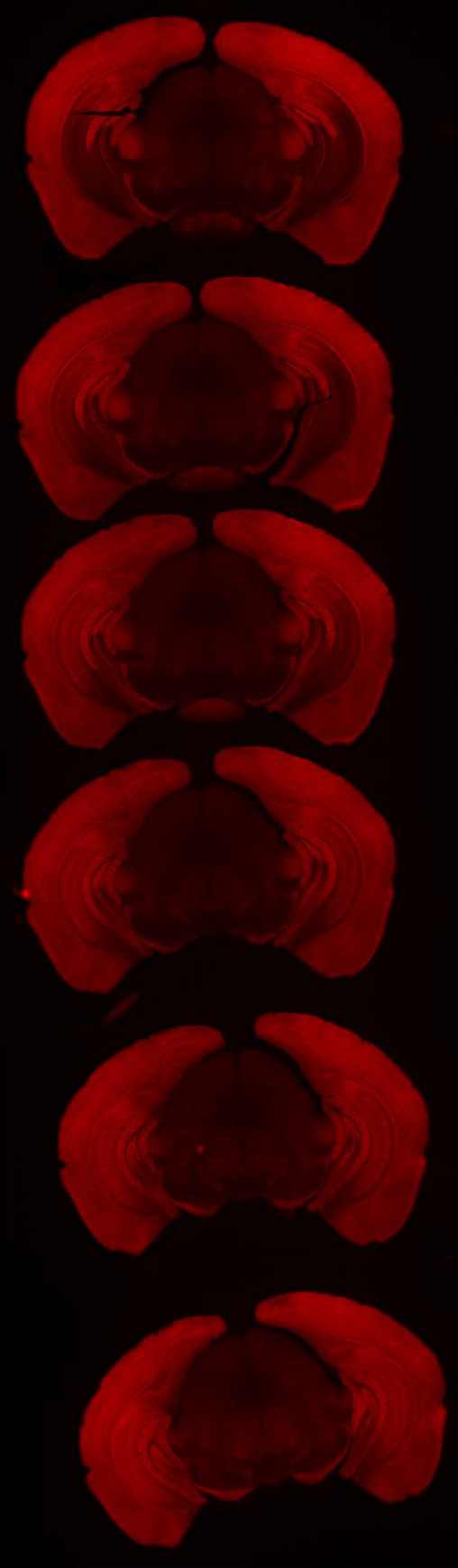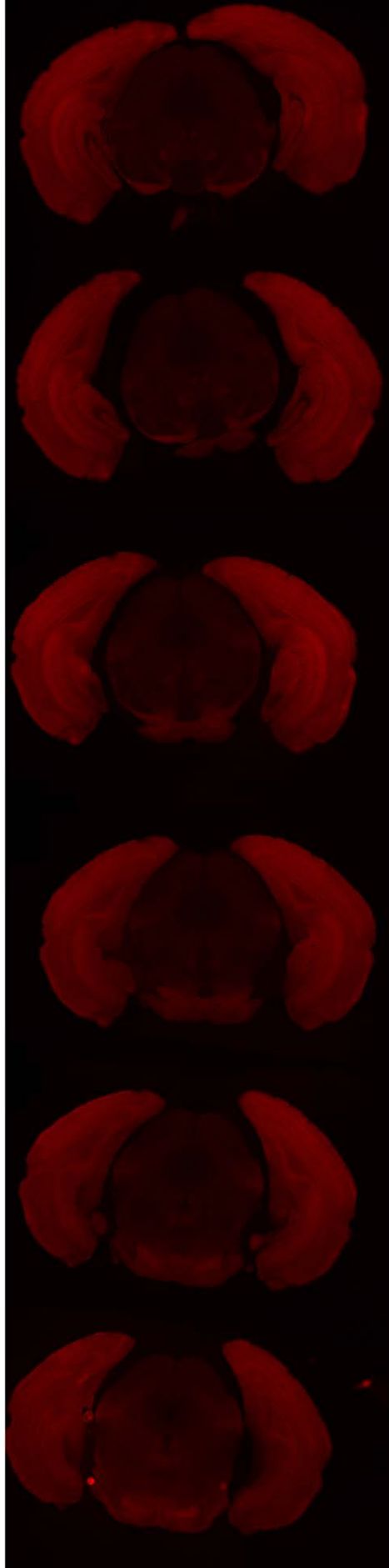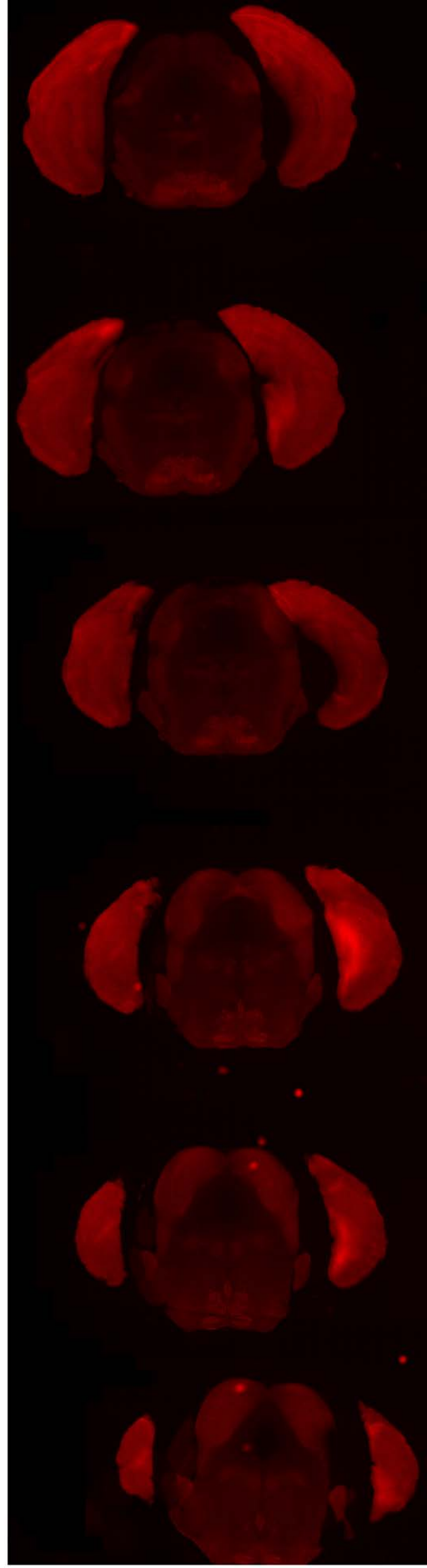

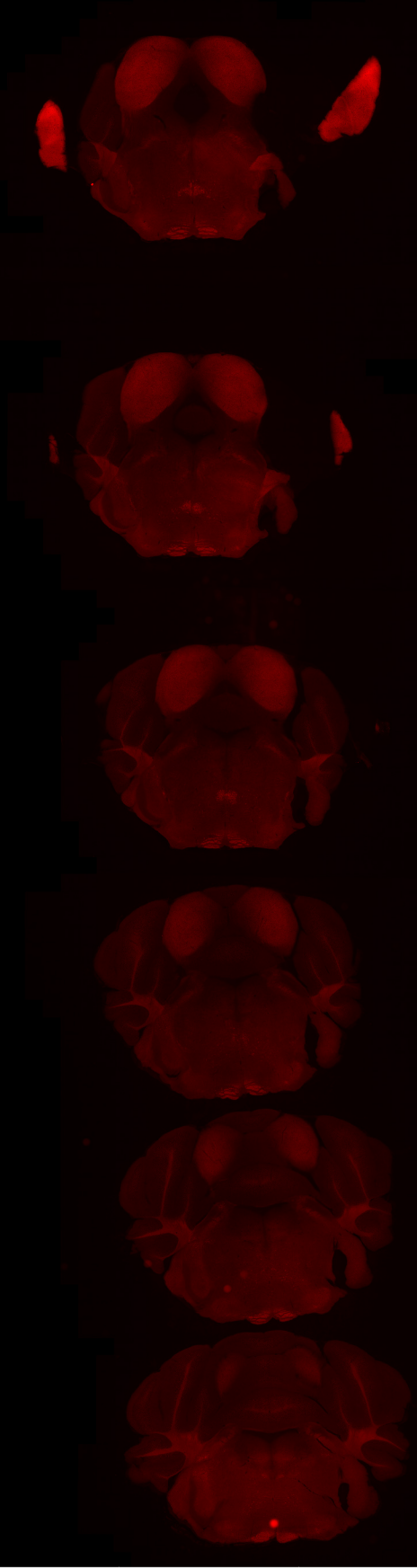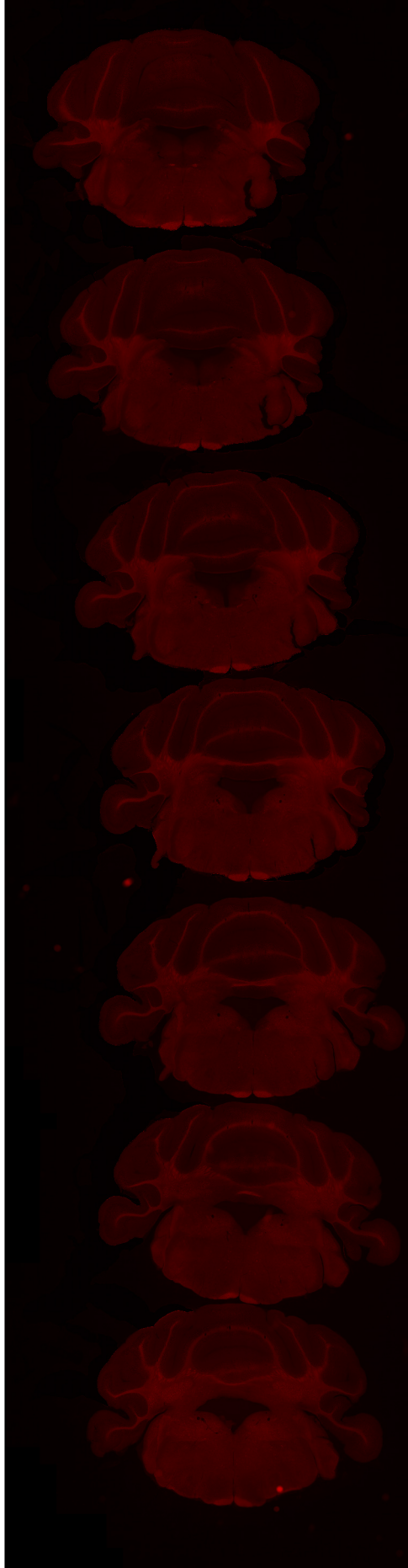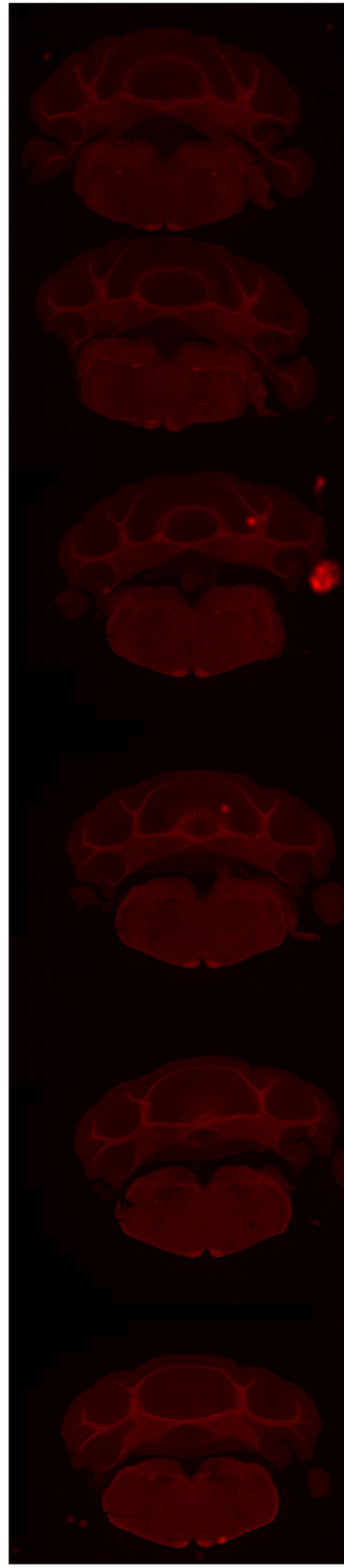

**GP 8.24**

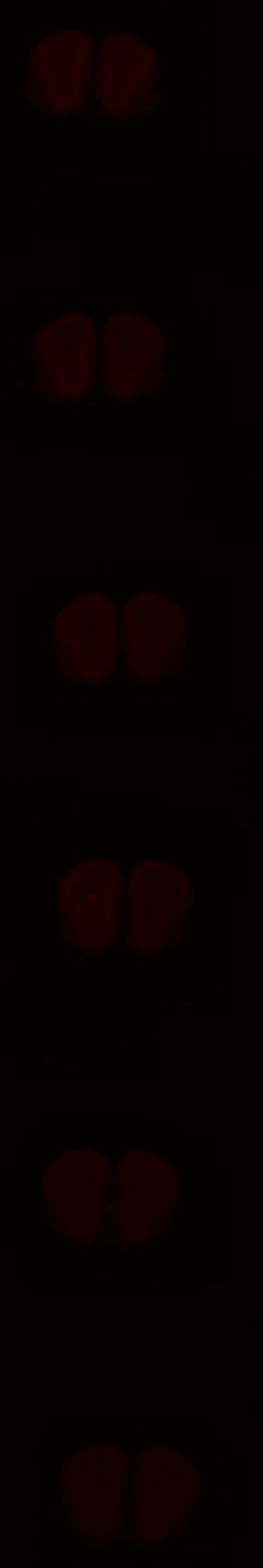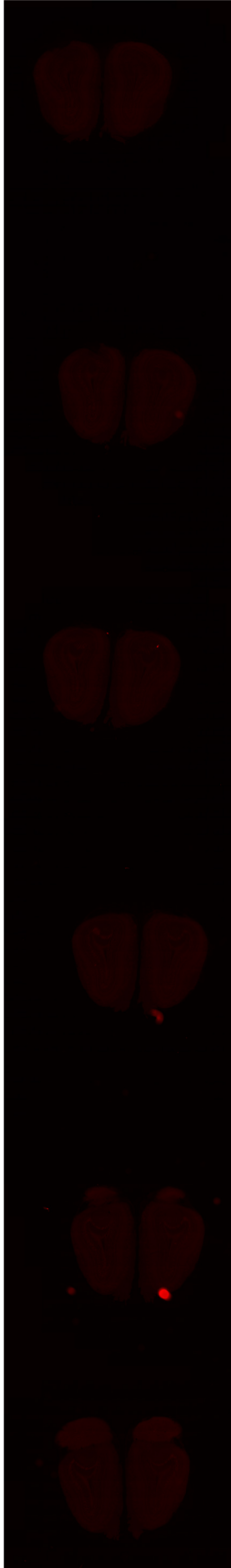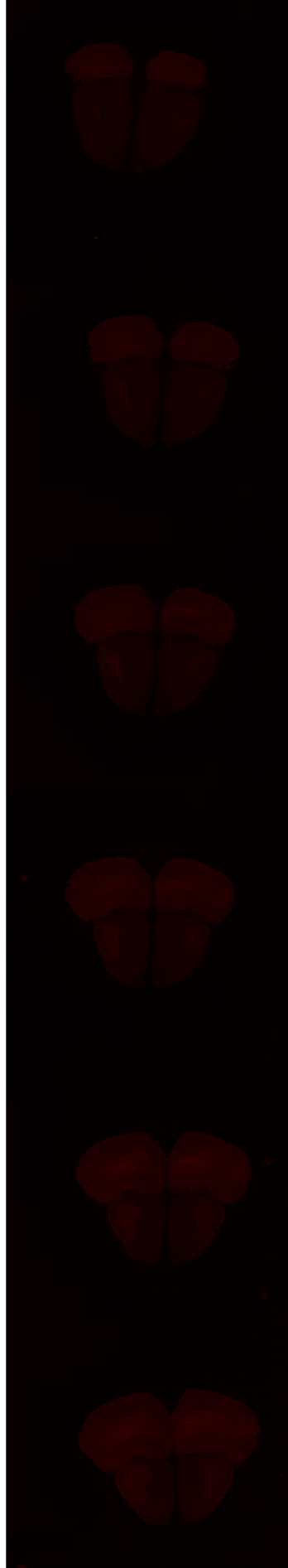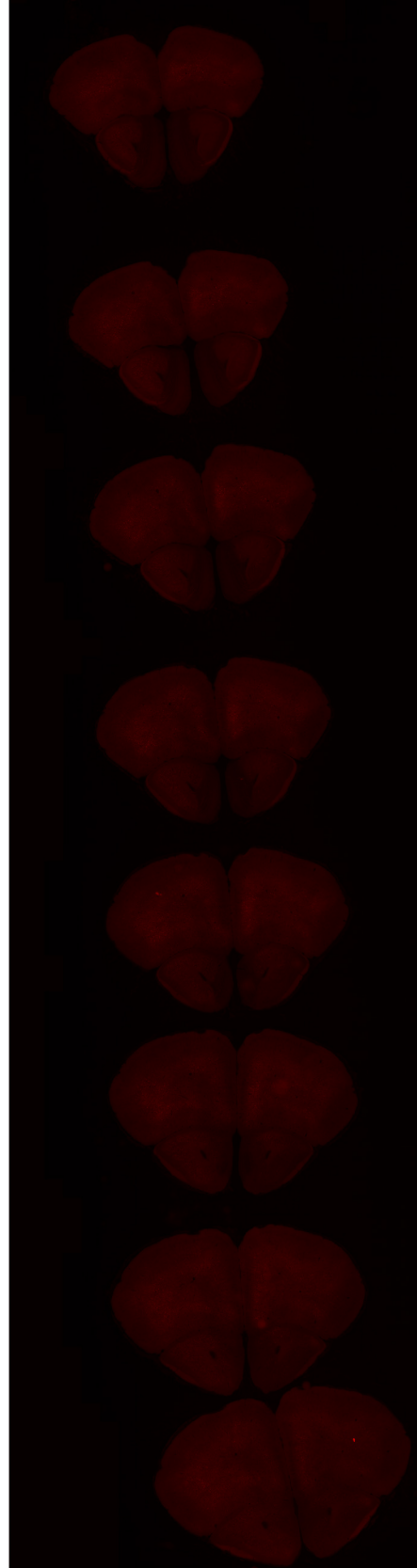

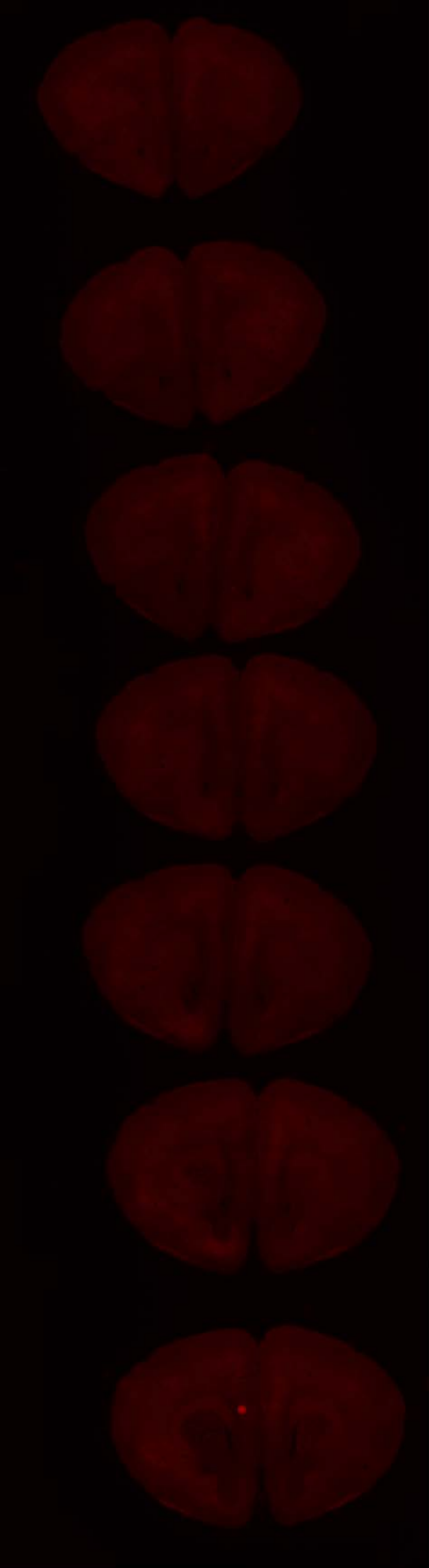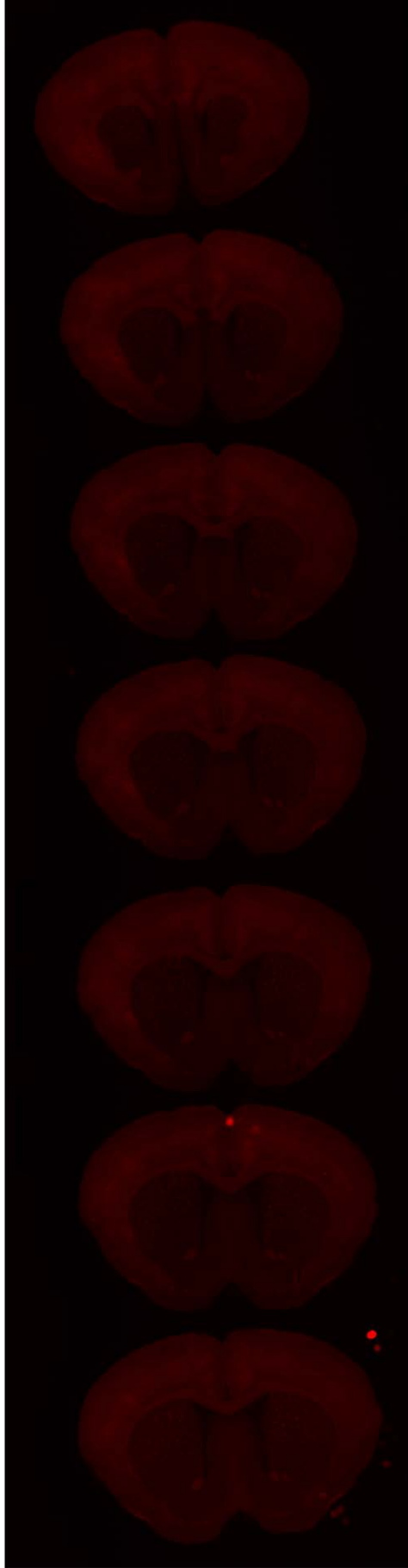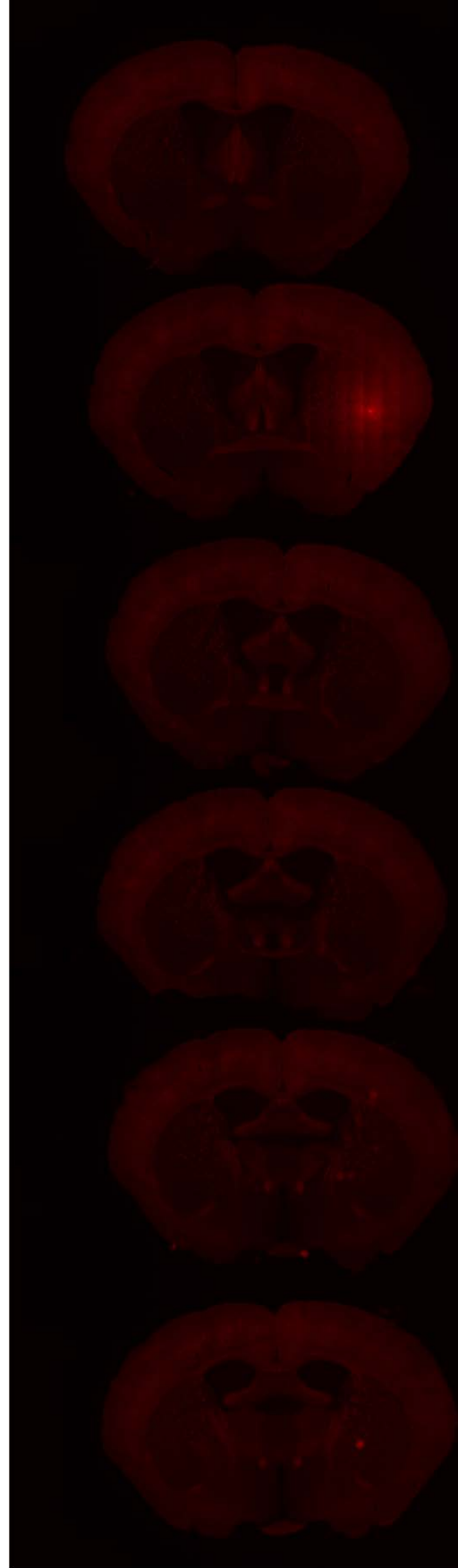

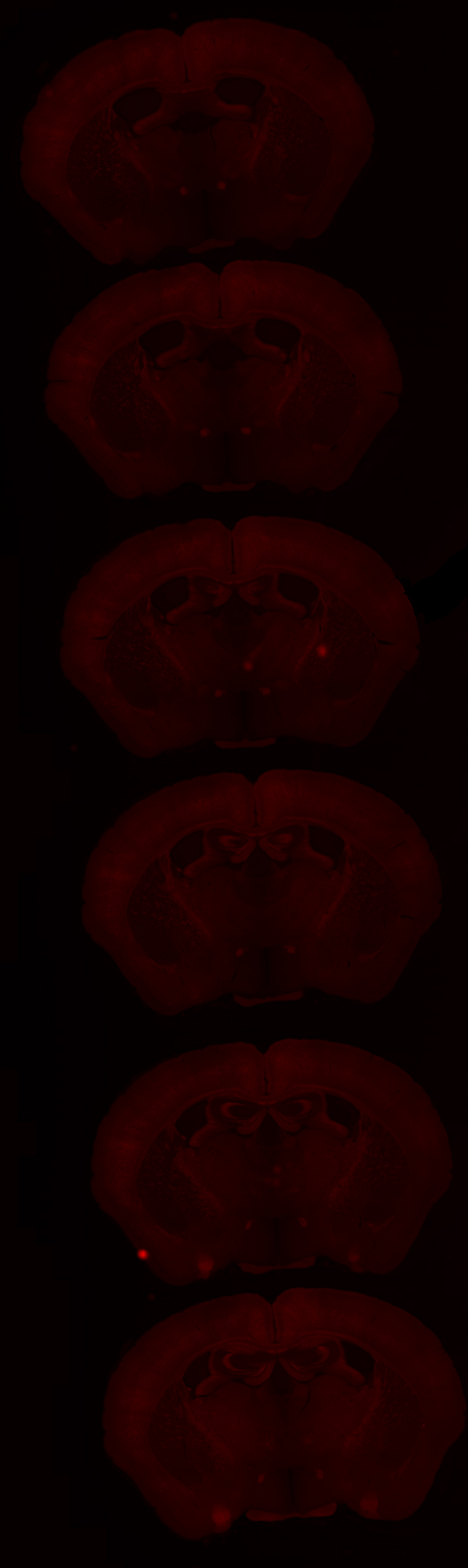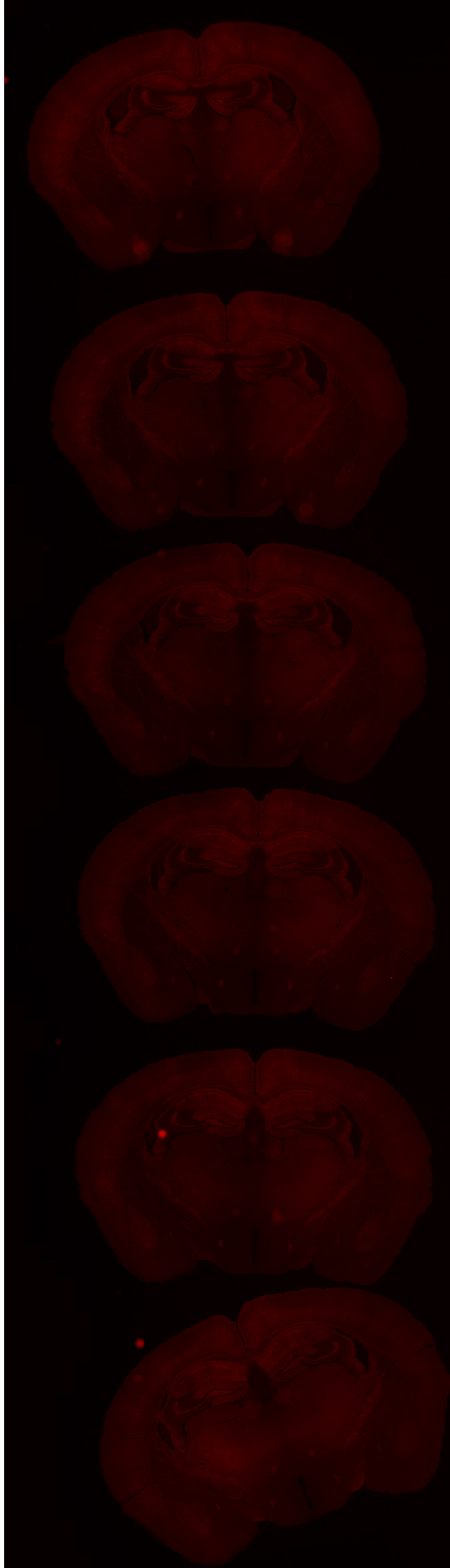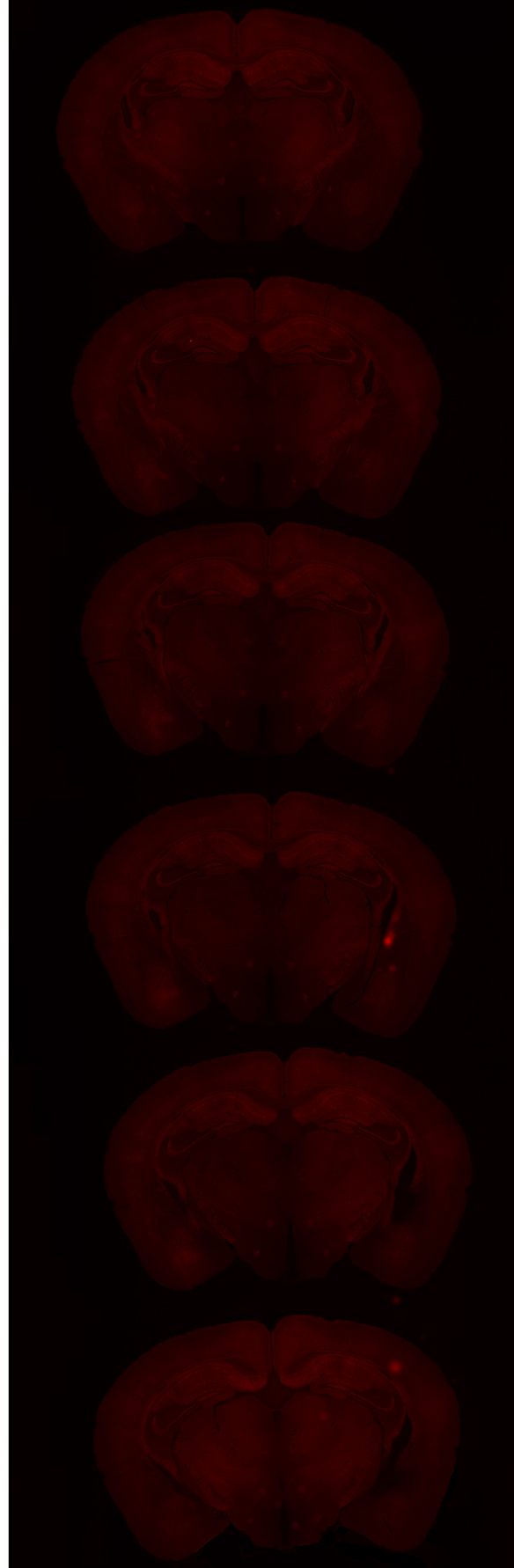

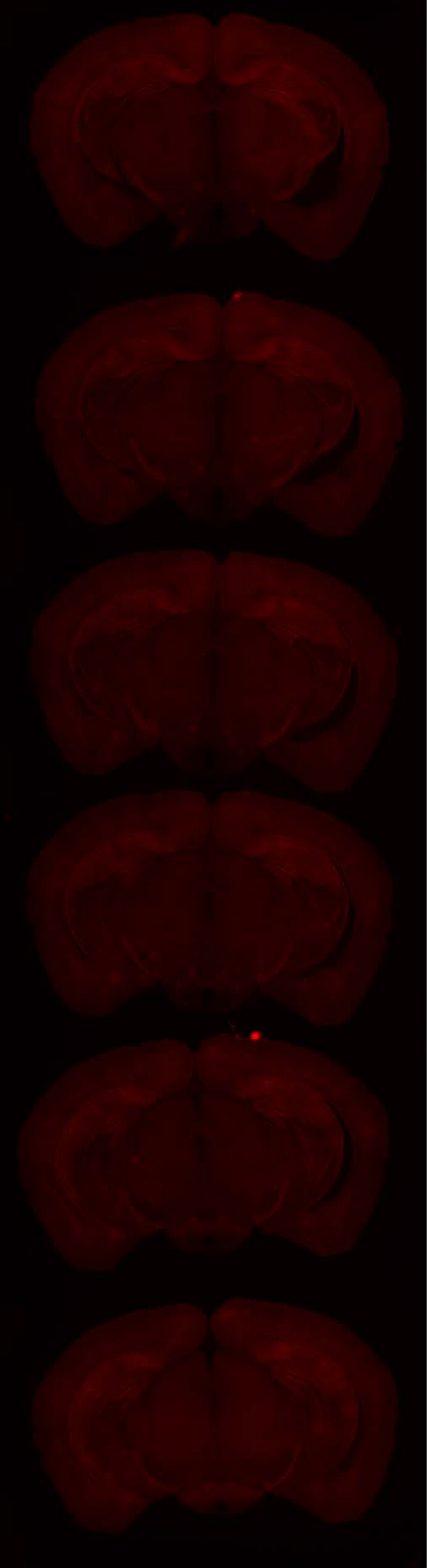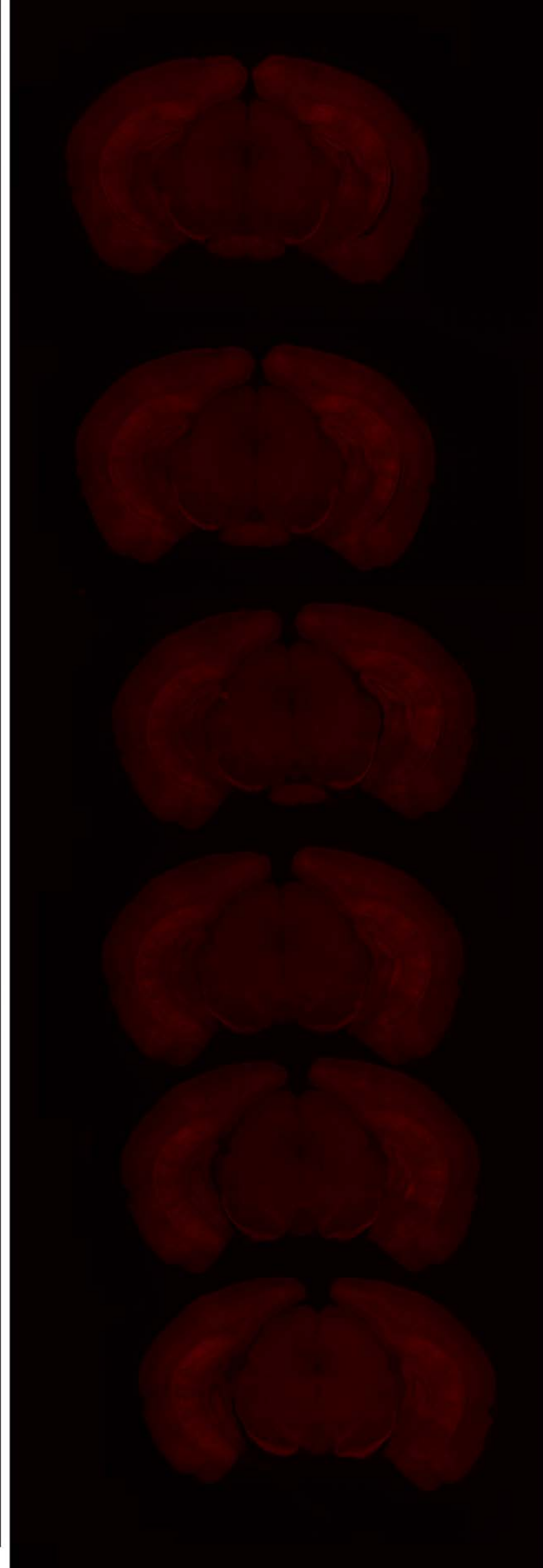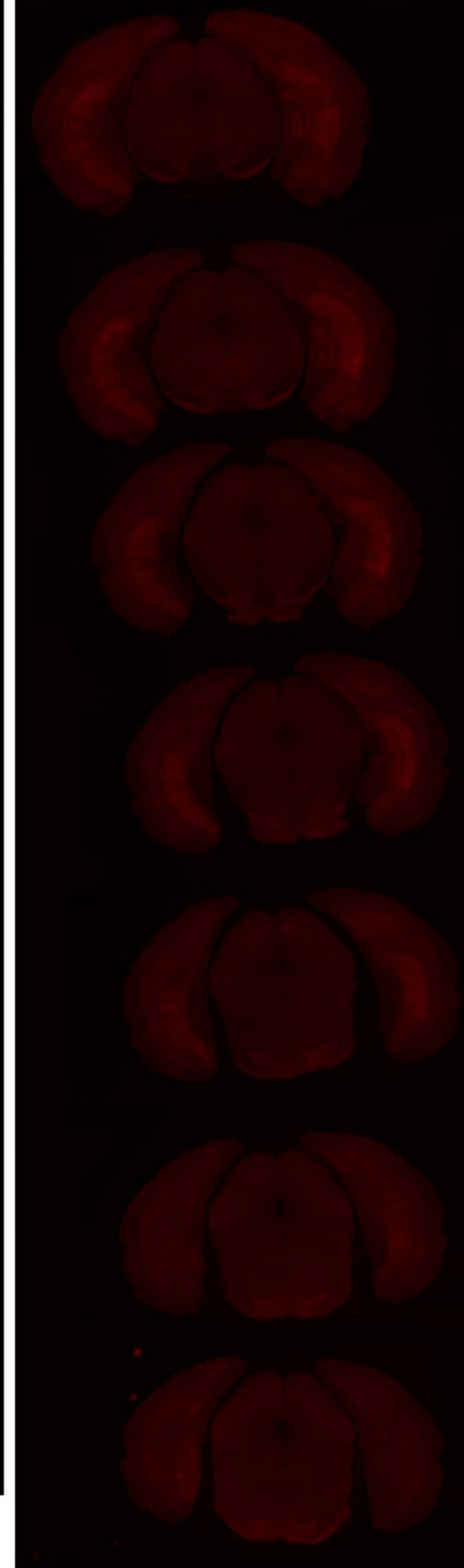

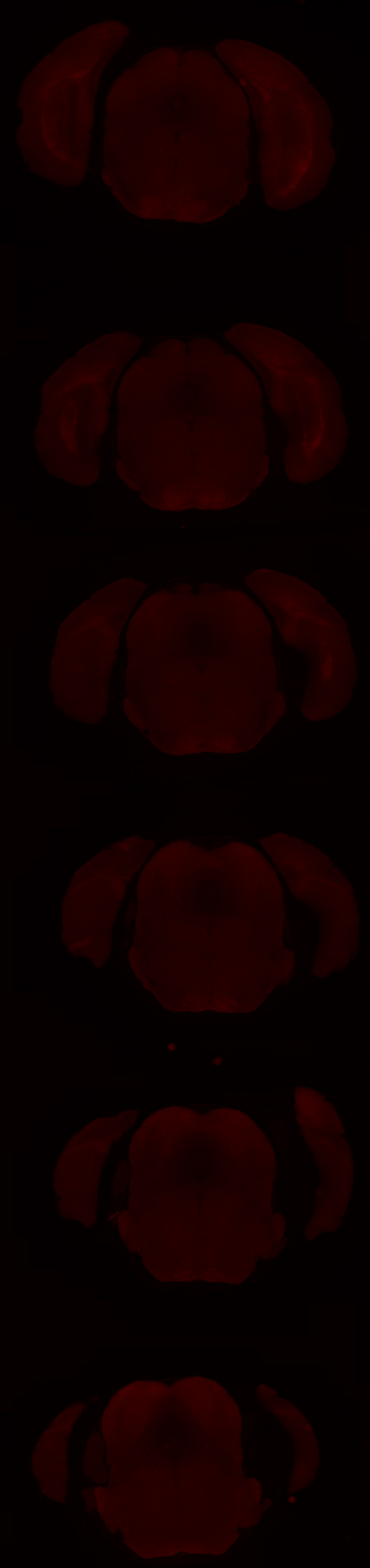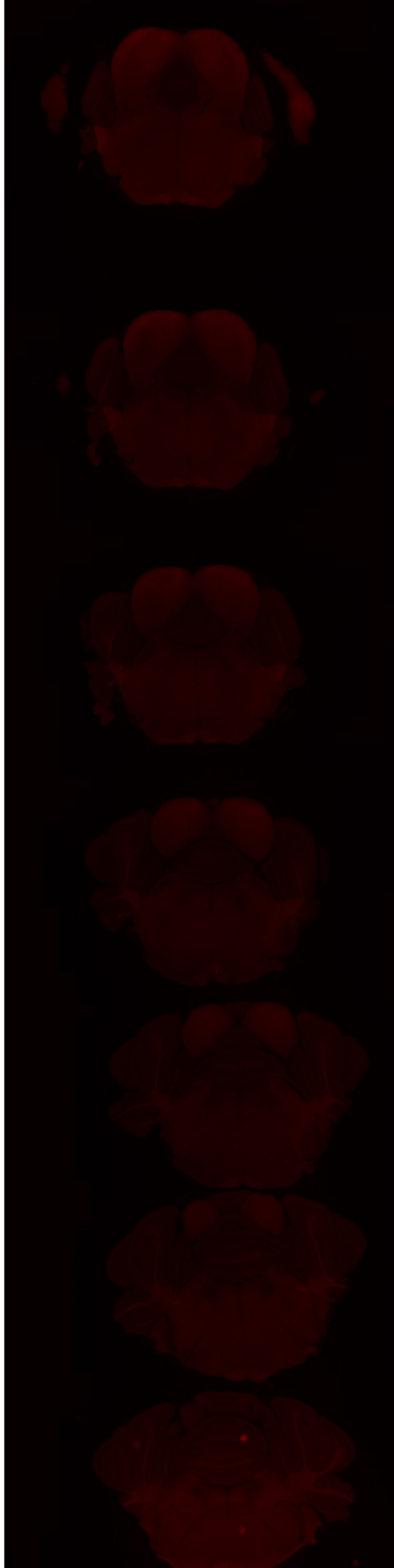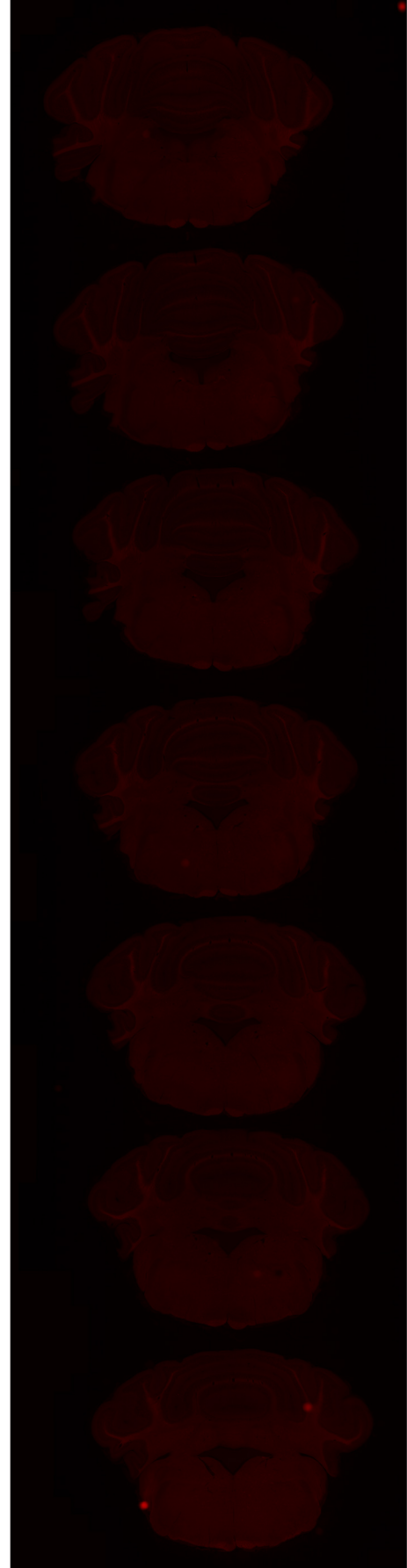

**GP 8.26**

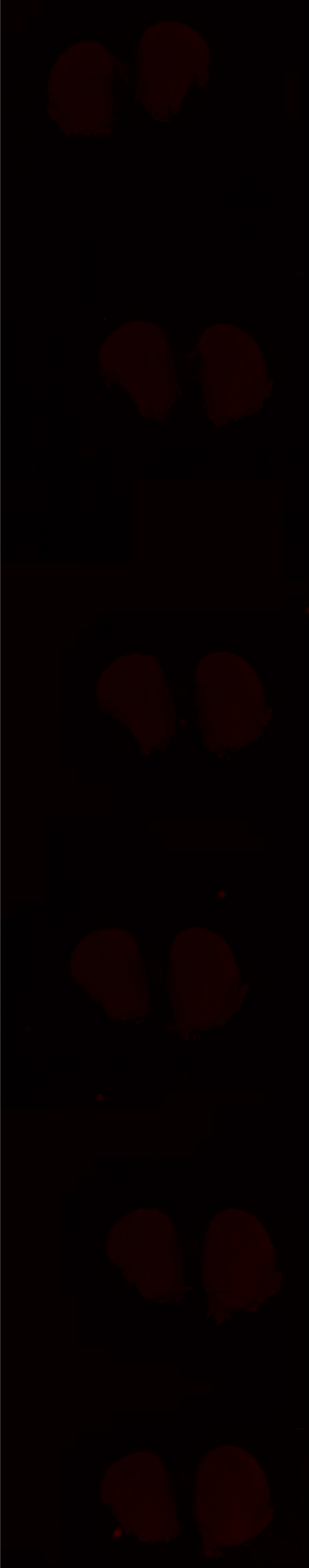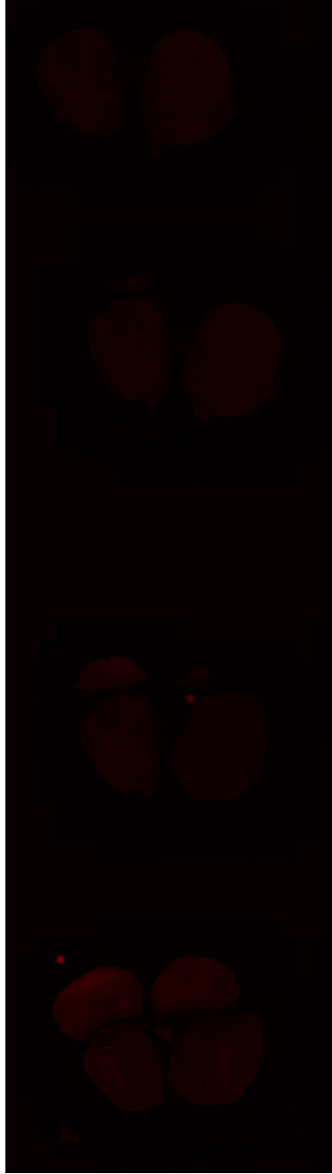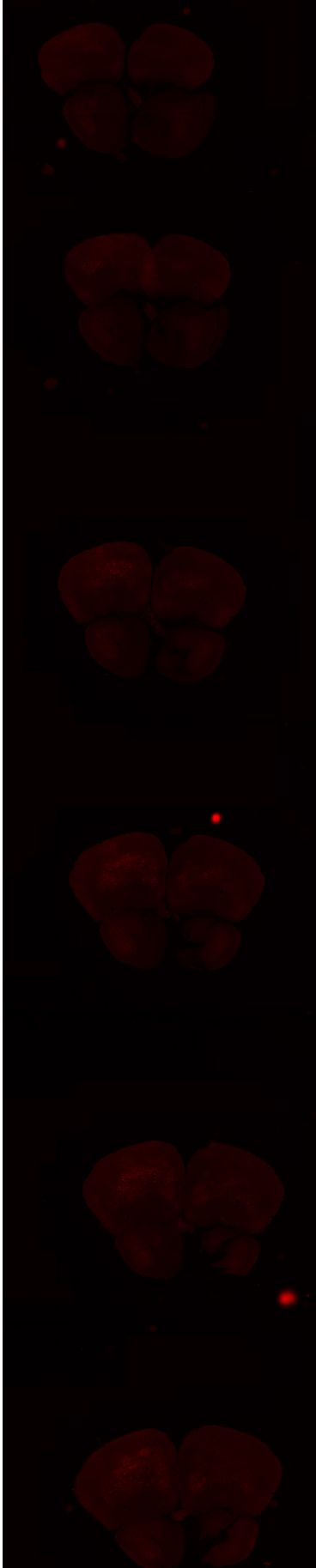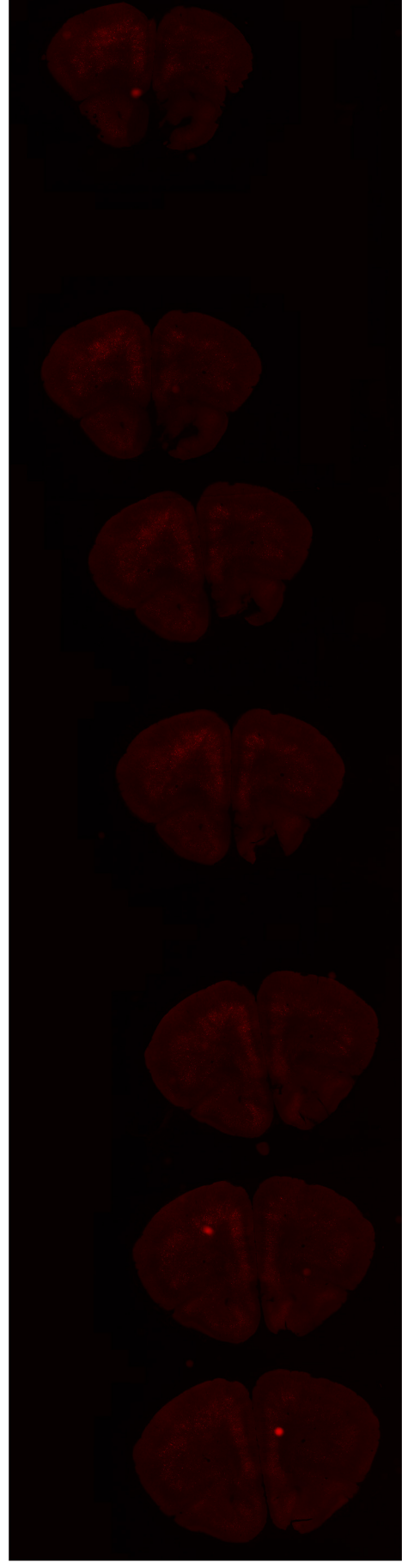

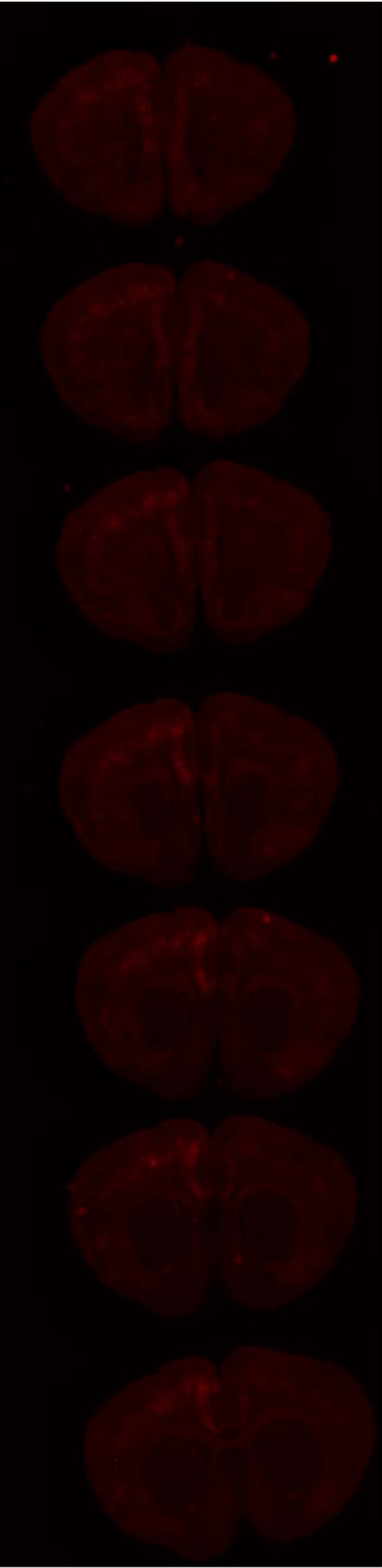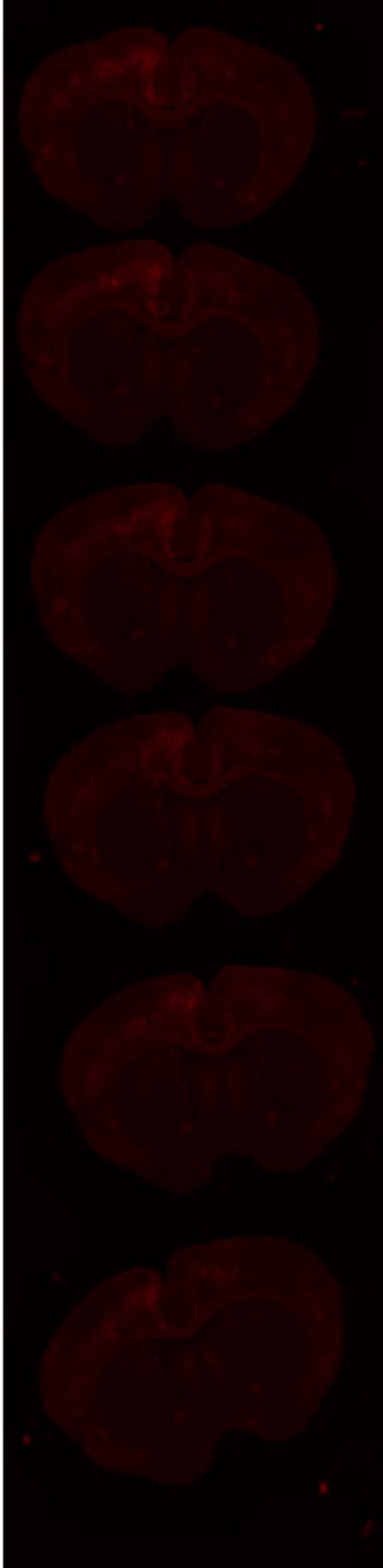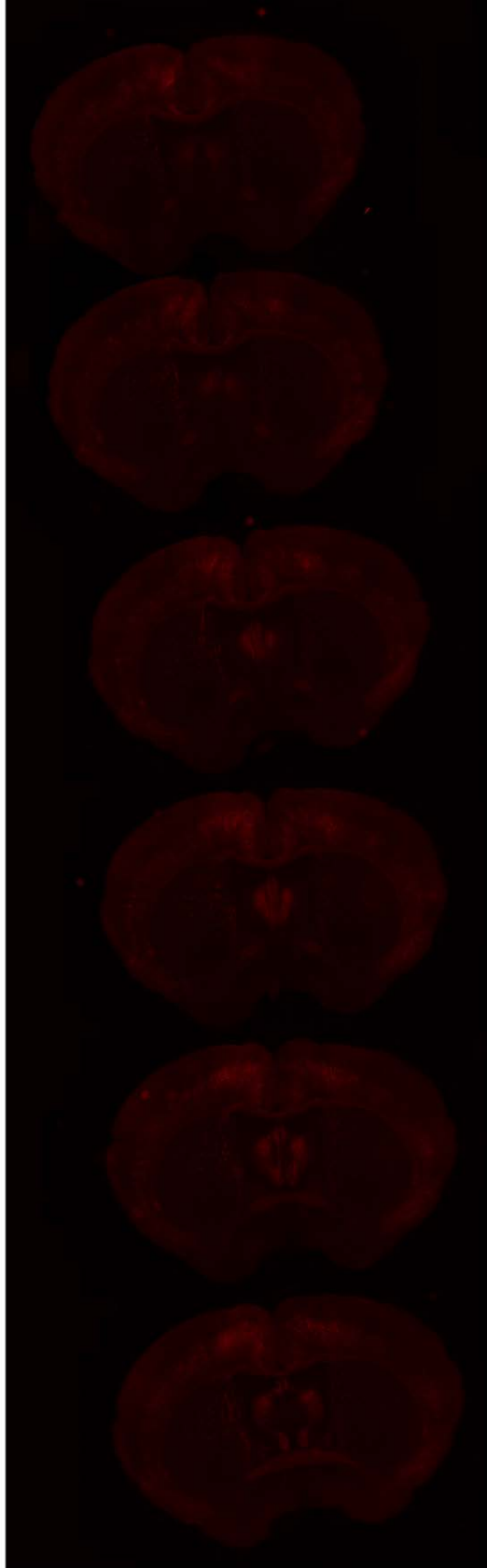



**GP 8.27**

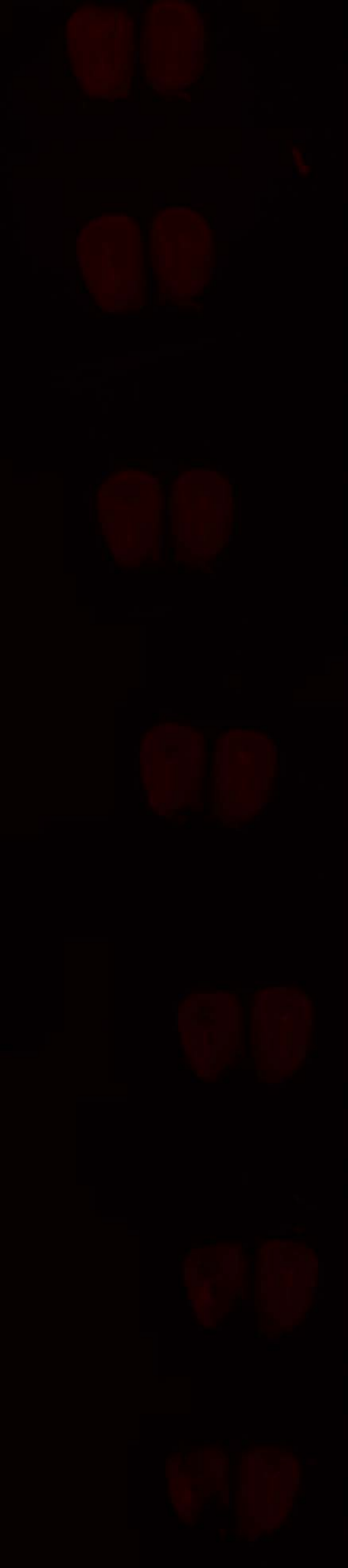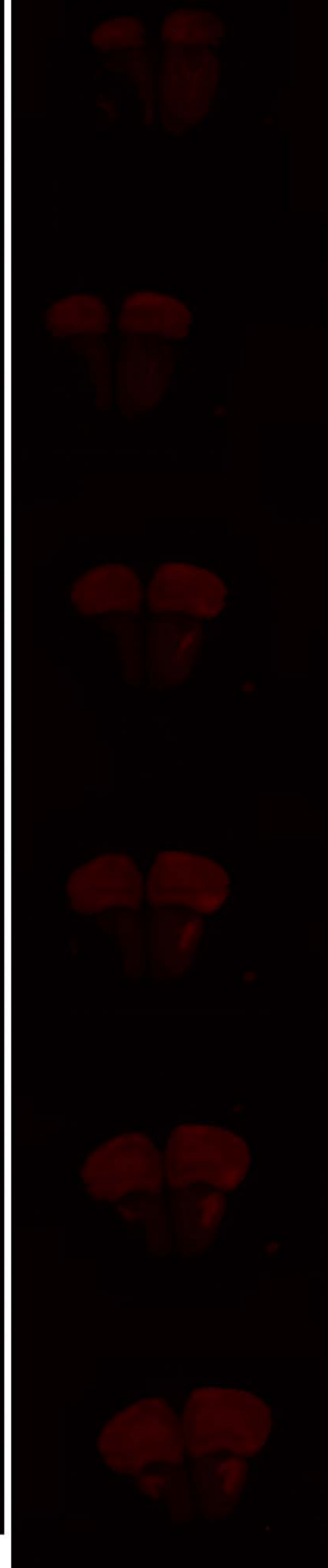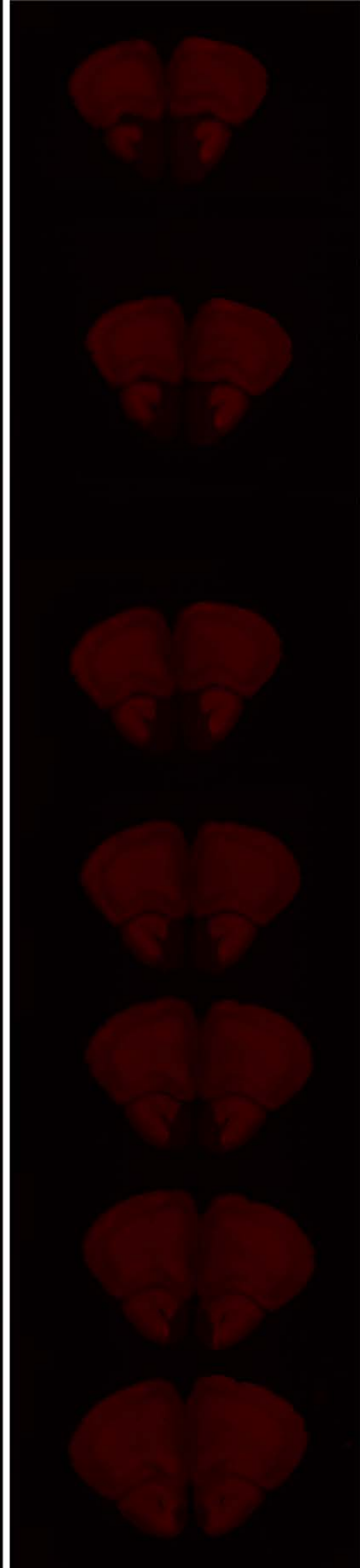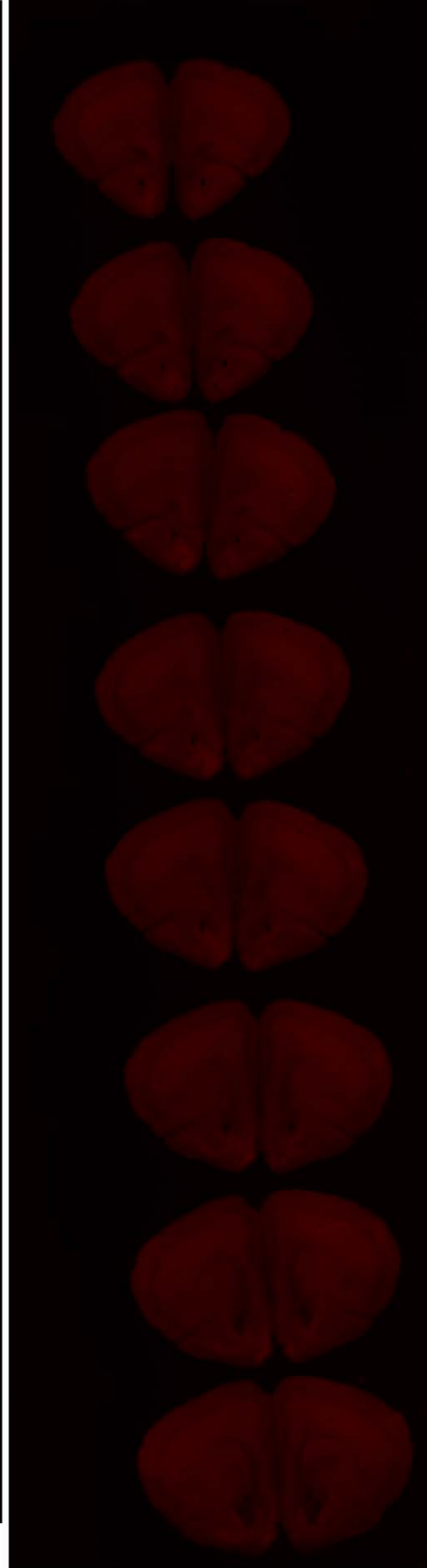

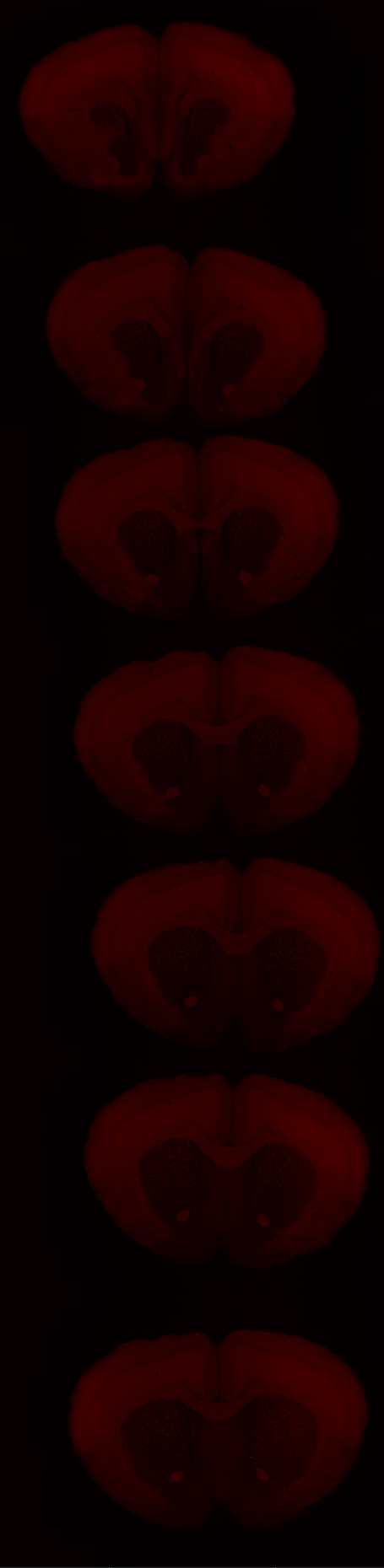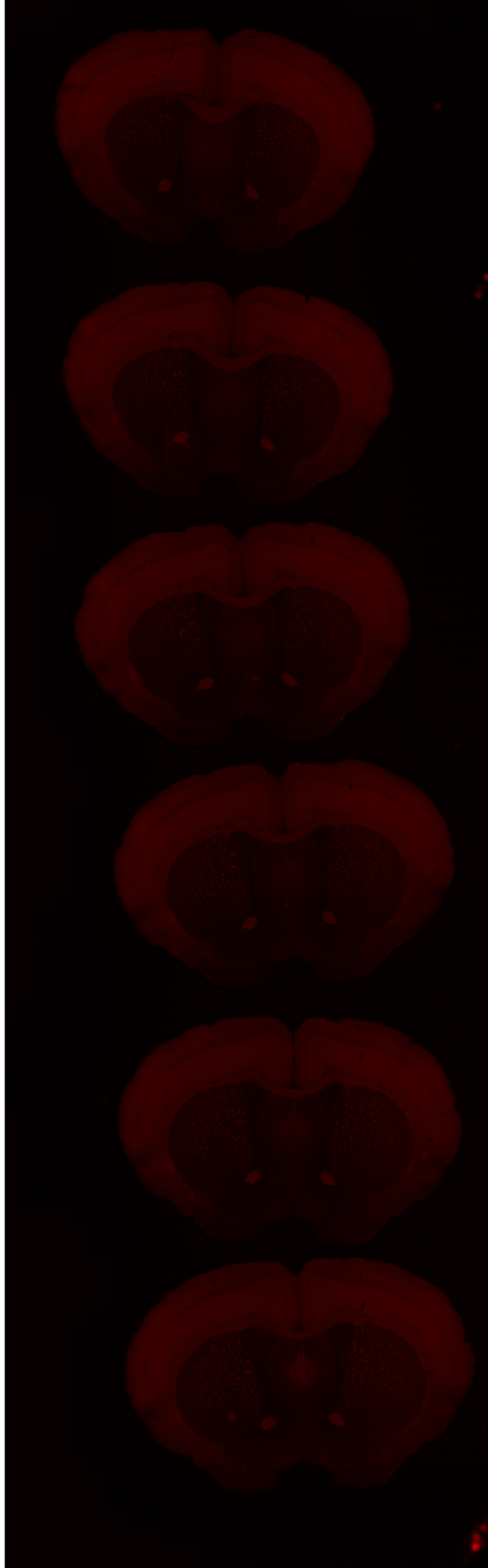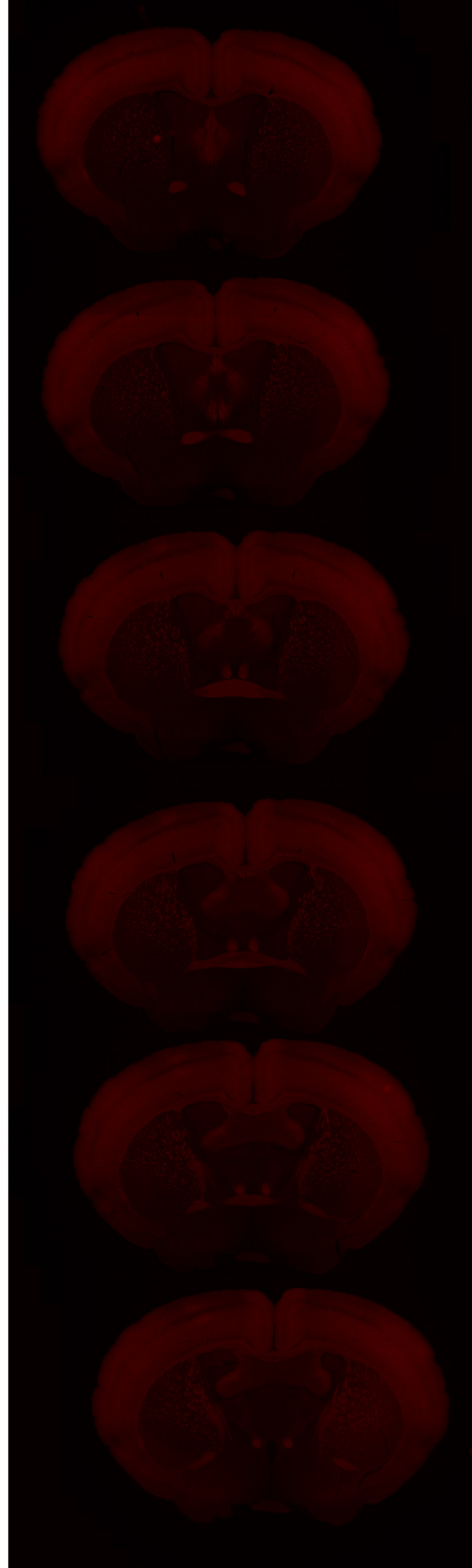

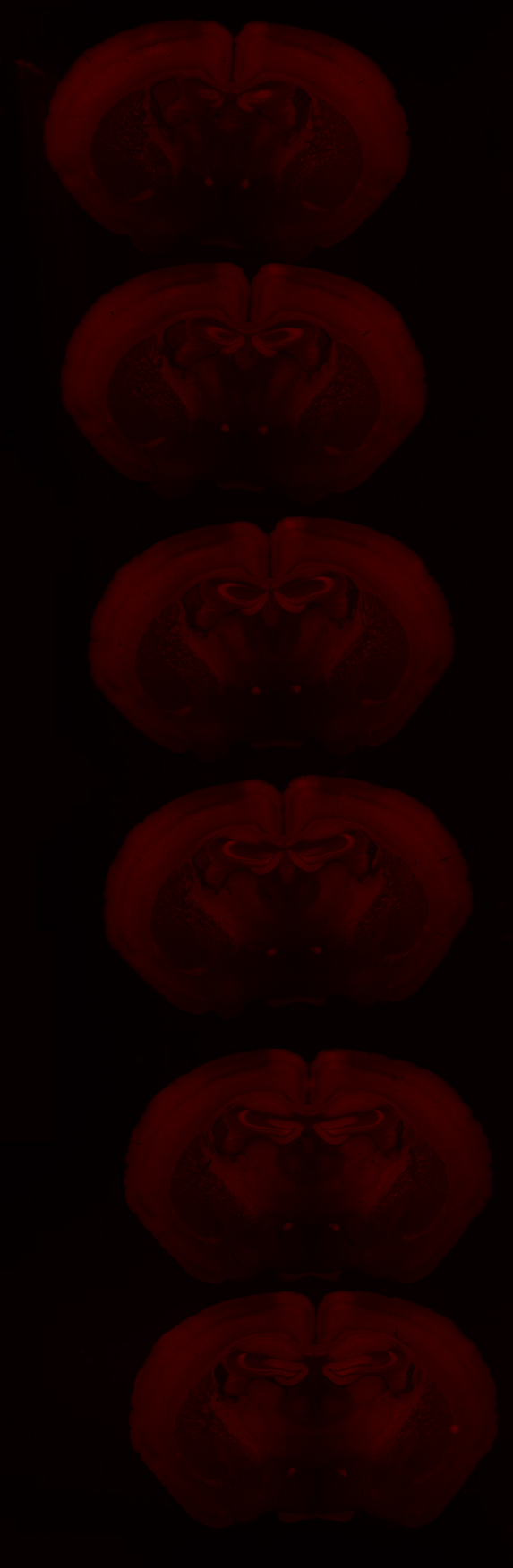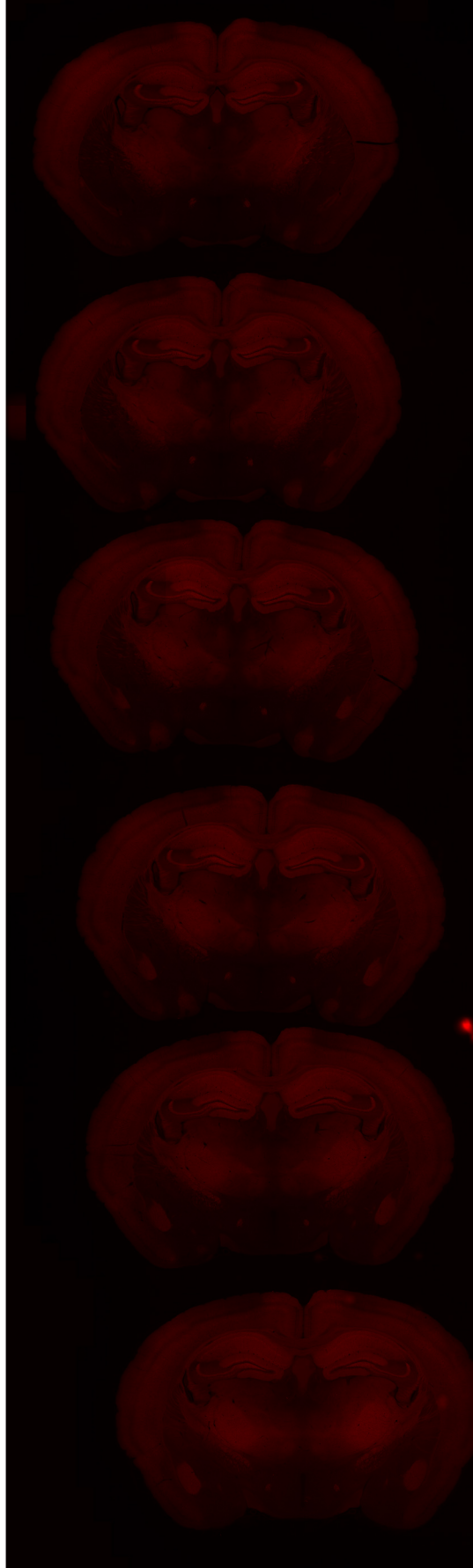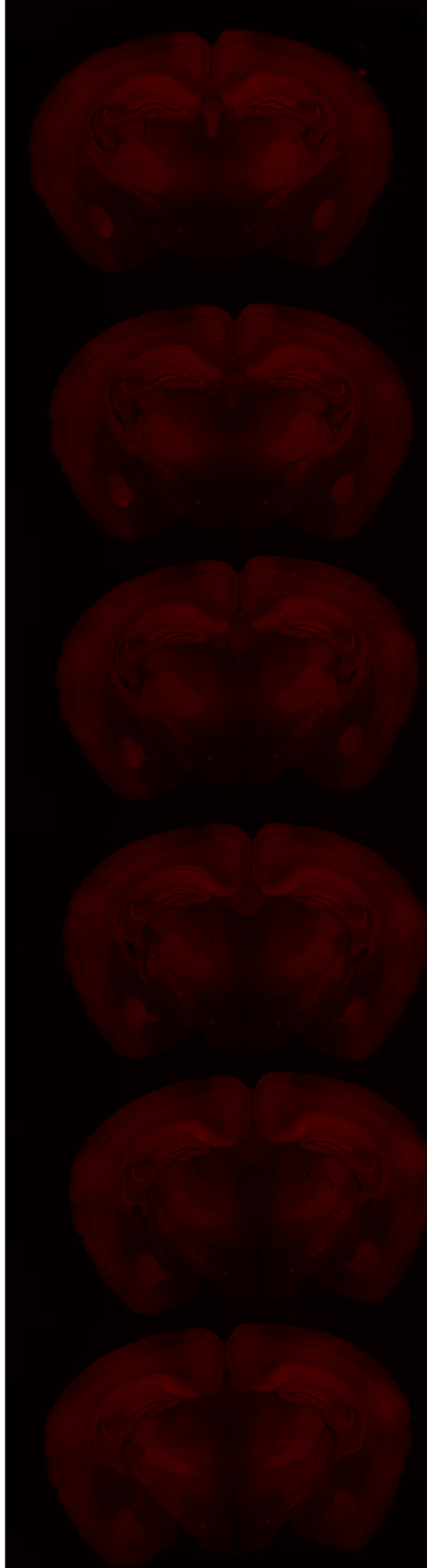

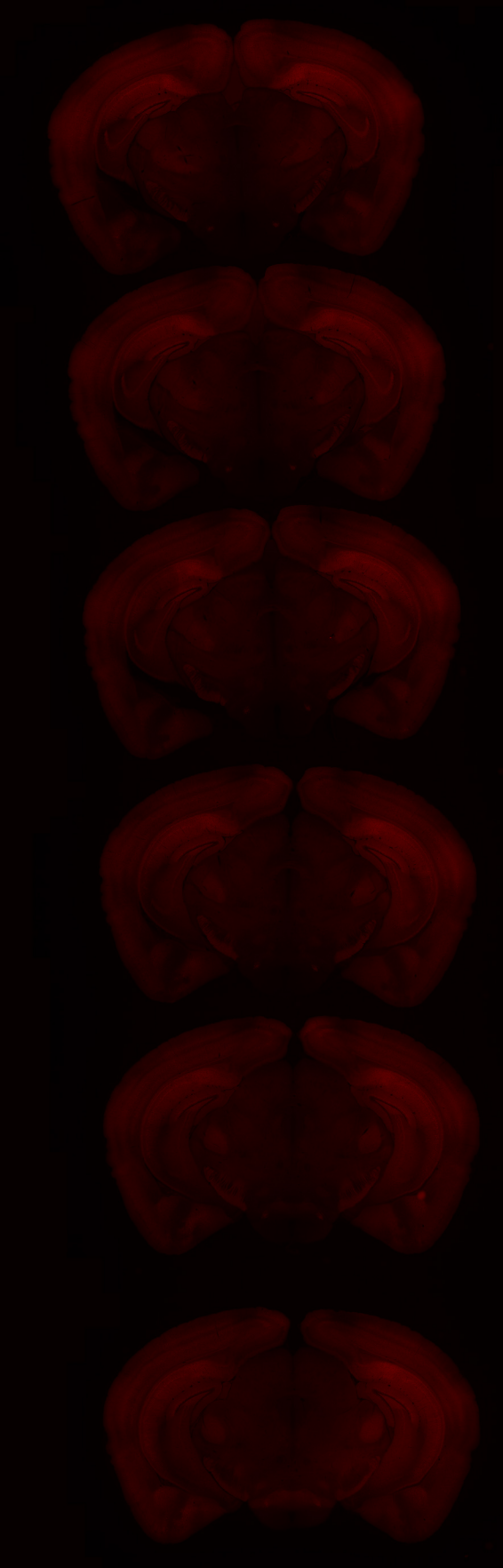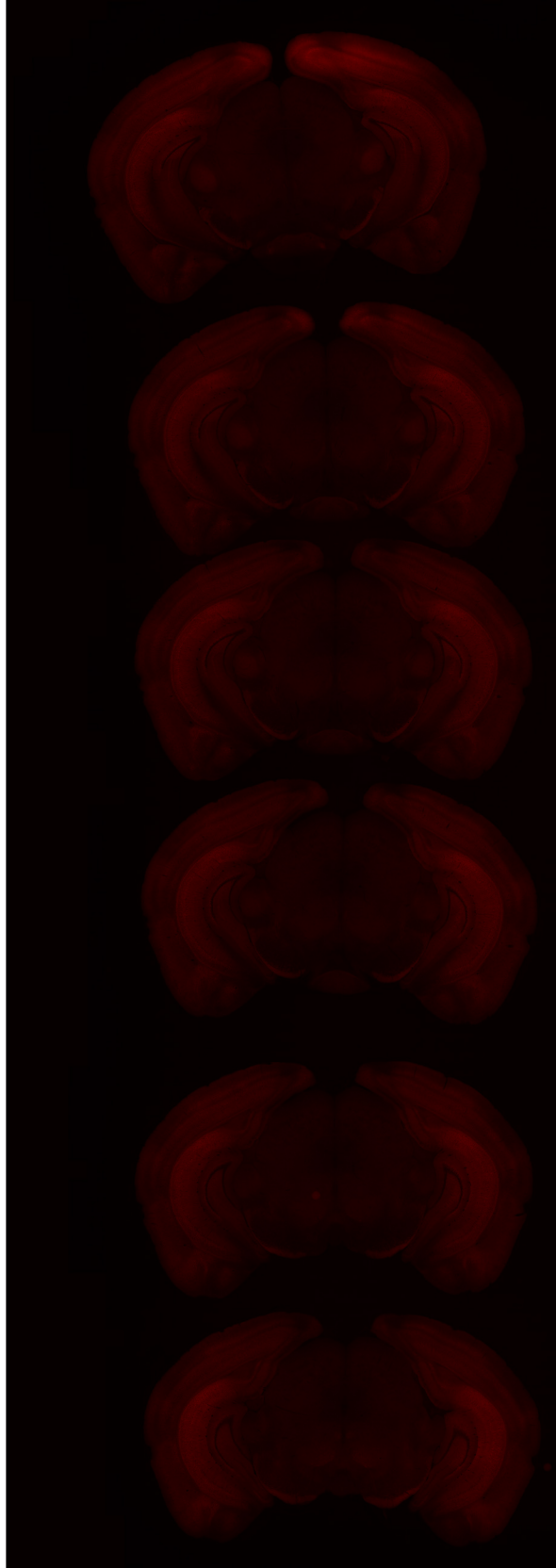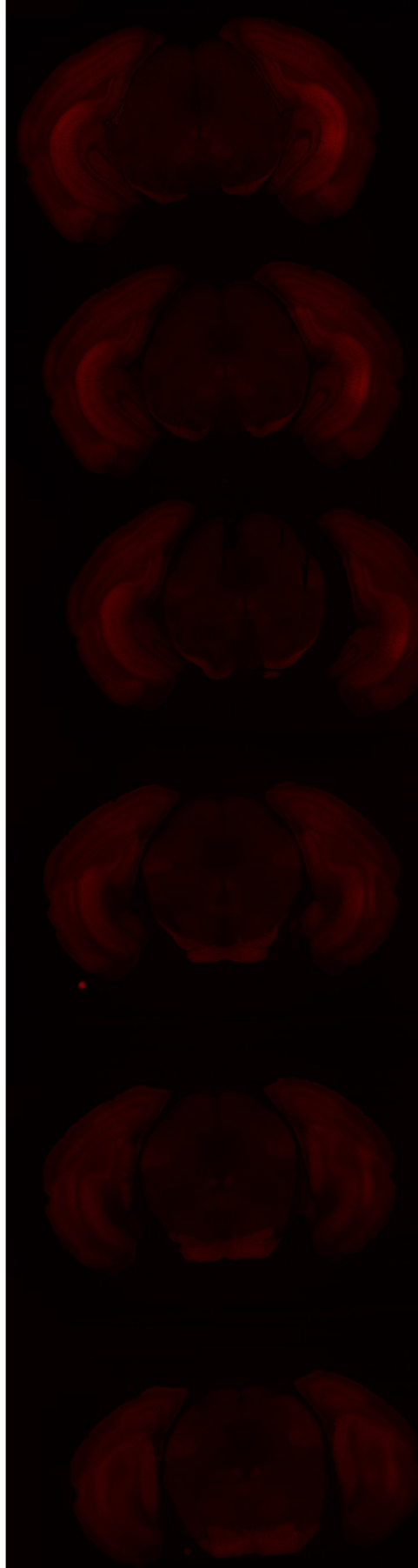

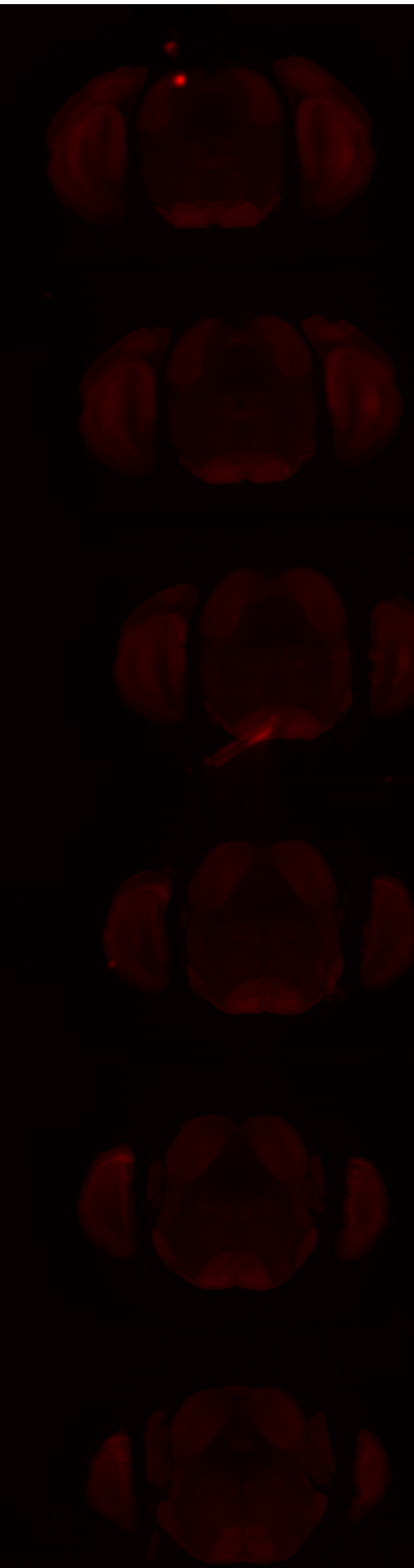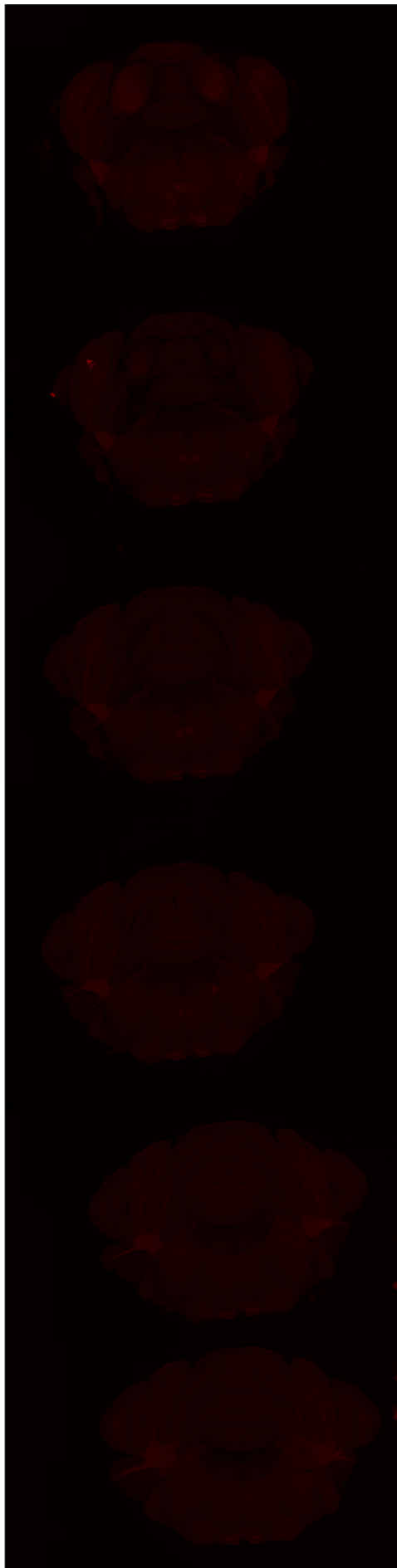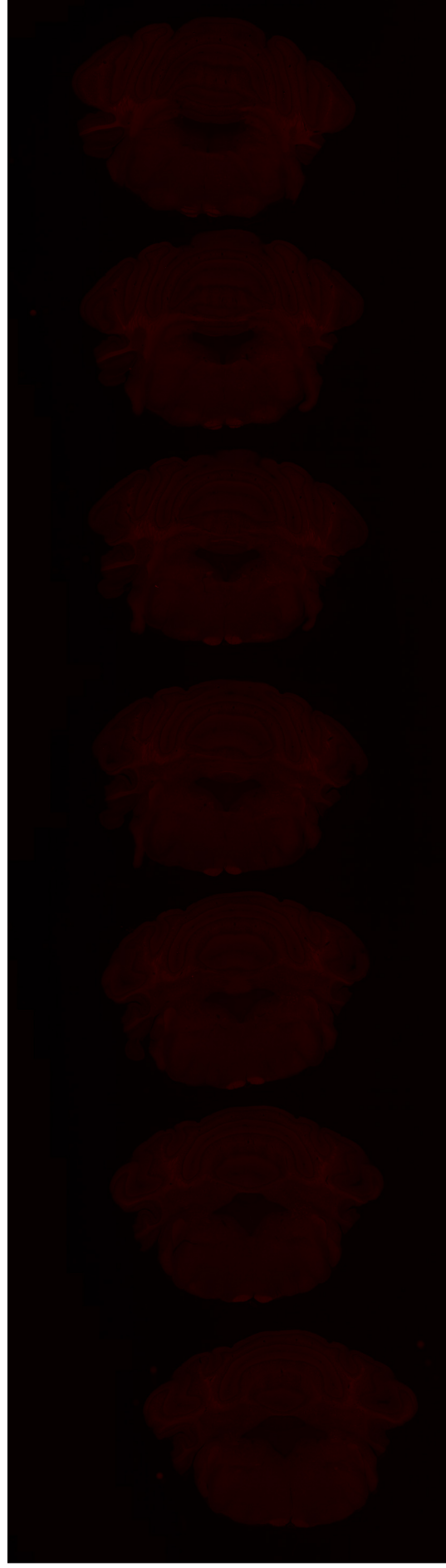

**GP 8.30**

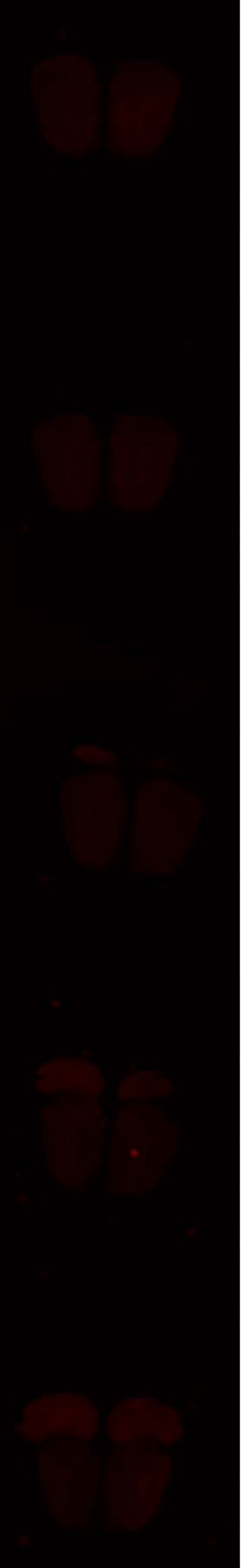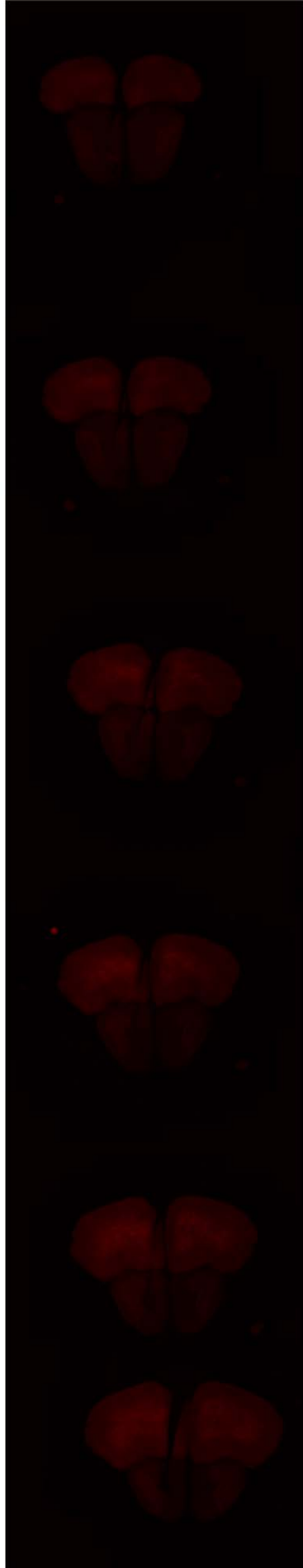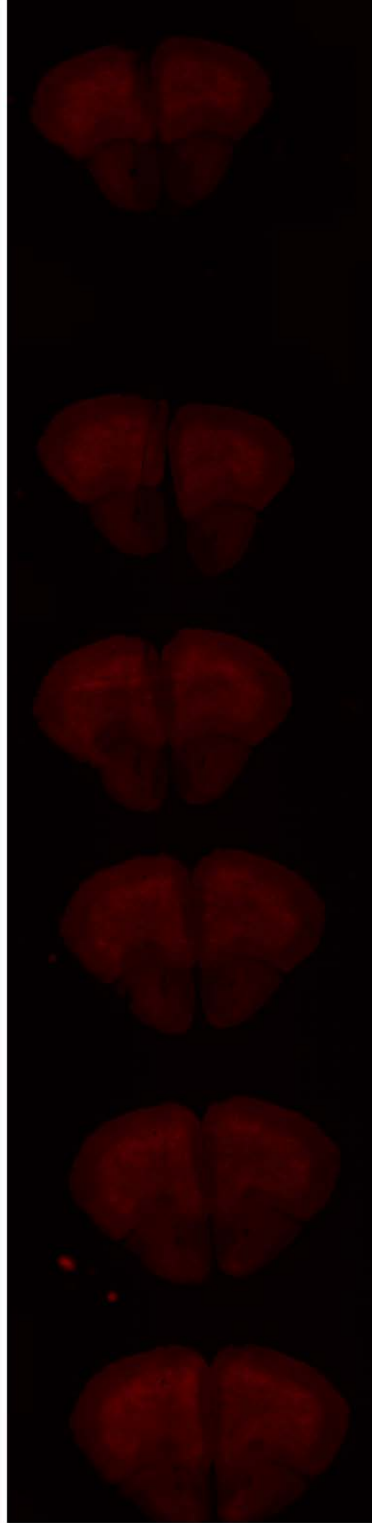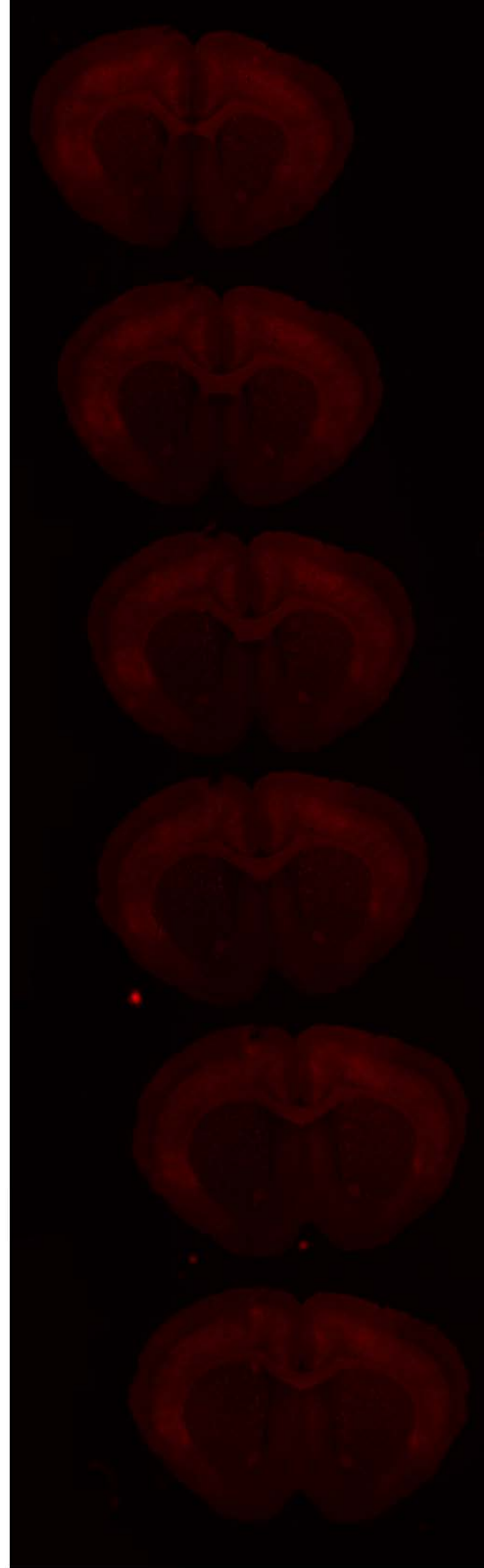

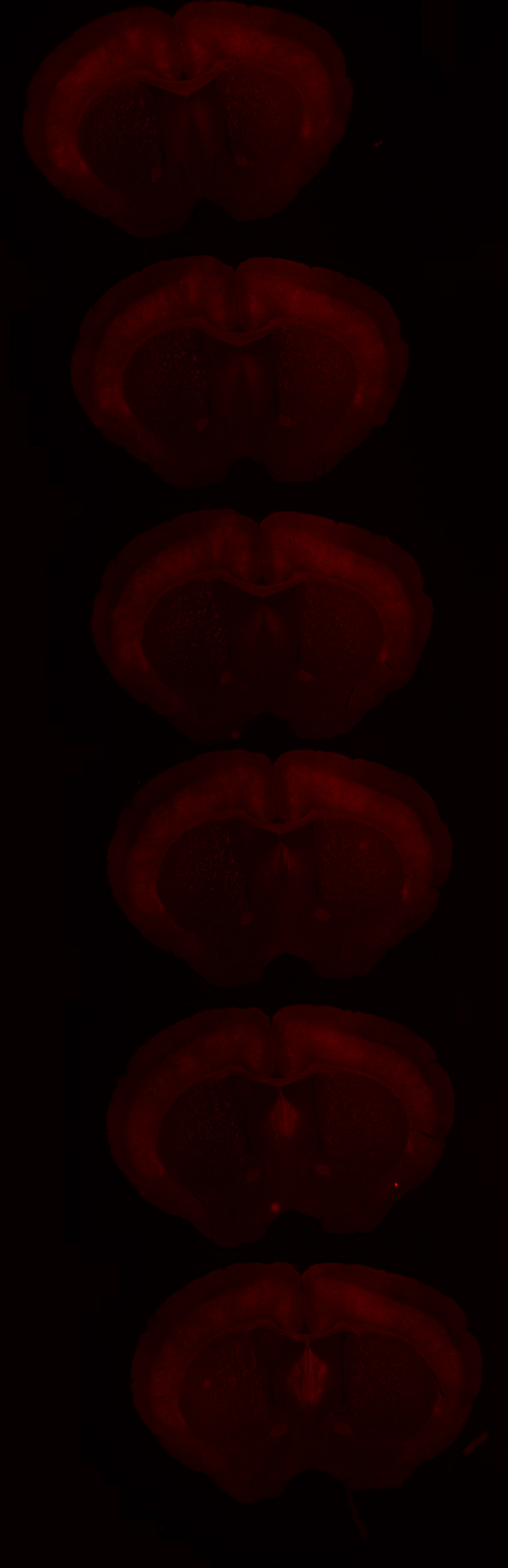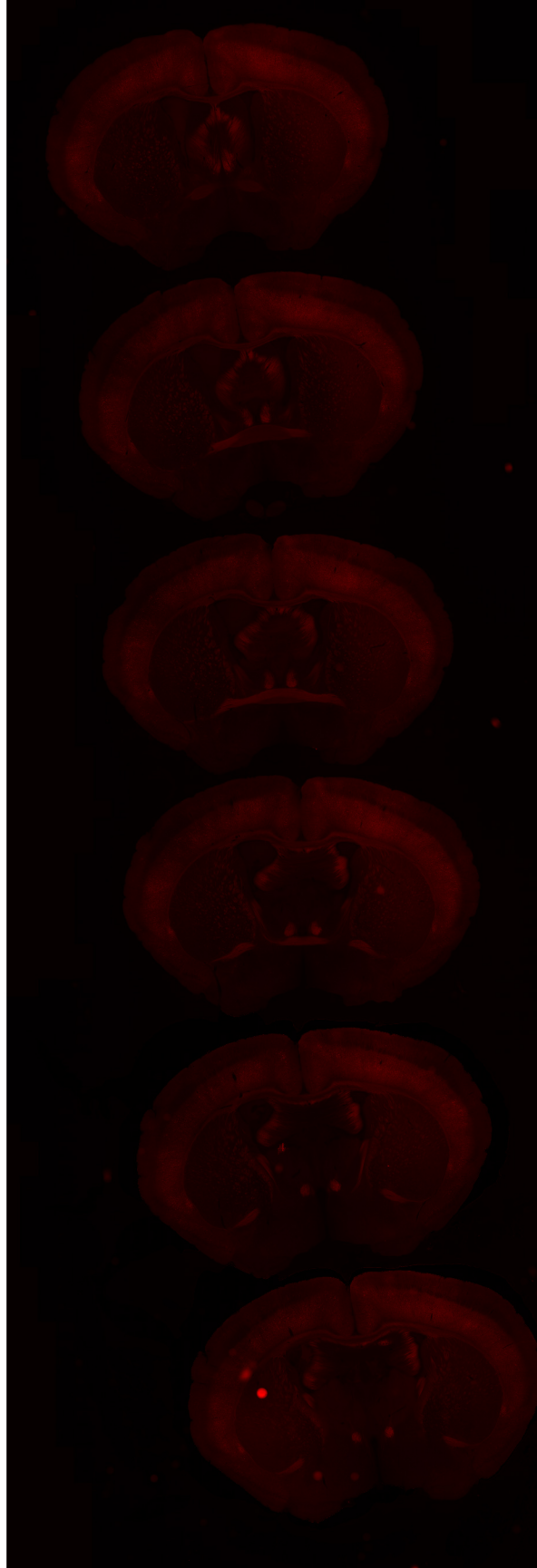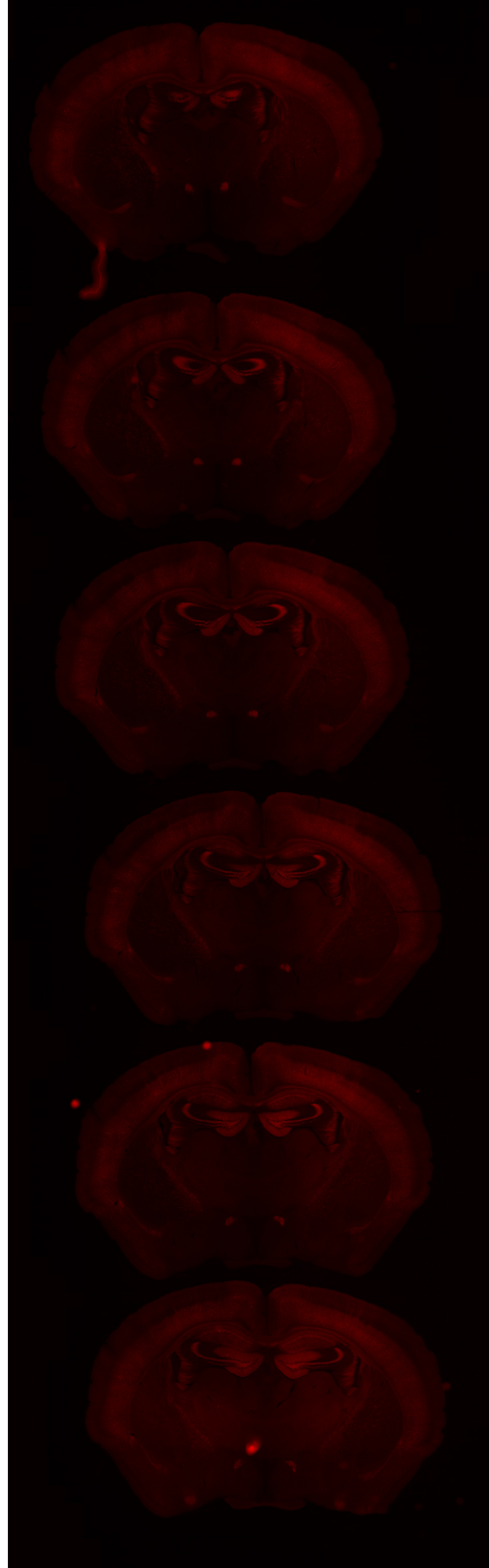

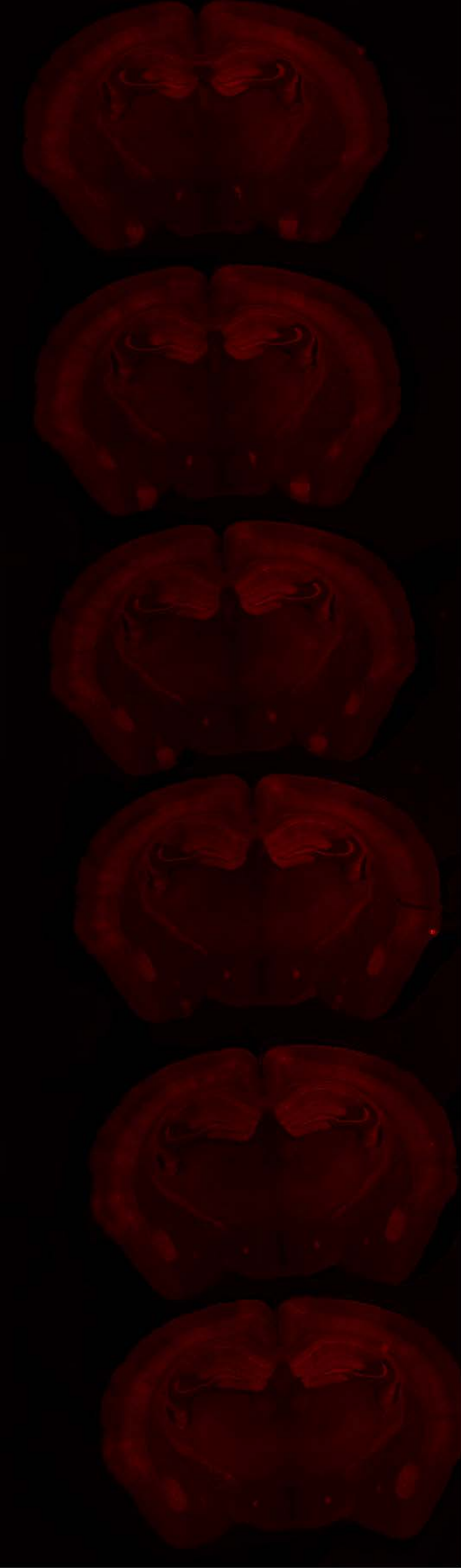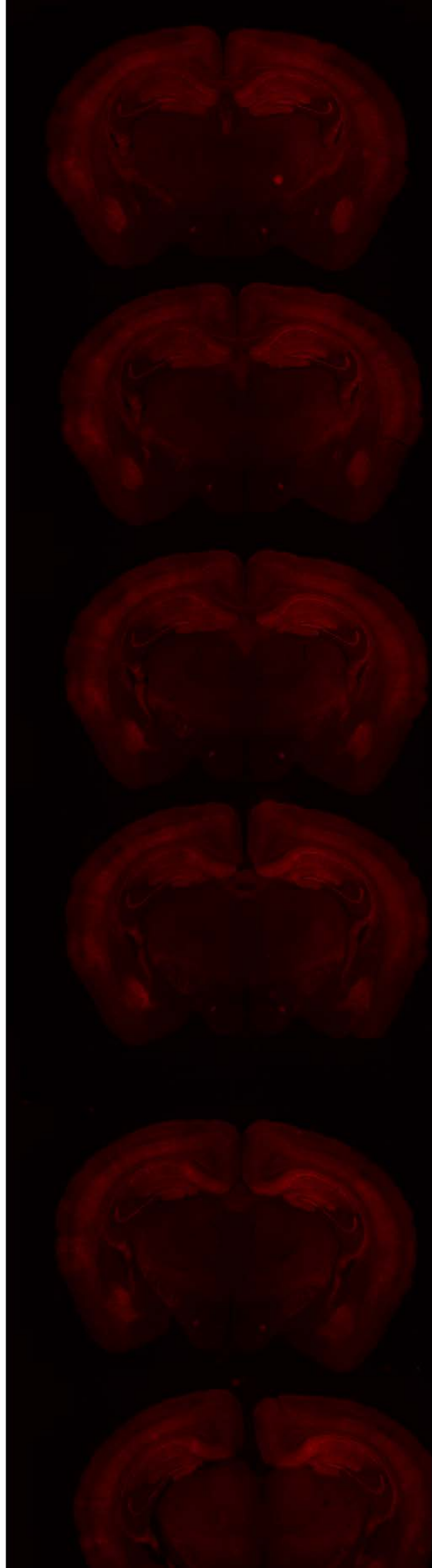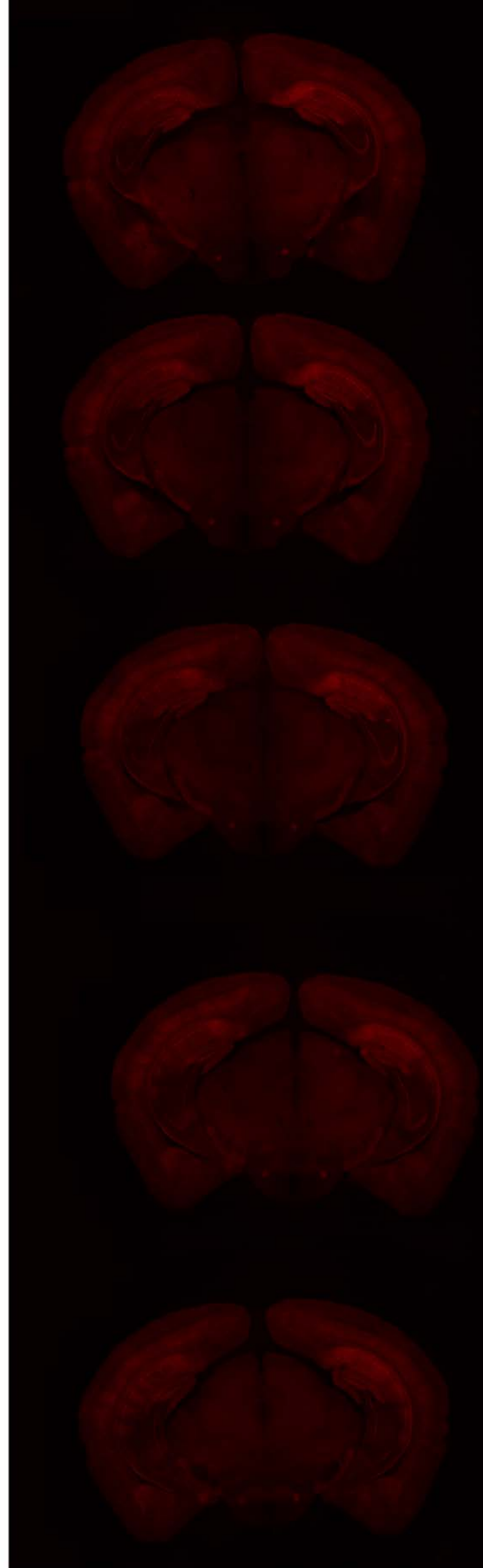

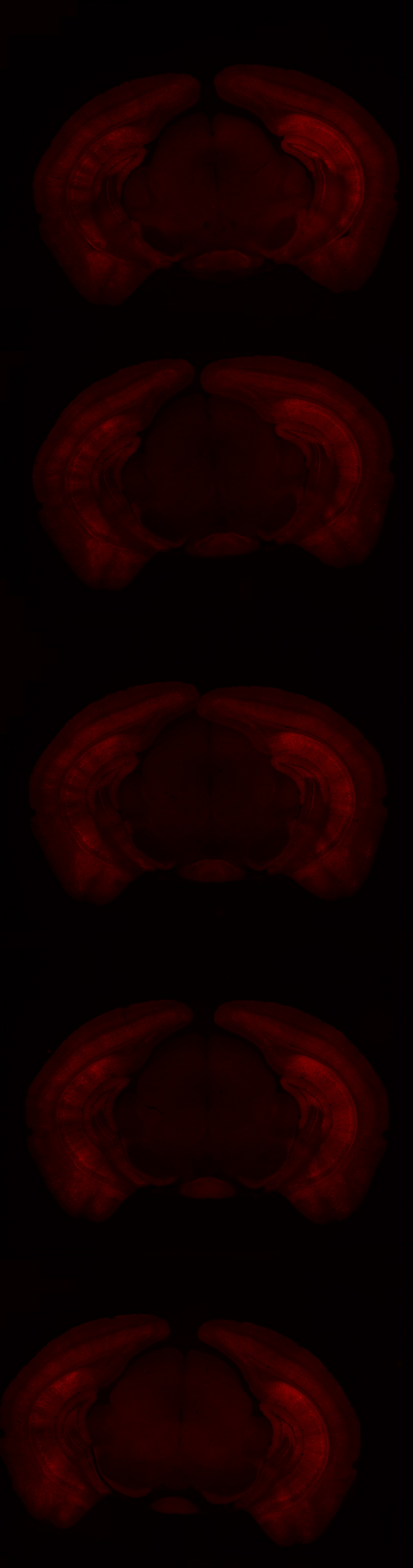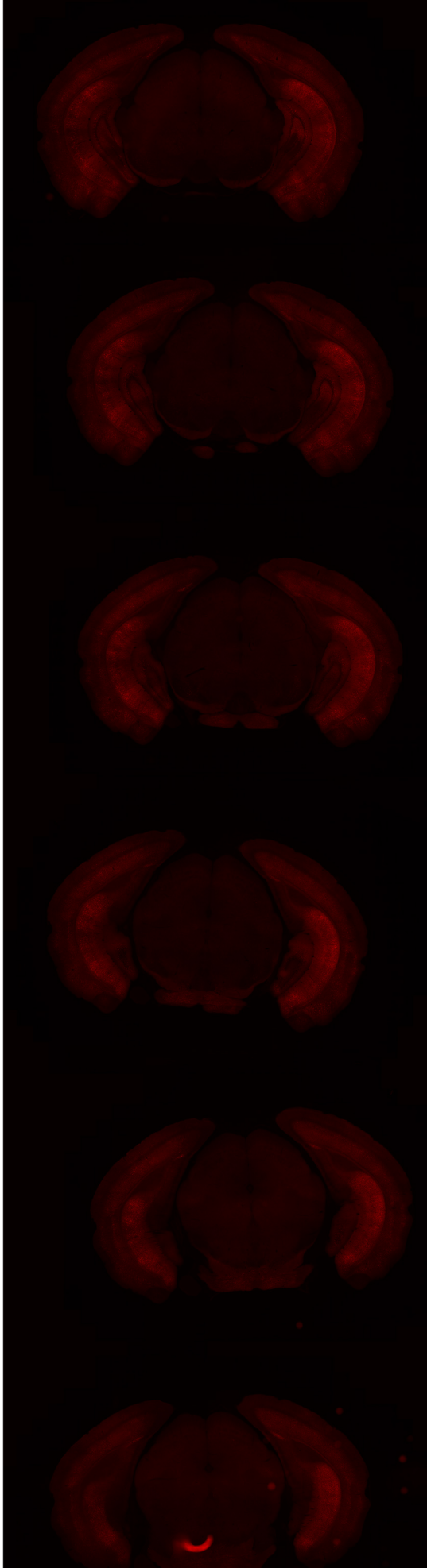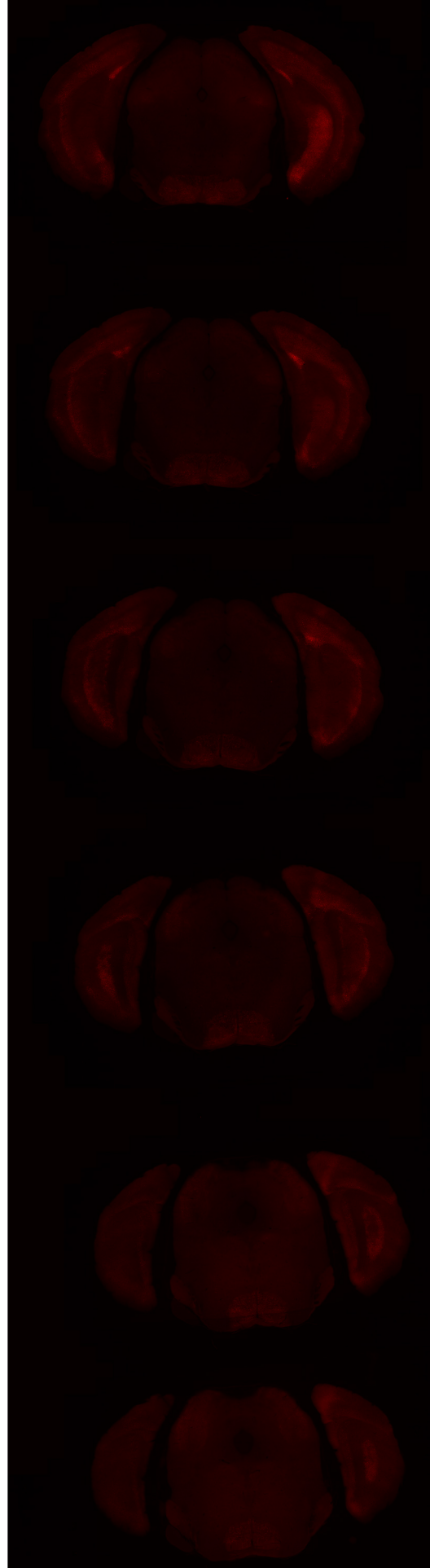

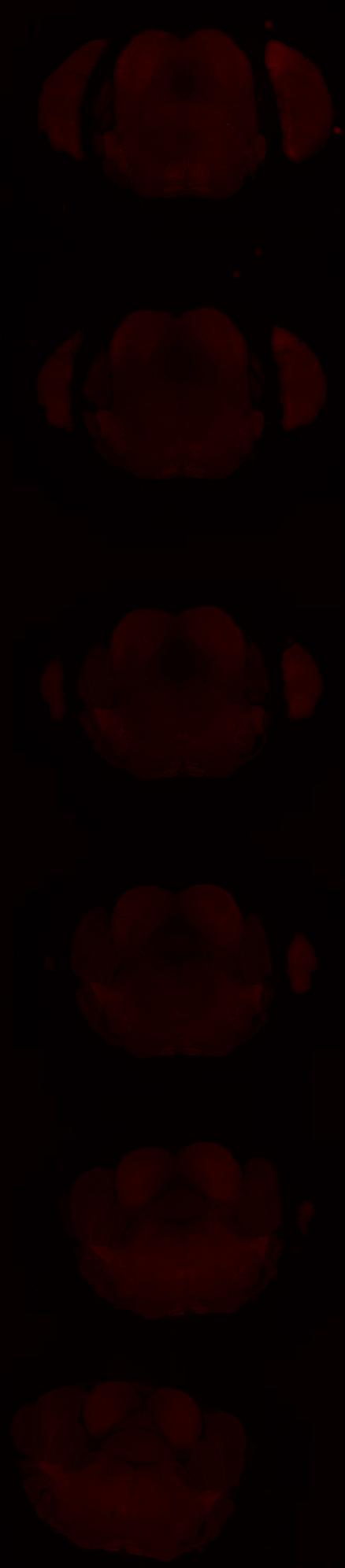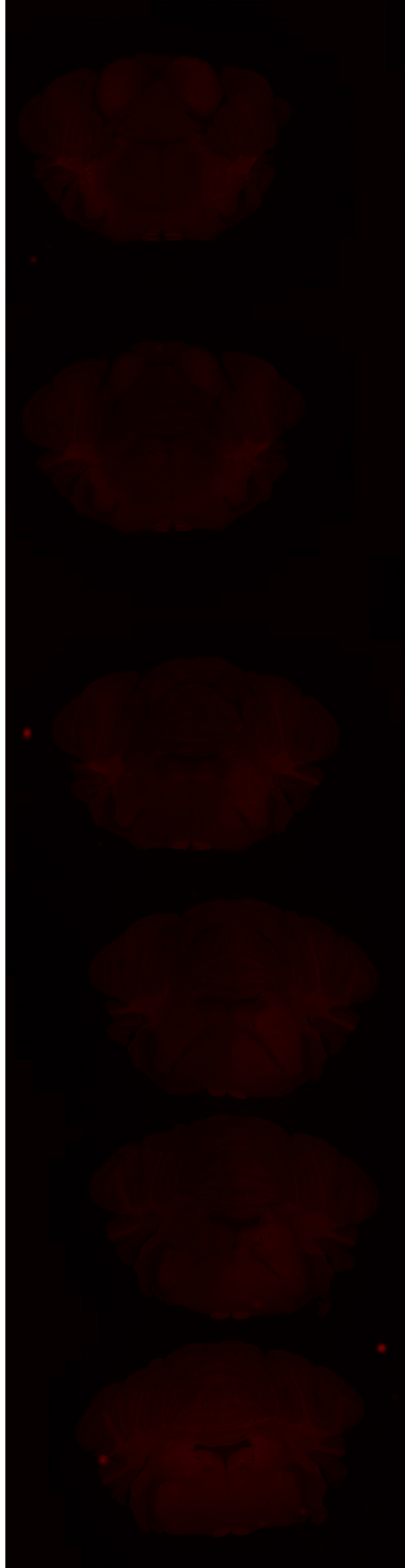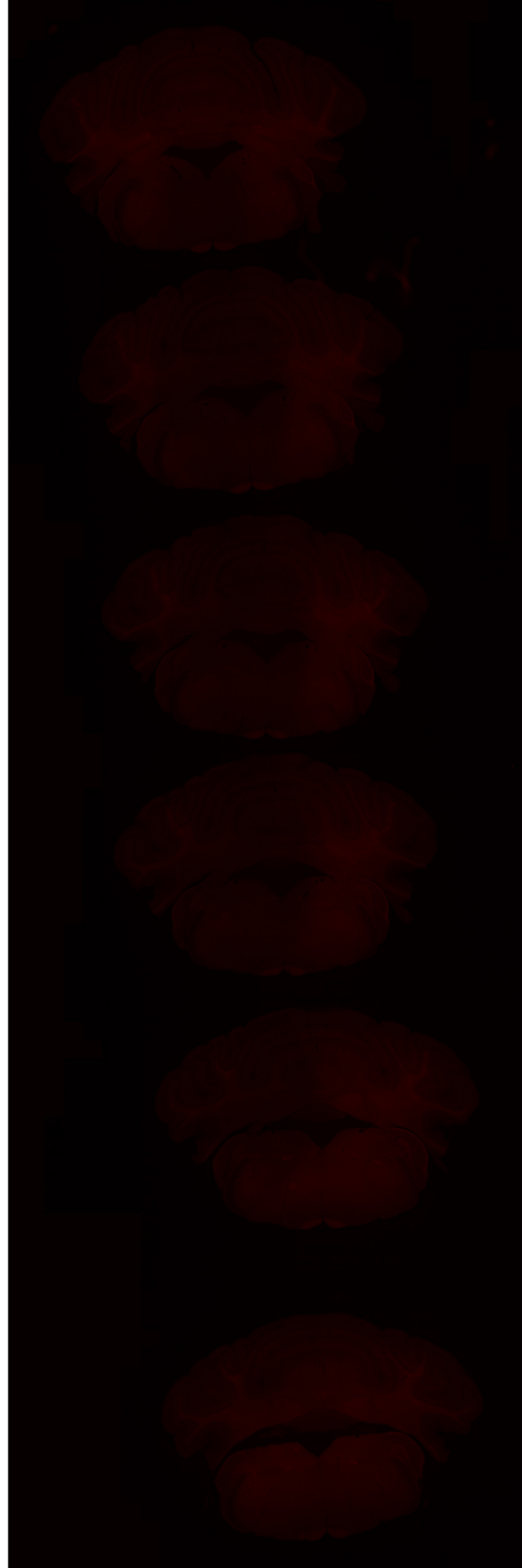

**GP 8.31**

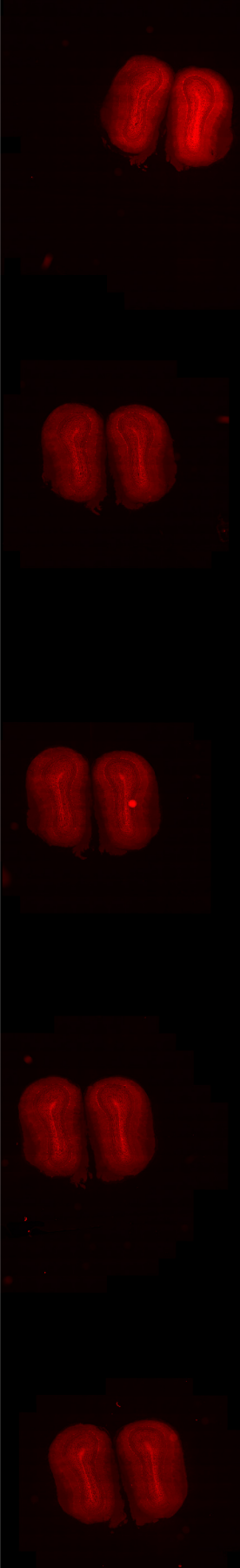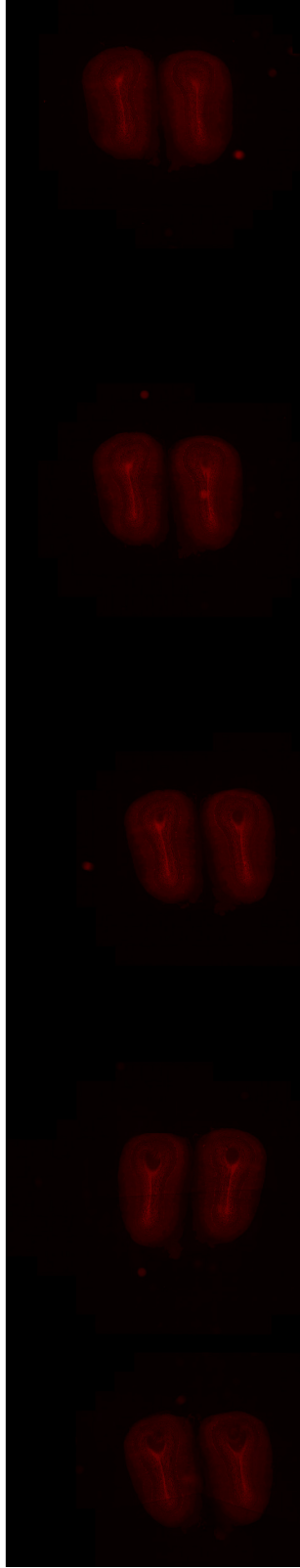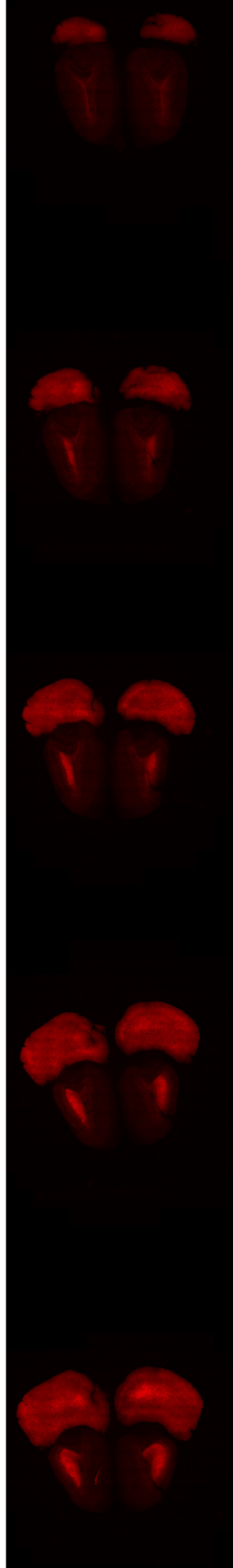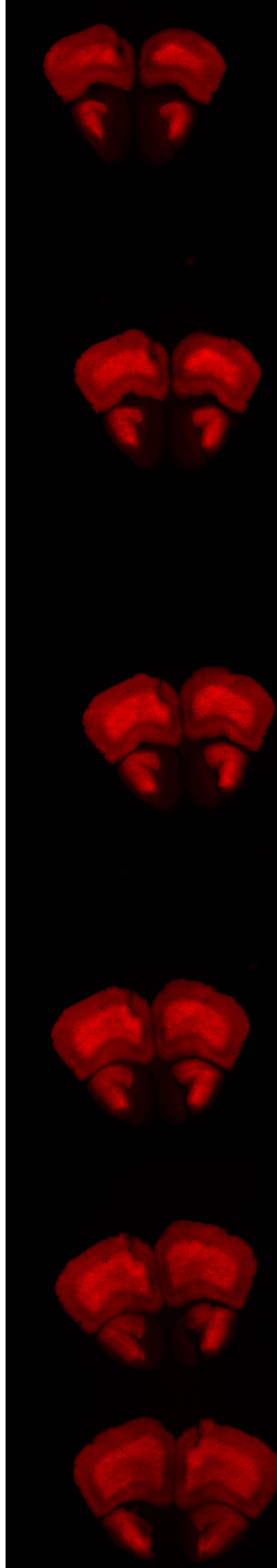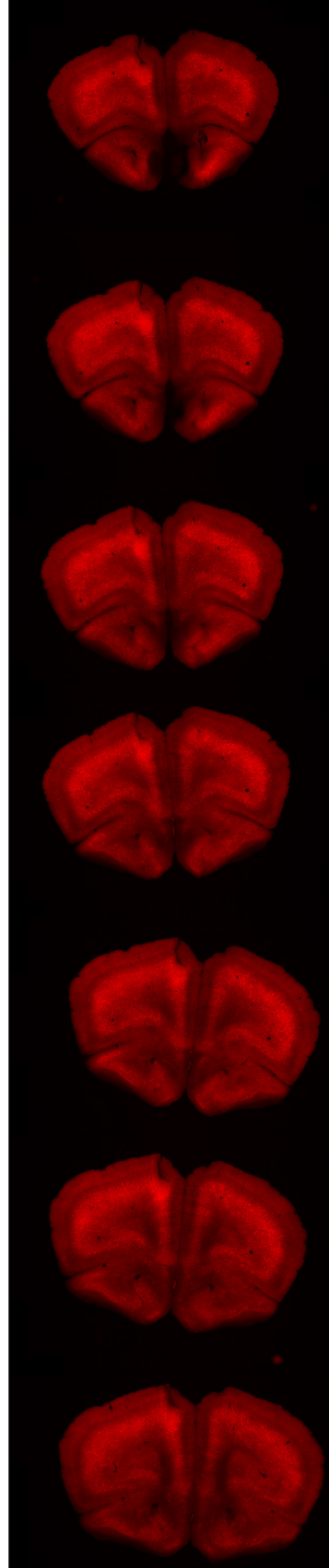

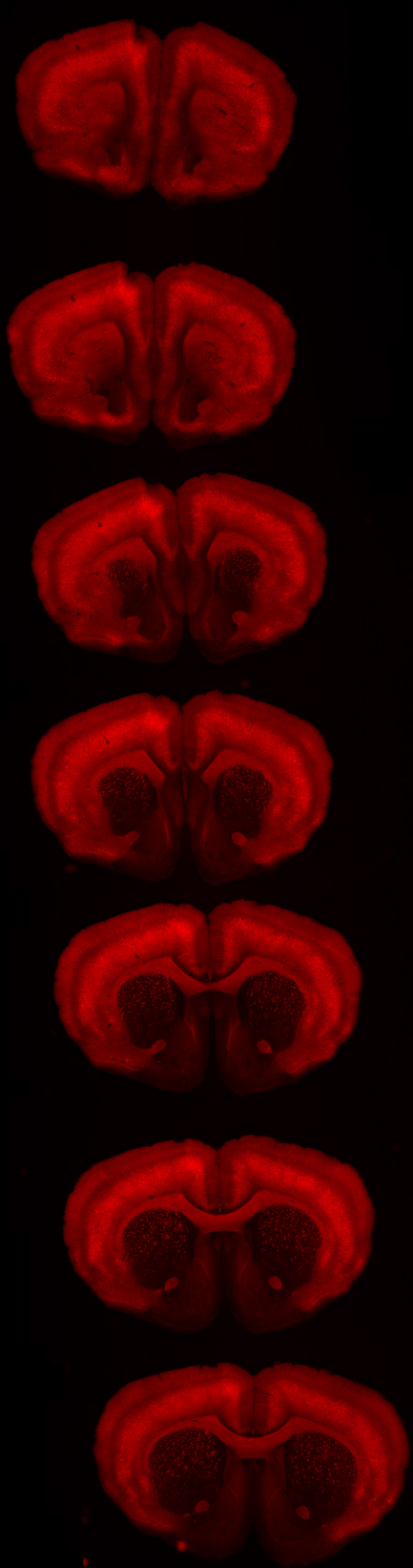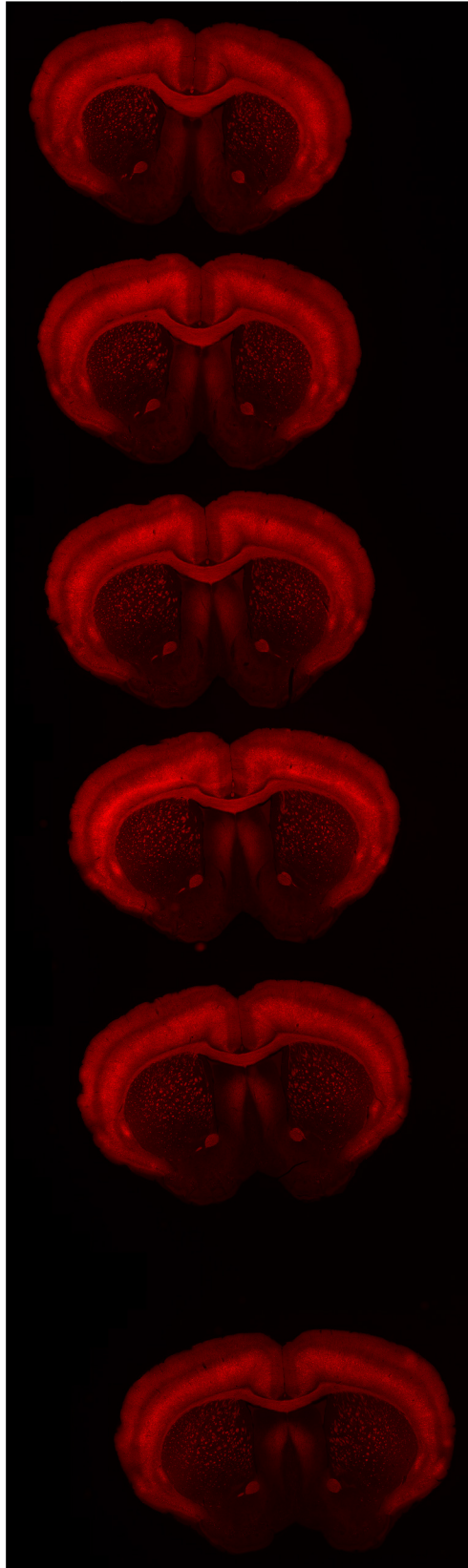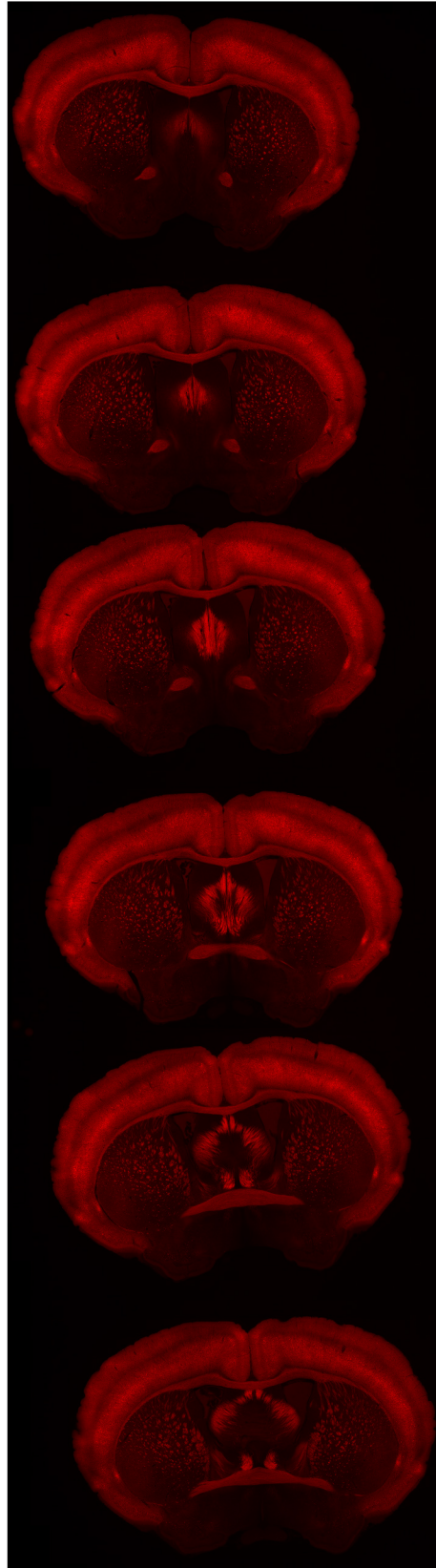

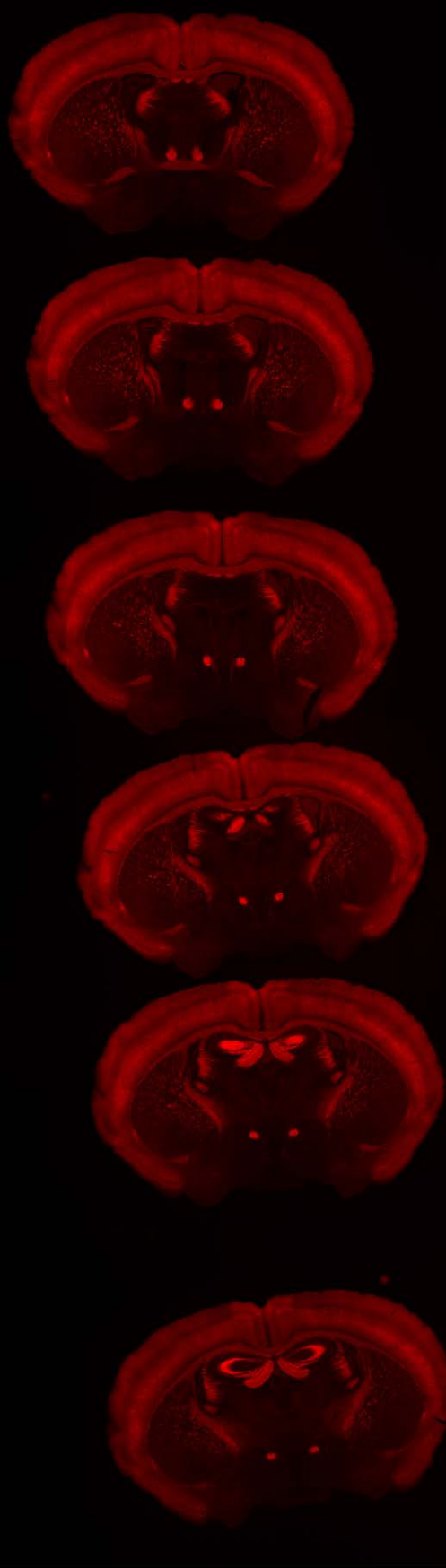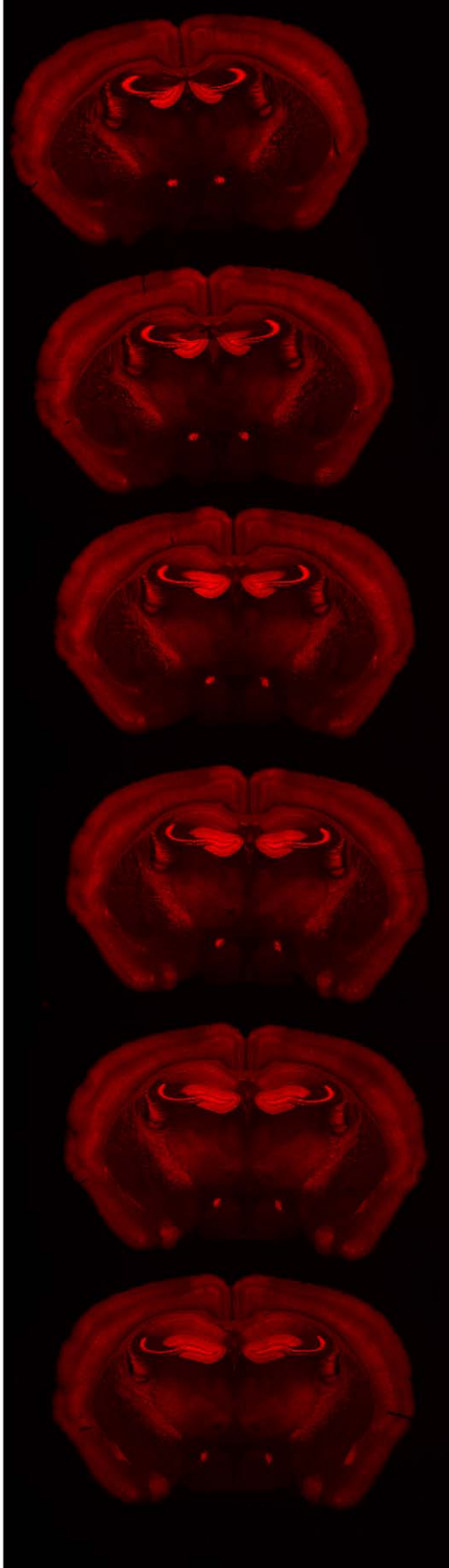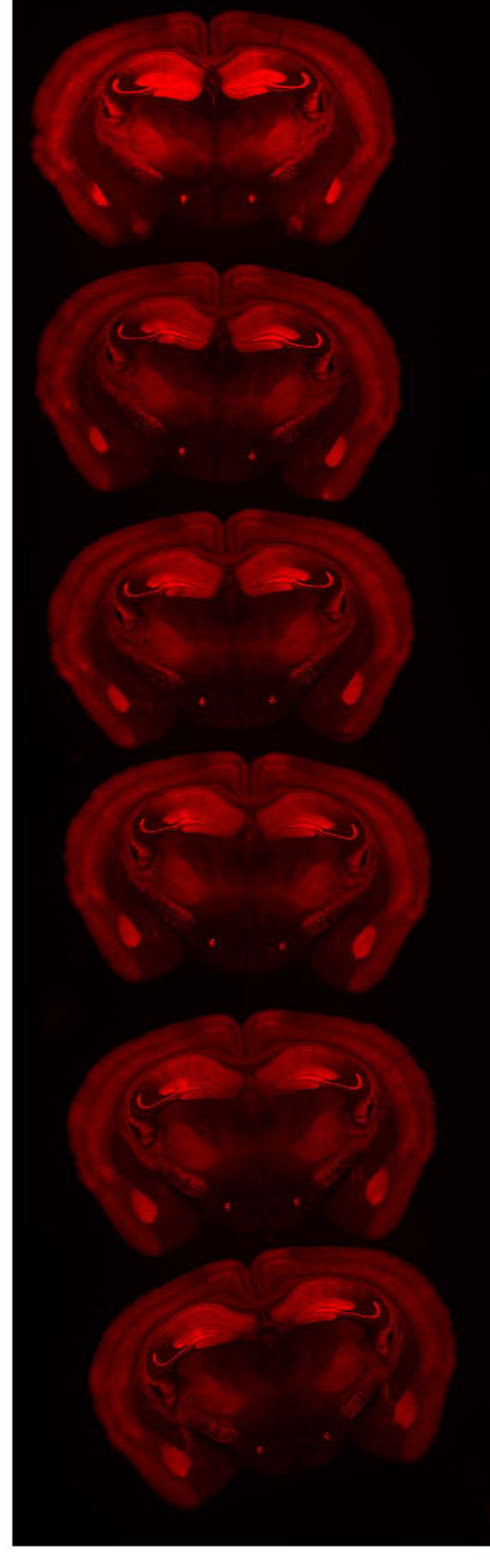

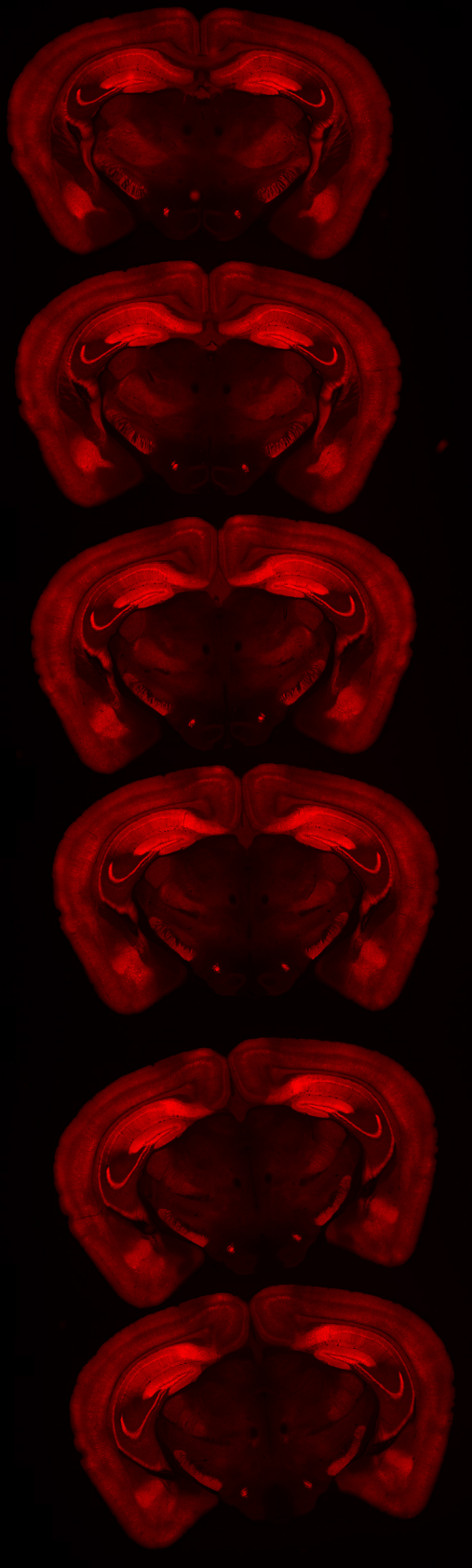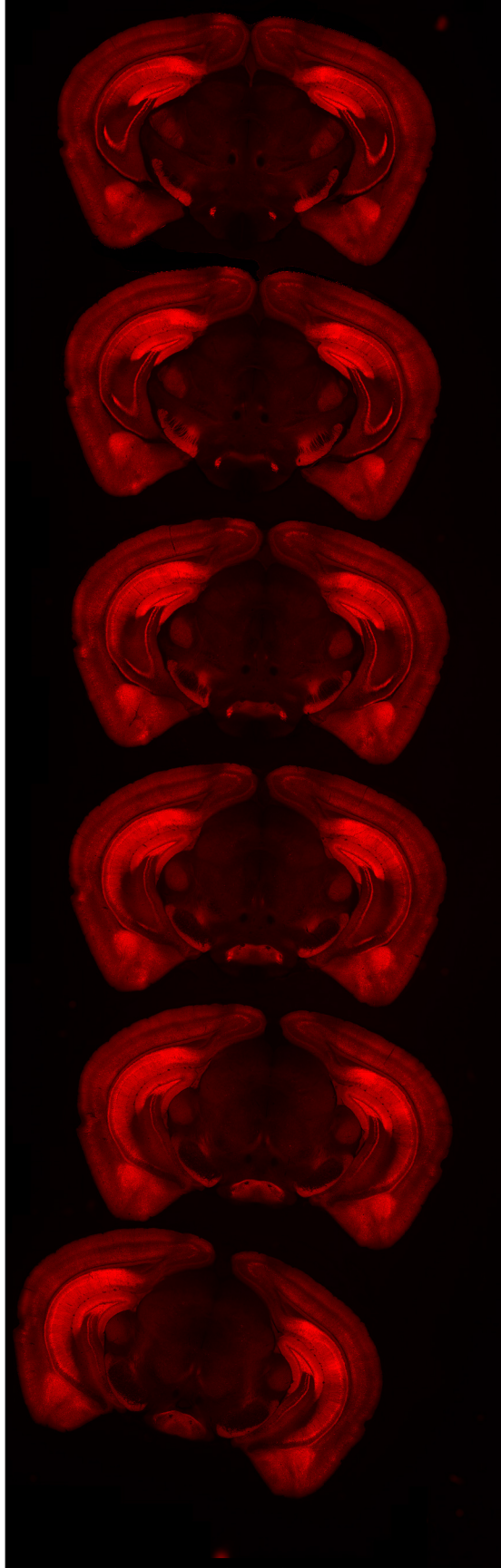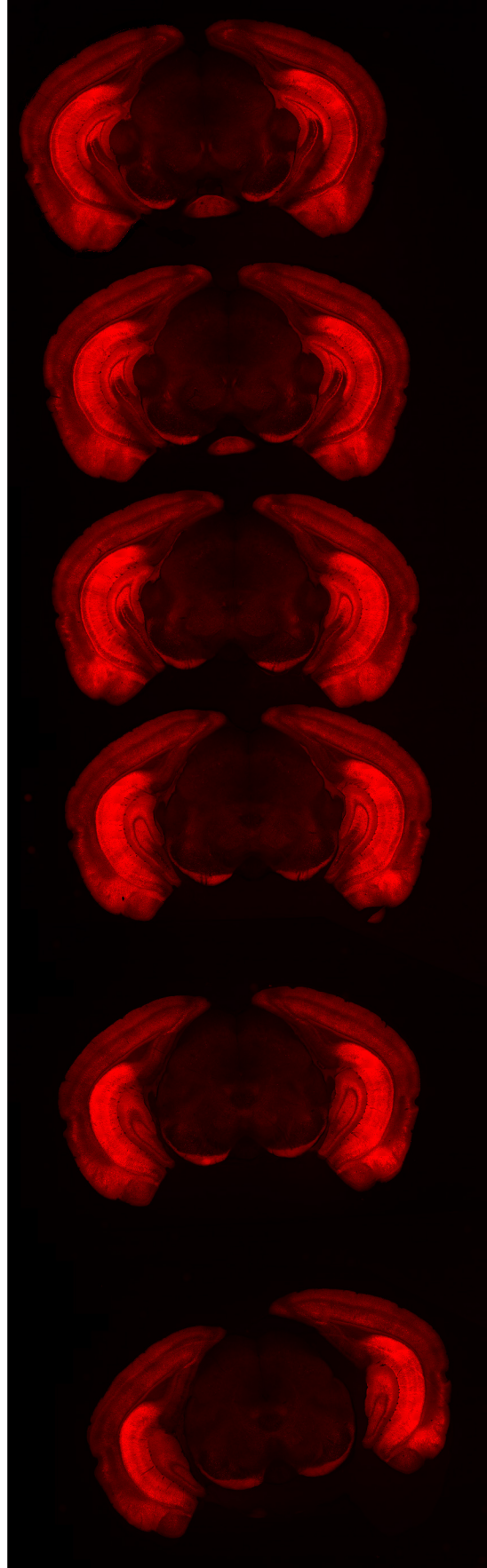

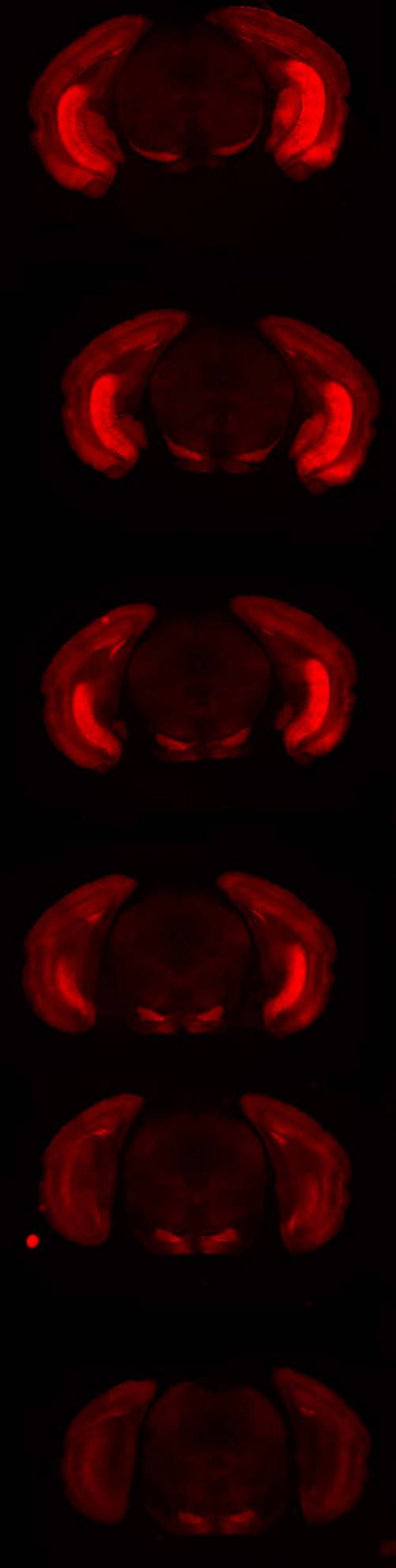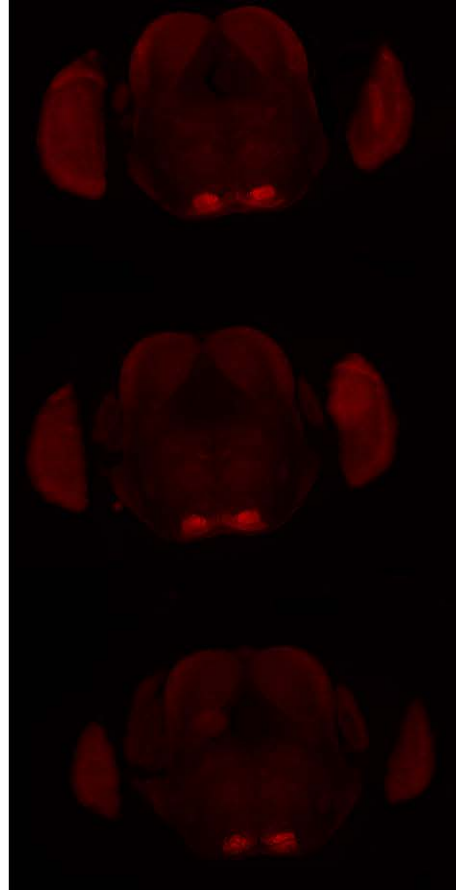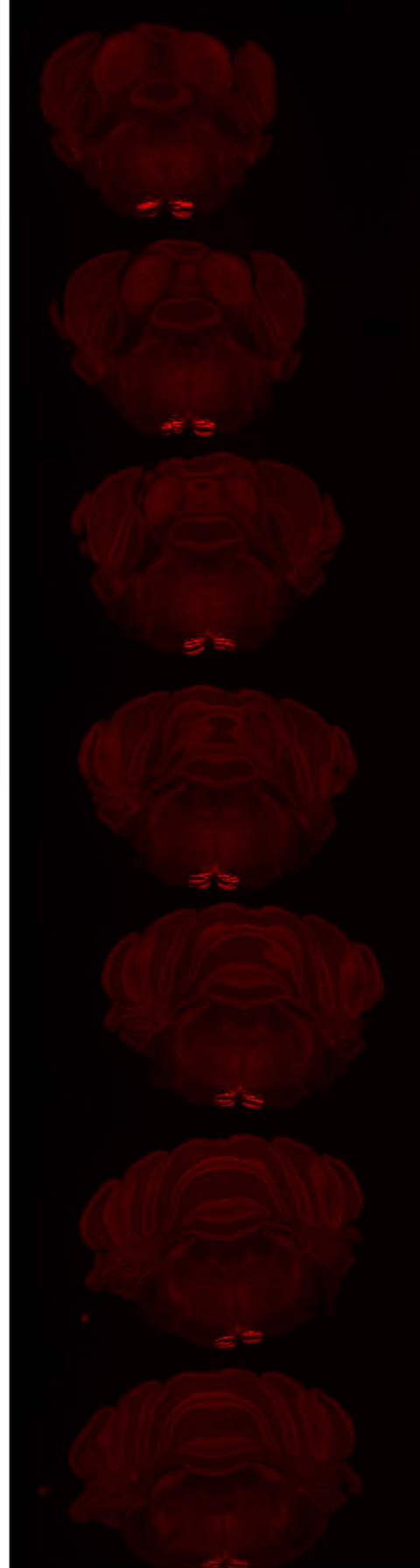

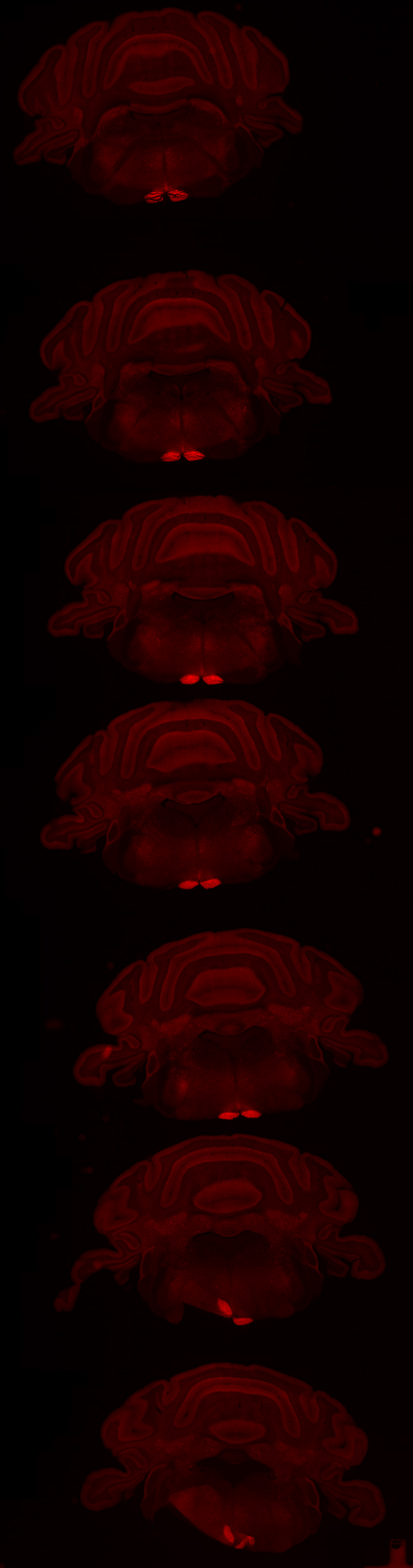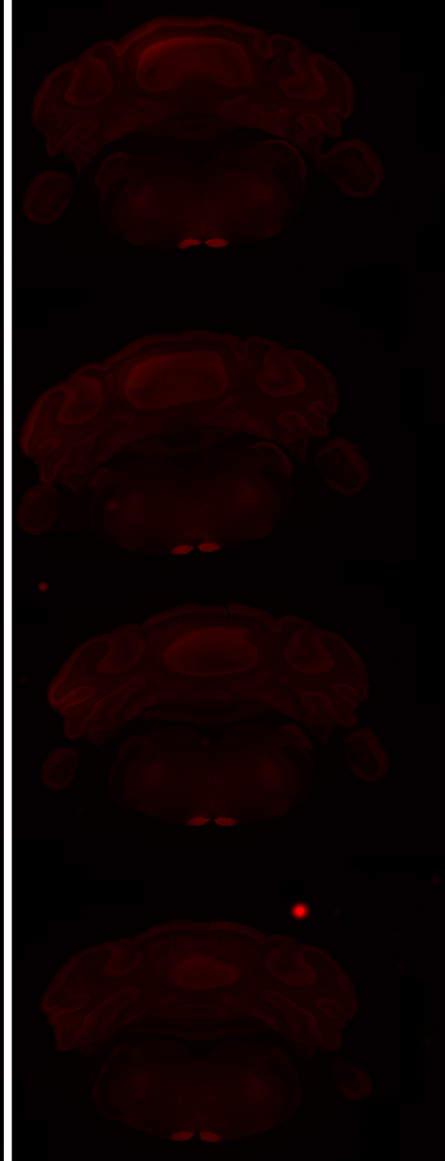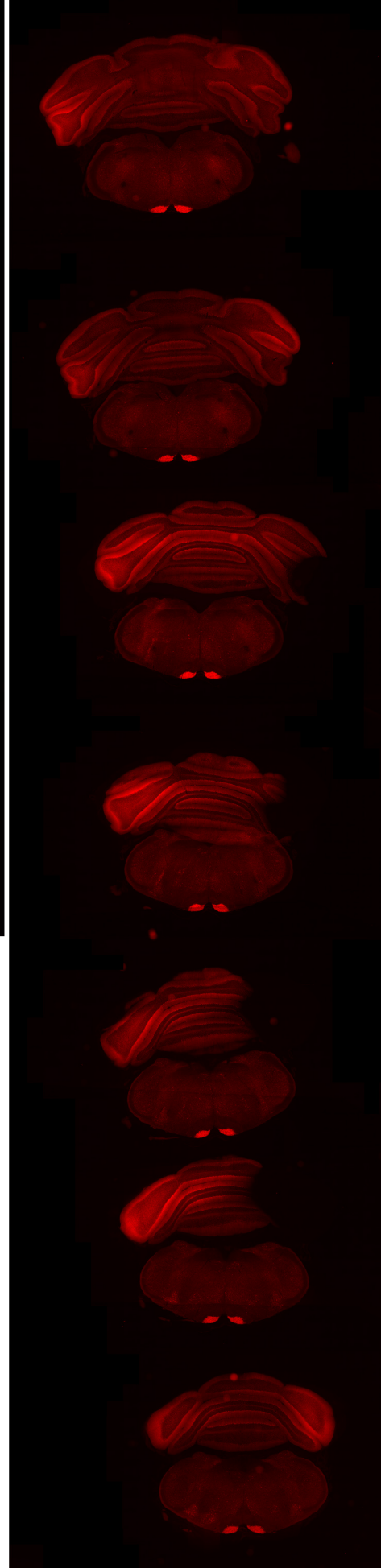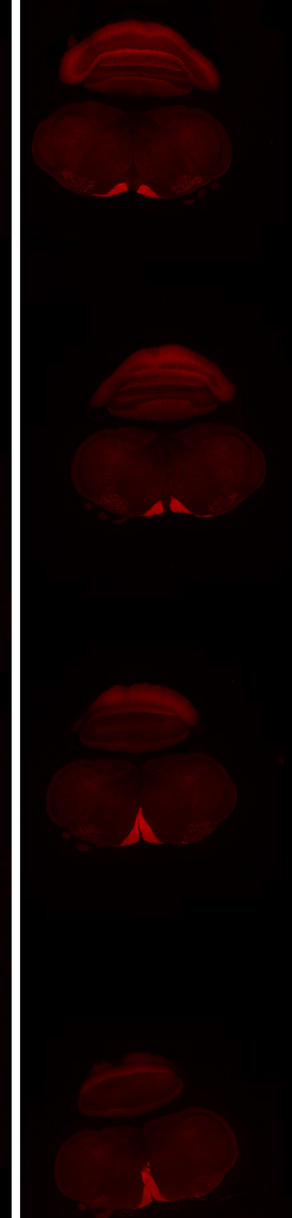

**GP 8.37**

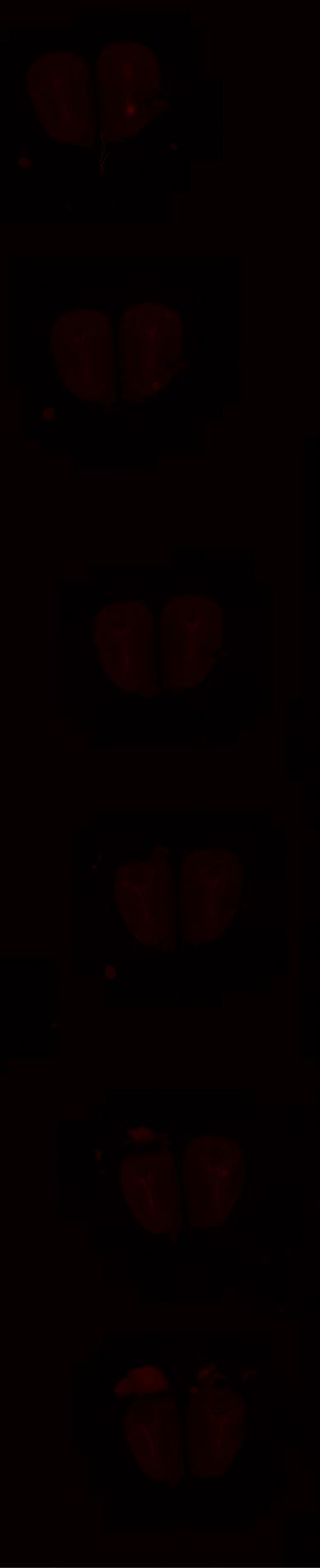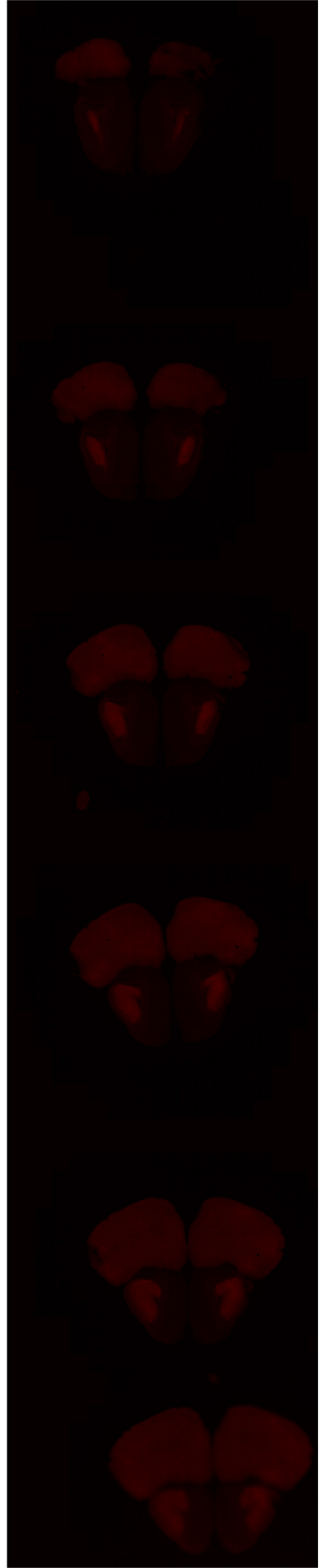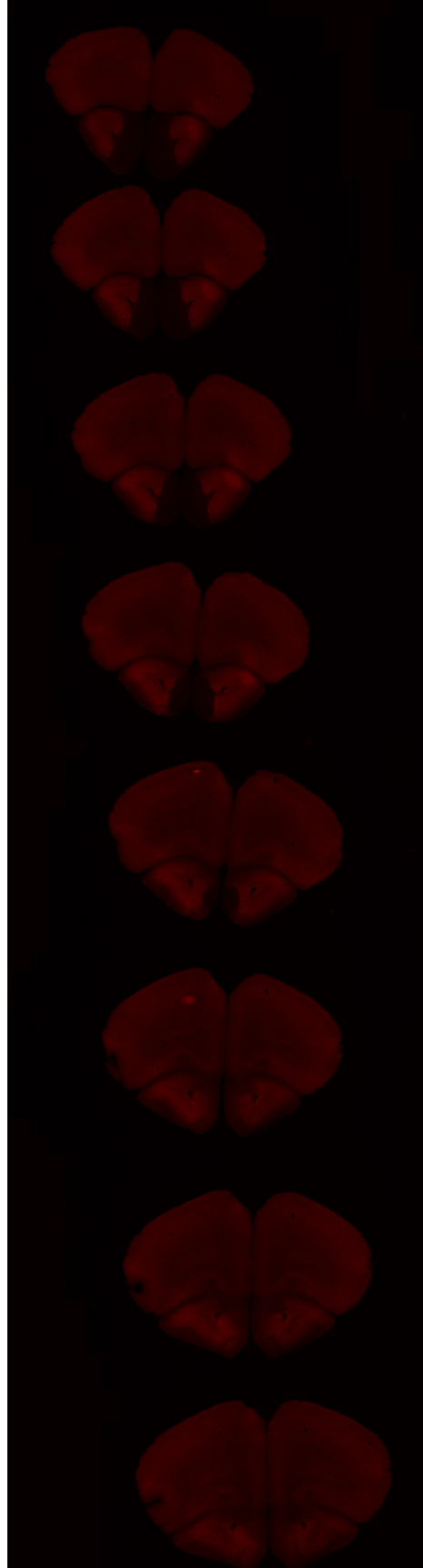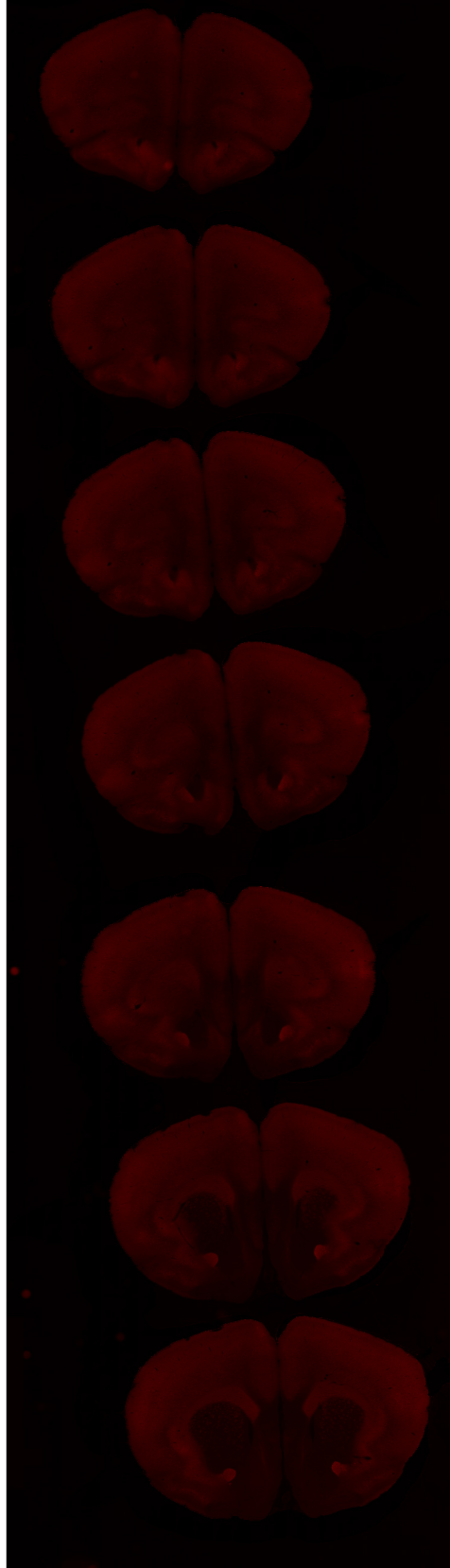

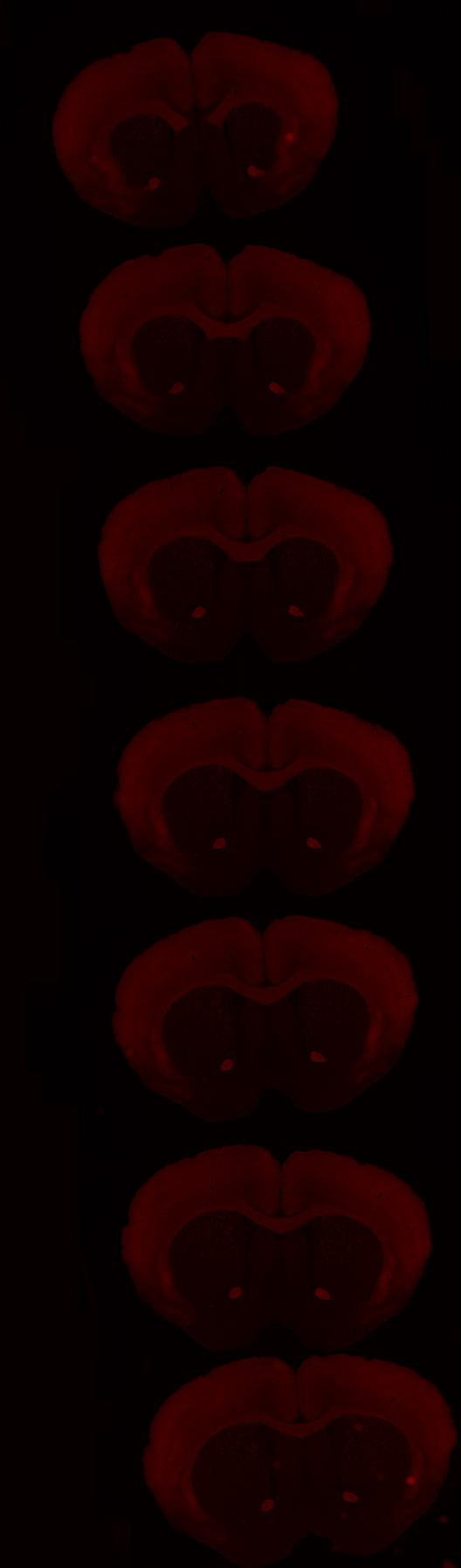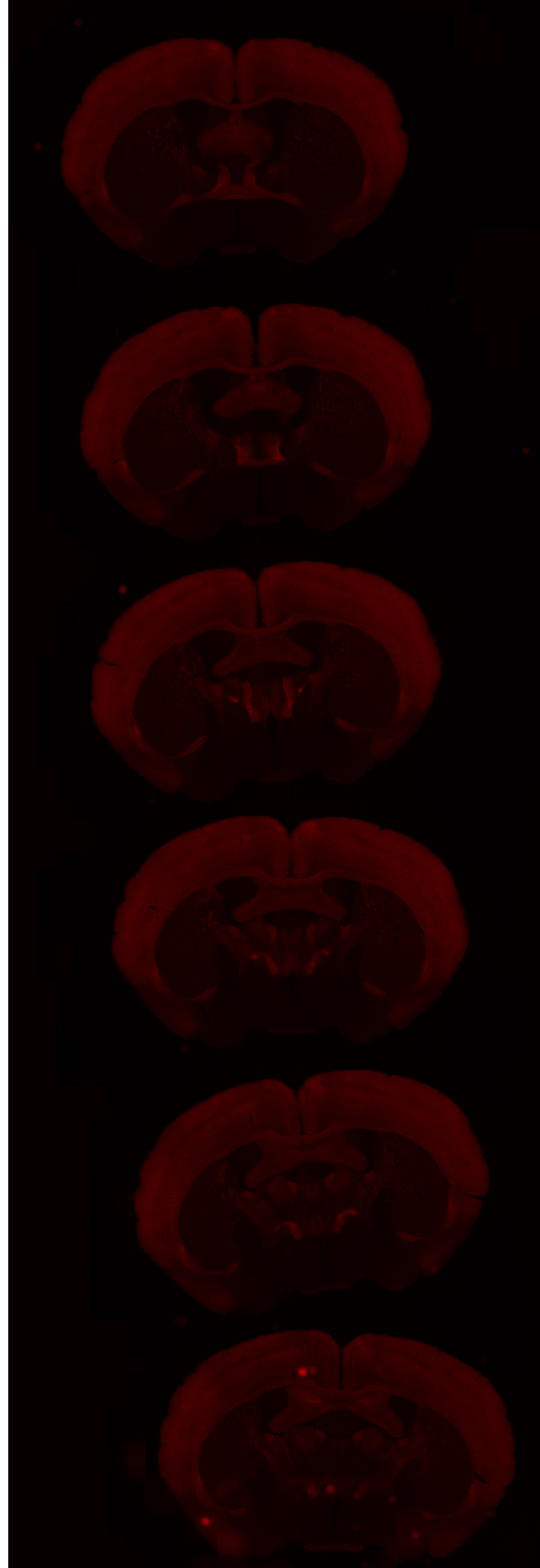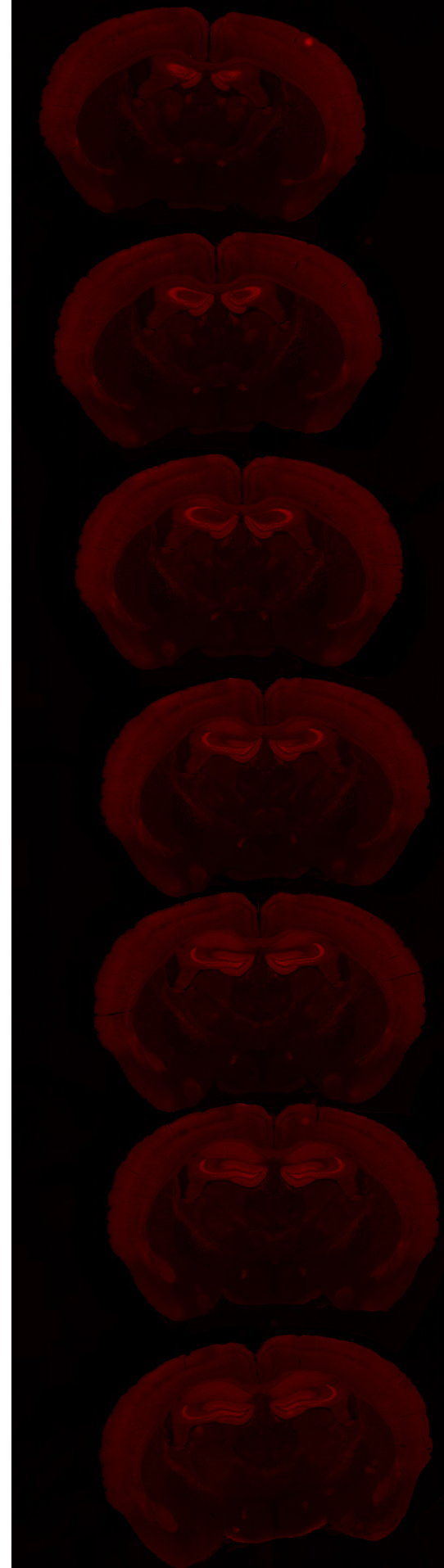

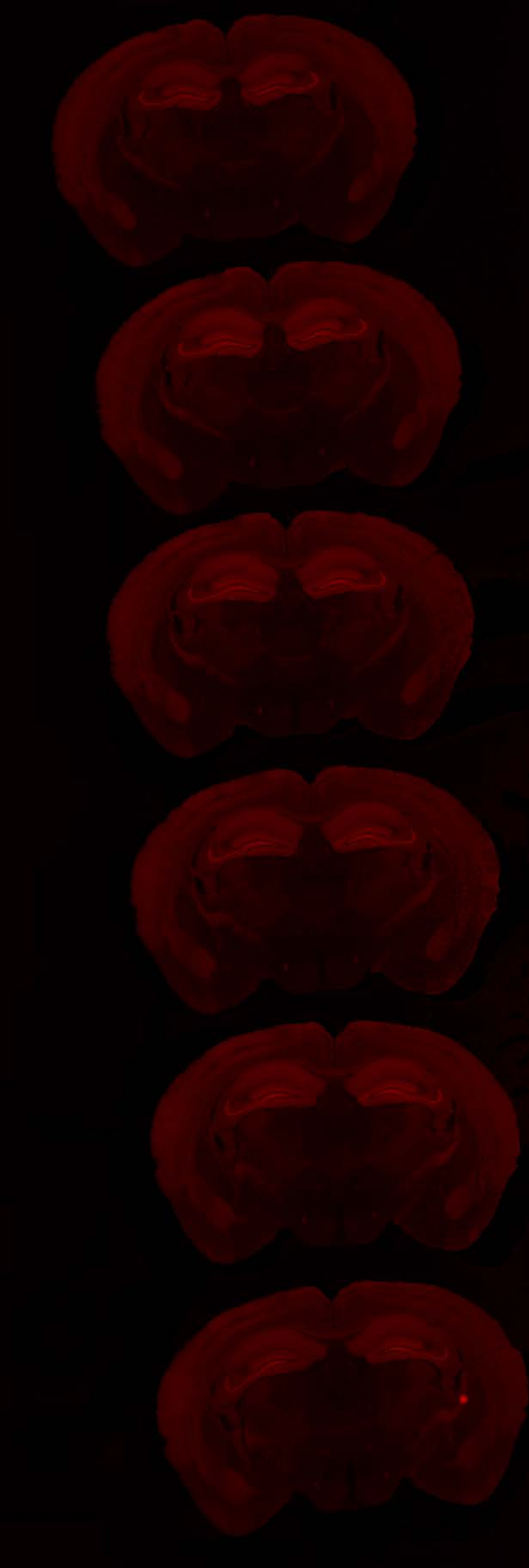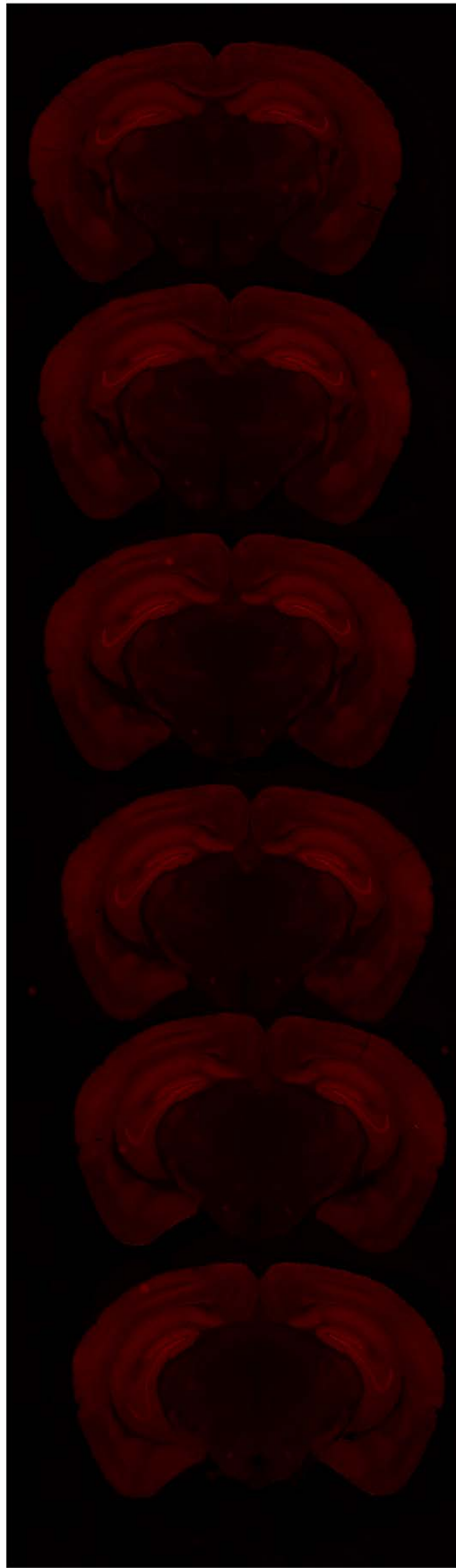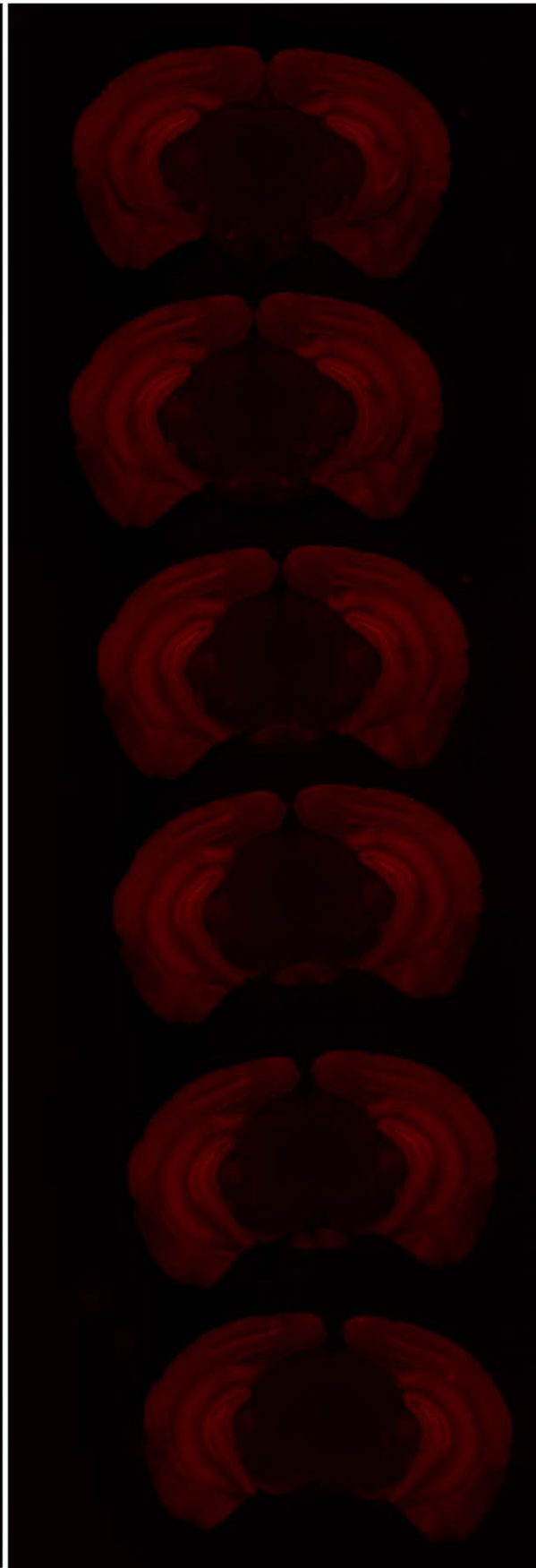

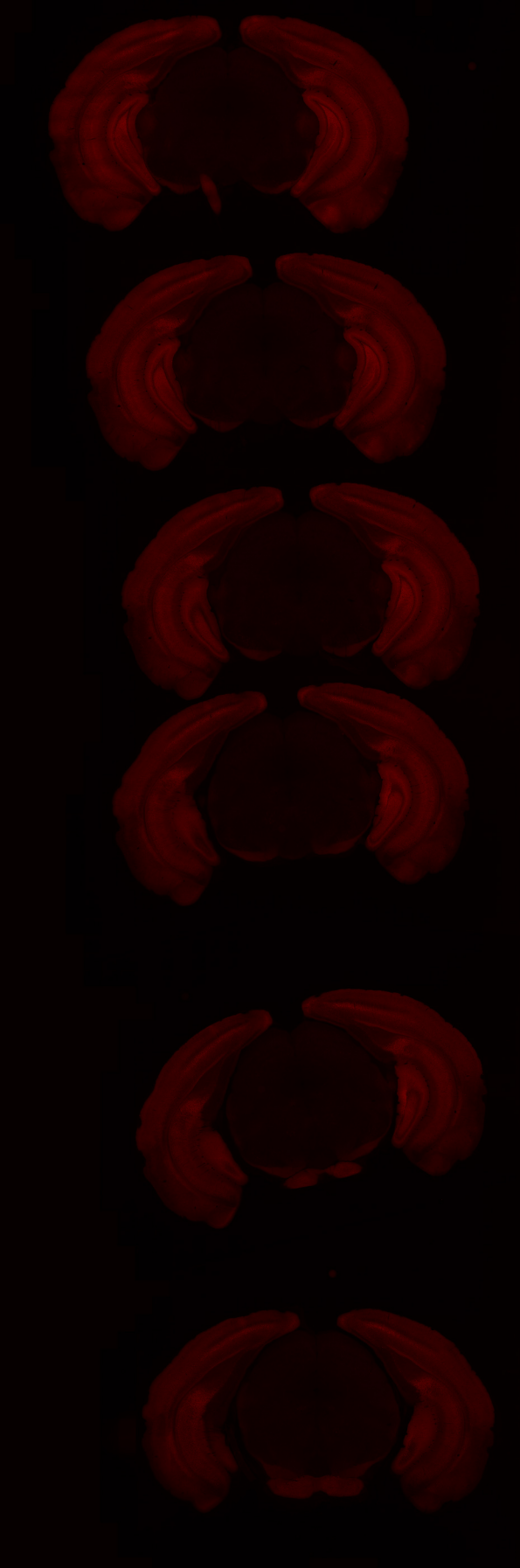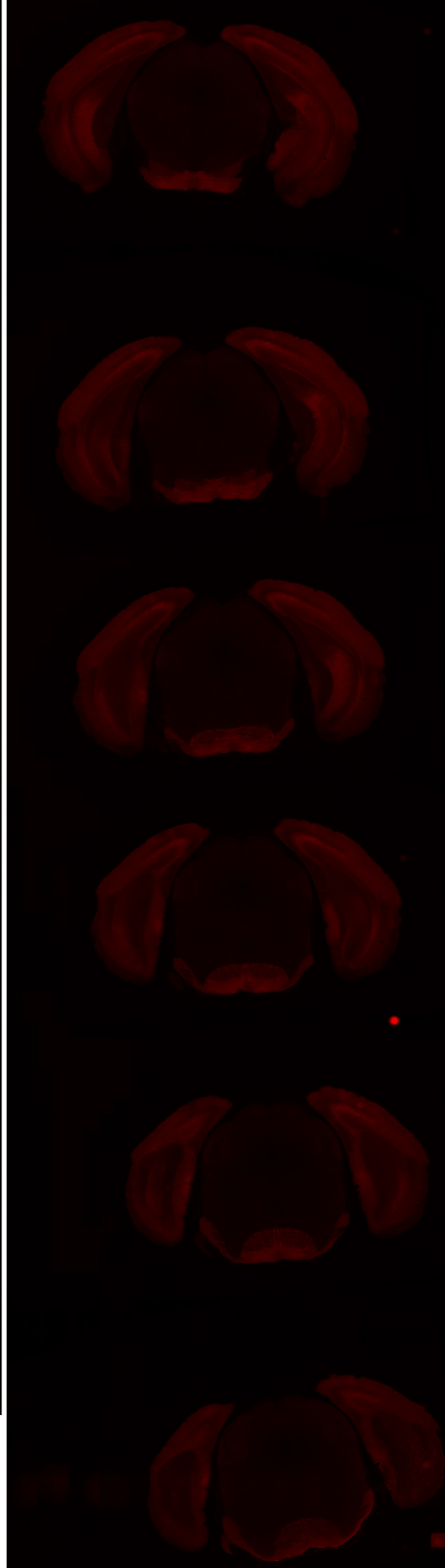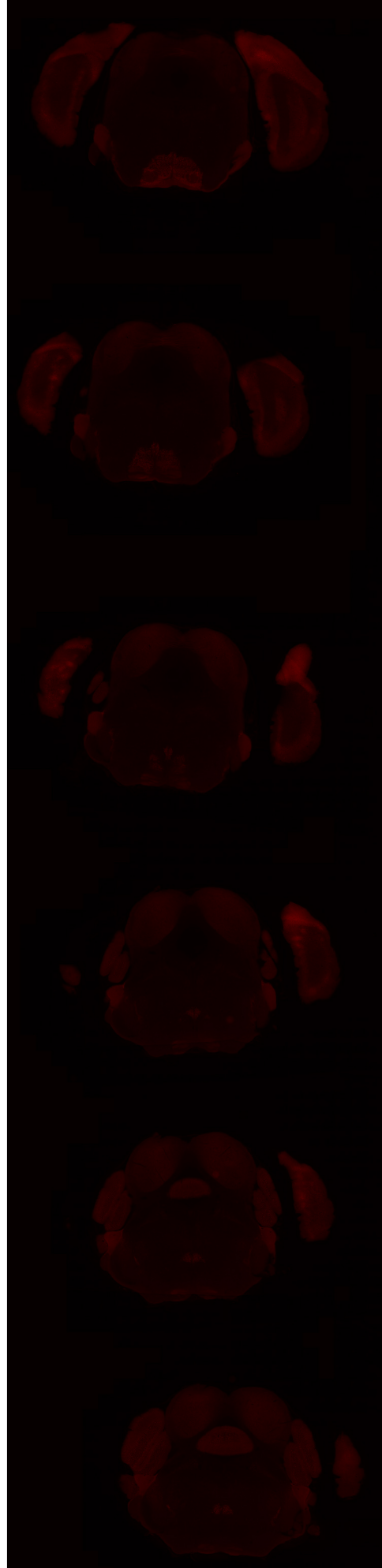

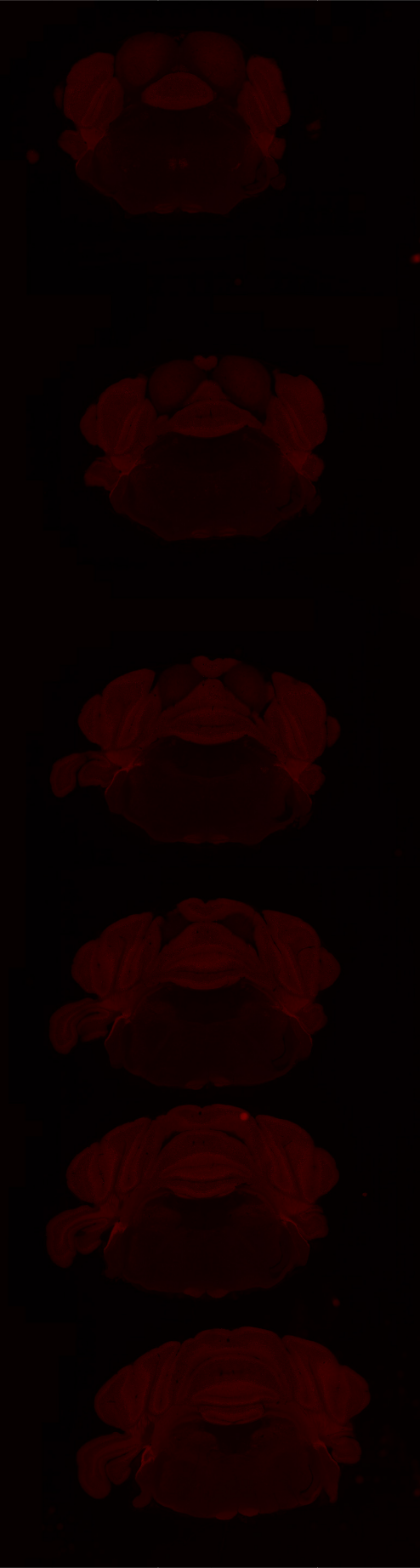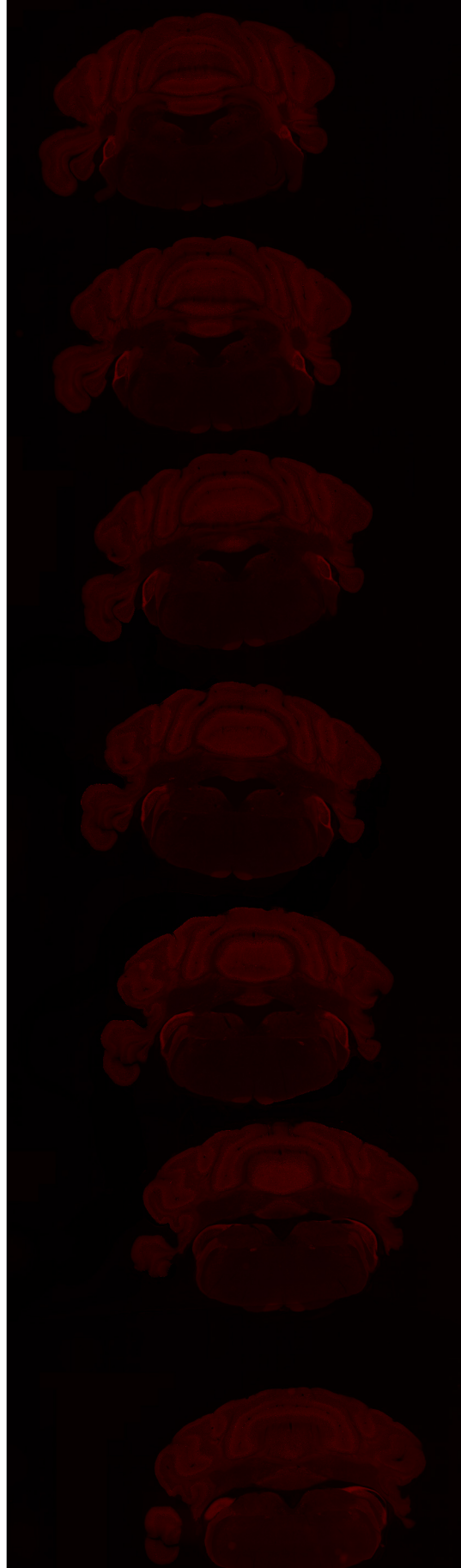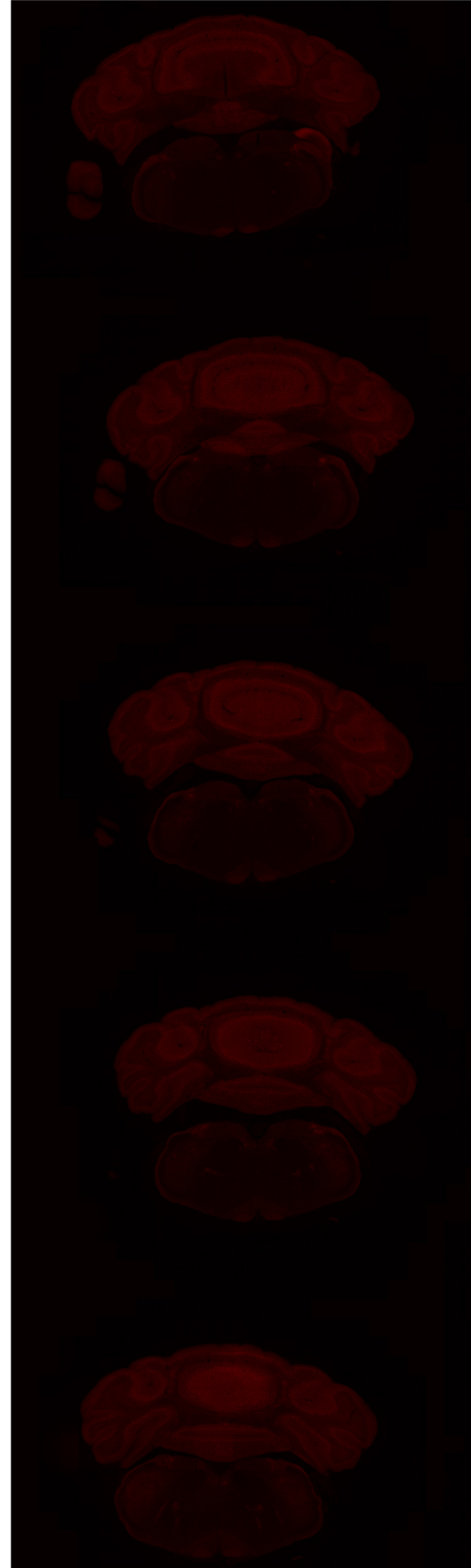

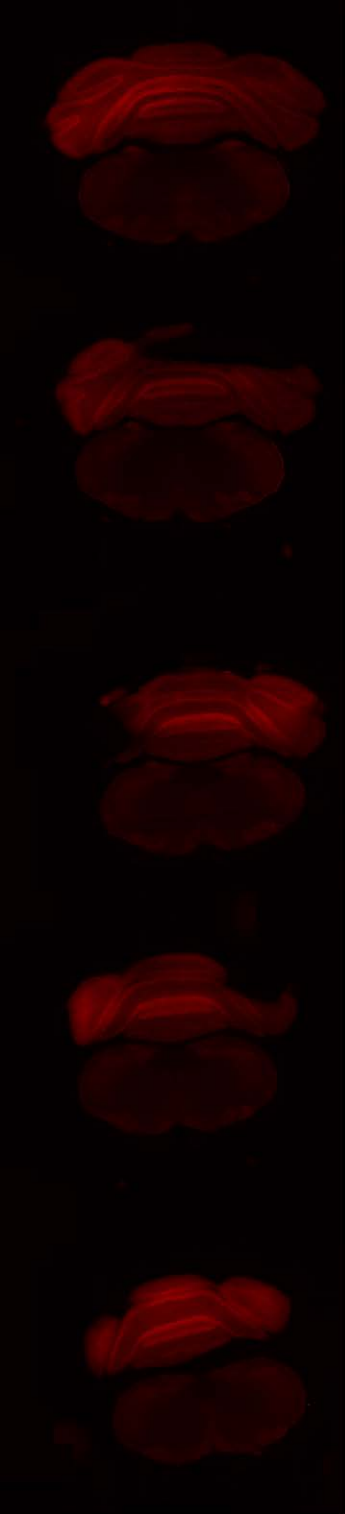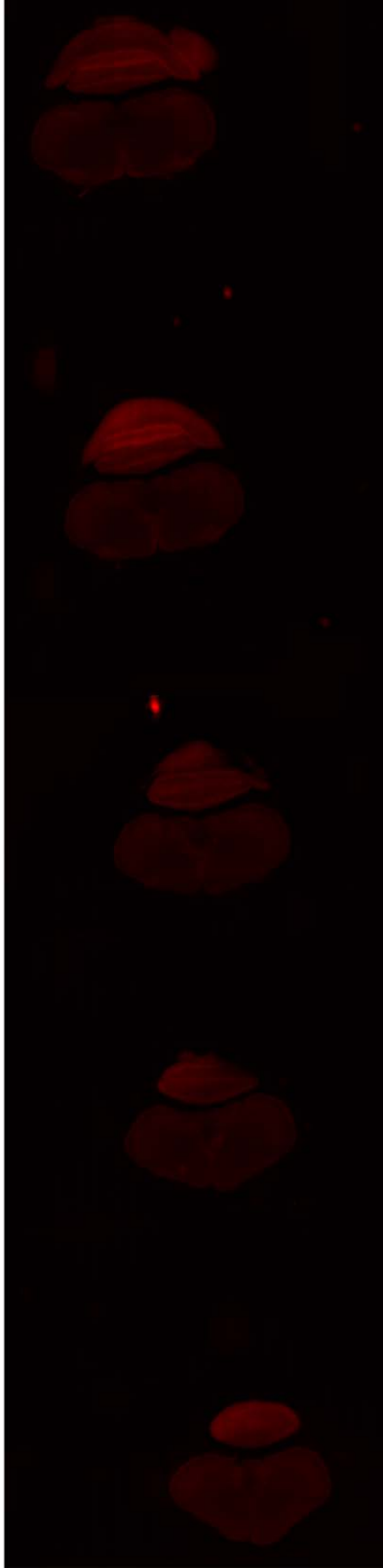

**GP 8.40**

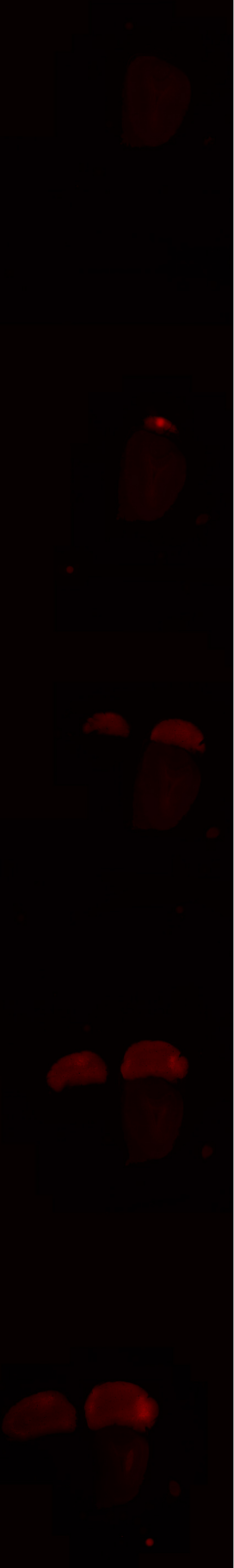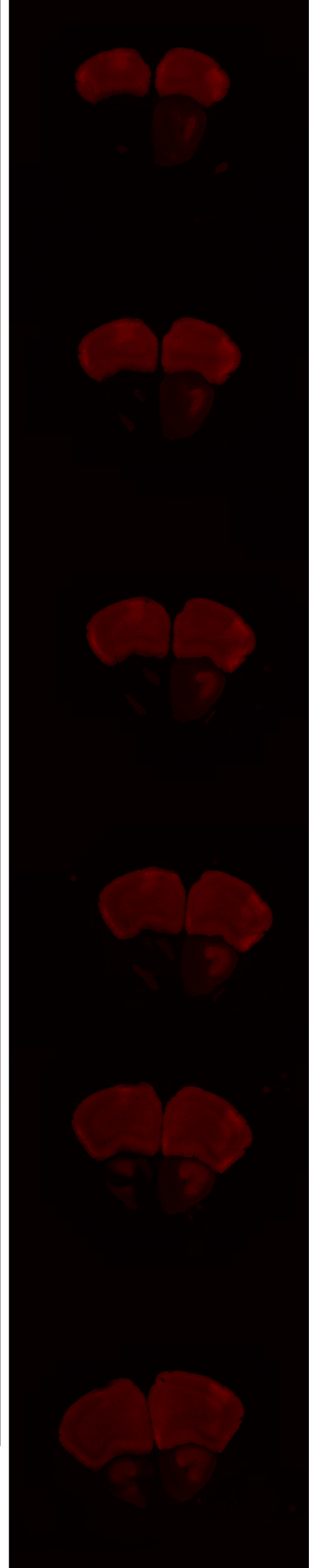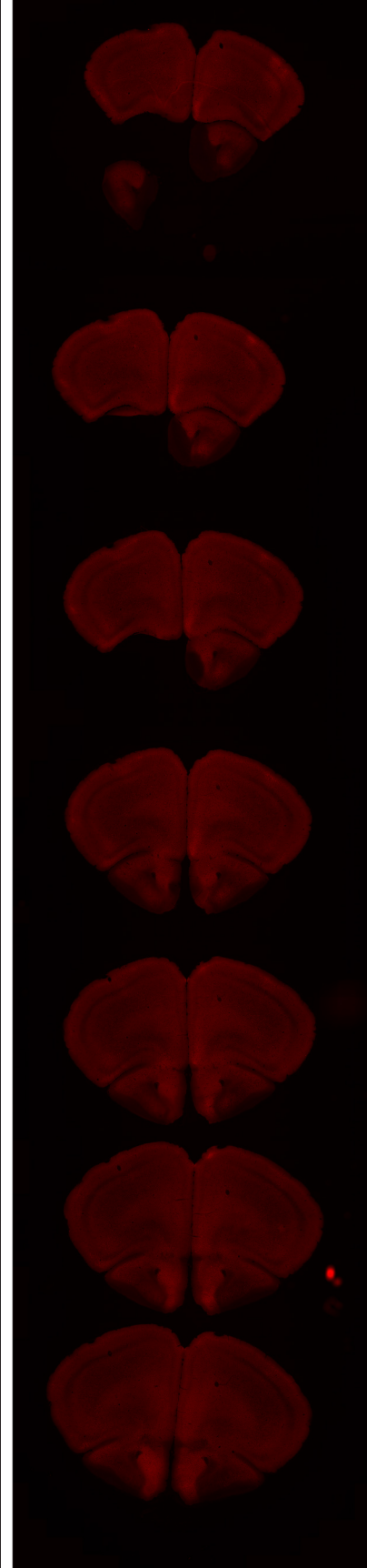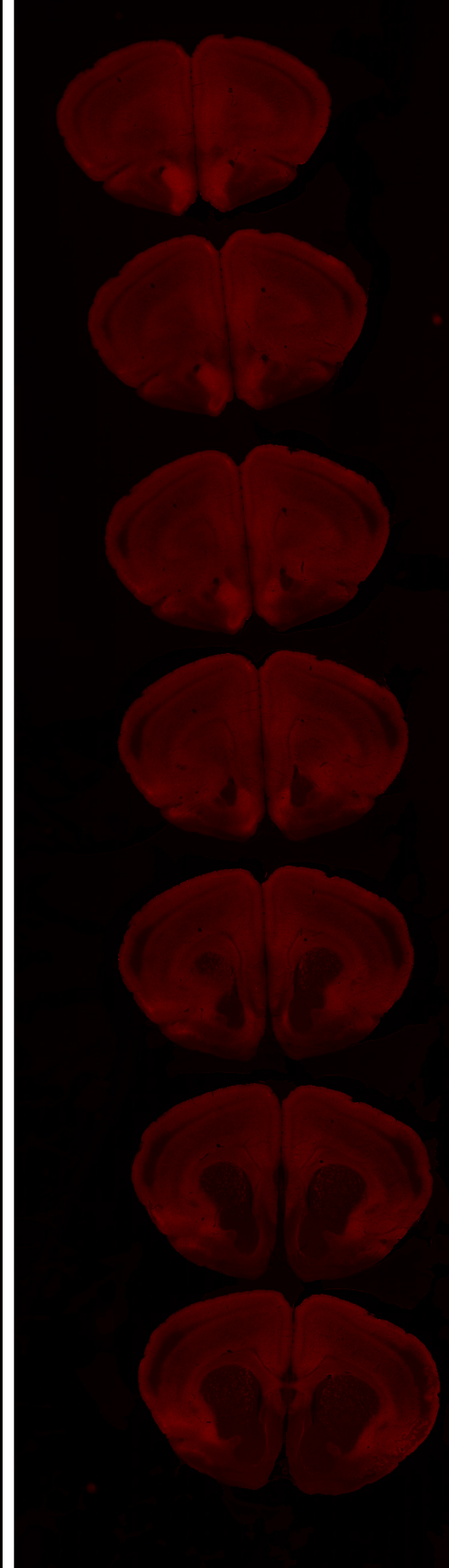

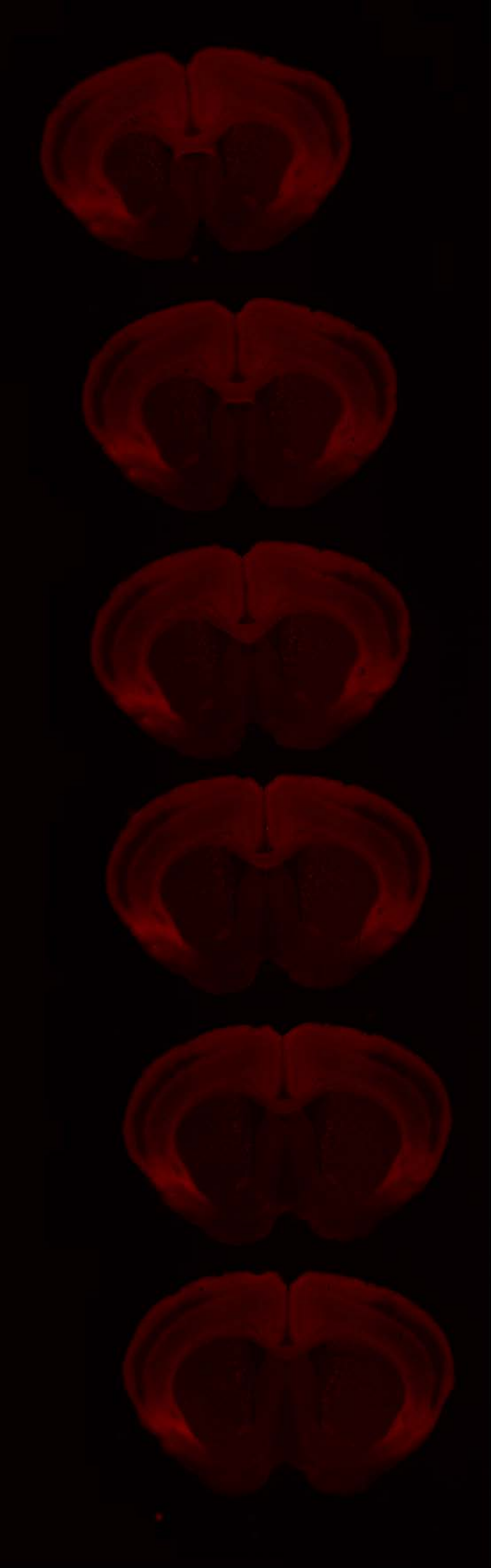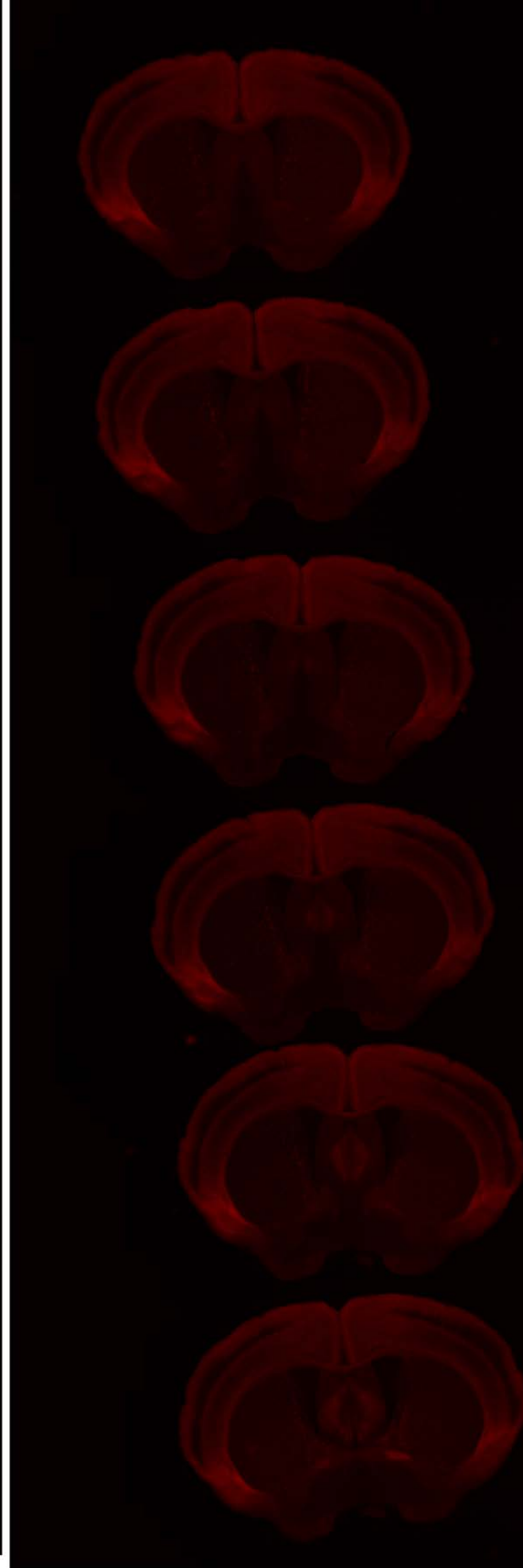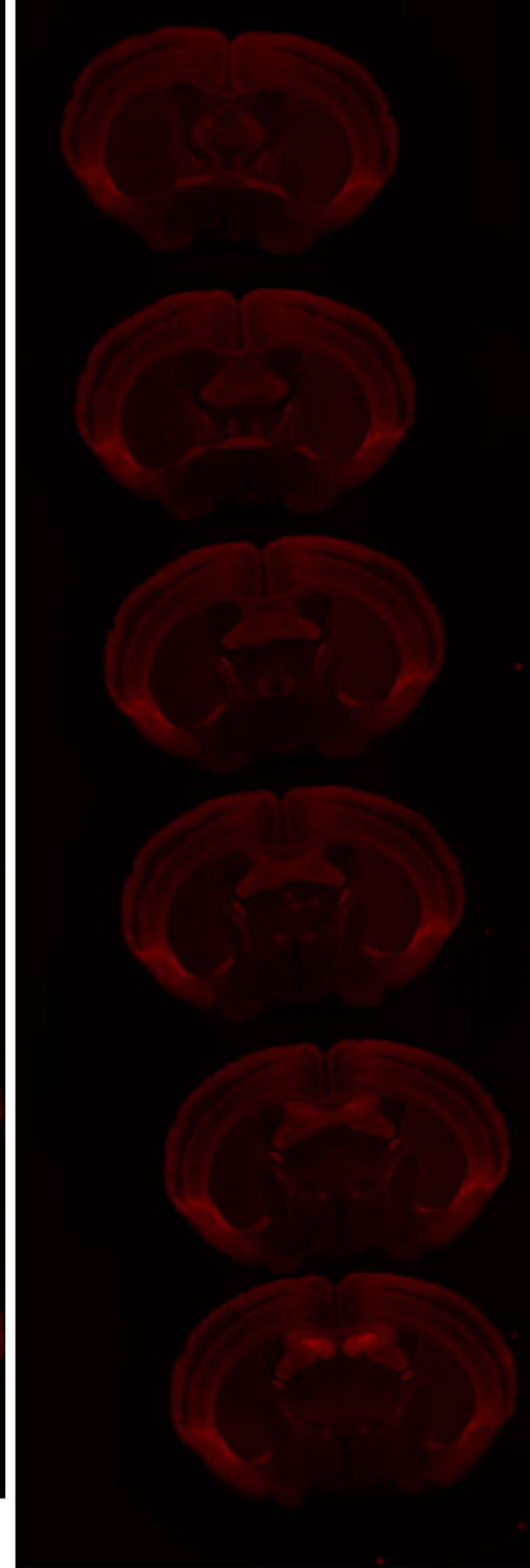

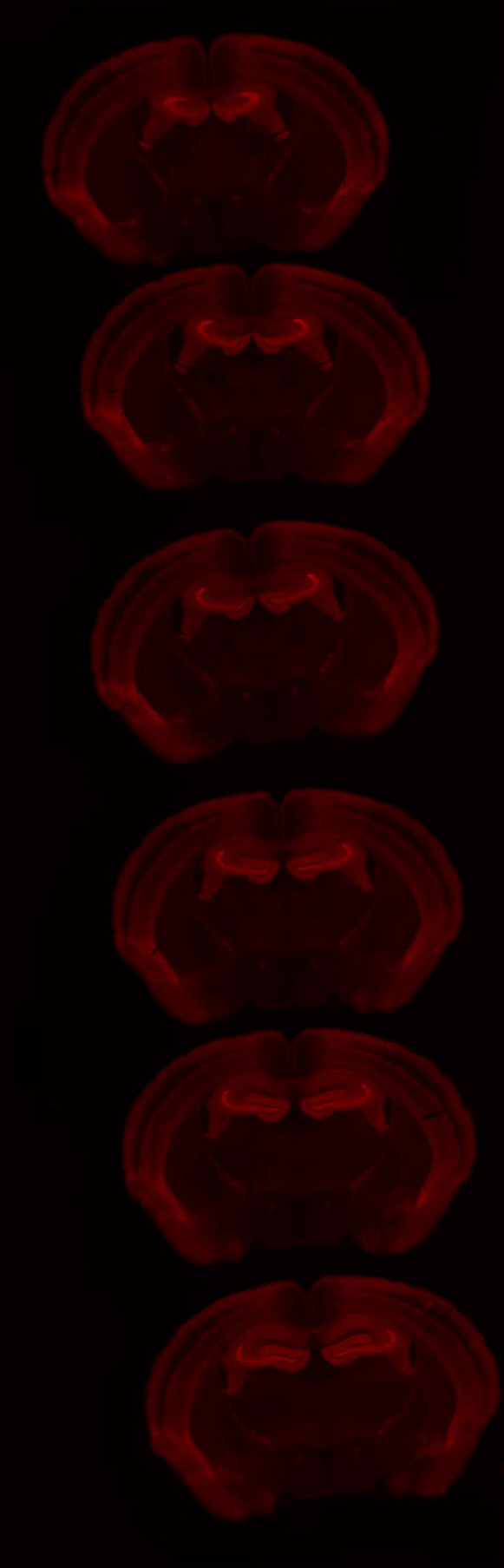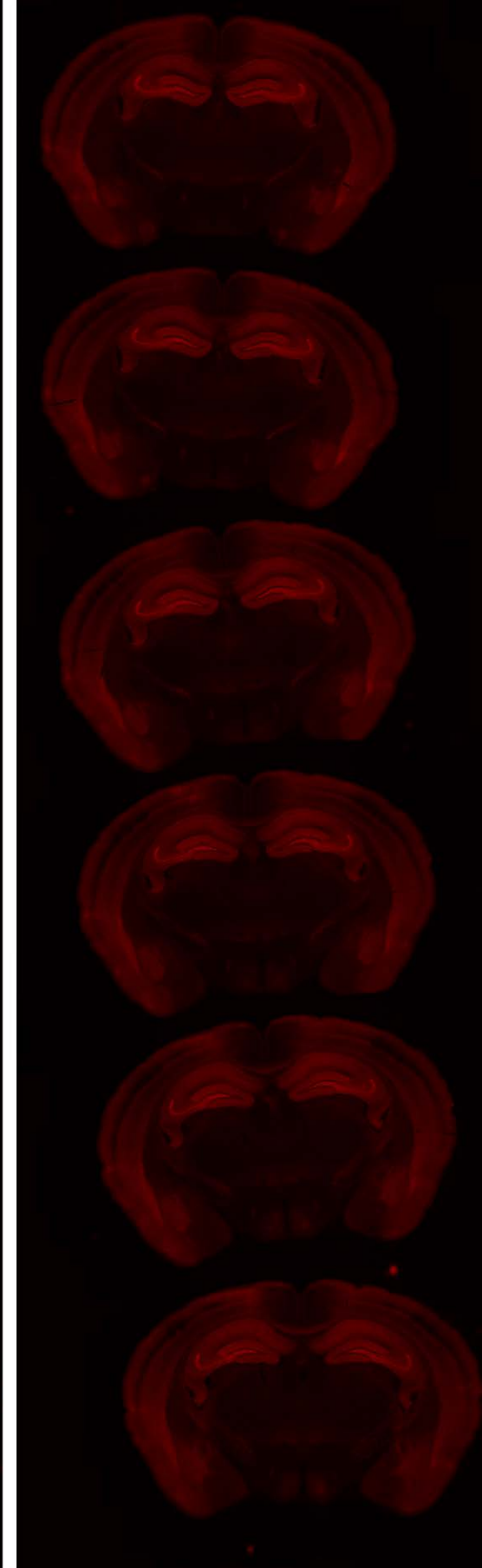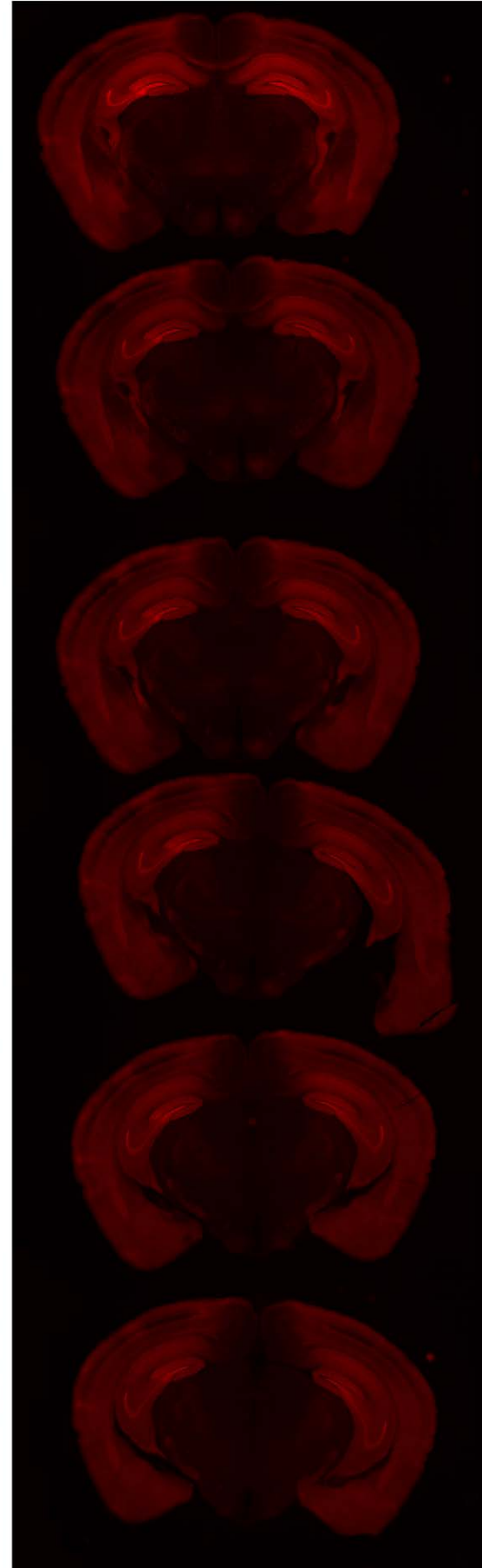

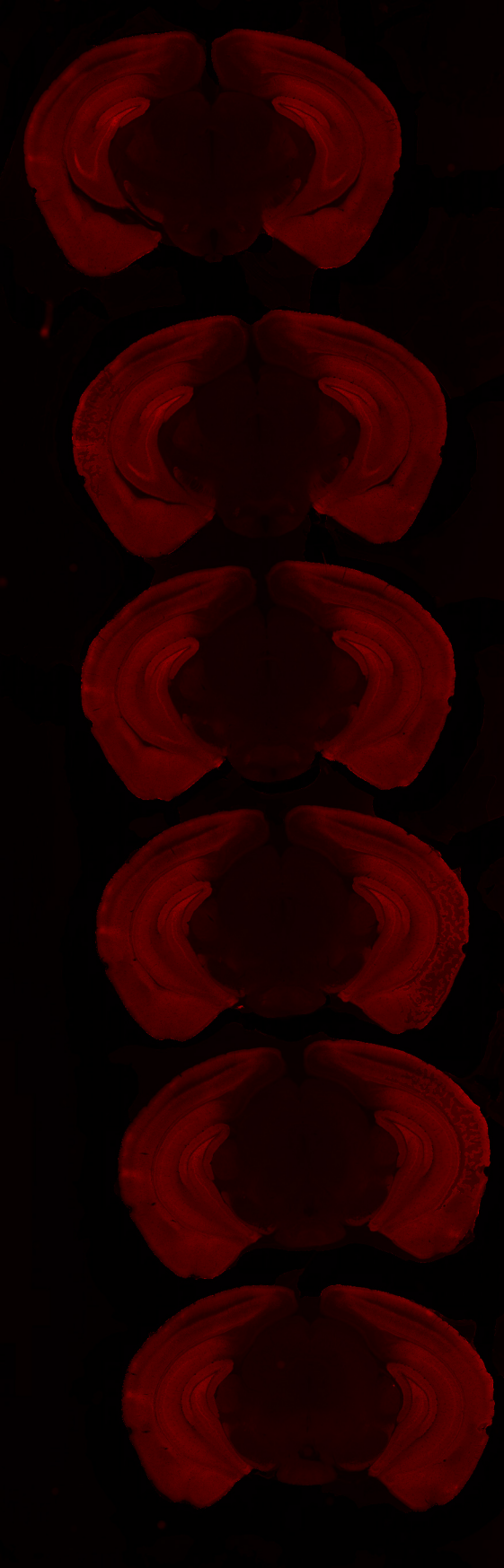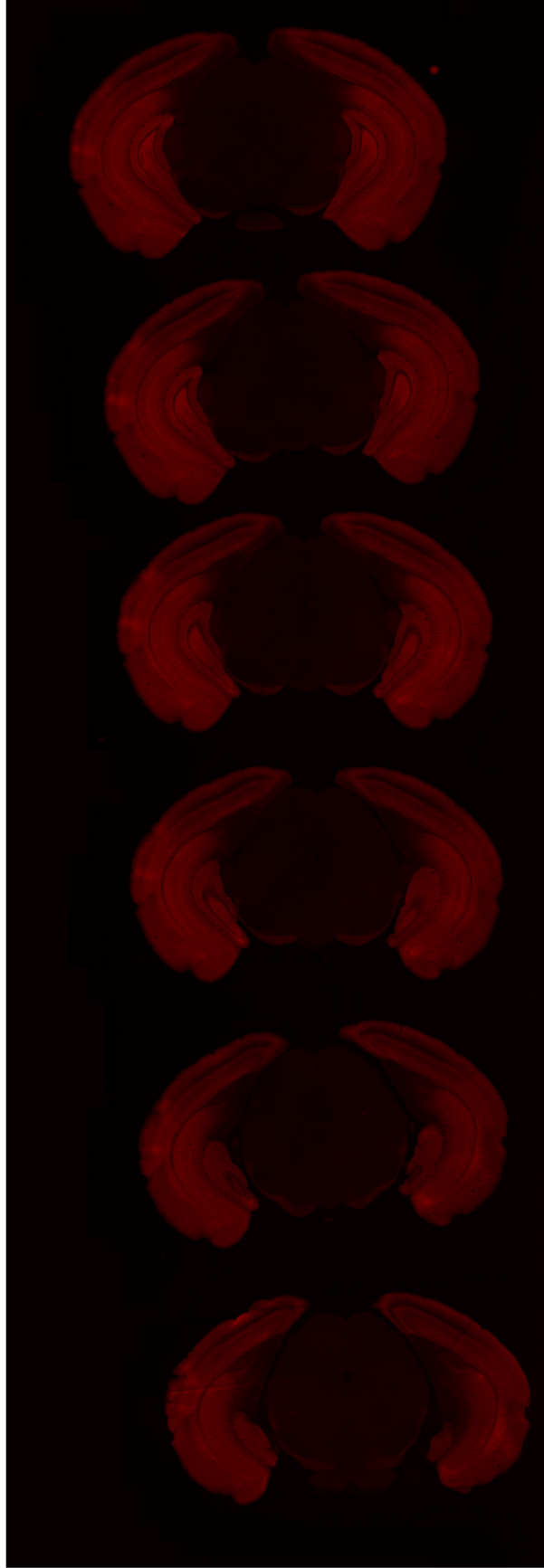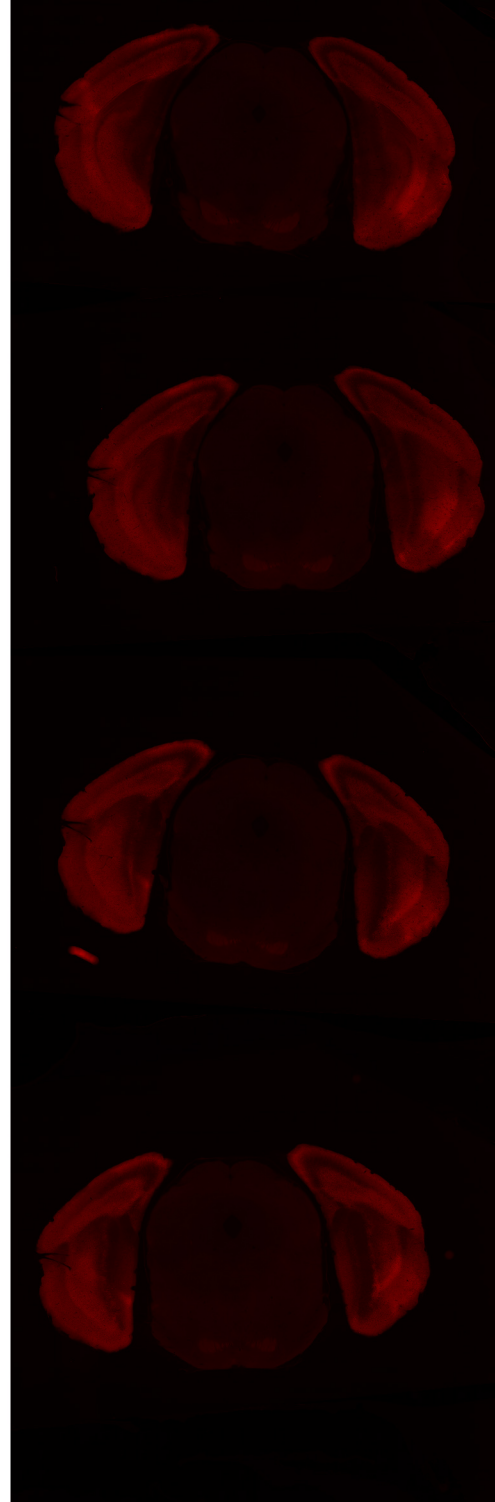

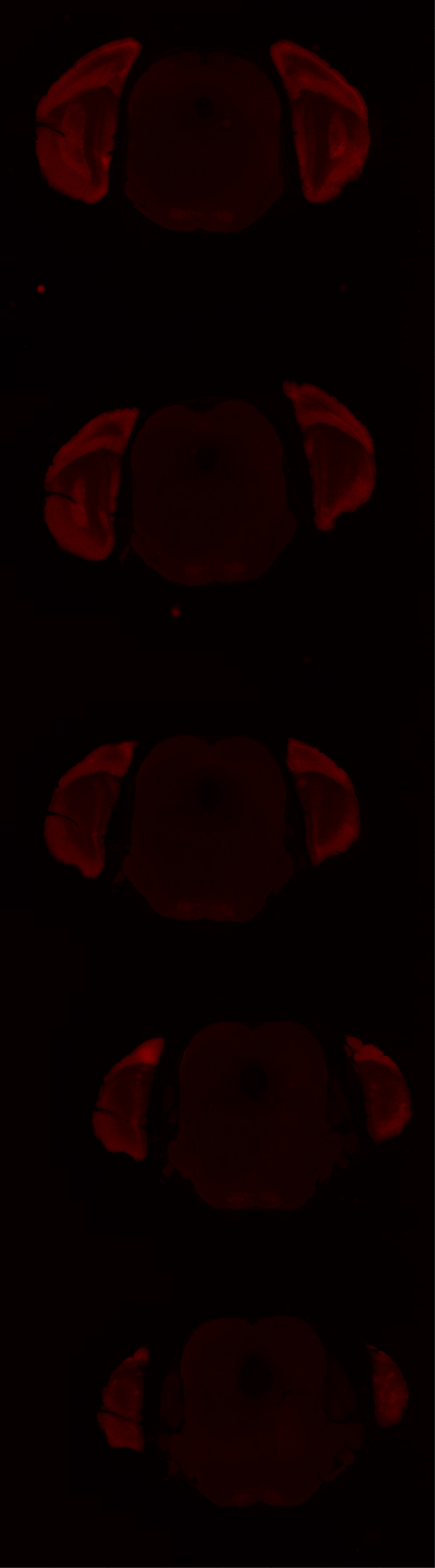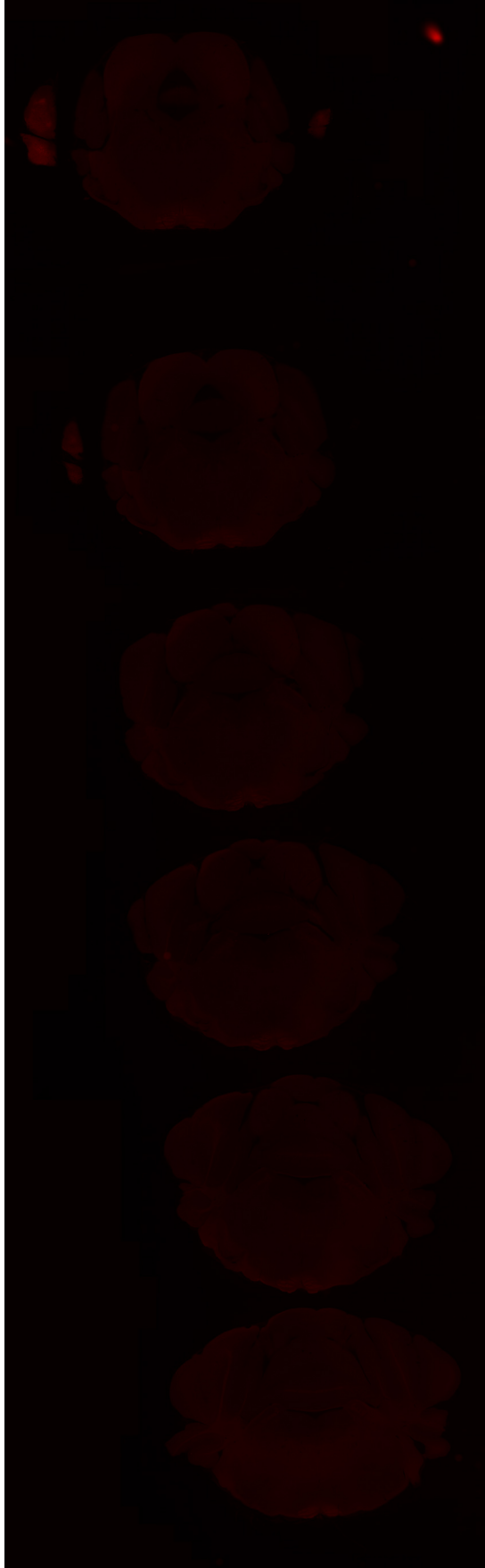

**GP 8.46**

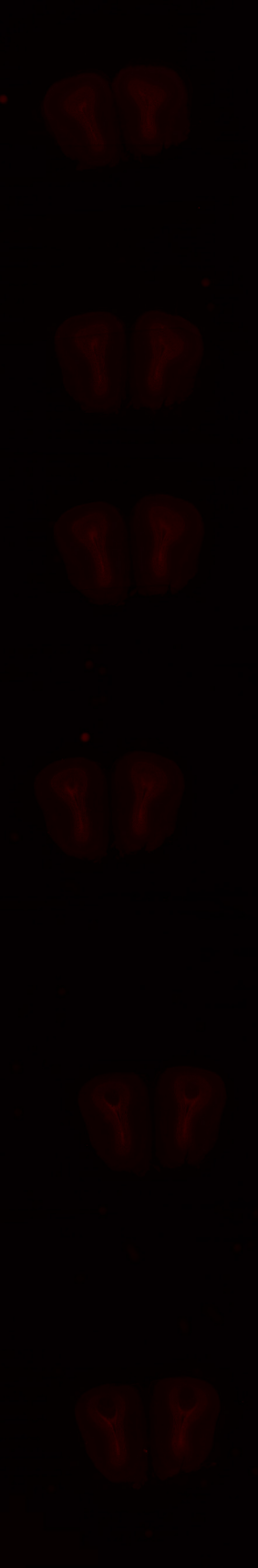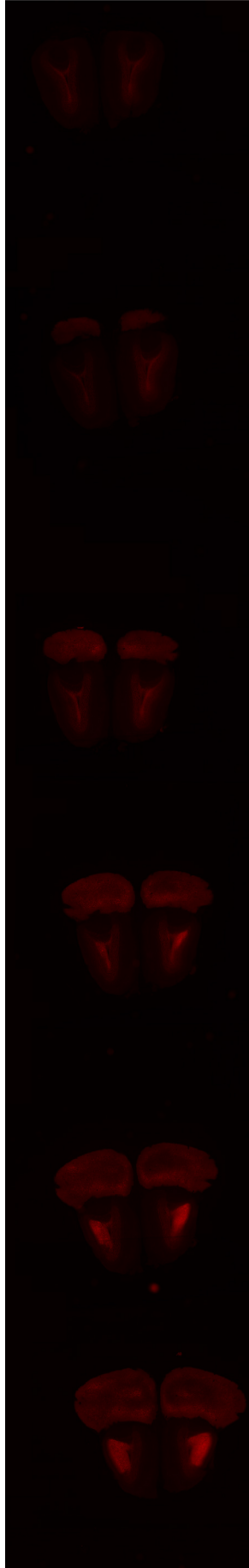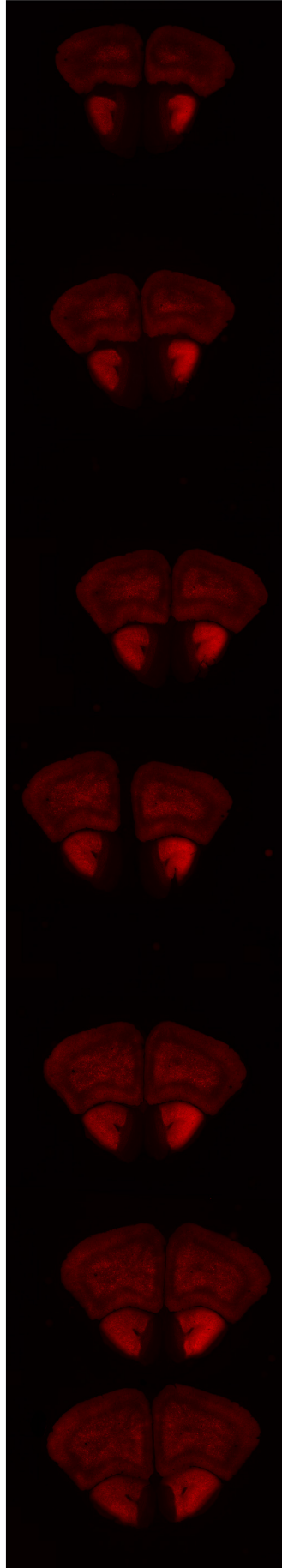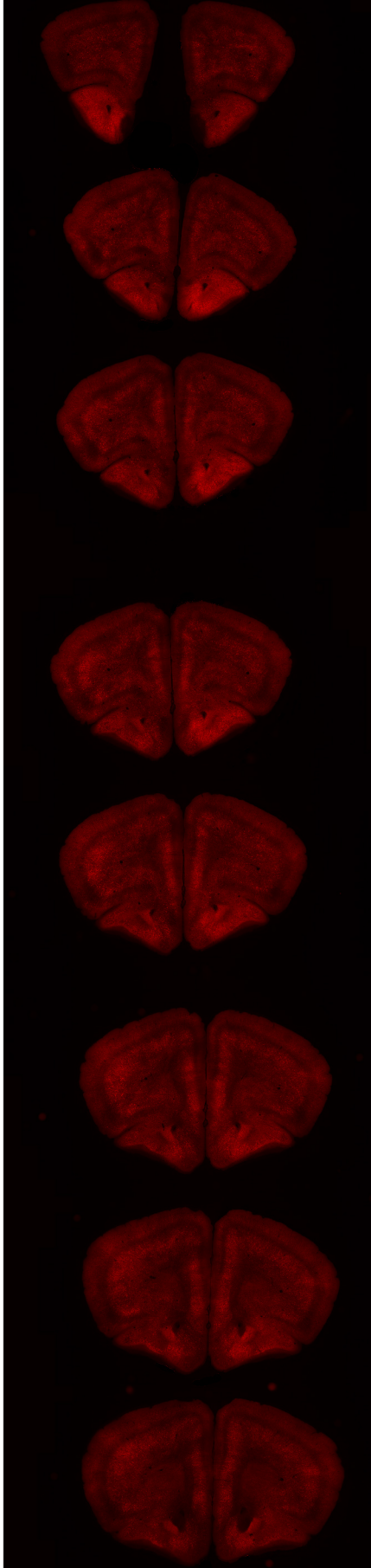

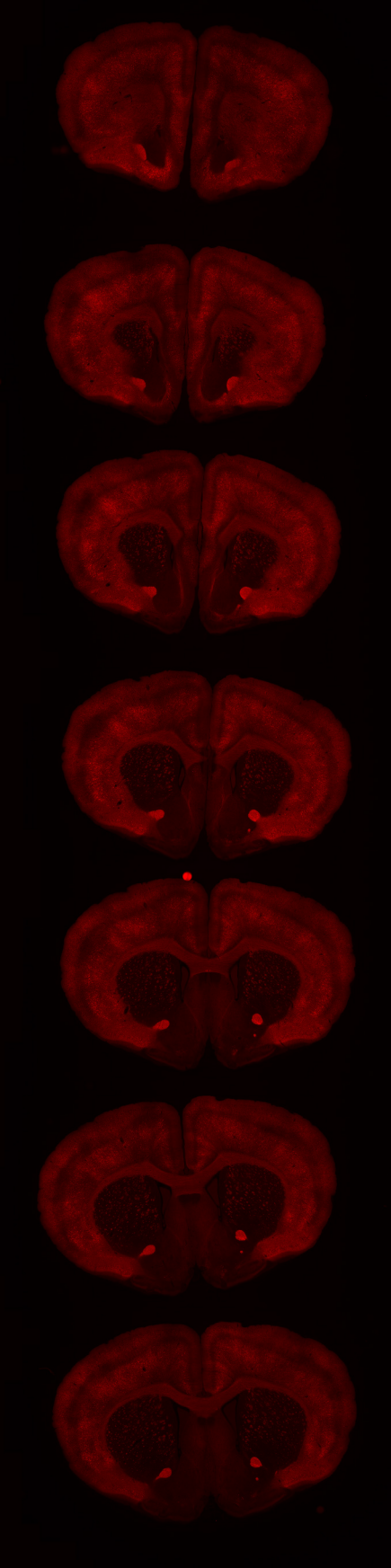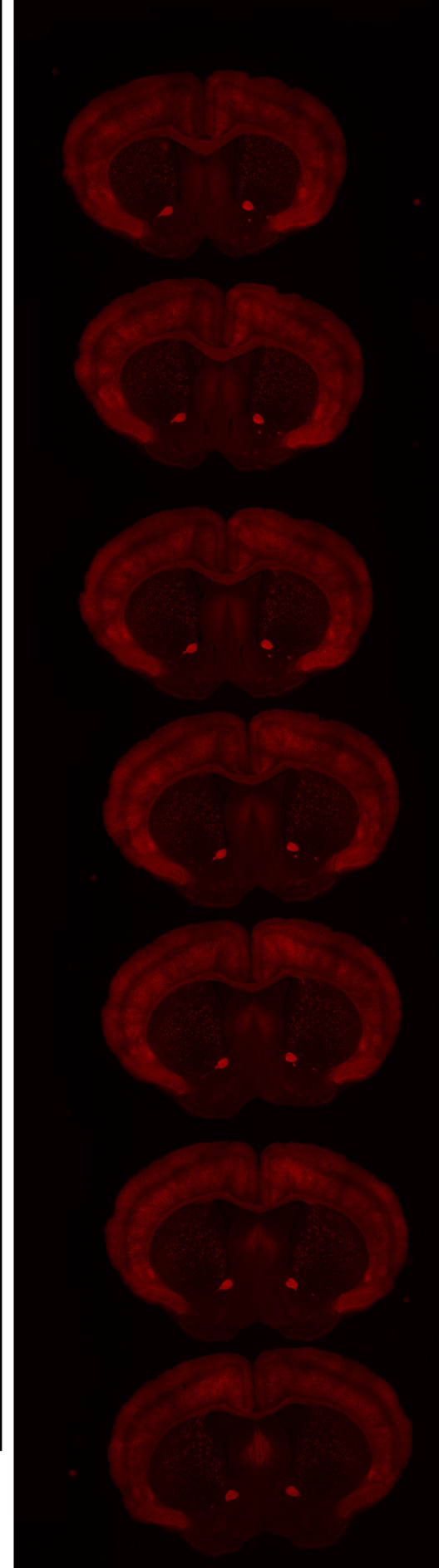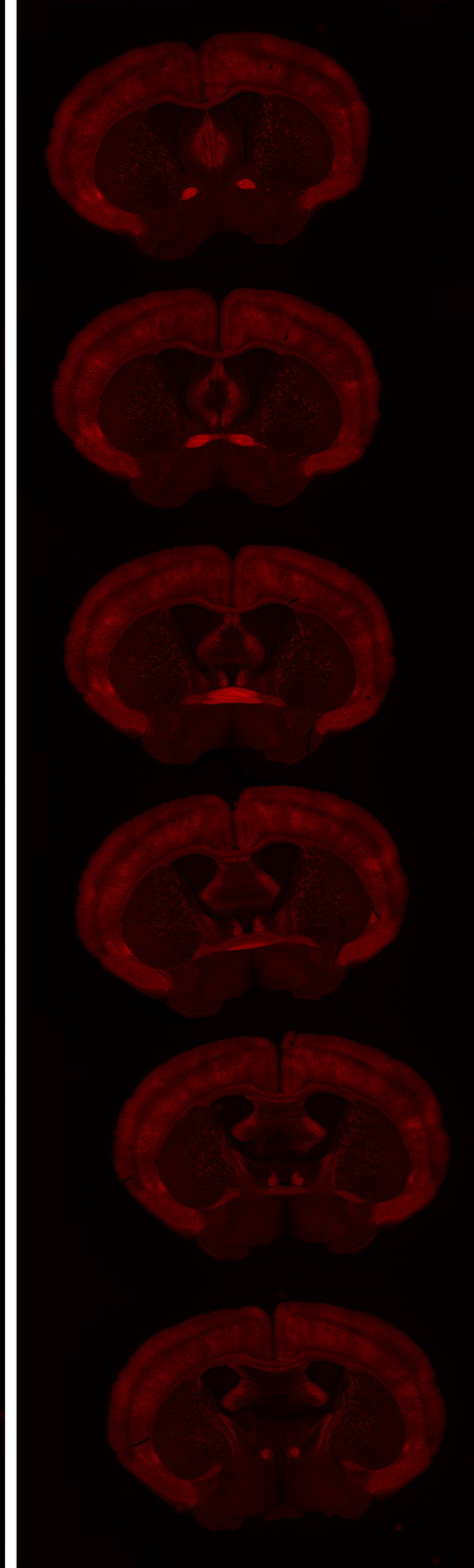

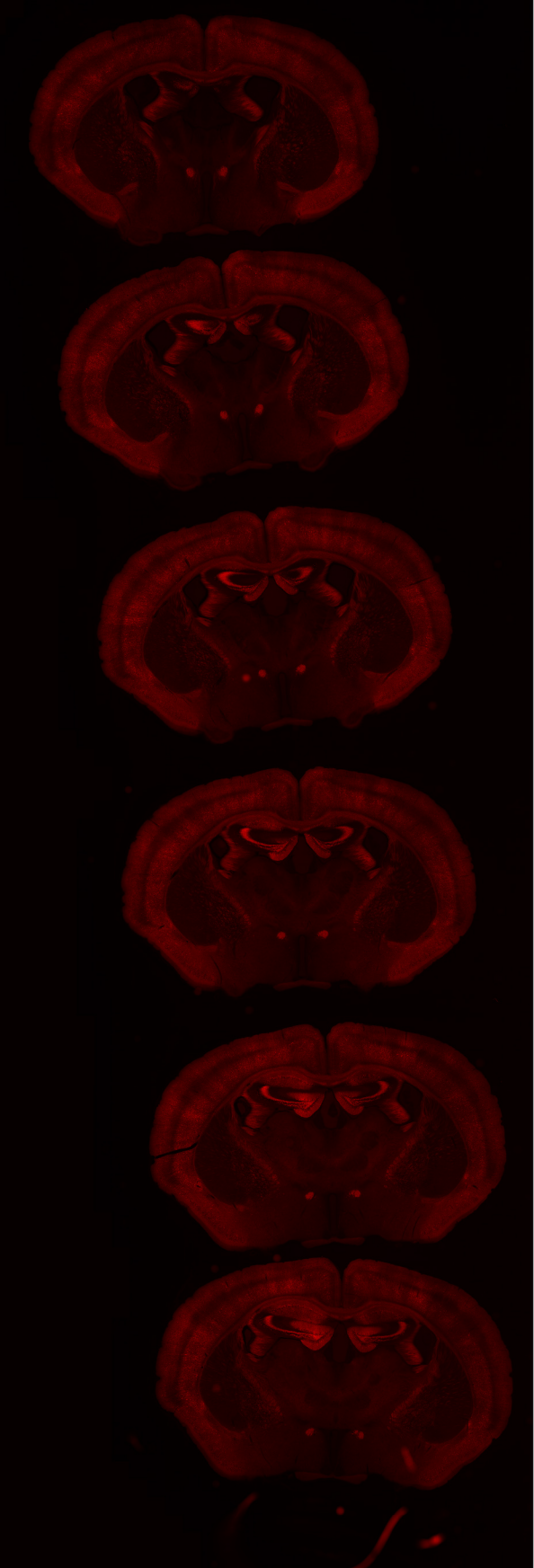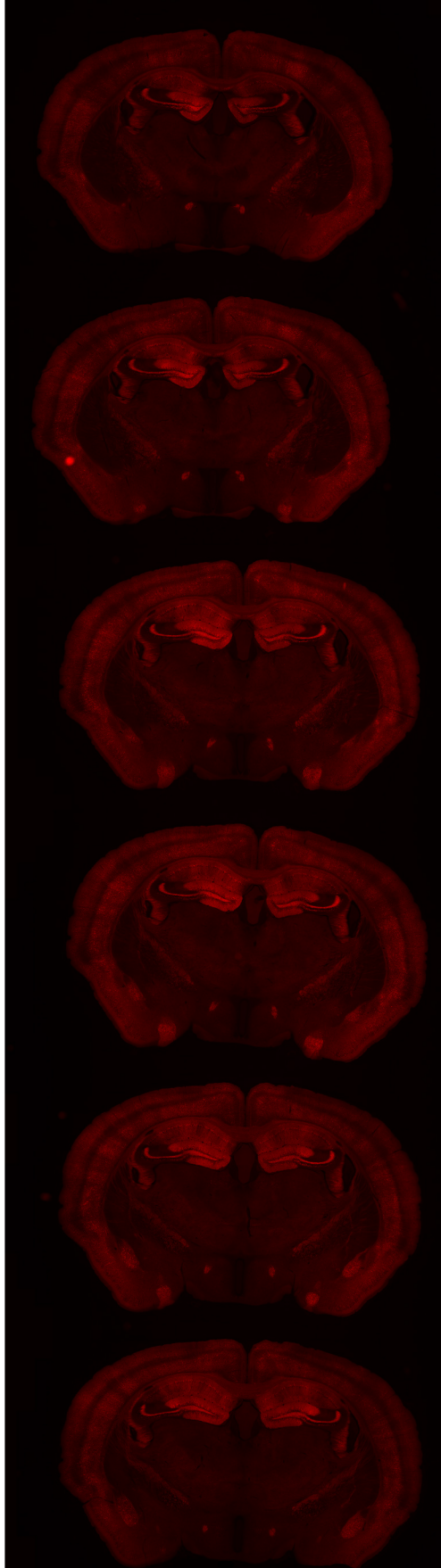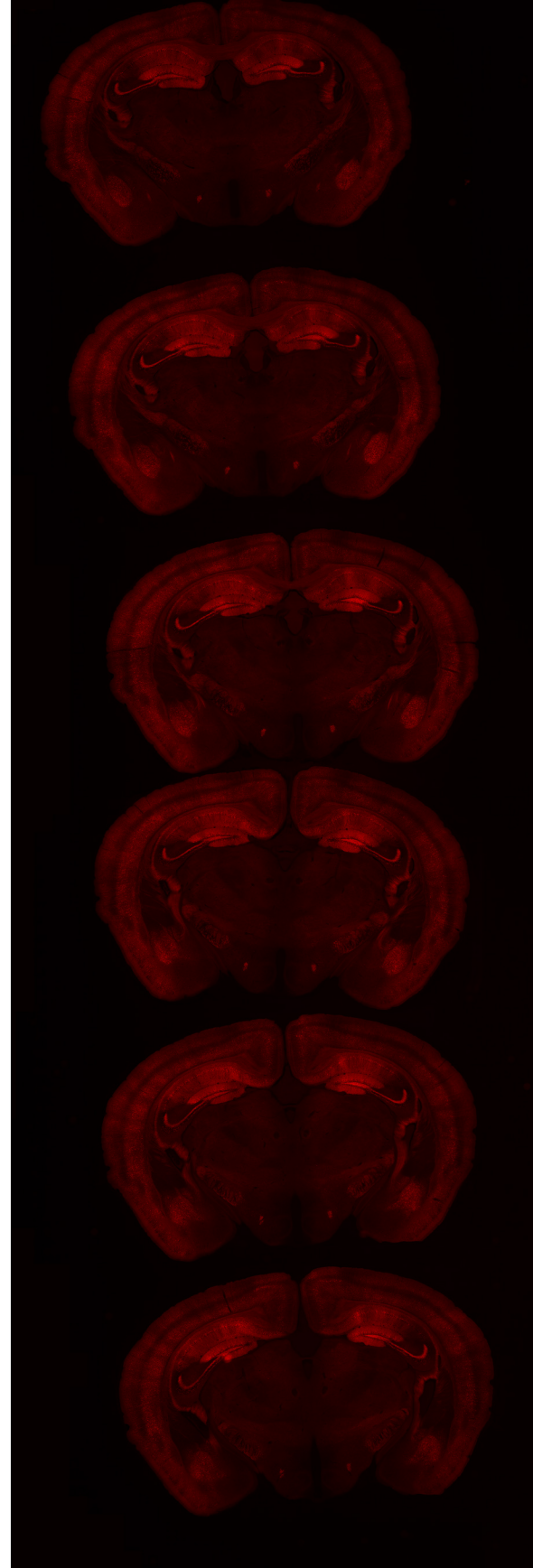

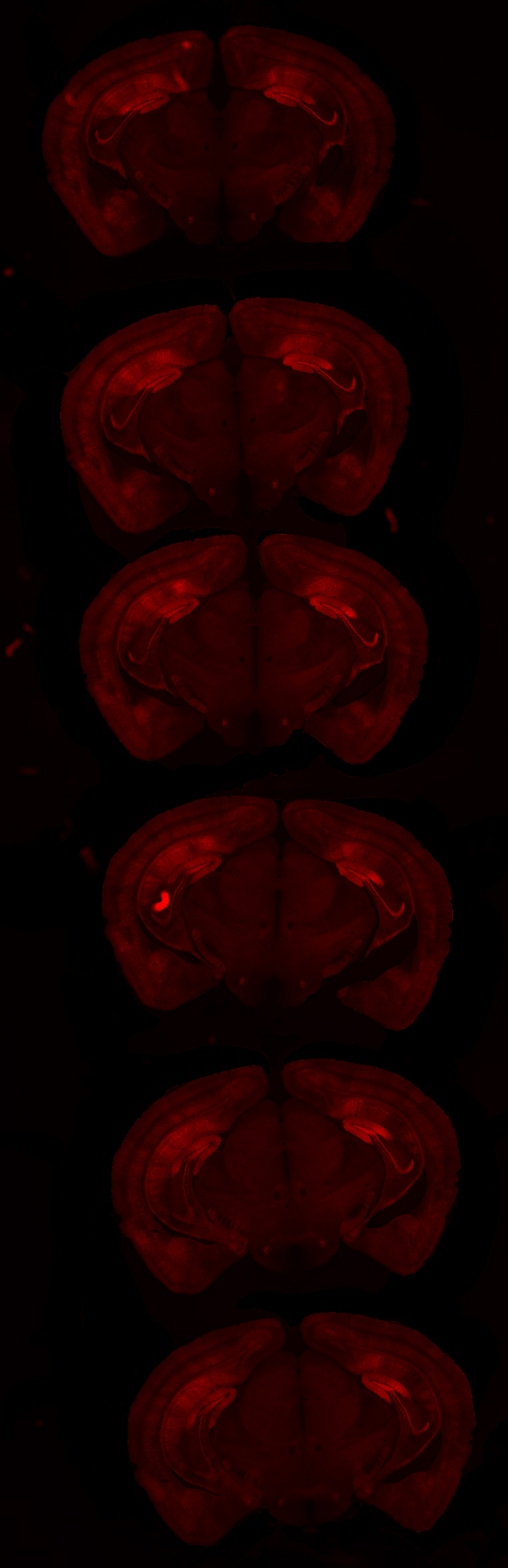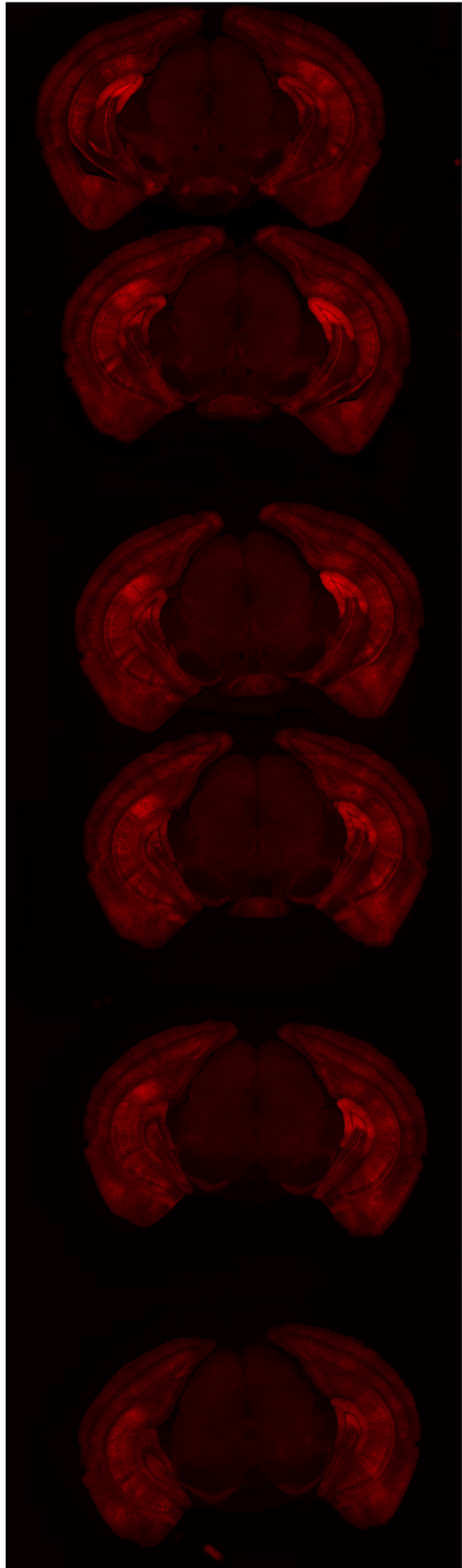

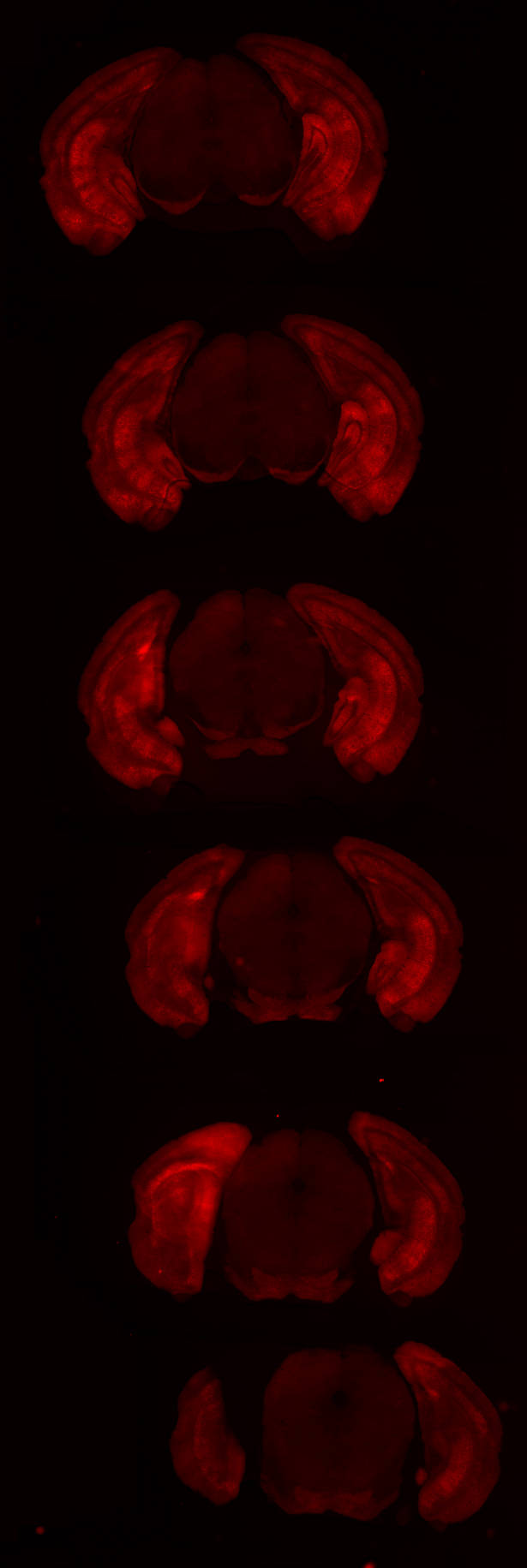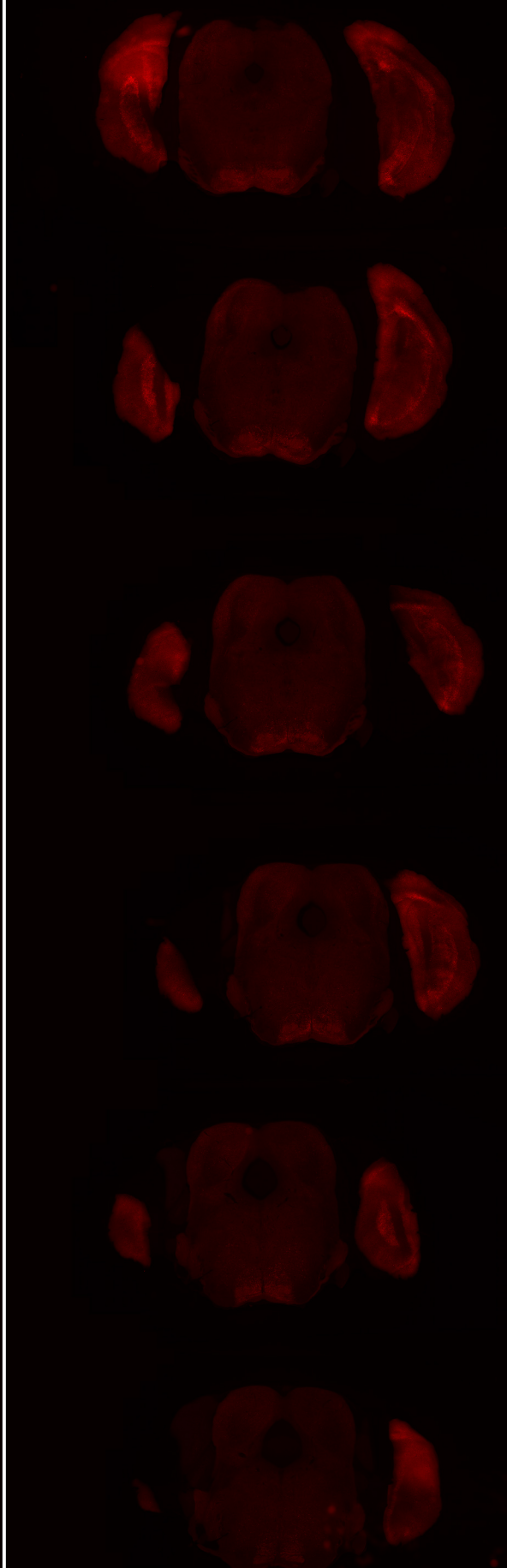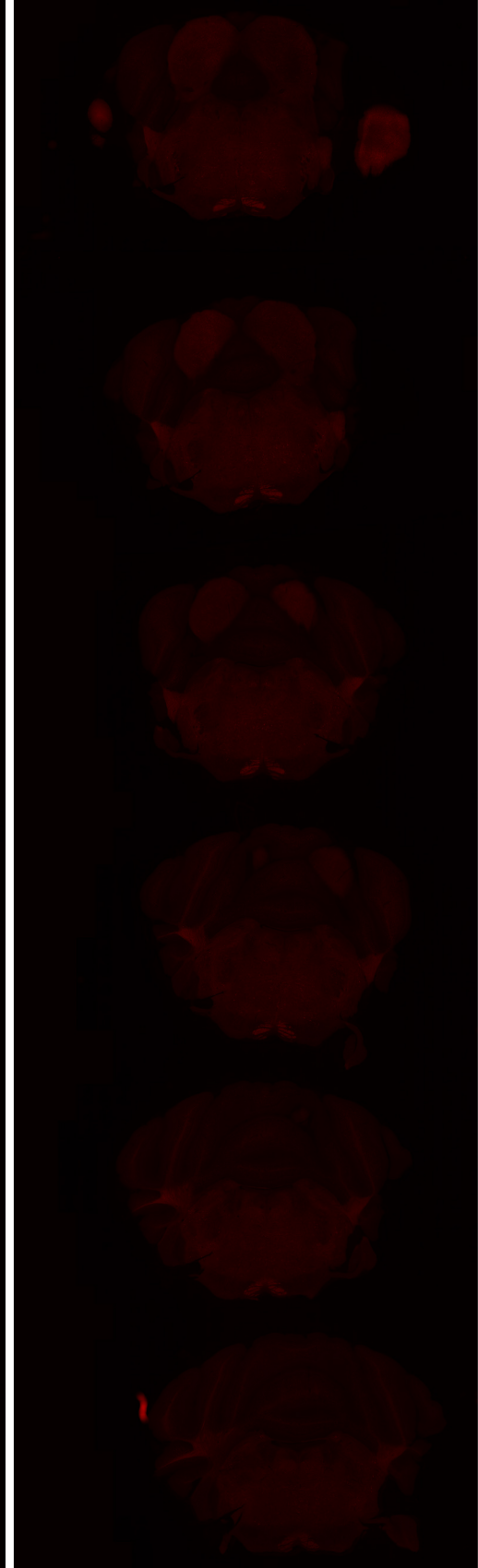

**GP 8.50**

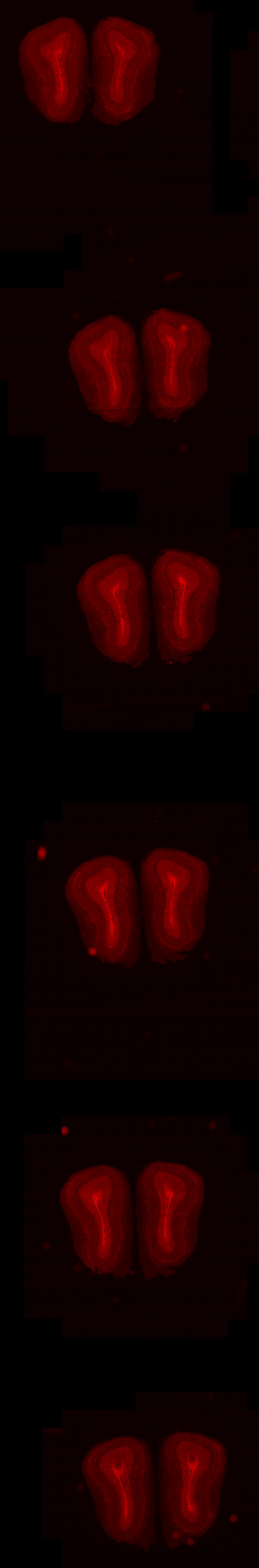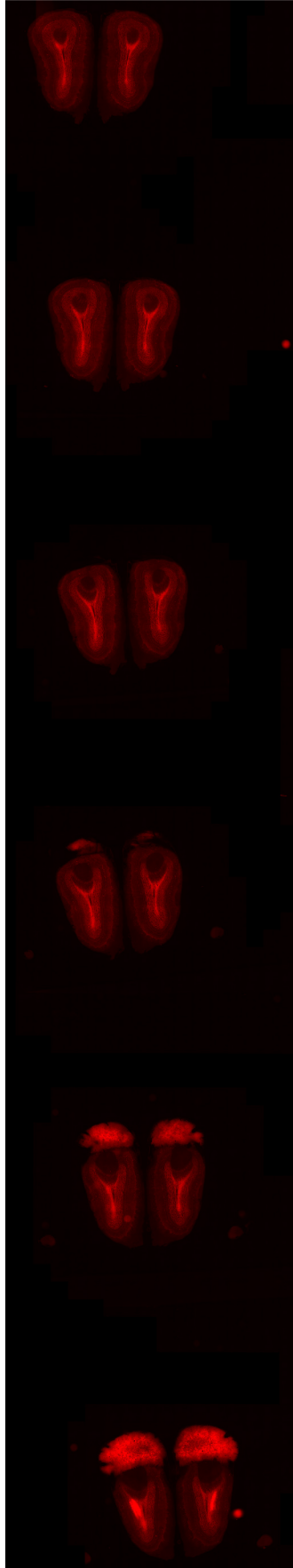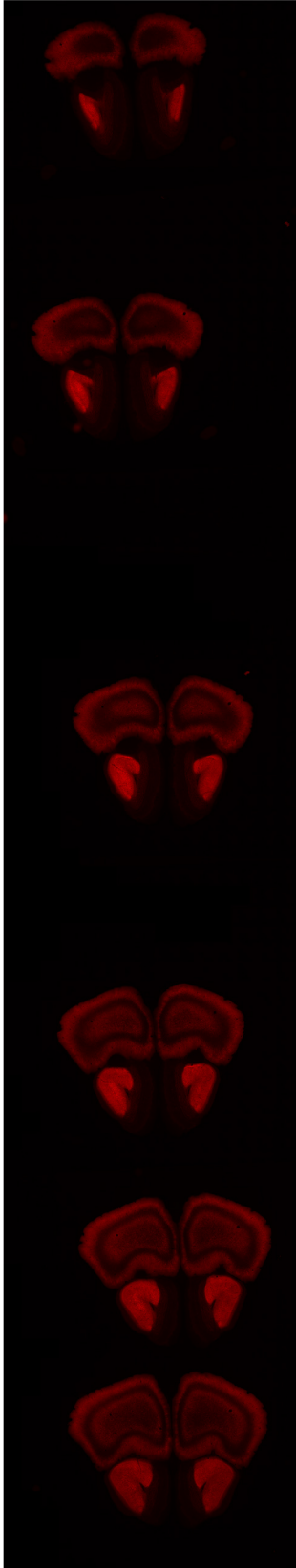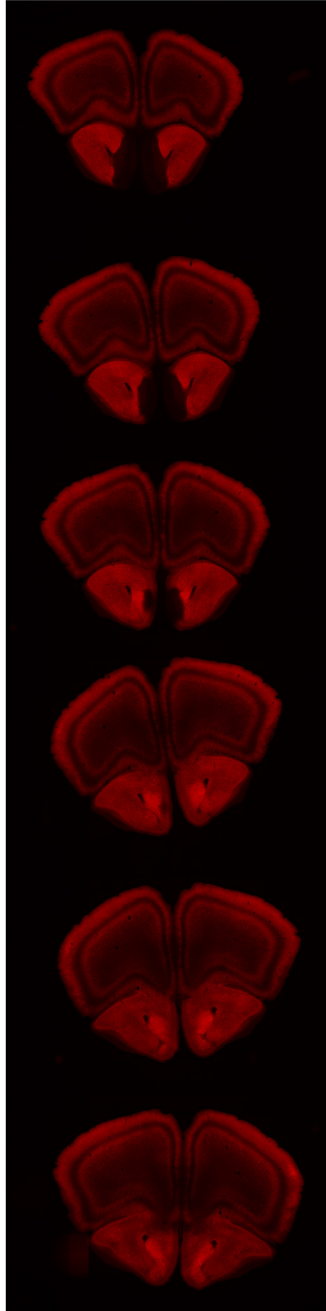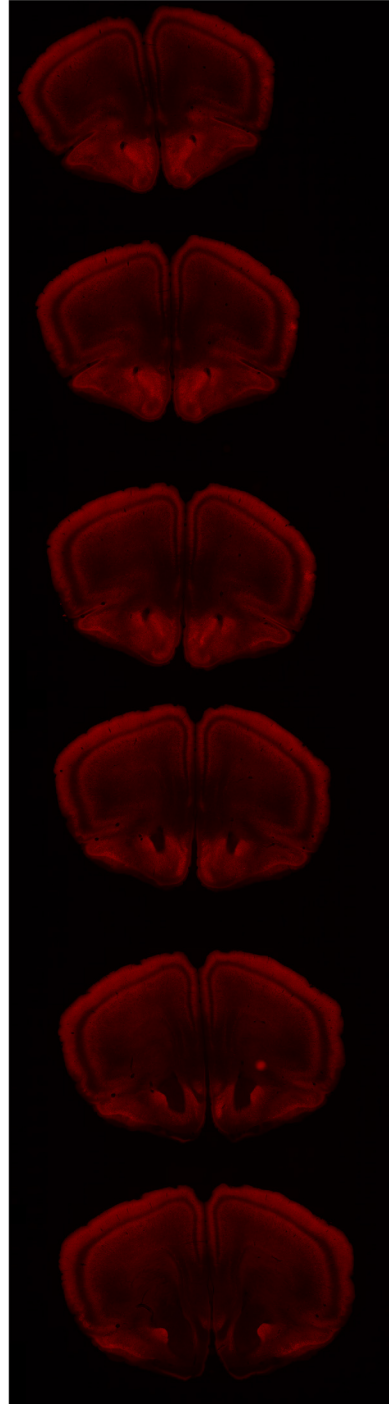

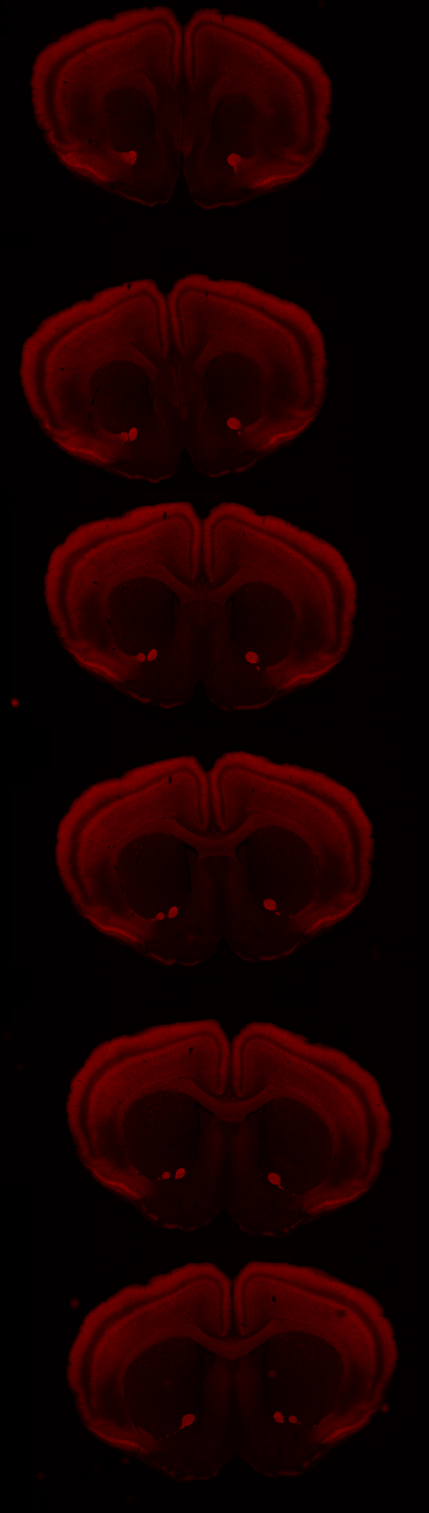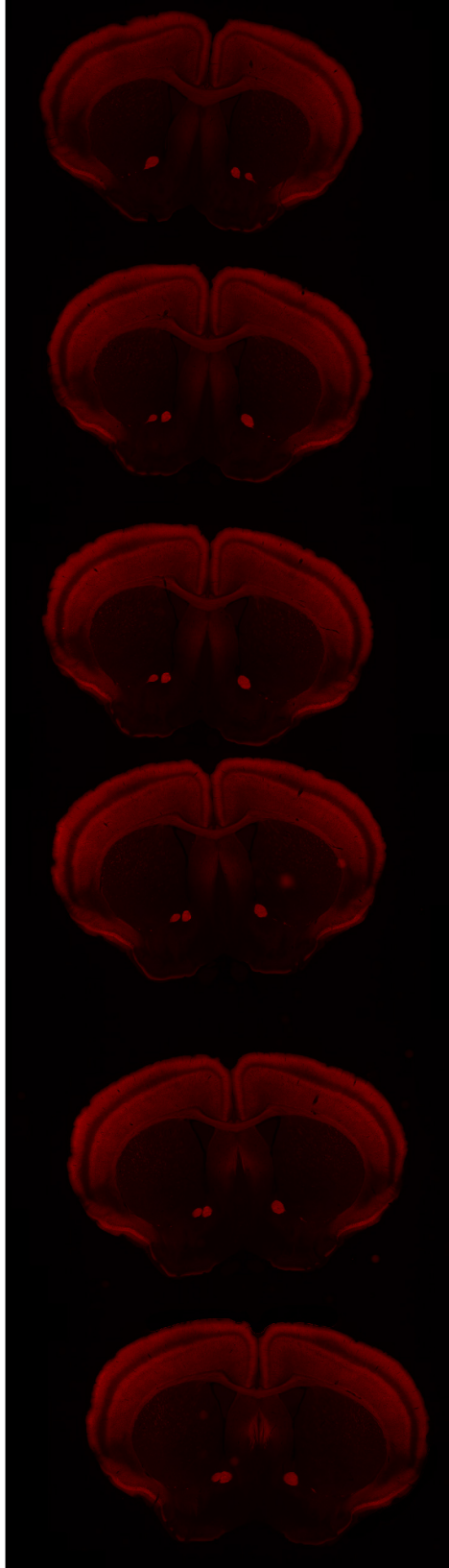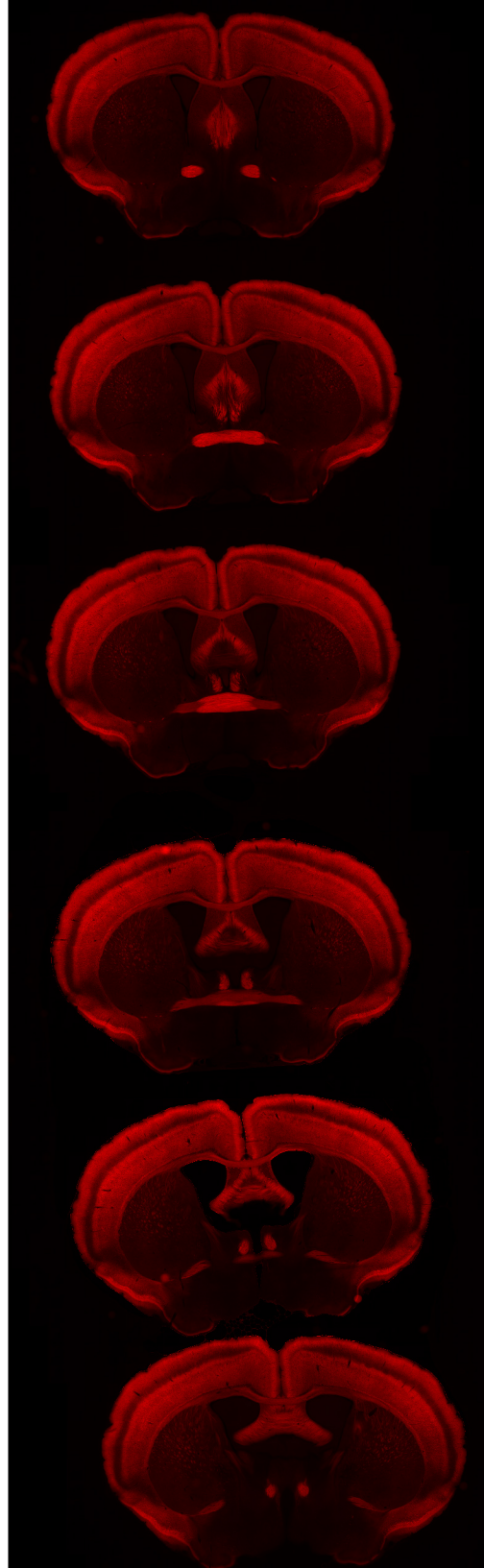

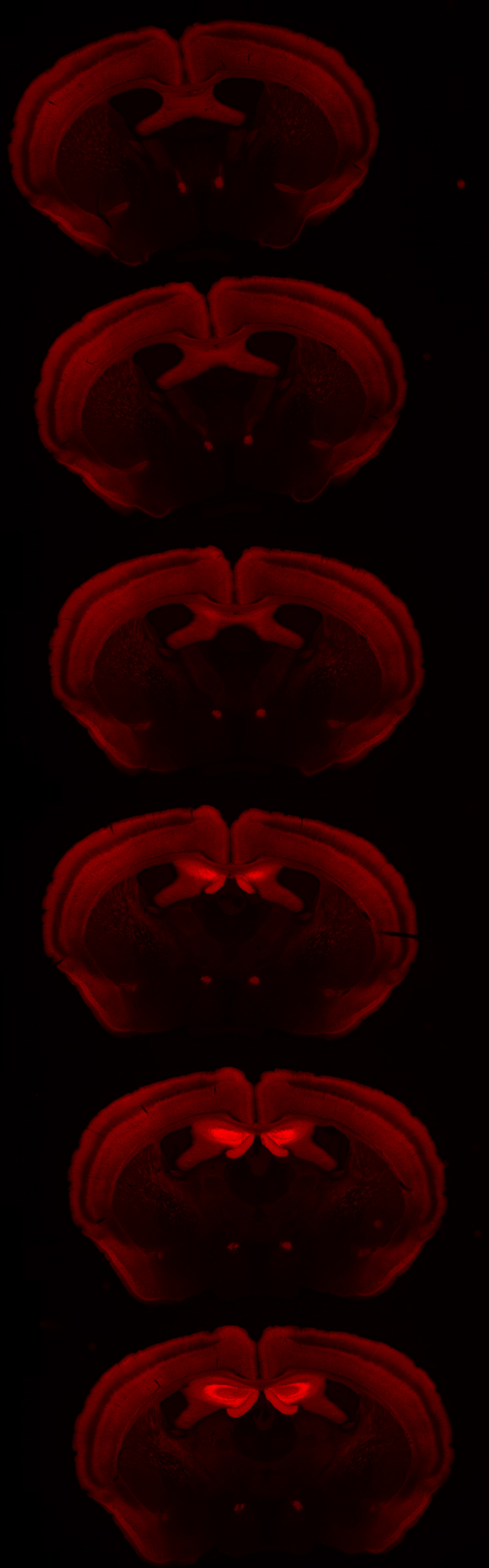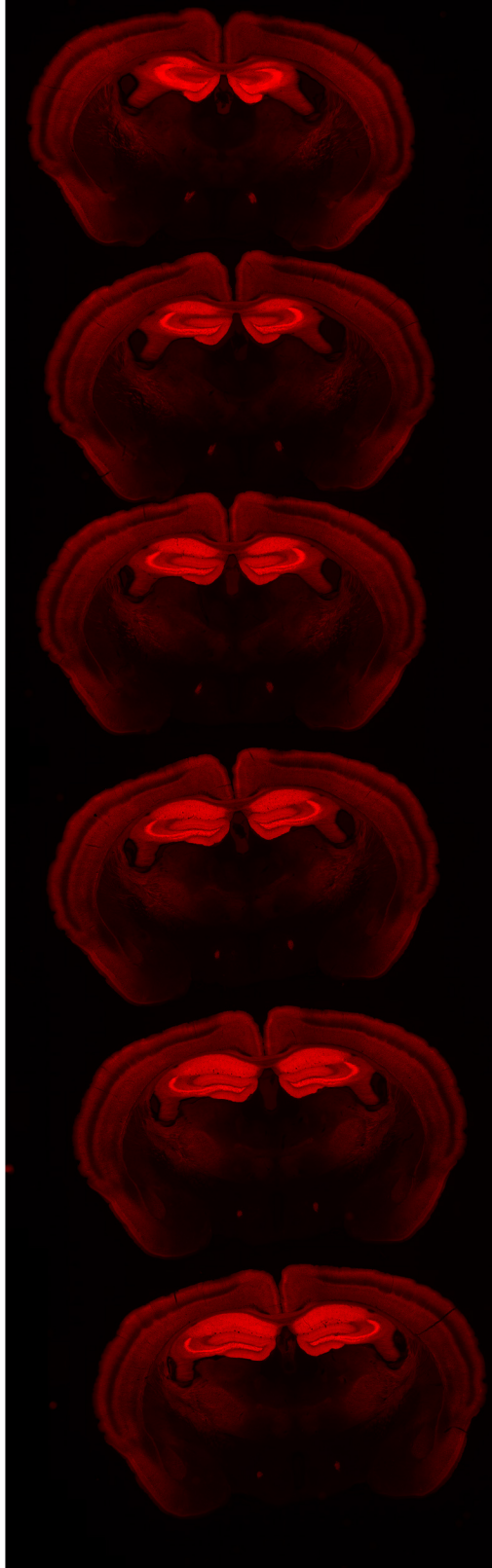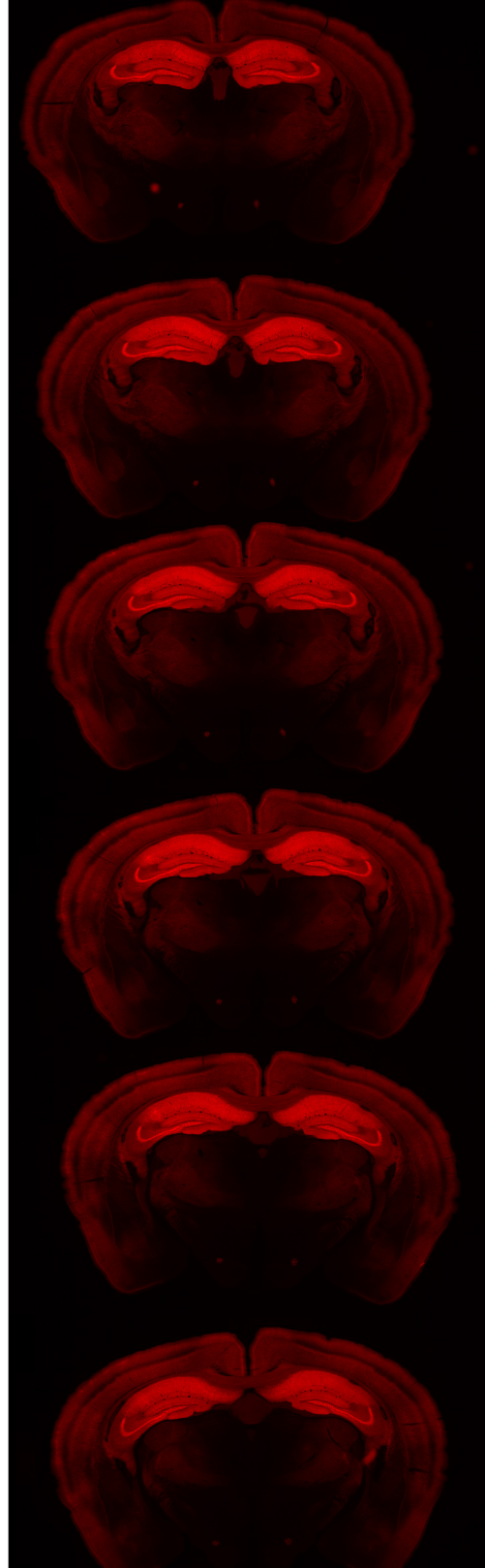

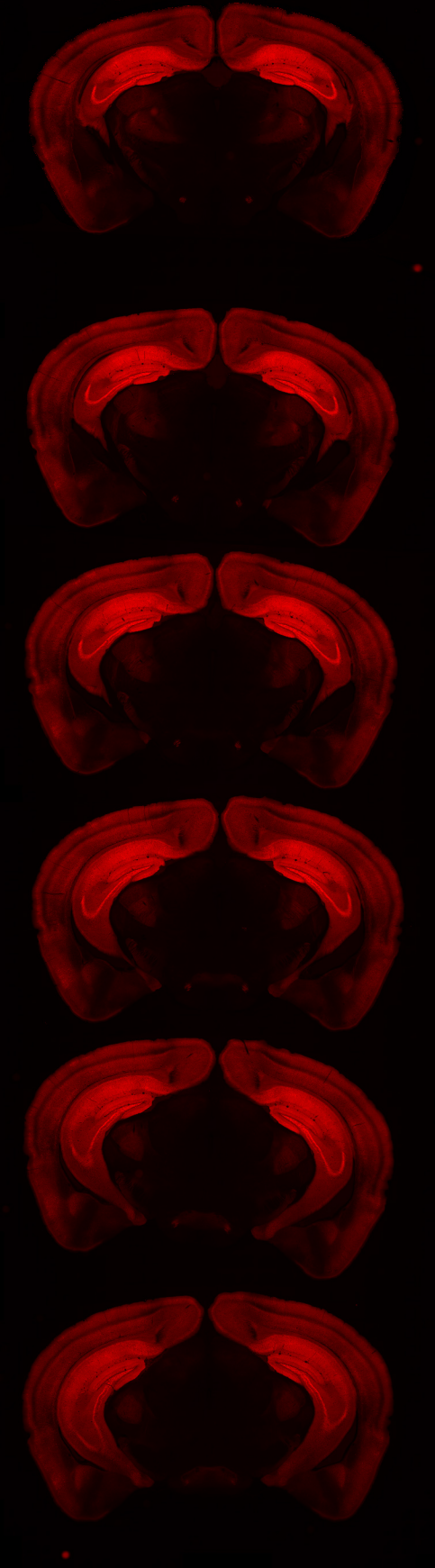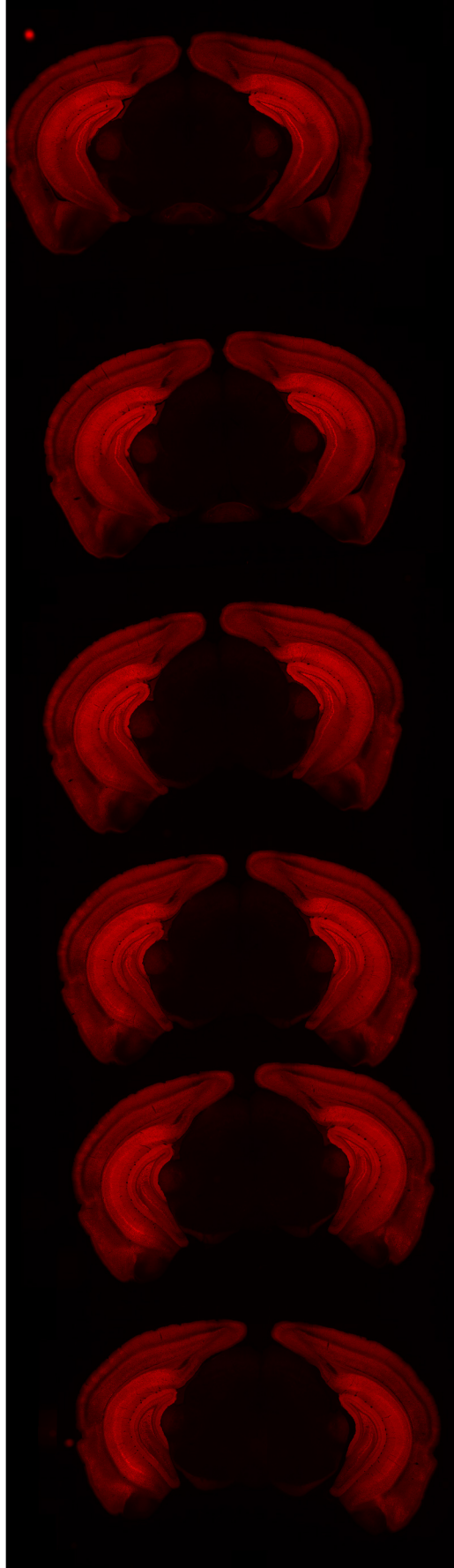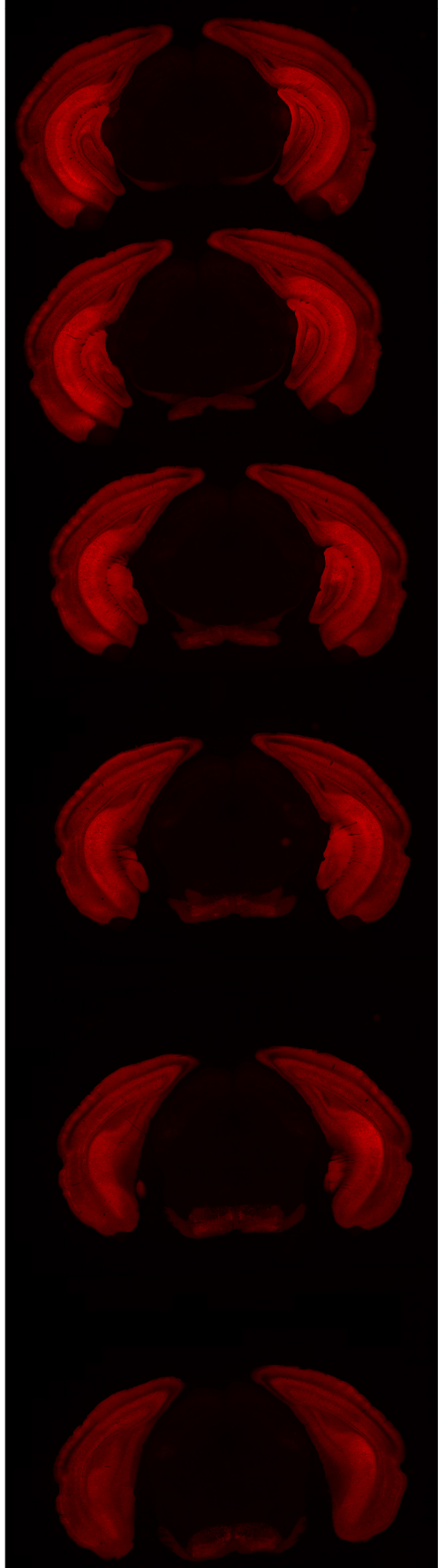

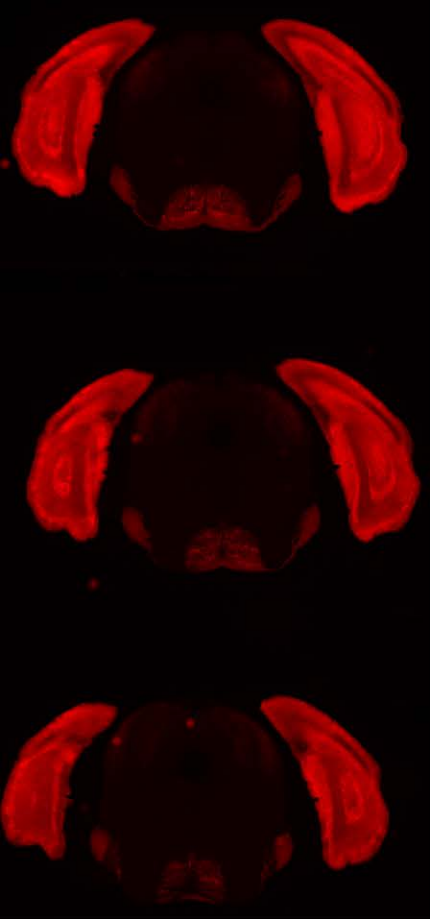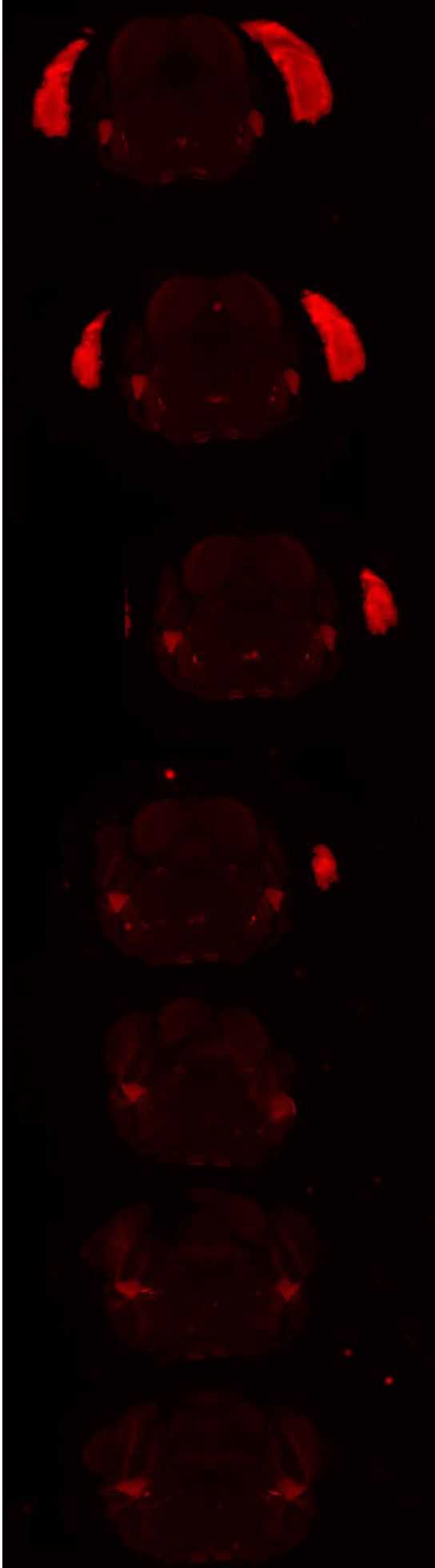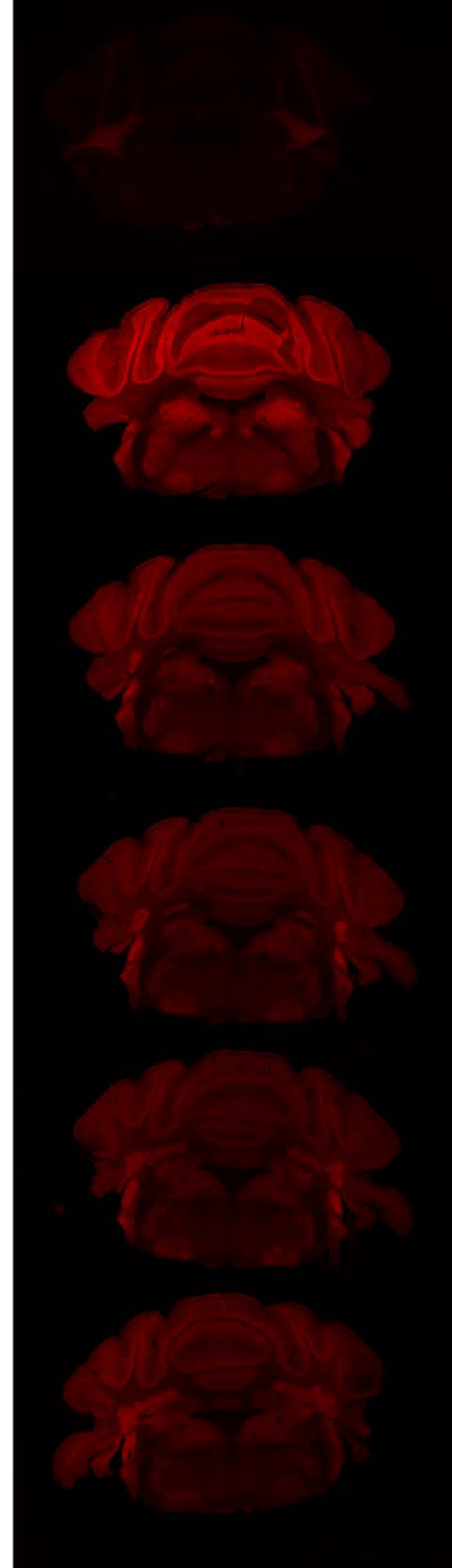

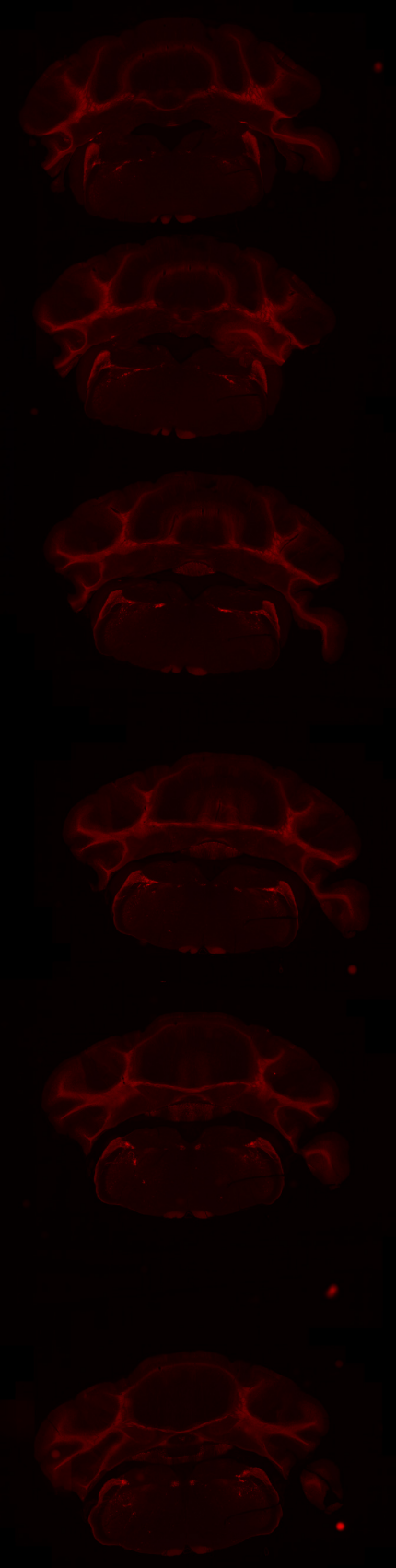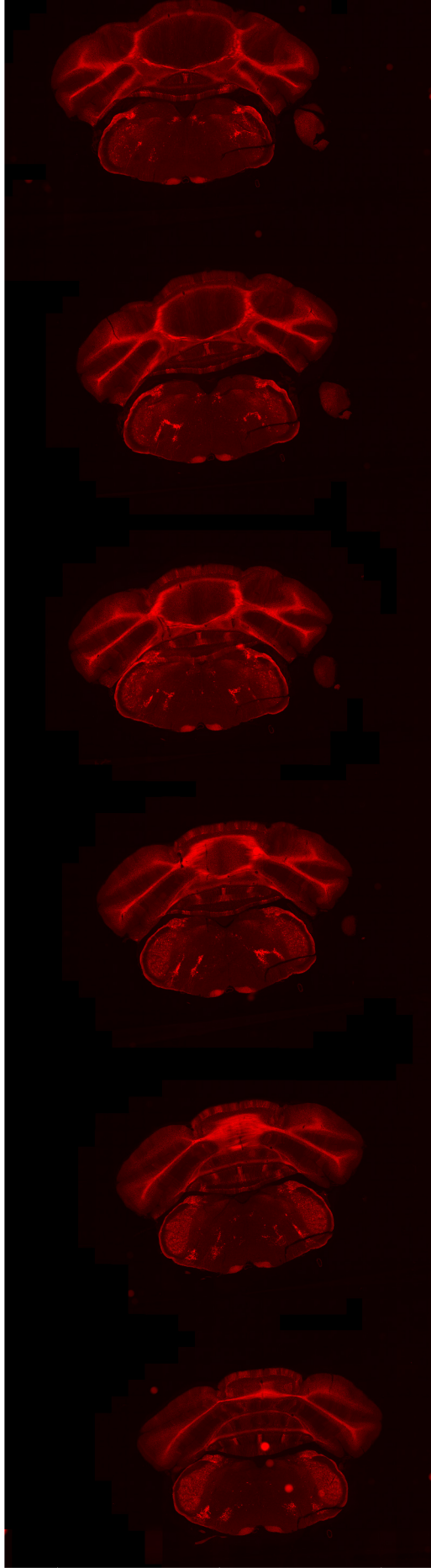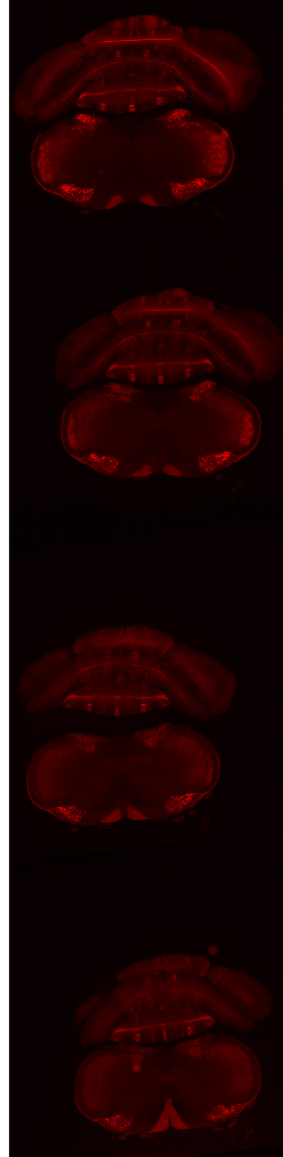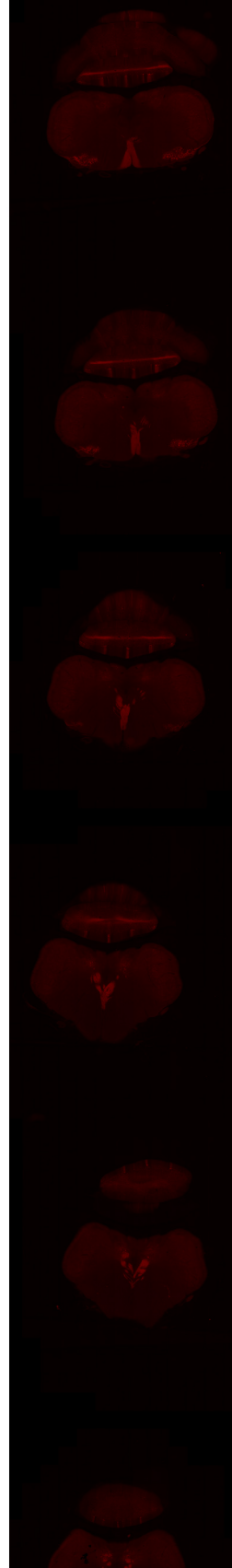

**GP 8.52**

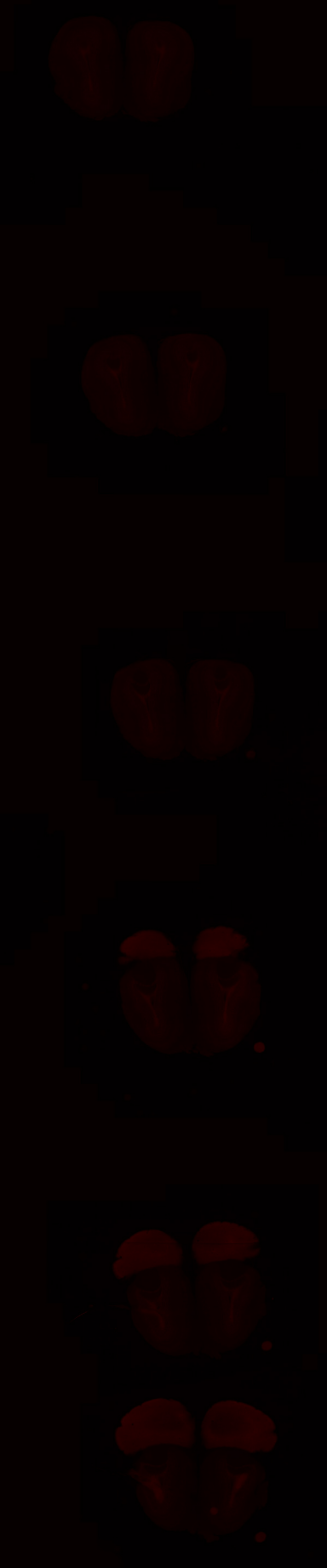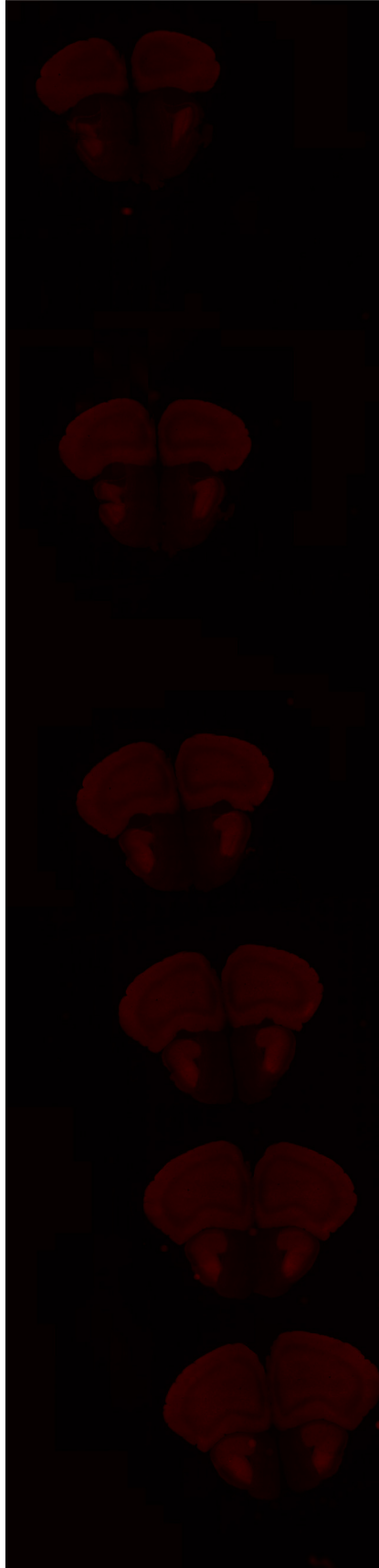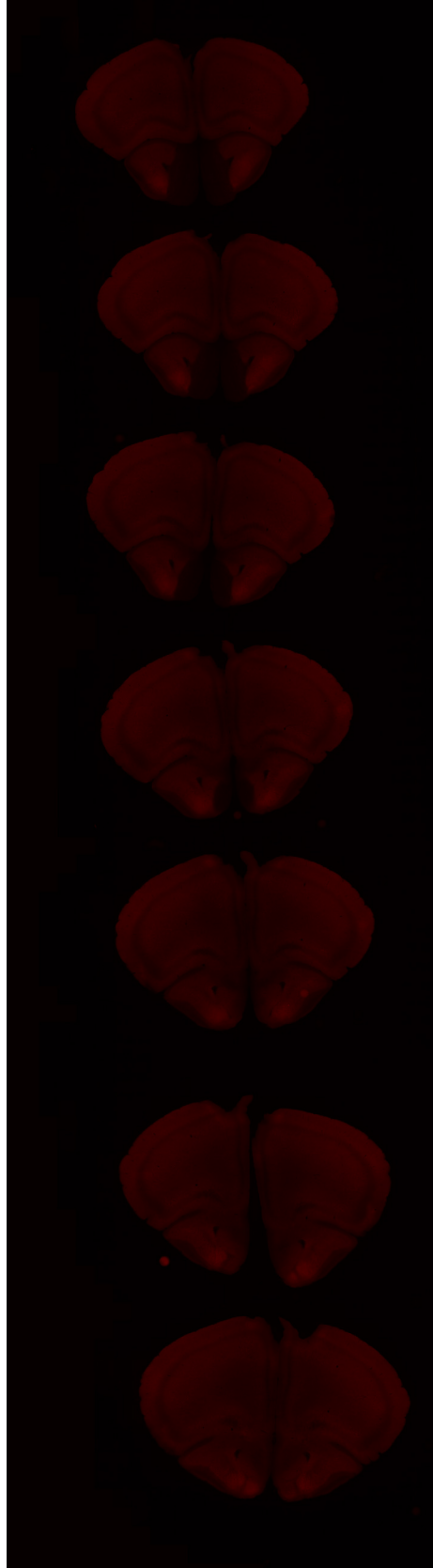

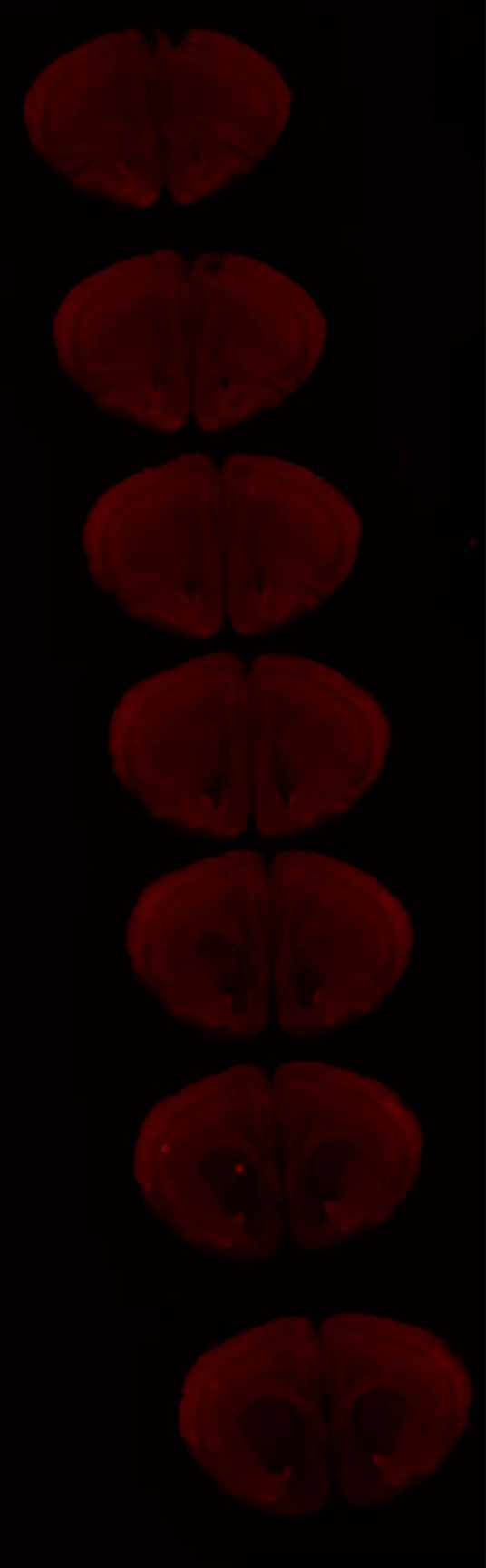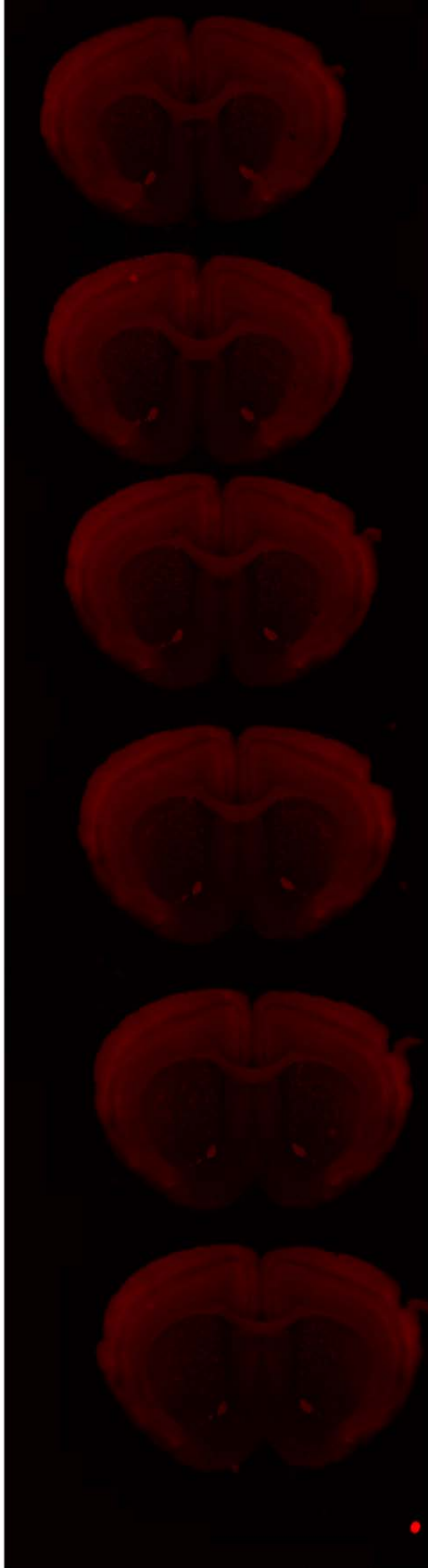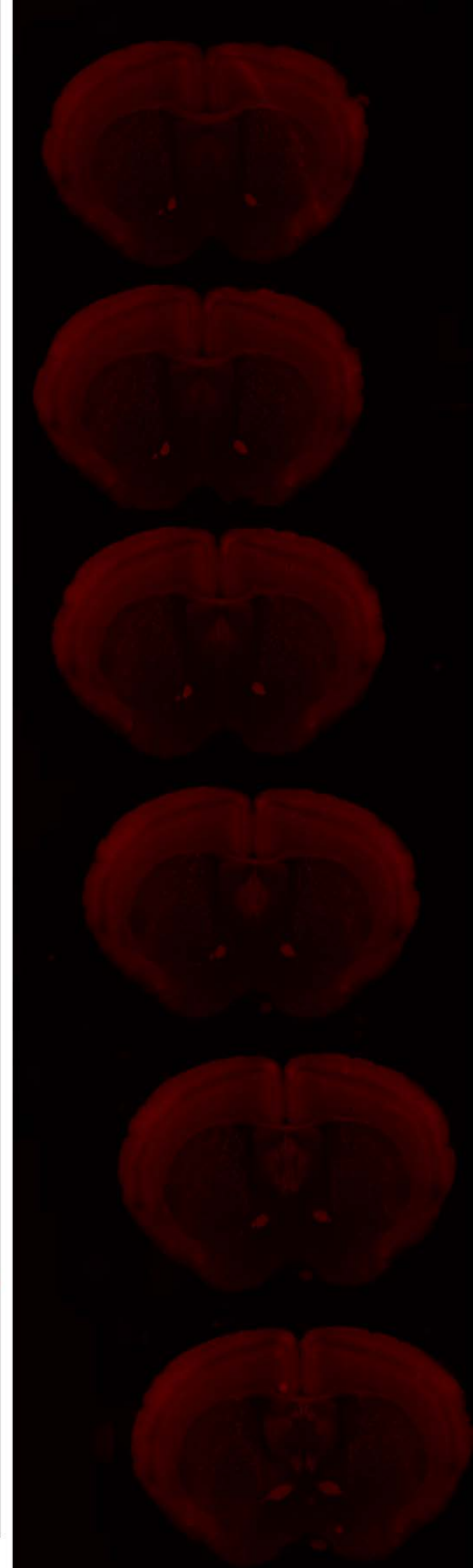

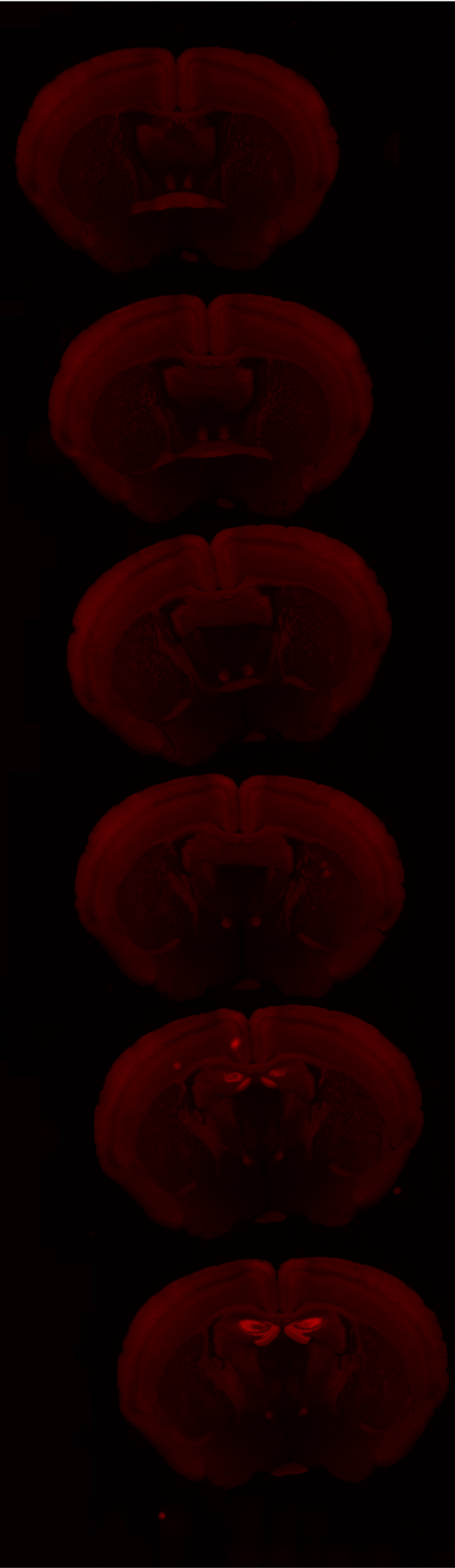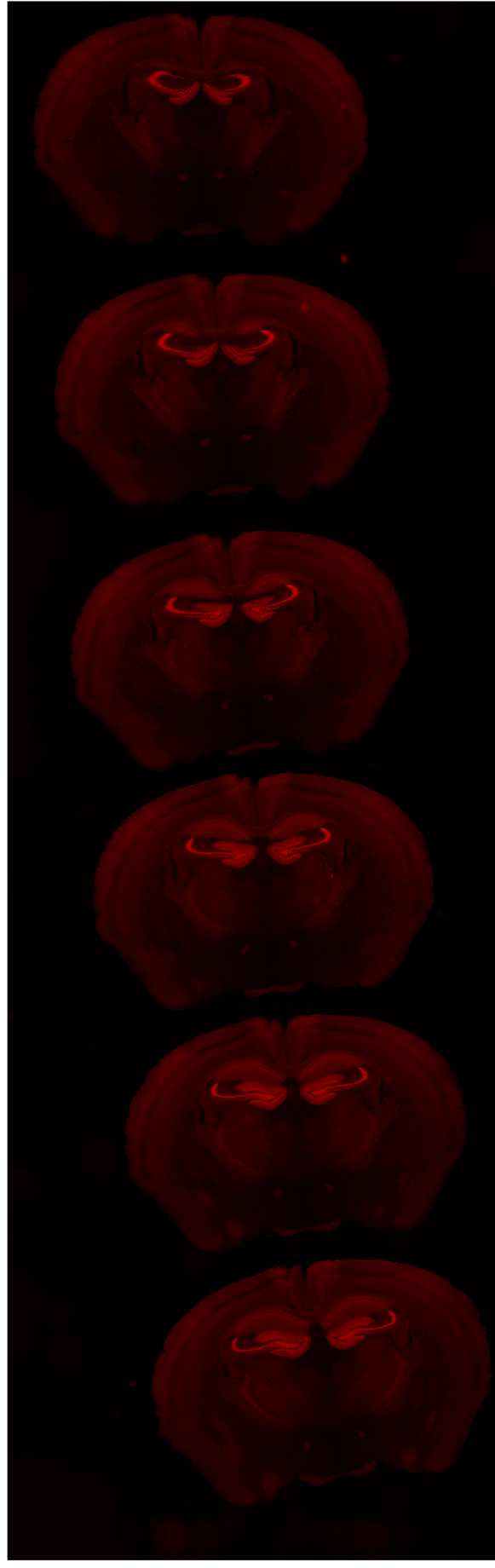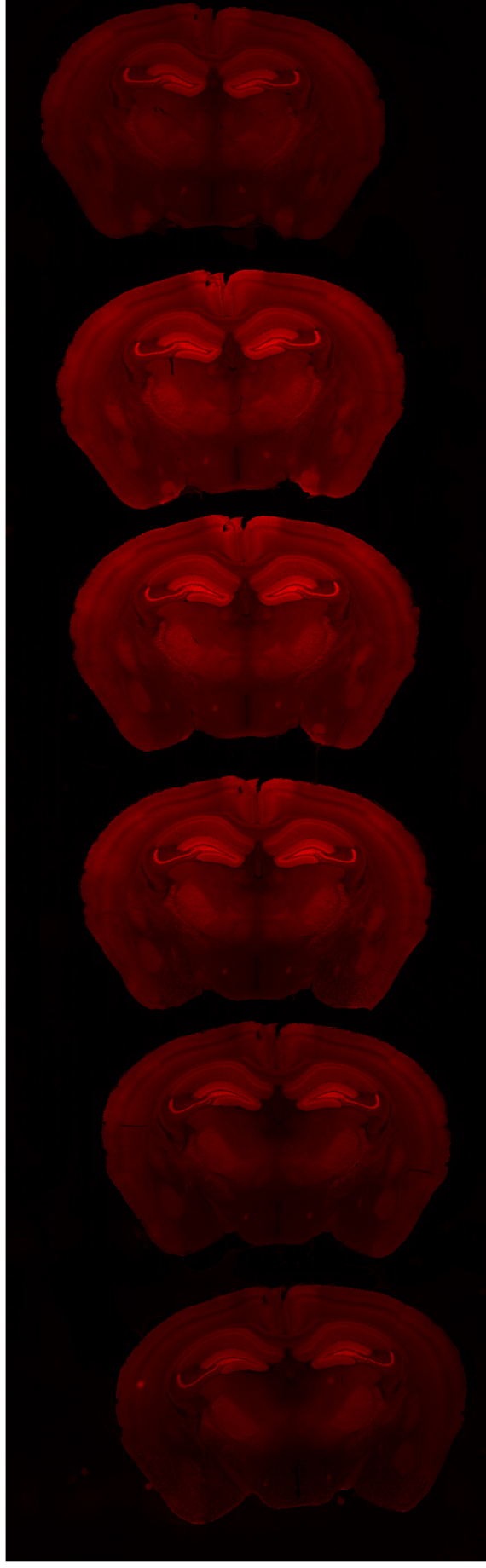

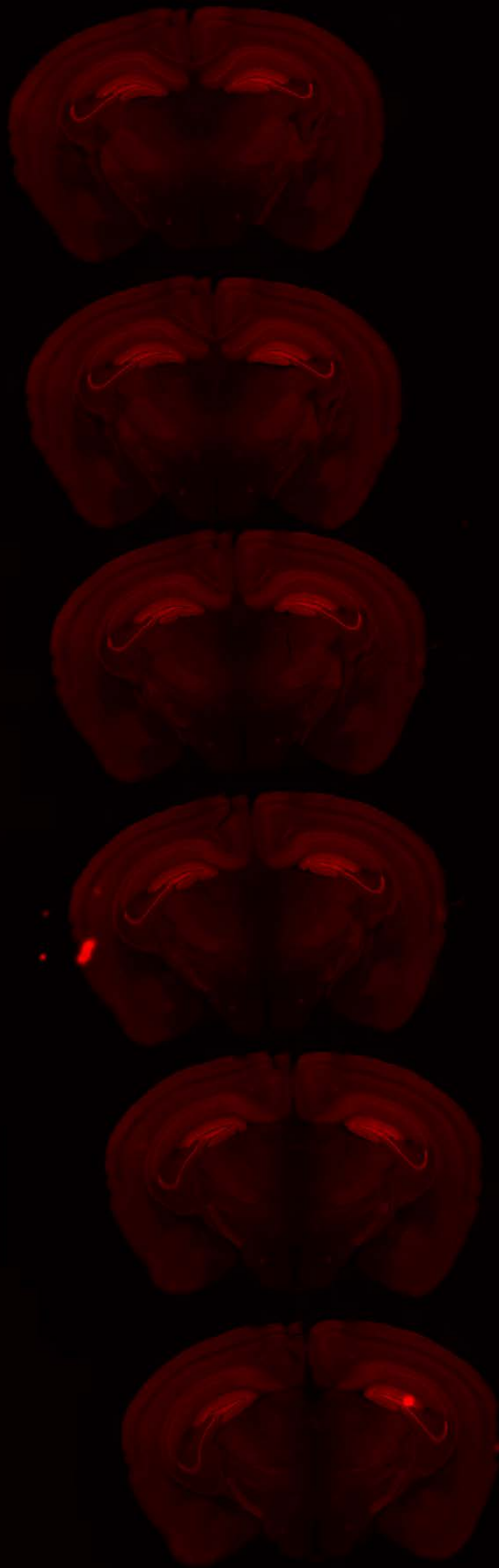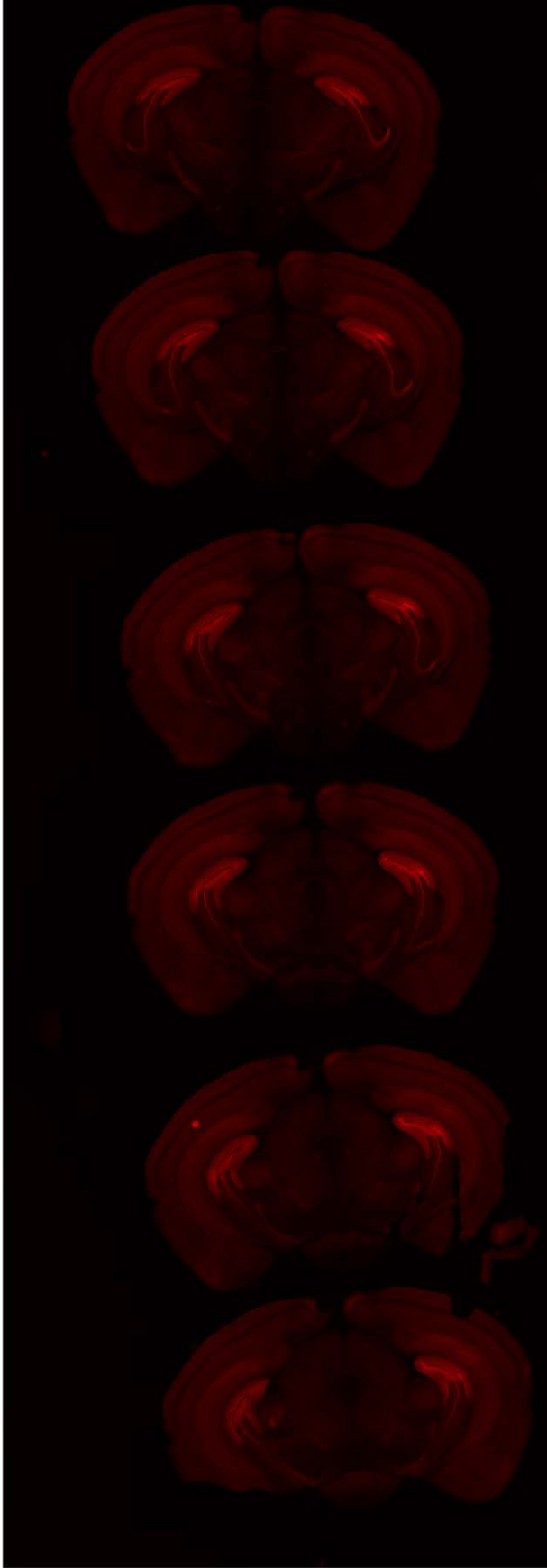

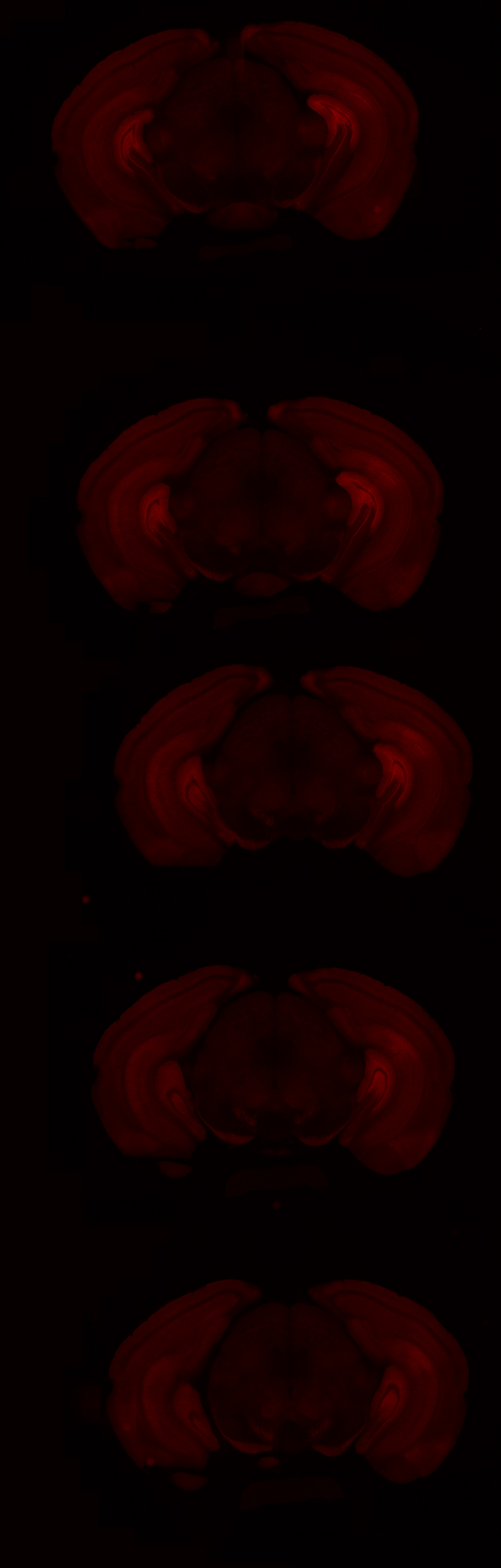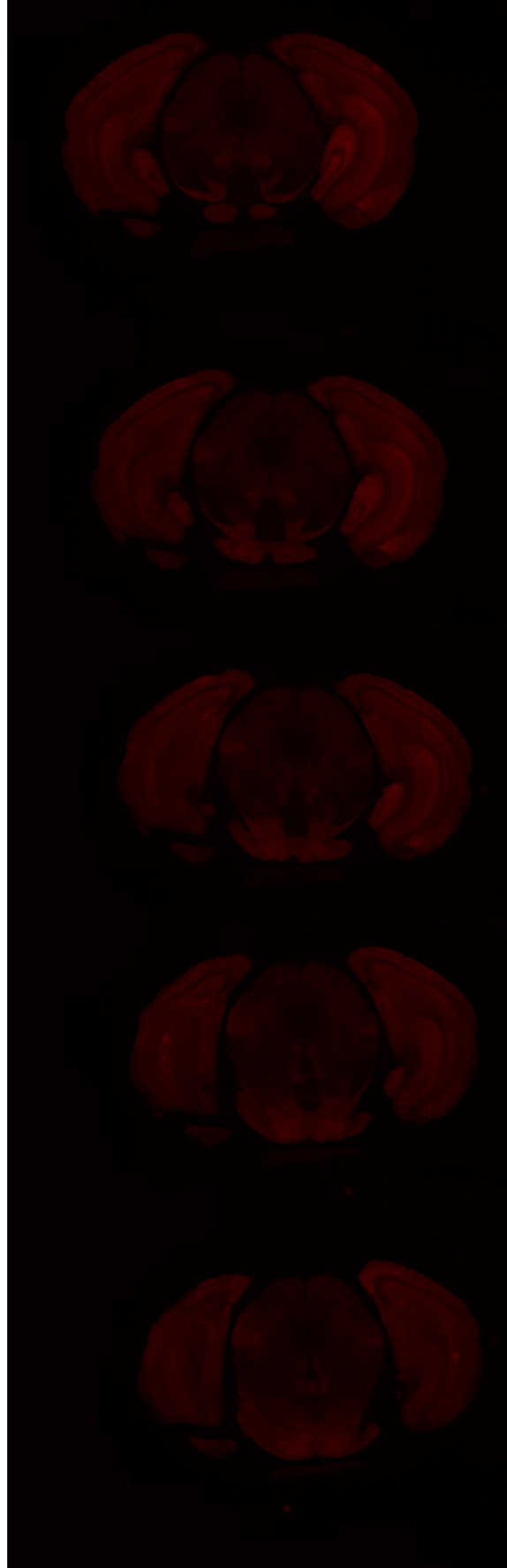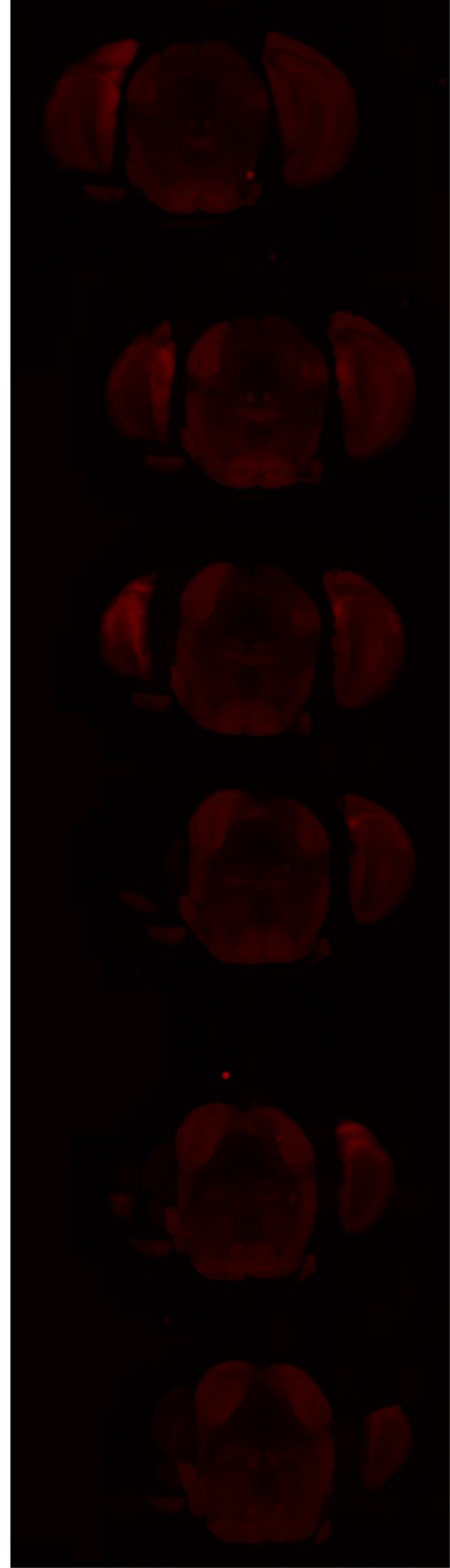

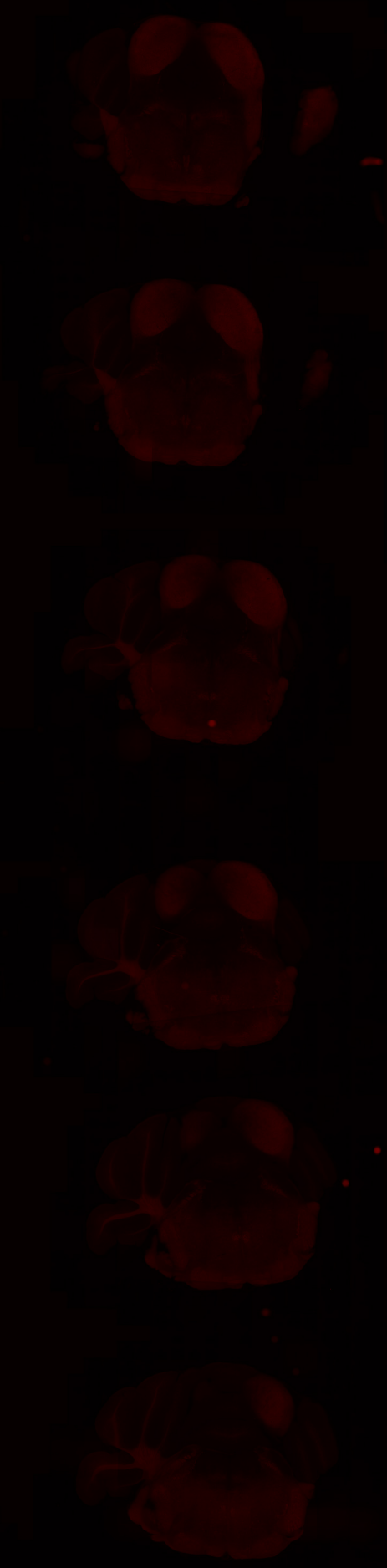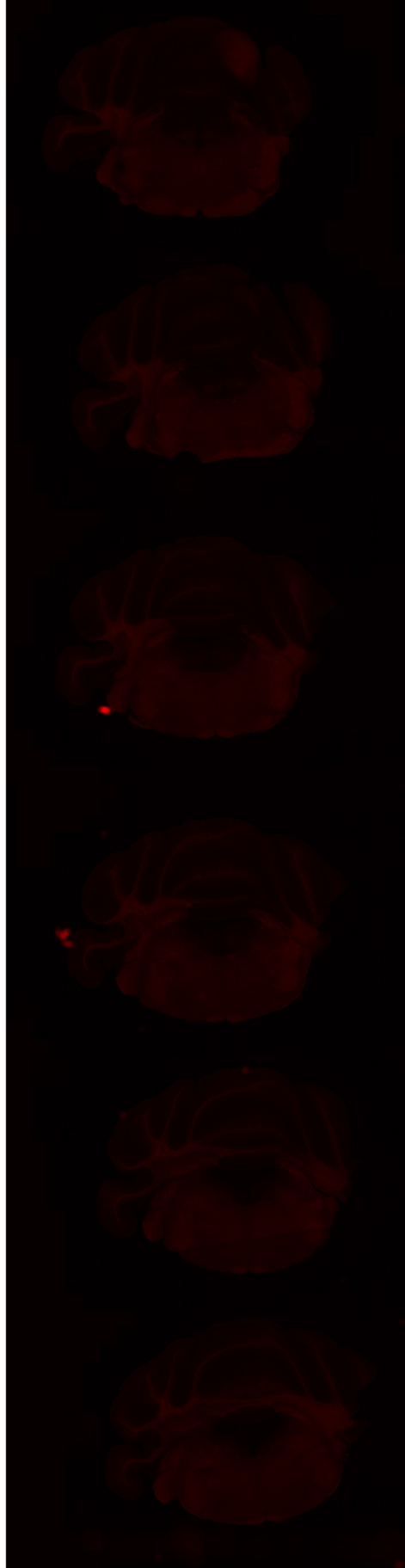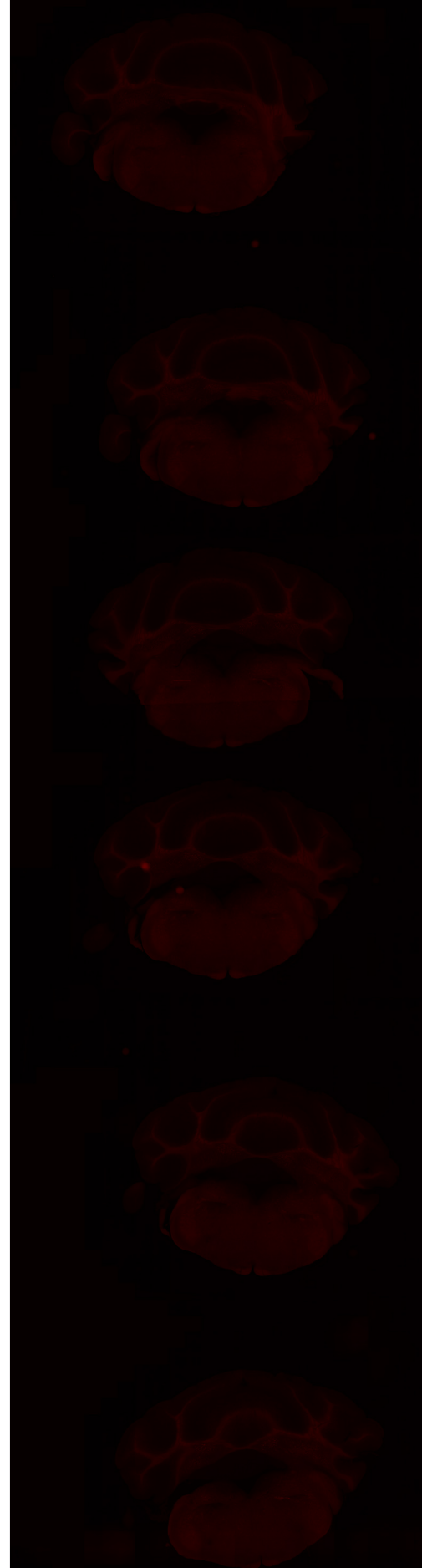

**GP 8.58**

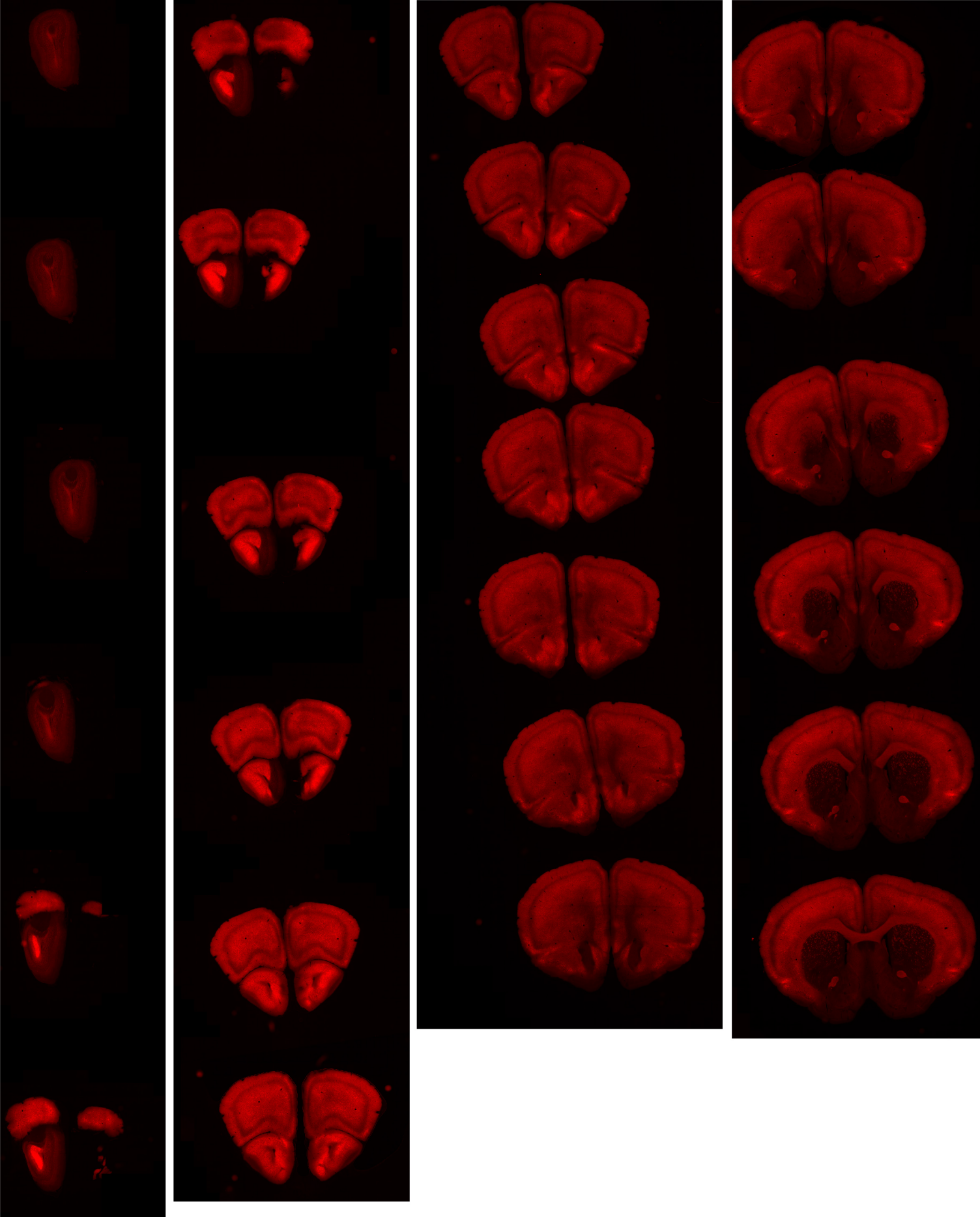

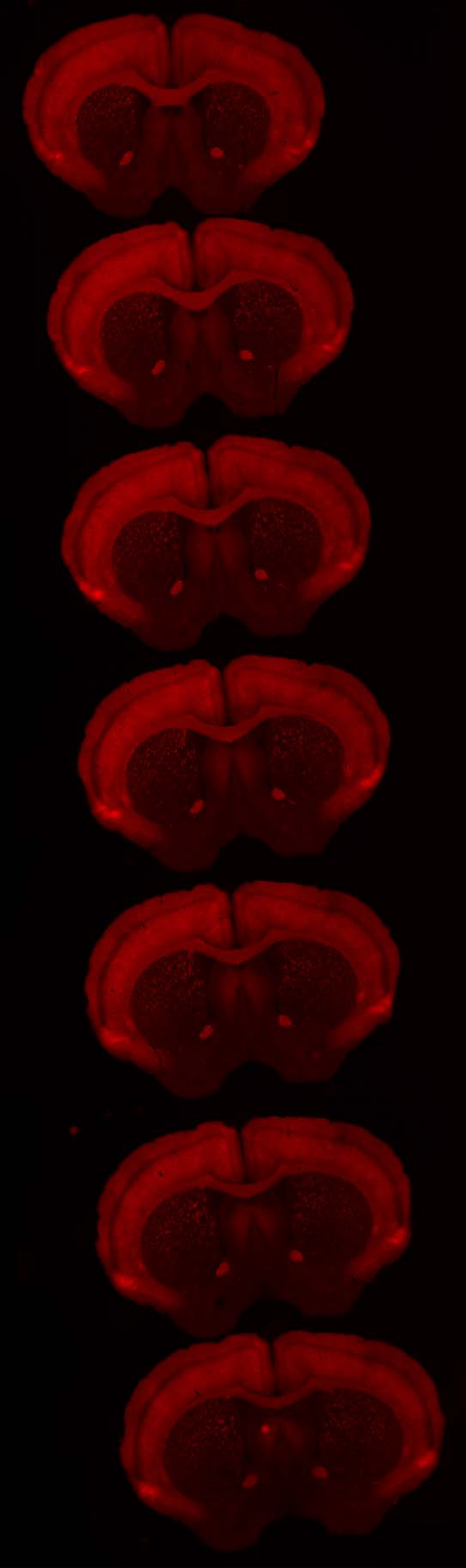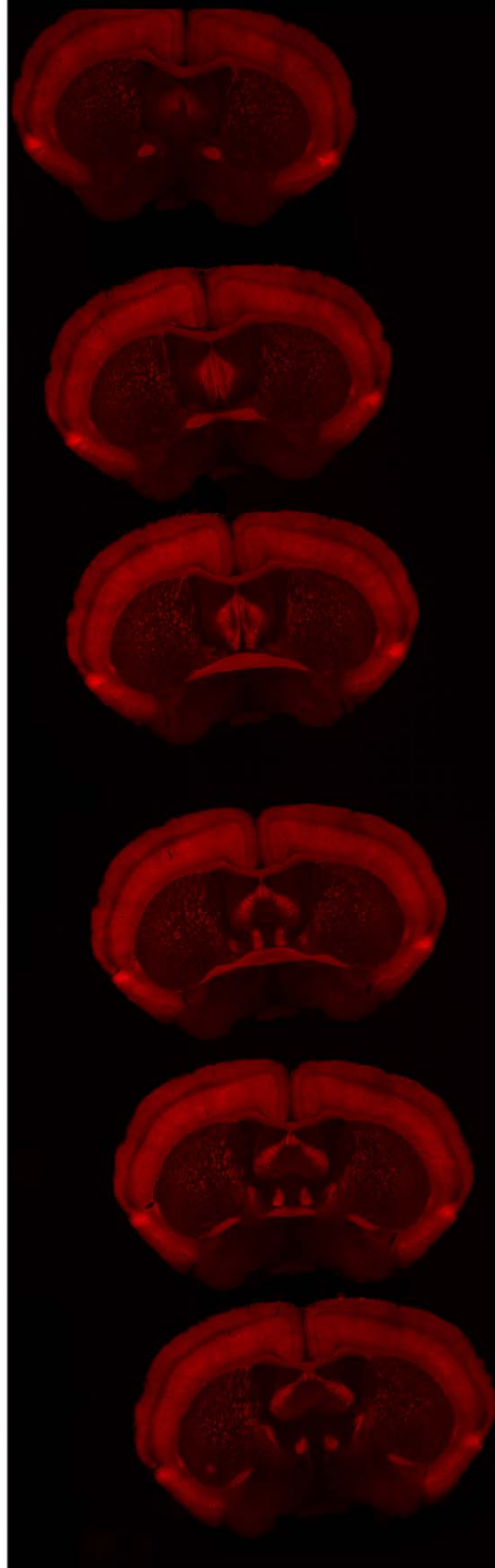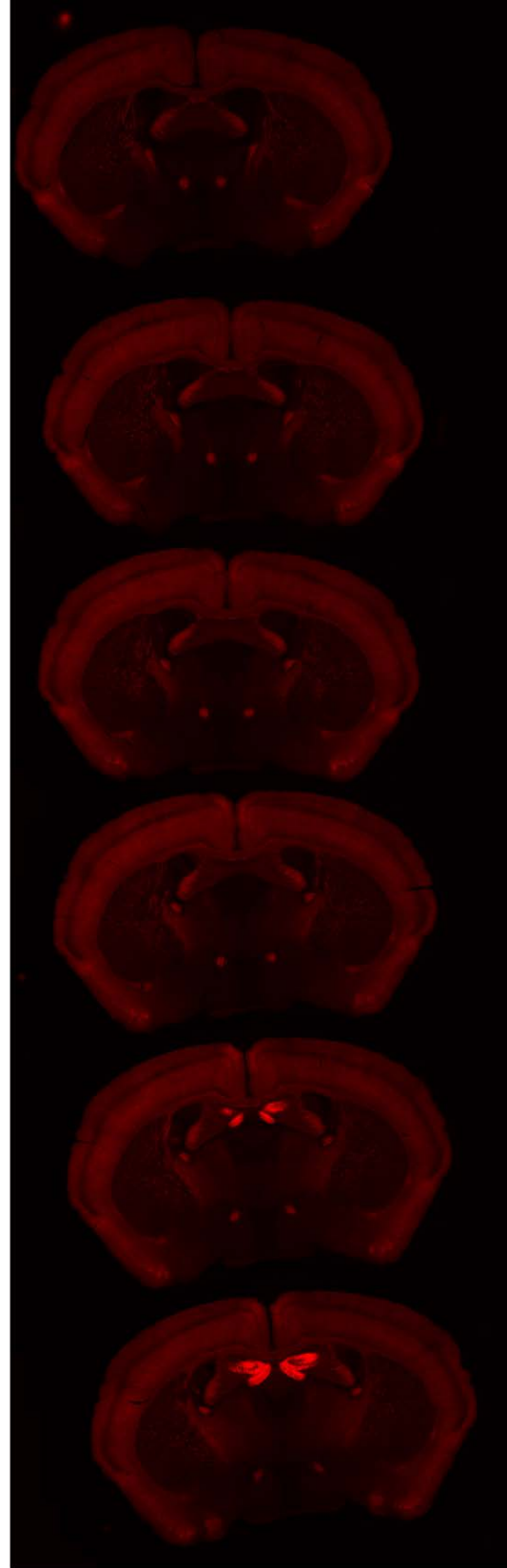

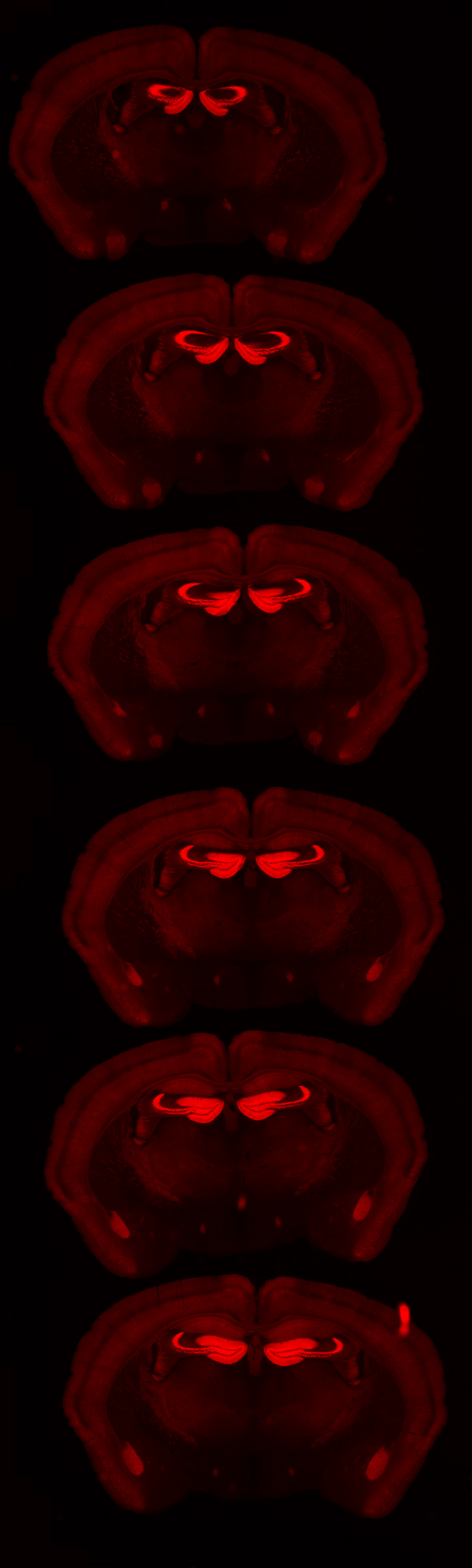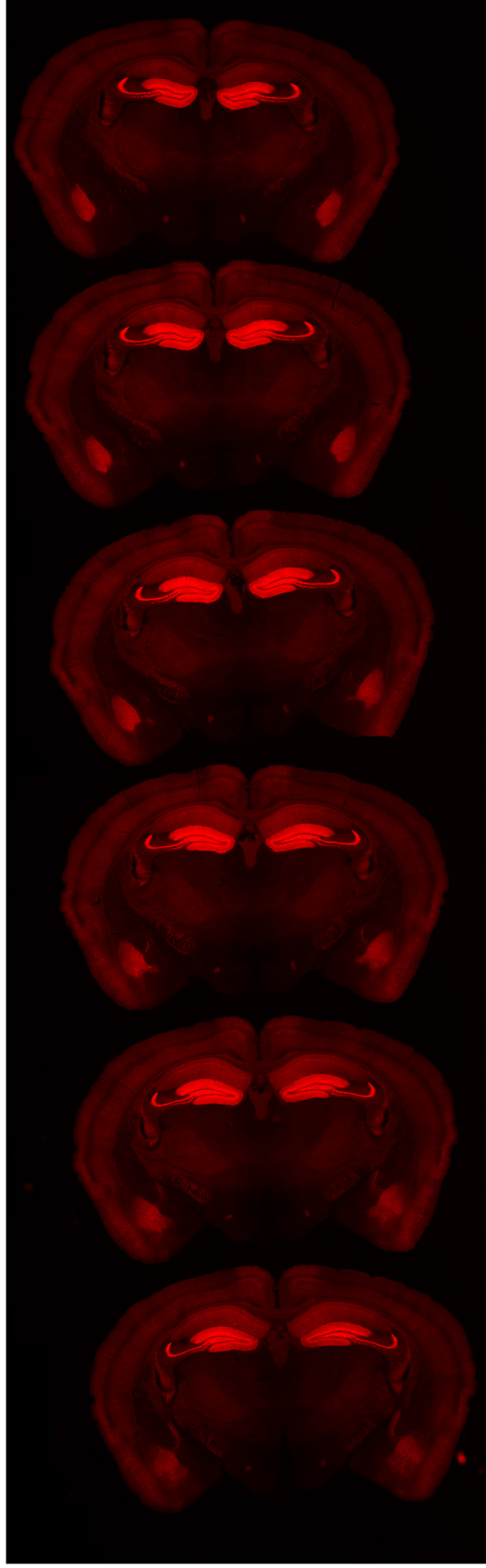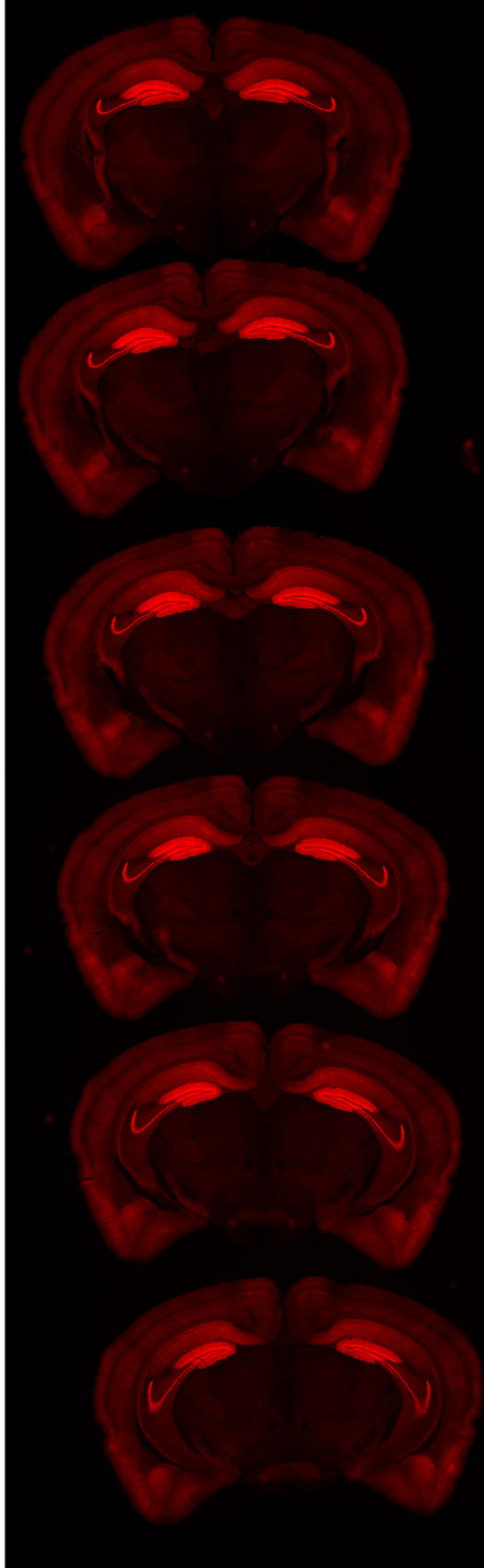

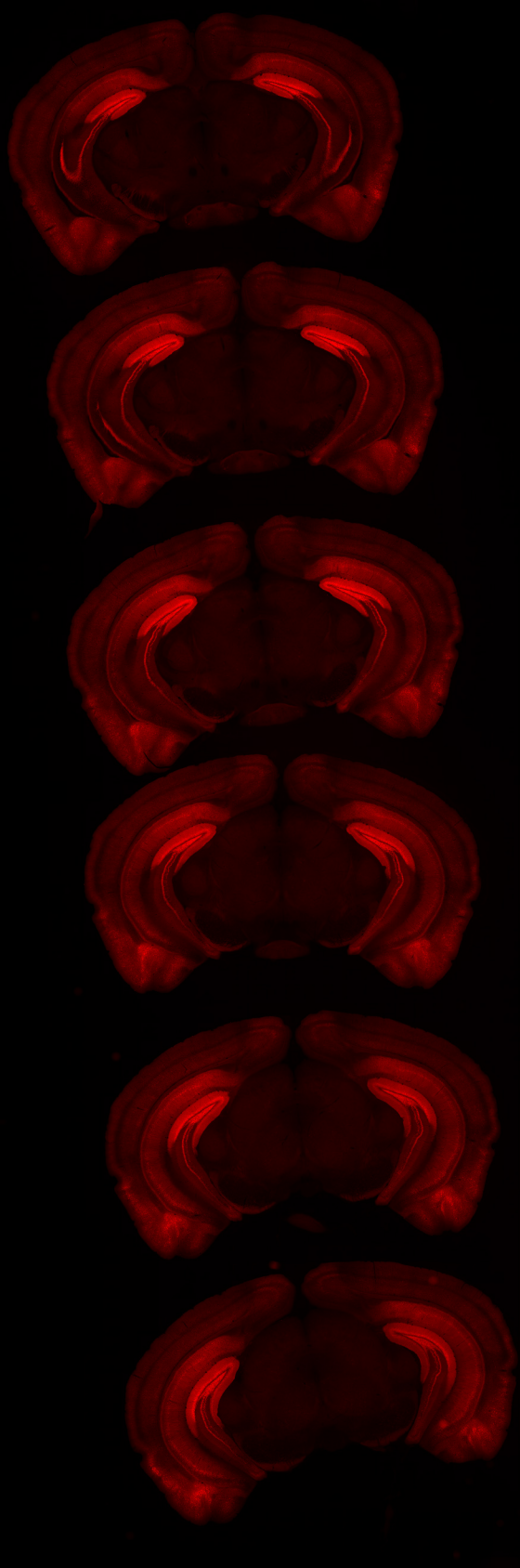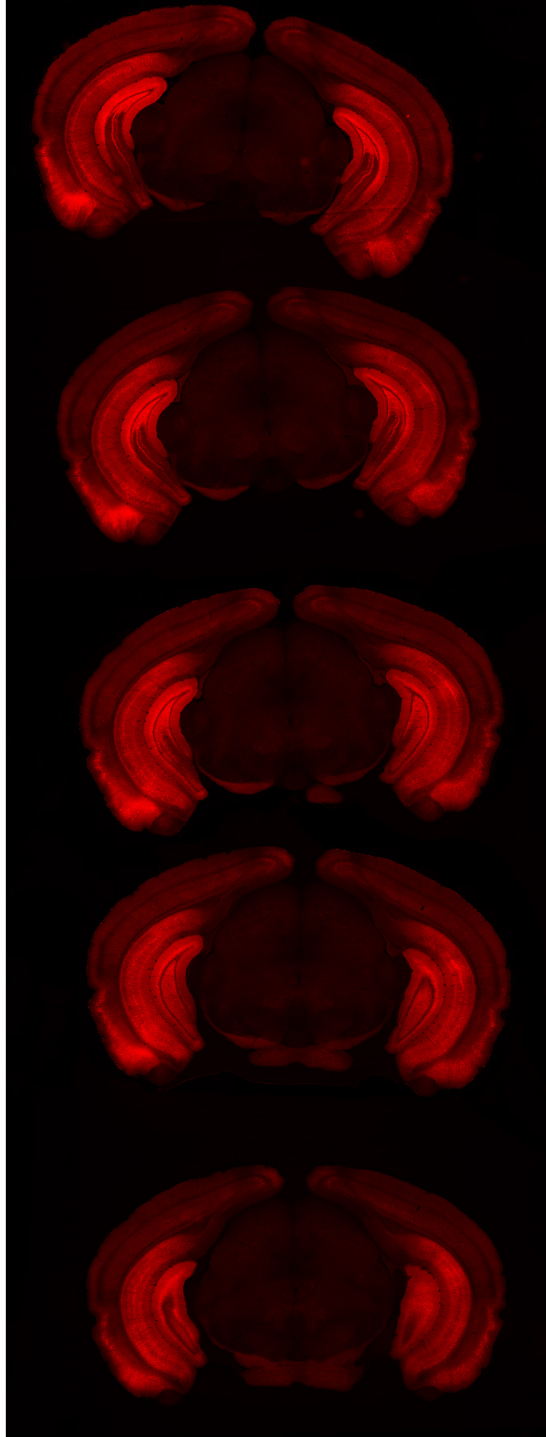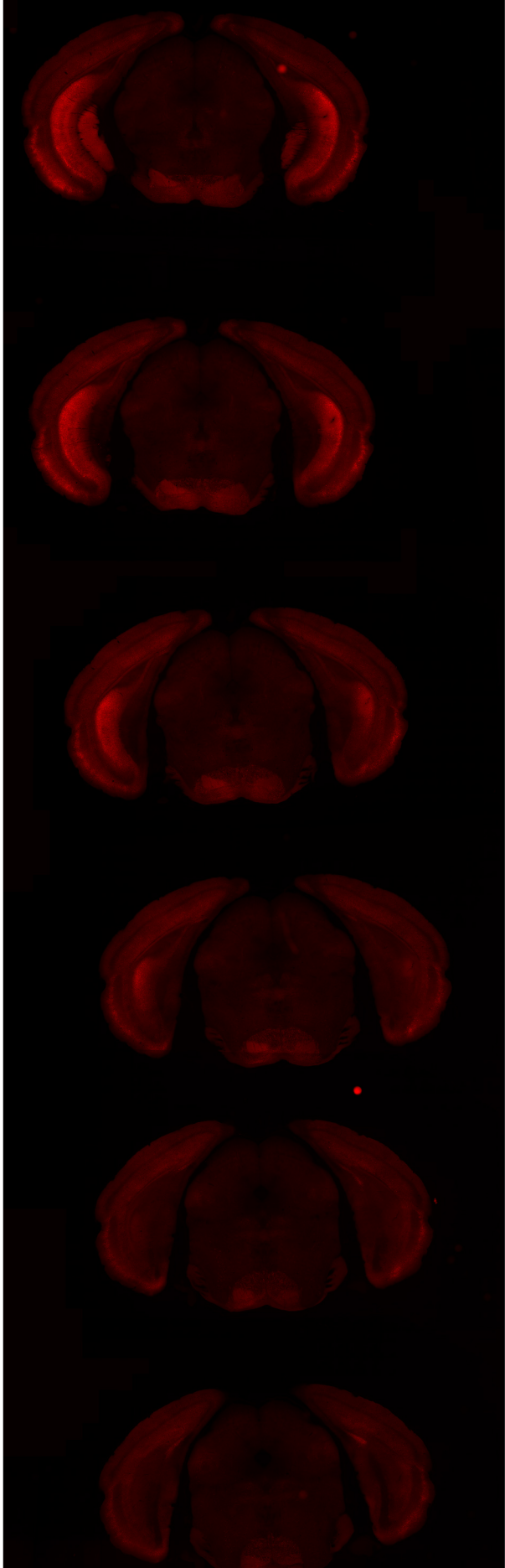

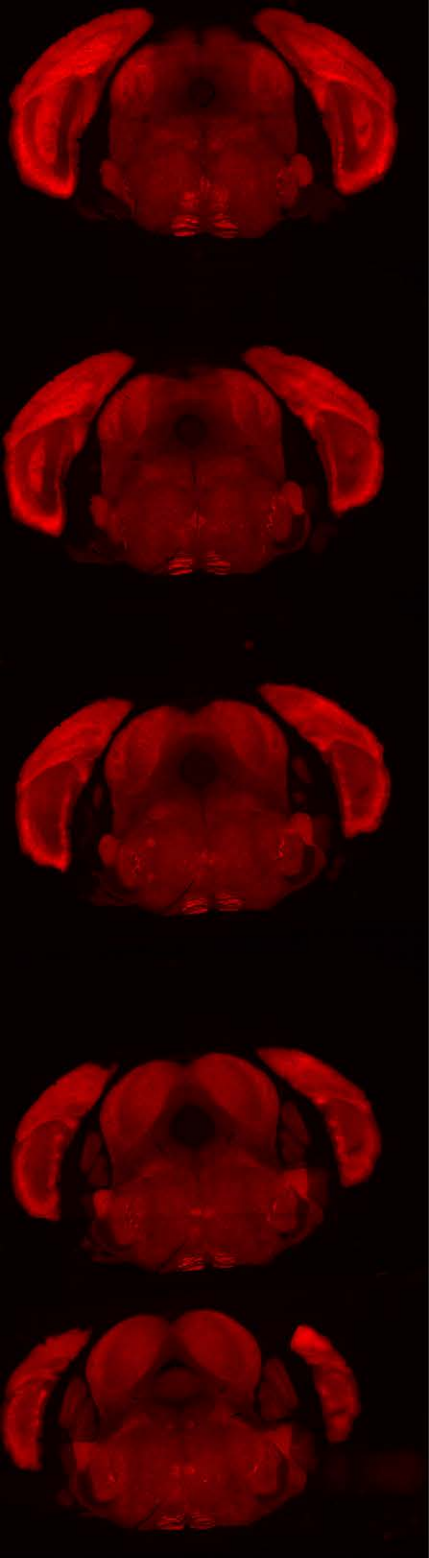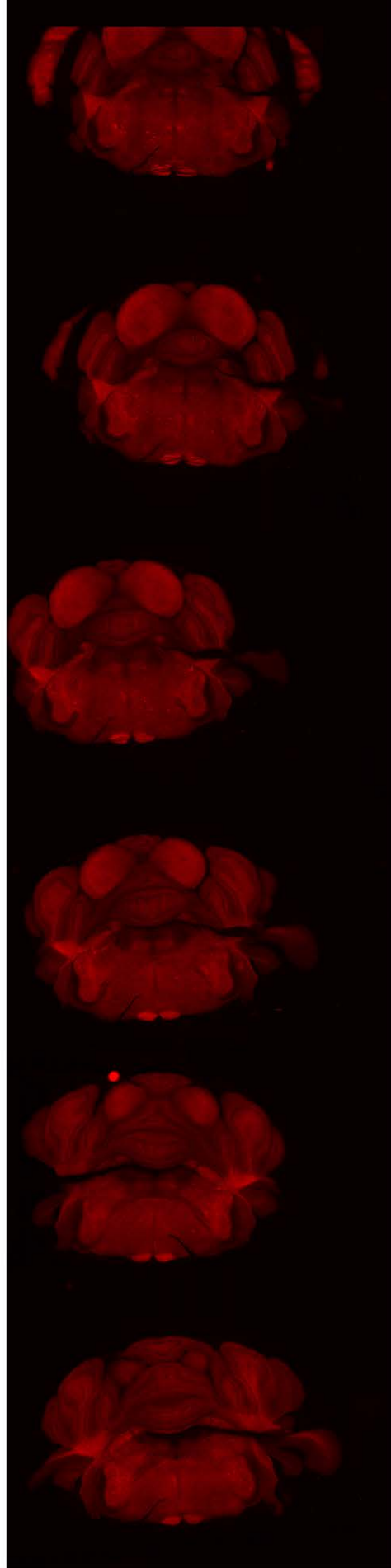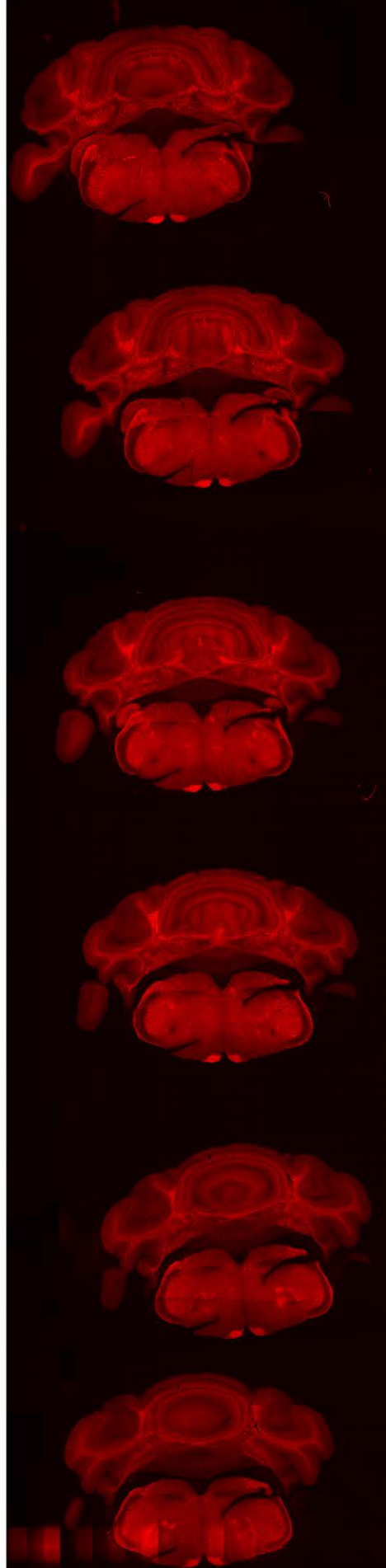

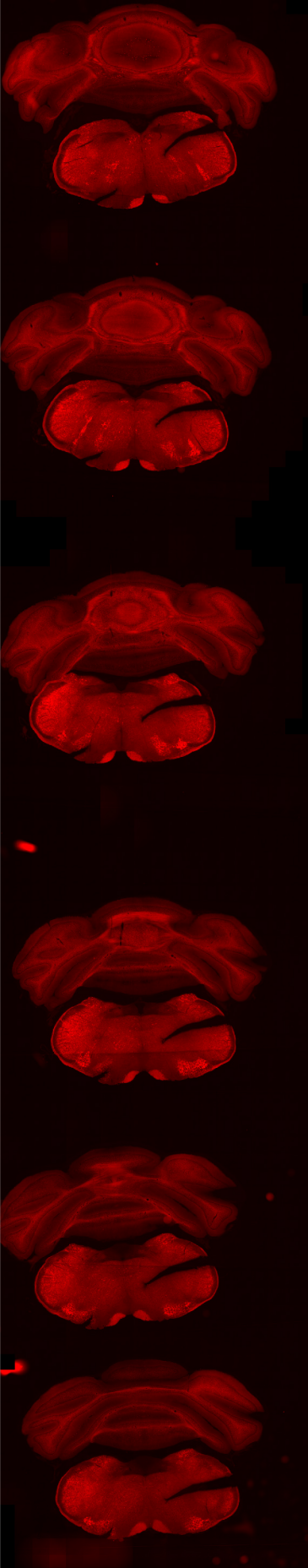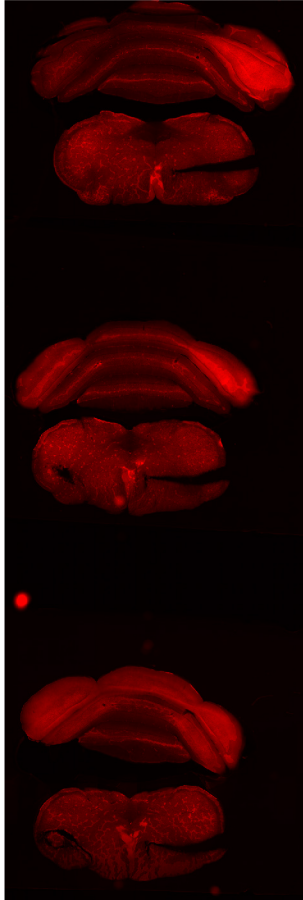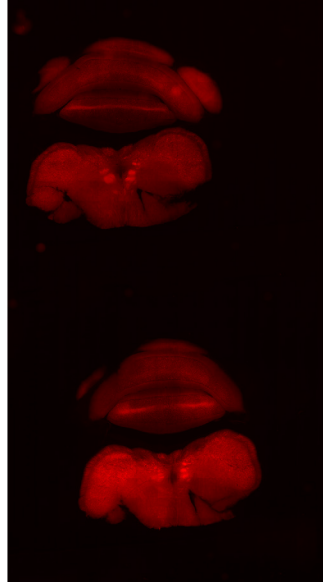

**GP 8.62**

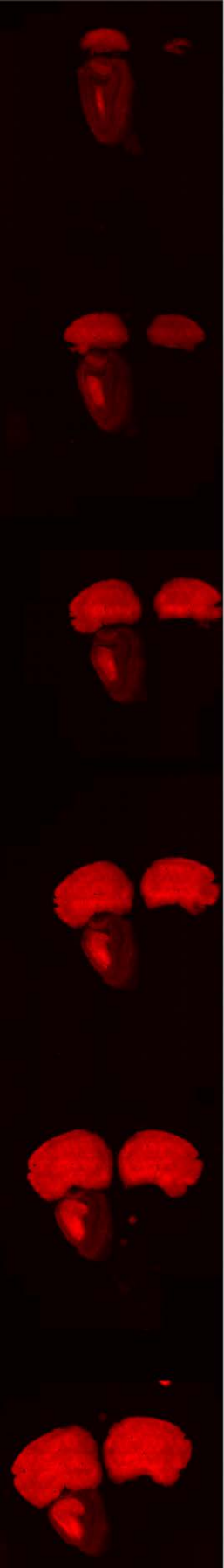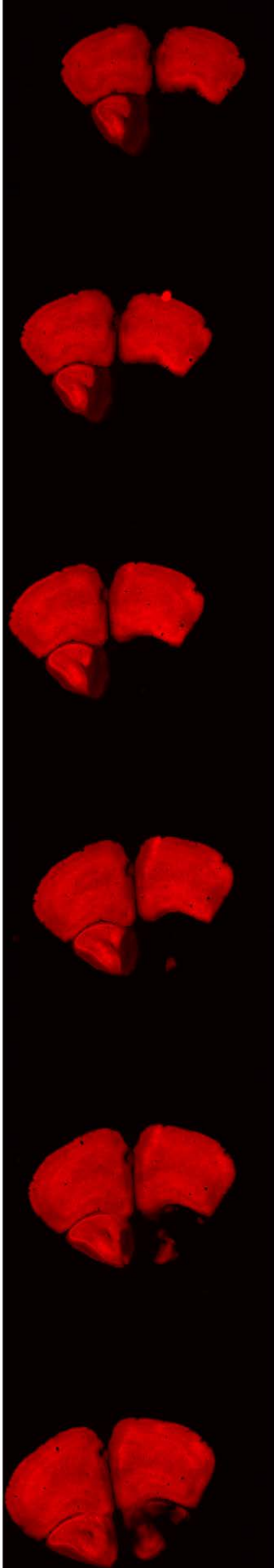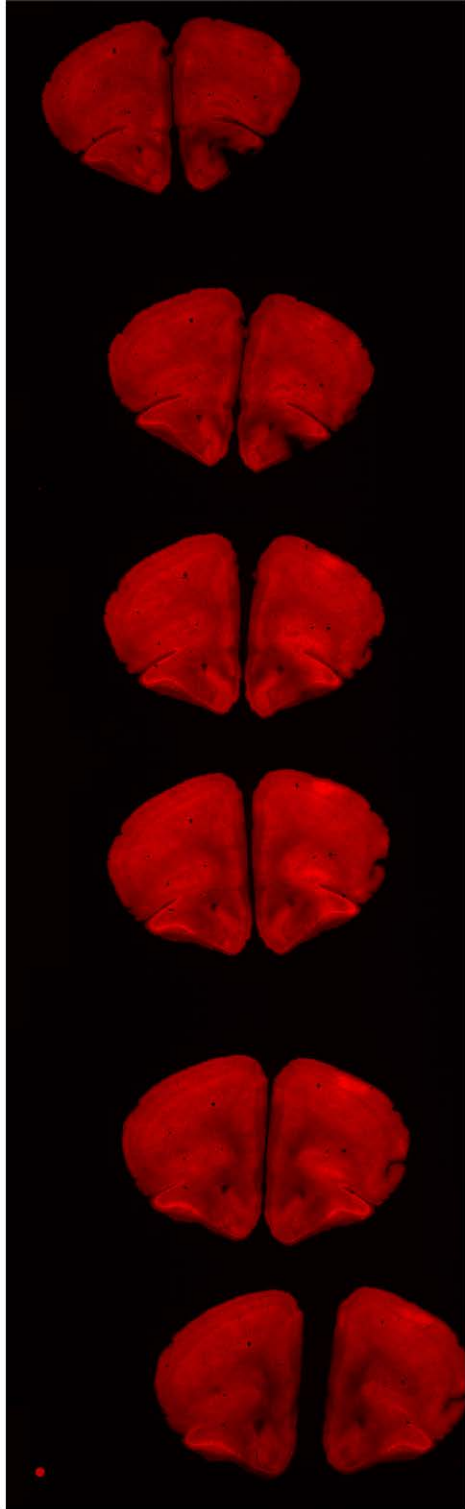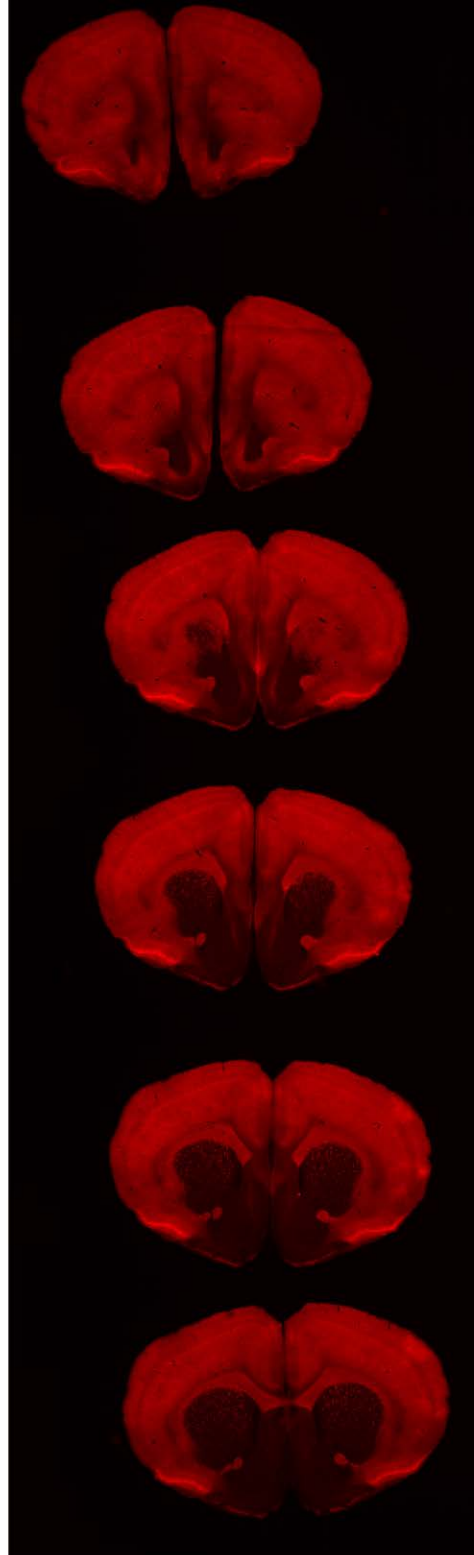

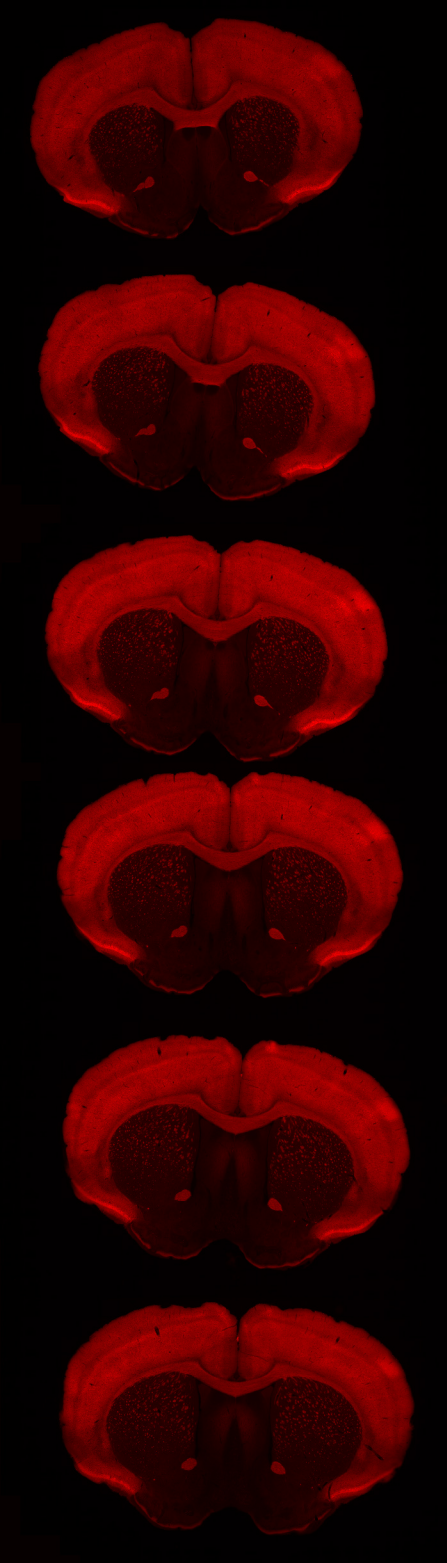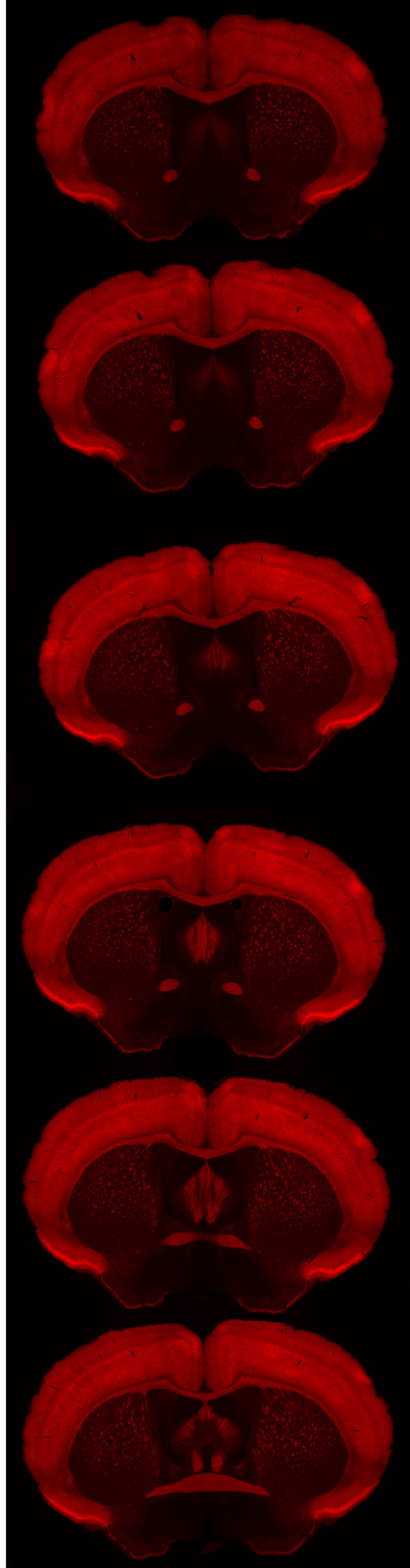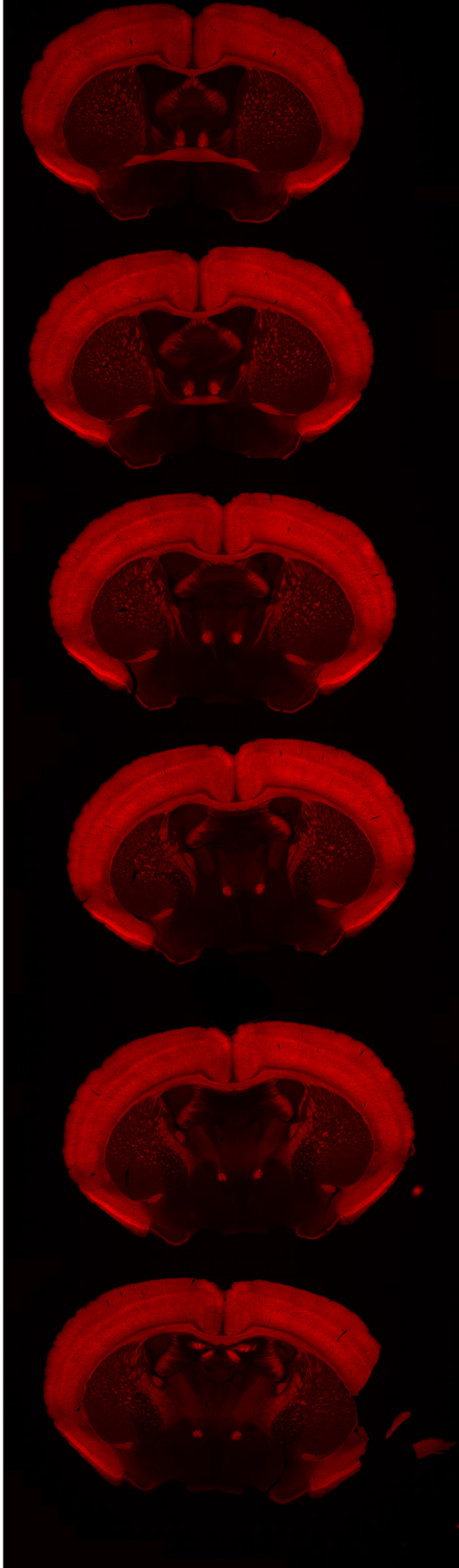

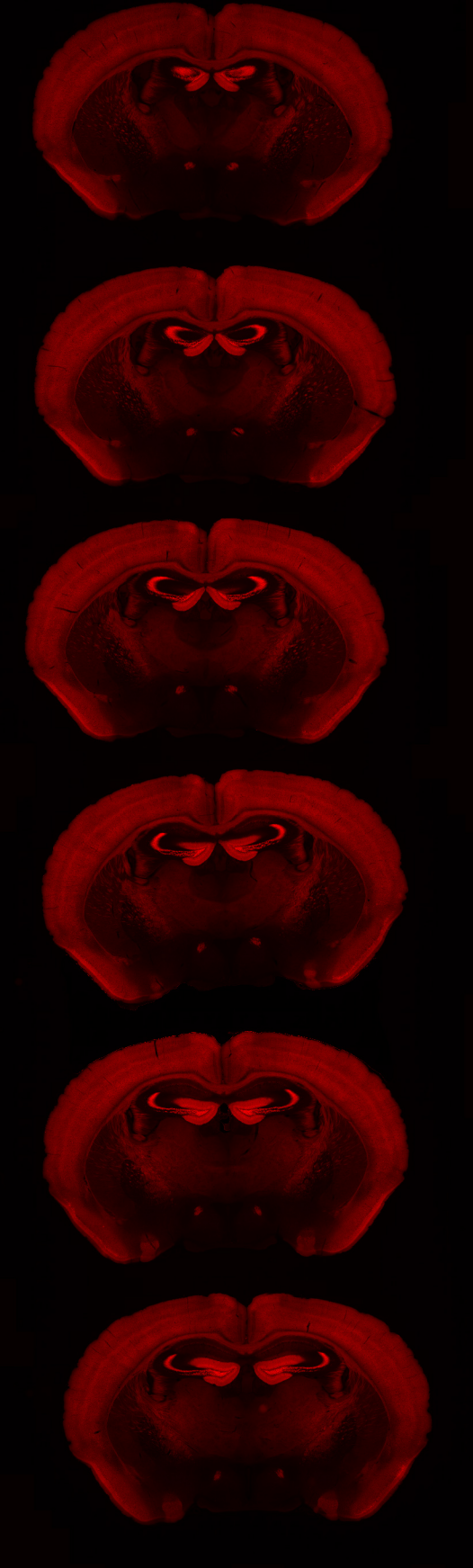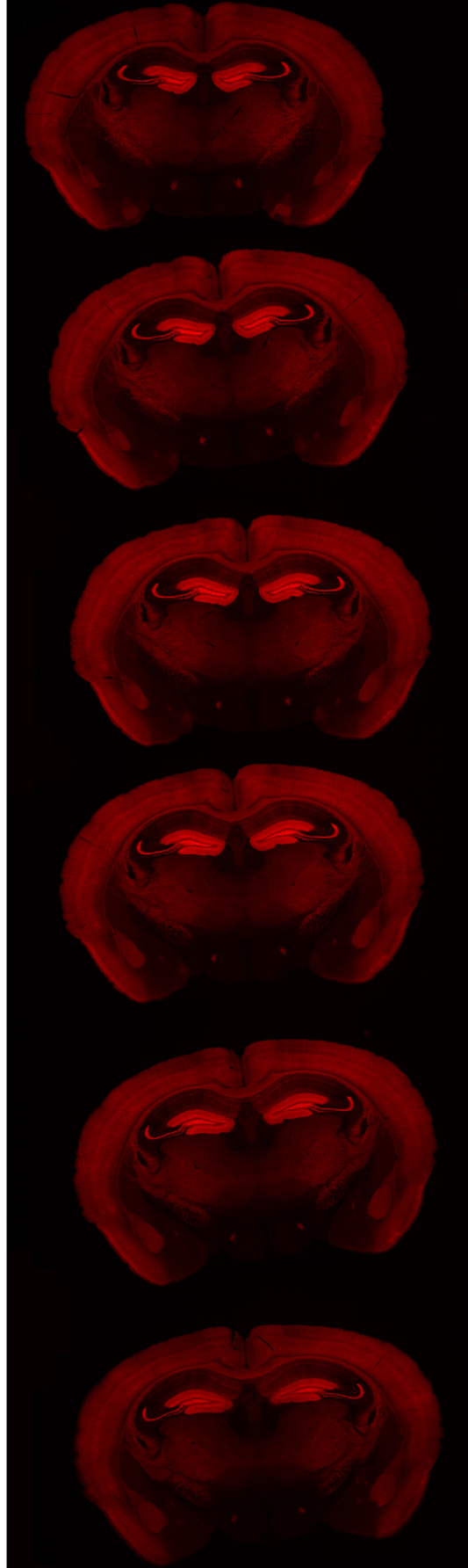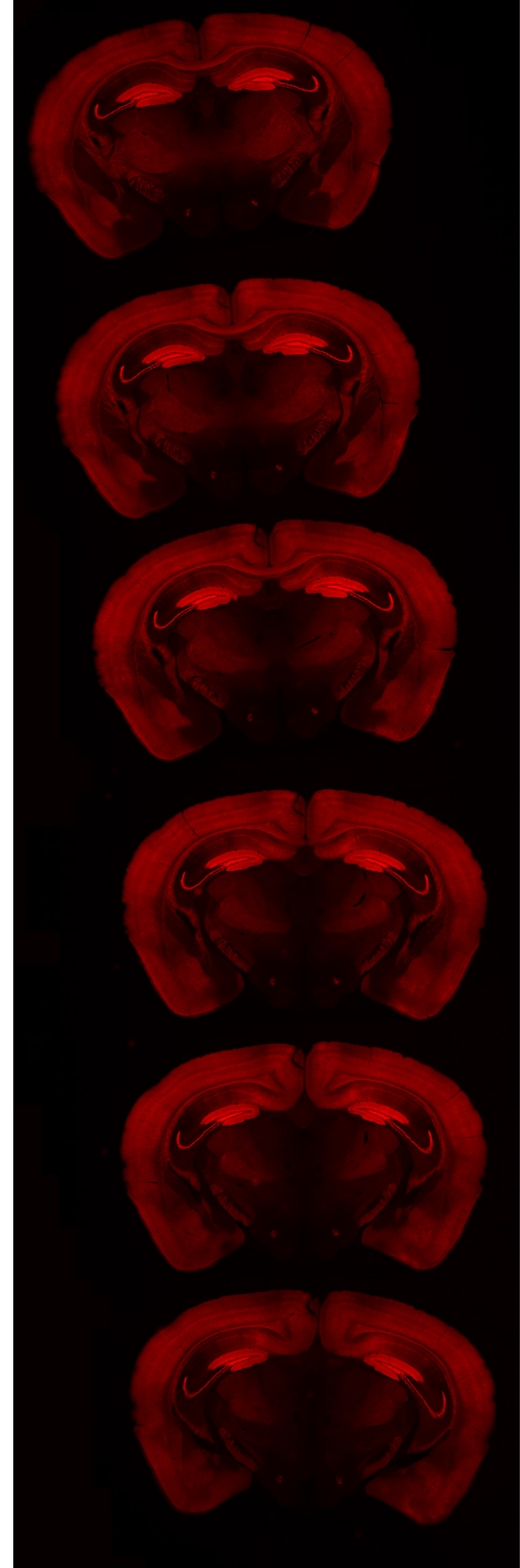

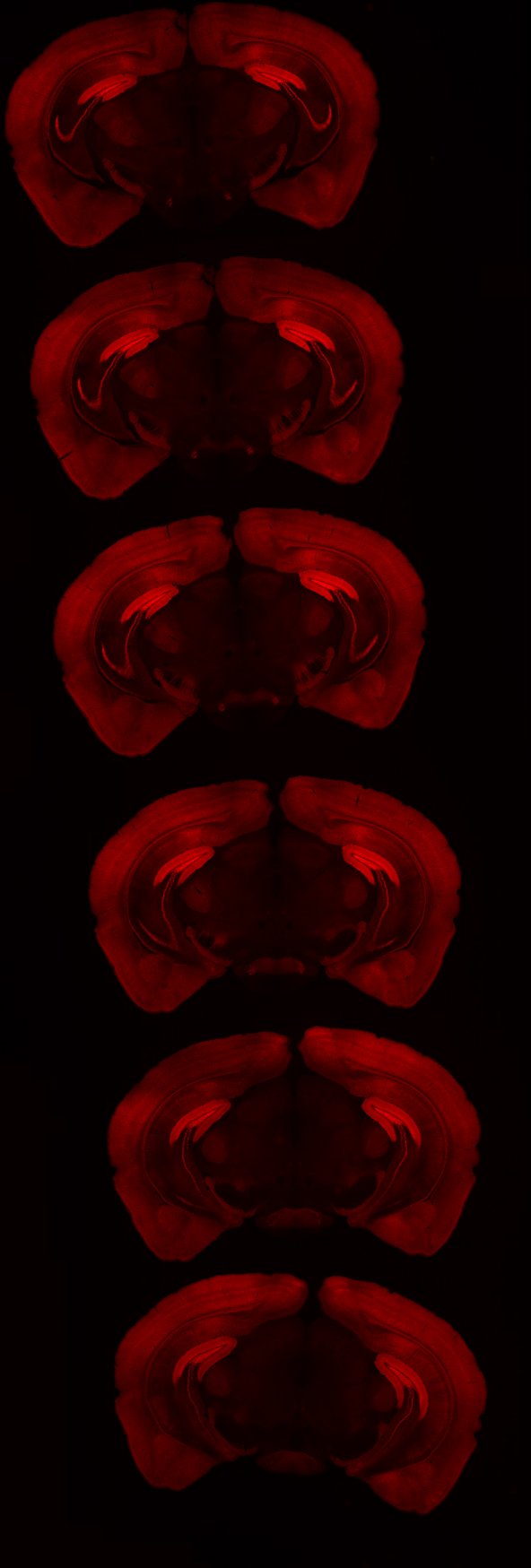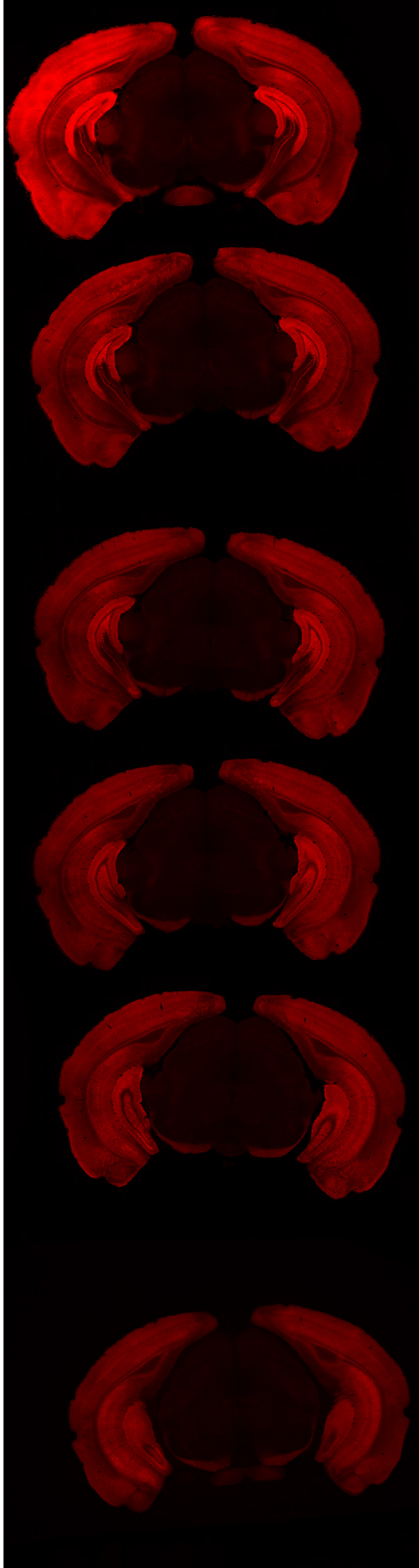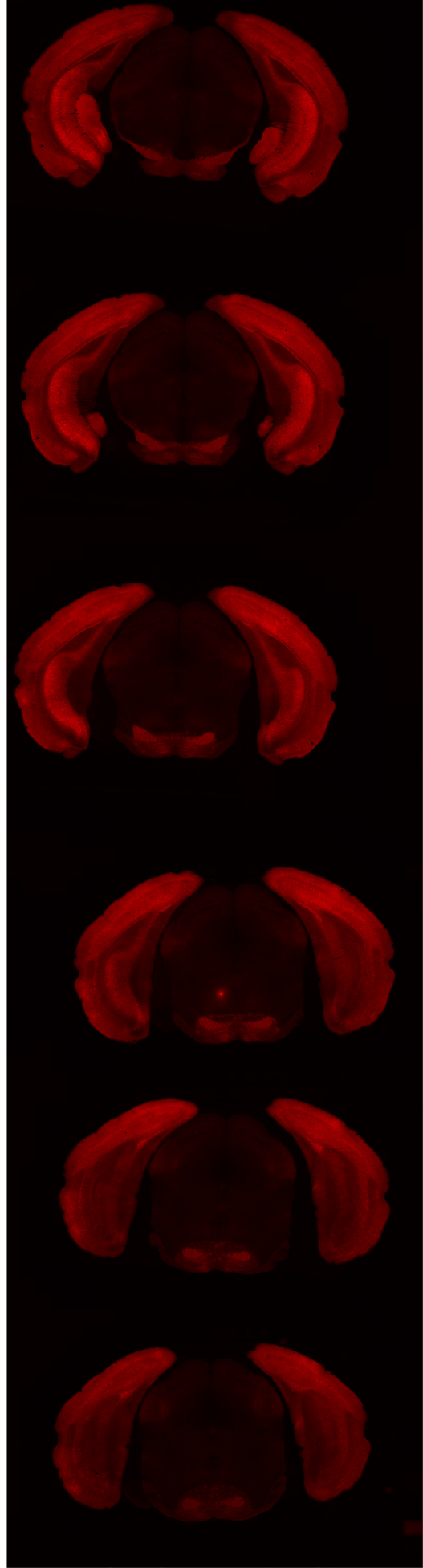

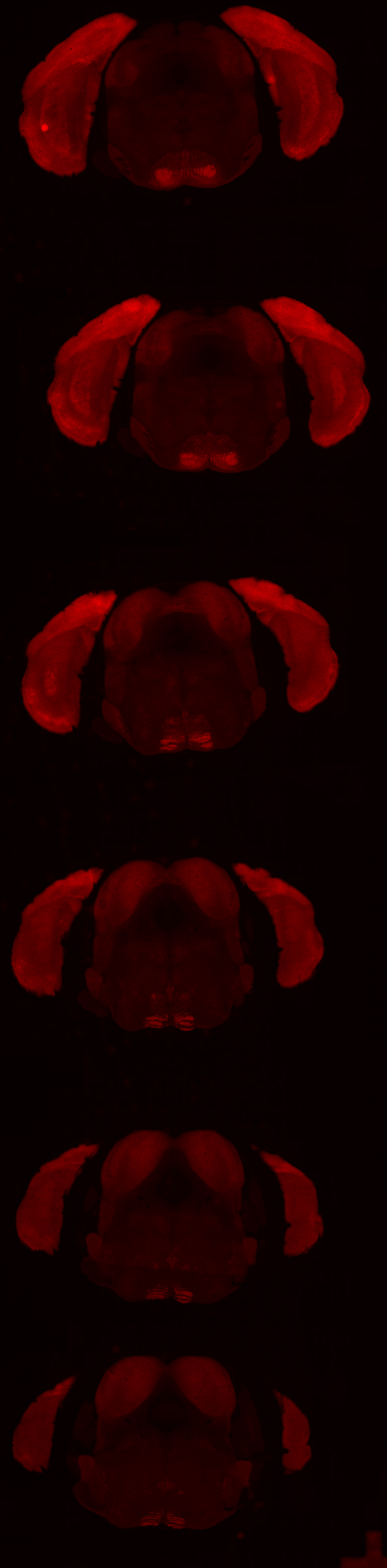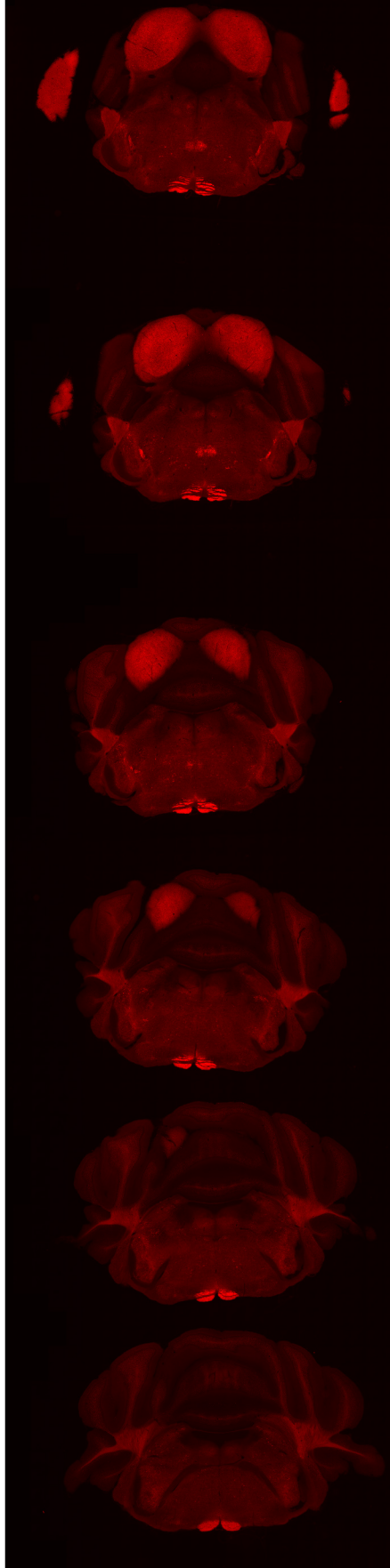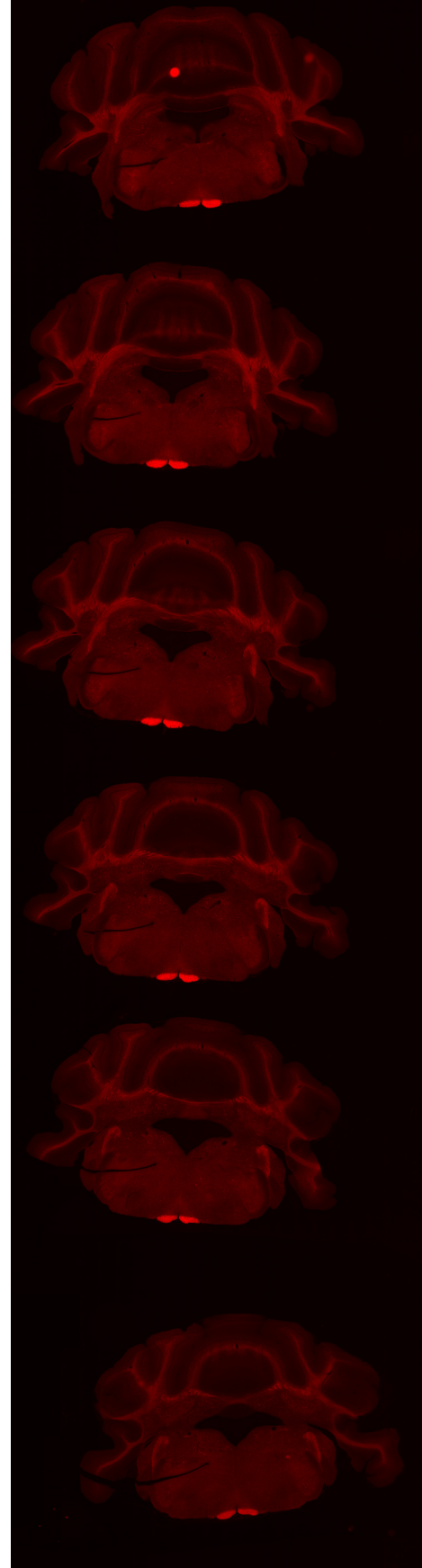

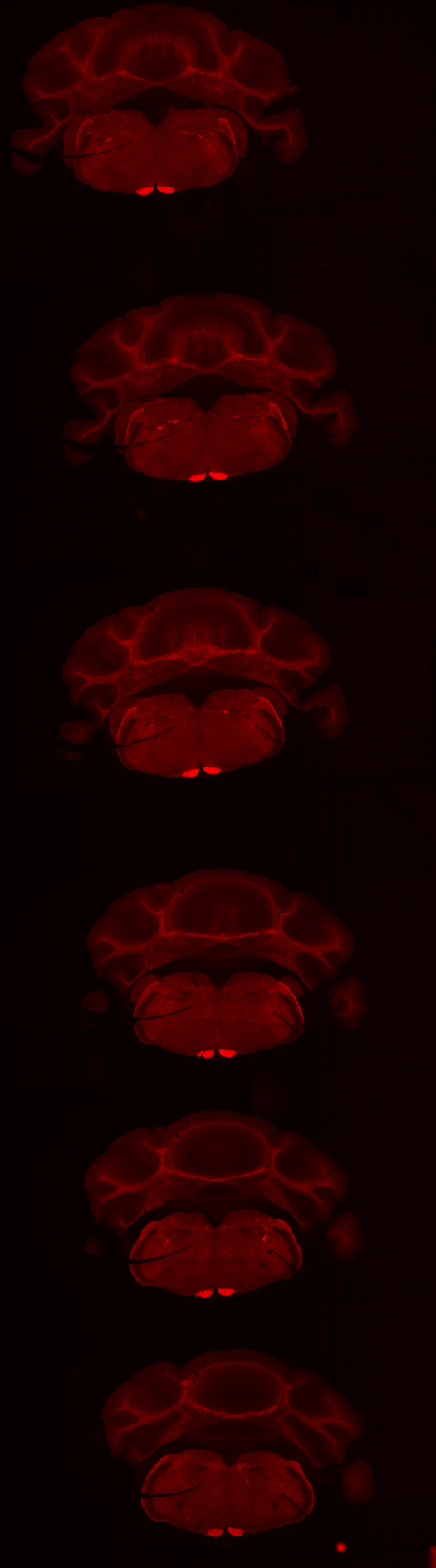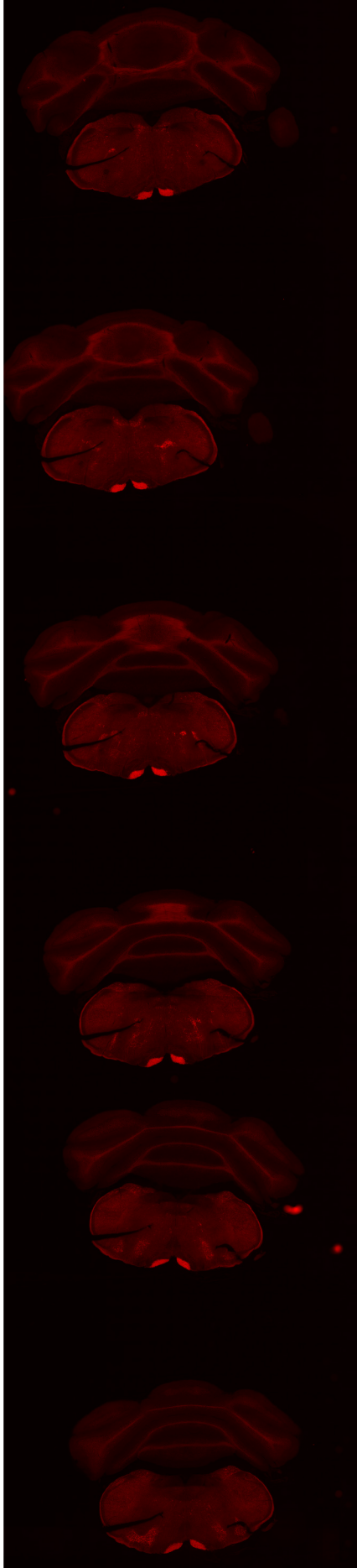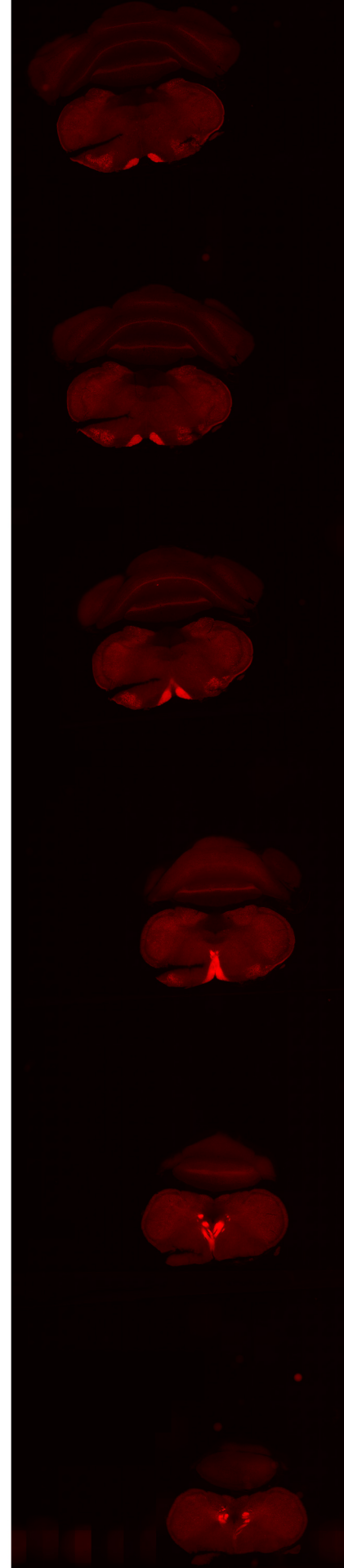

**GP 8.64**

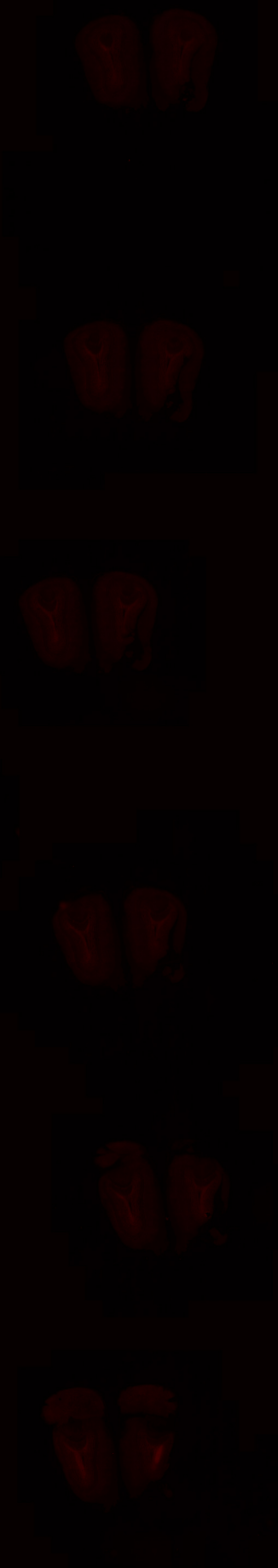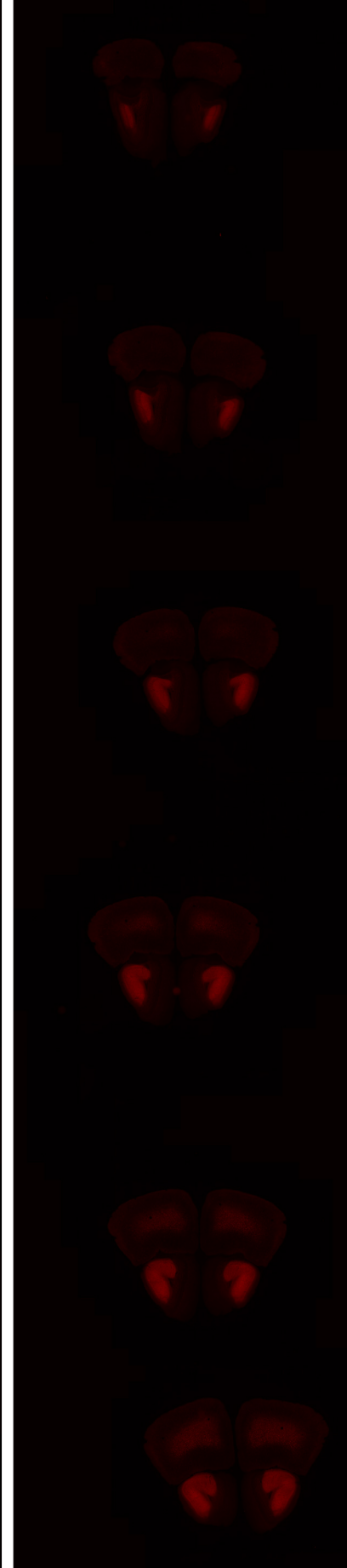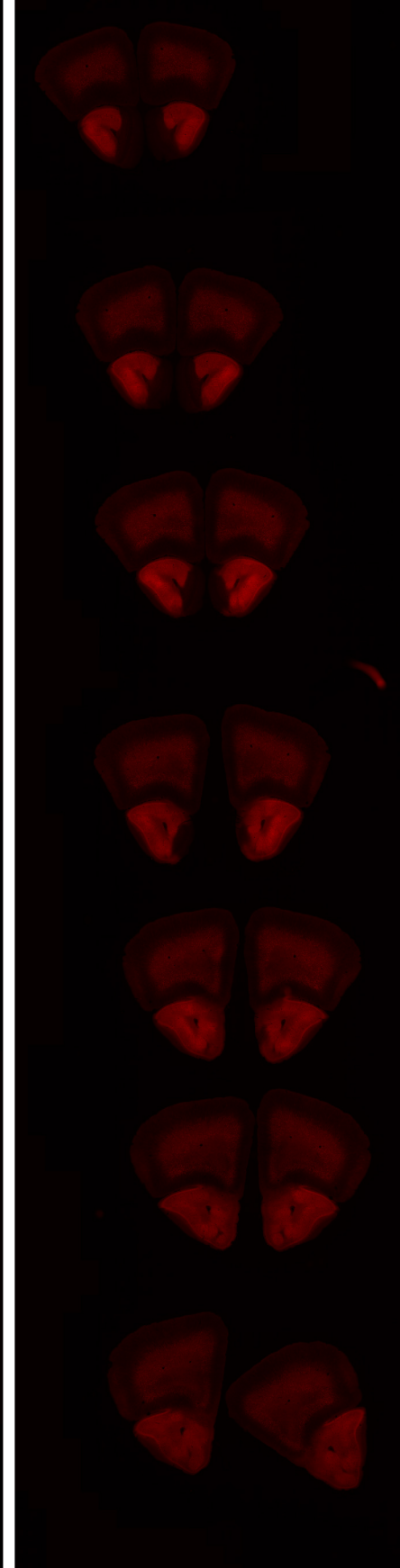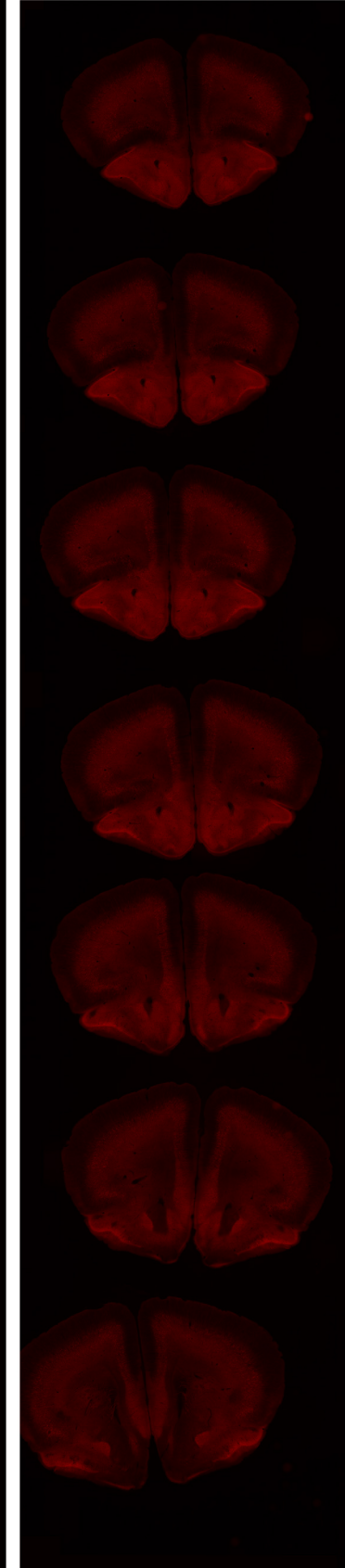

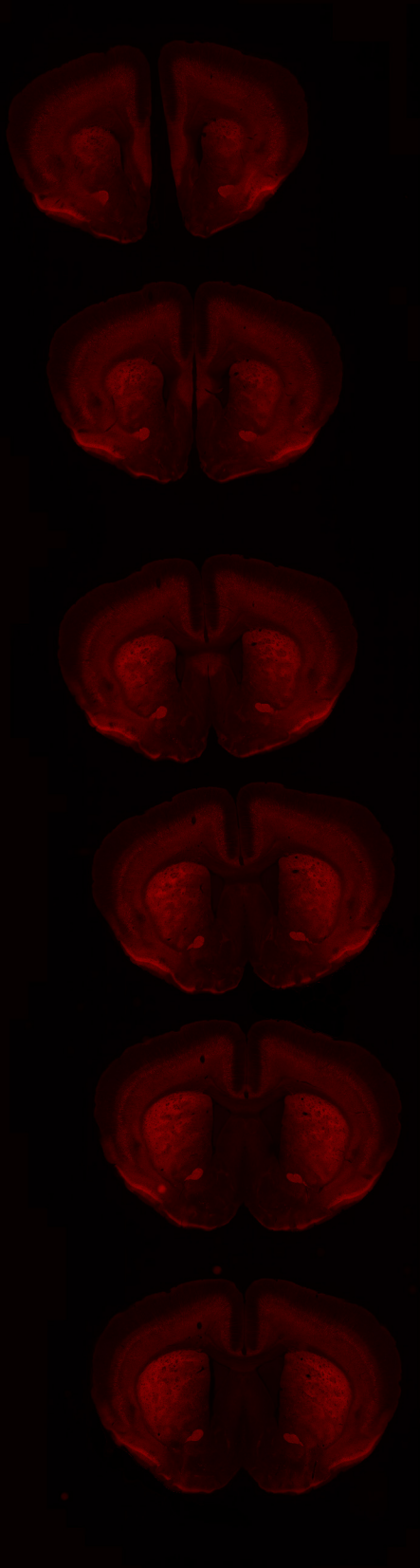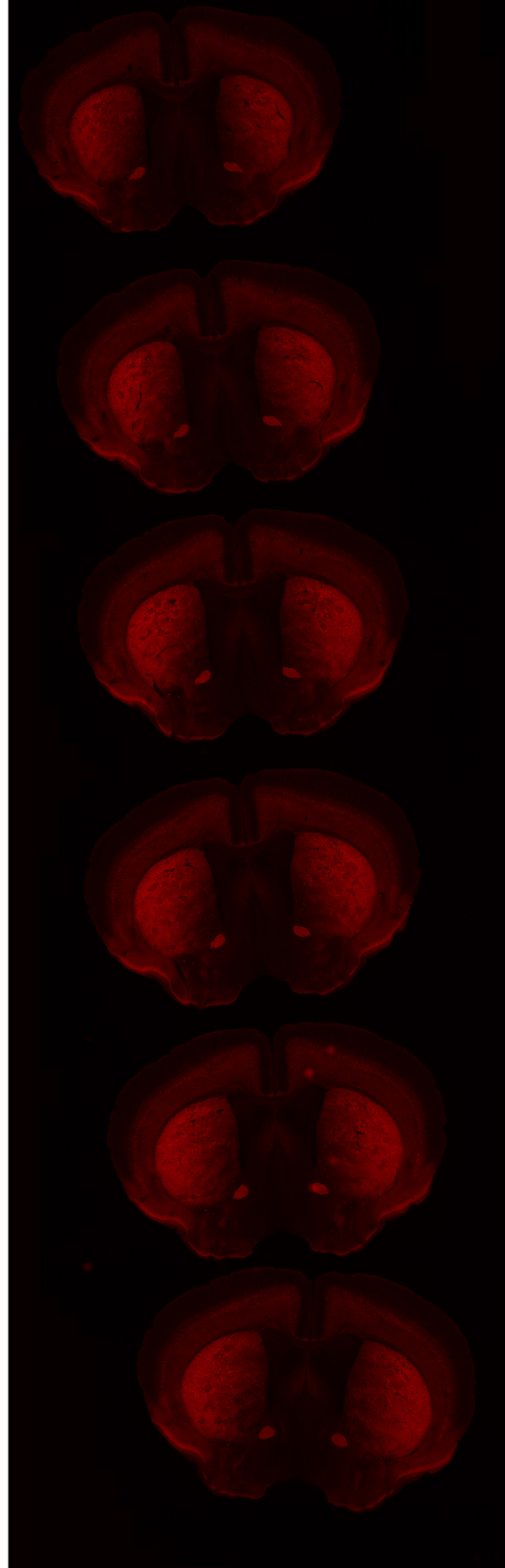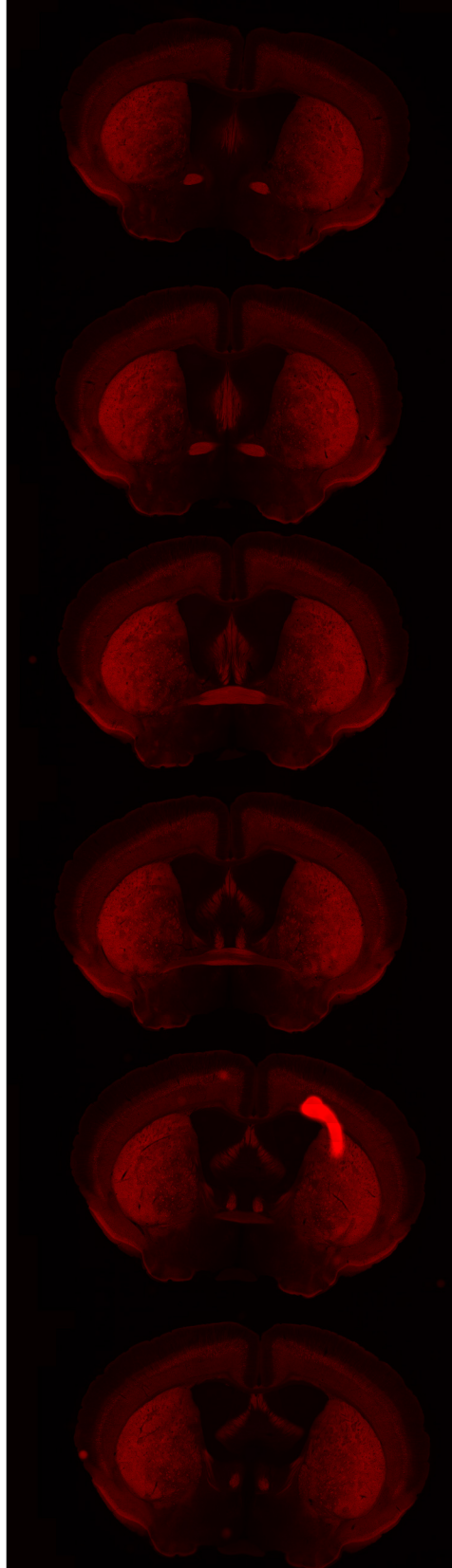

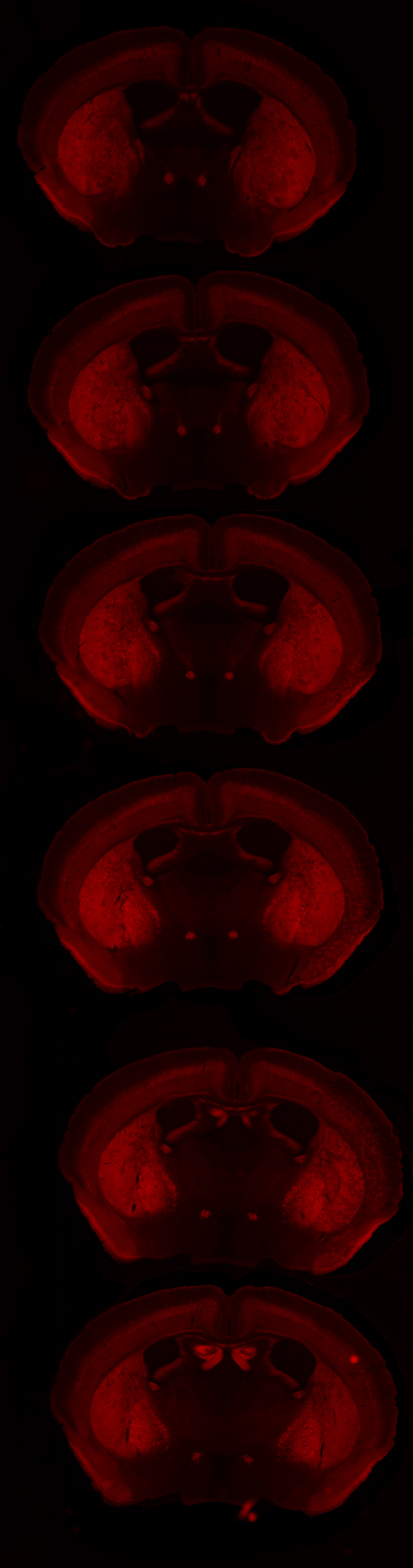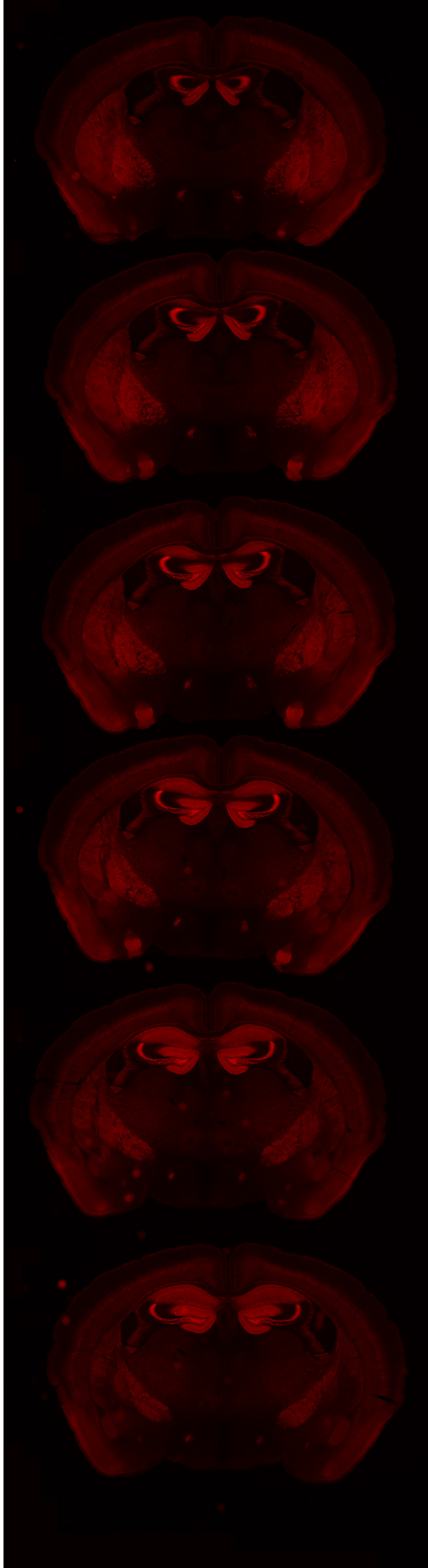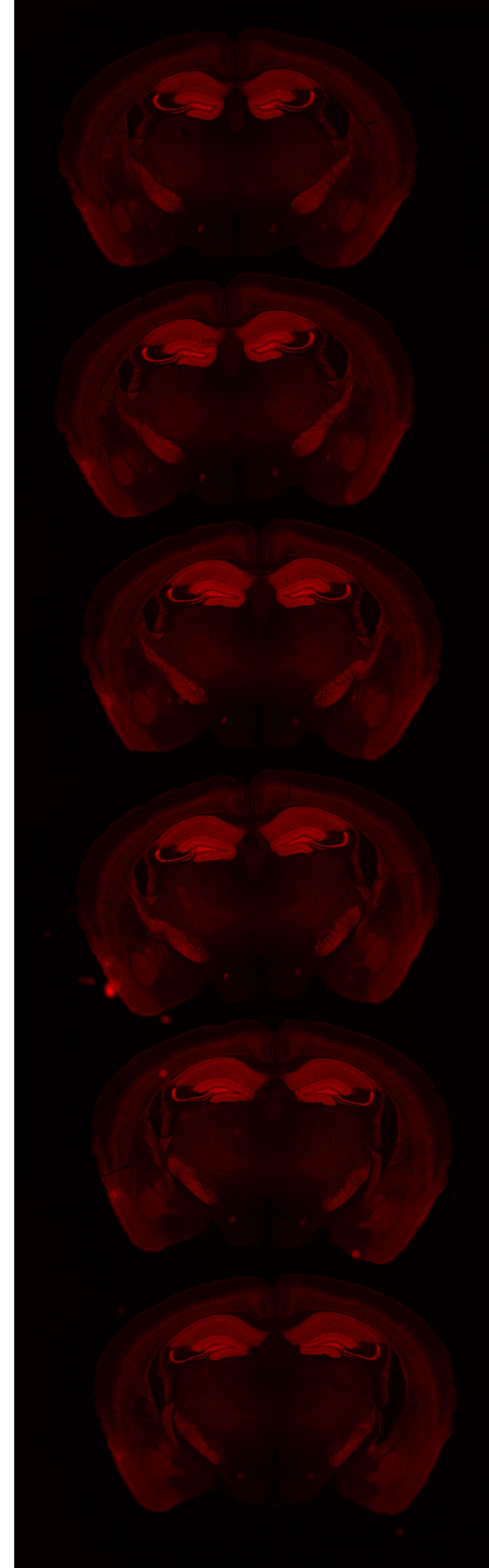

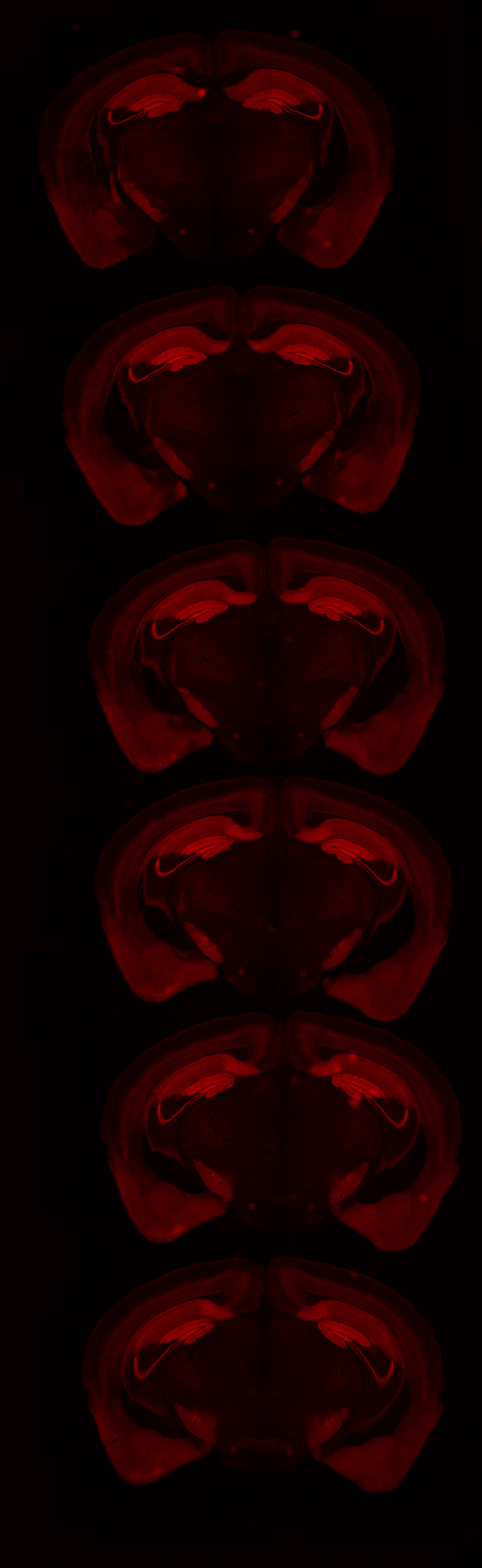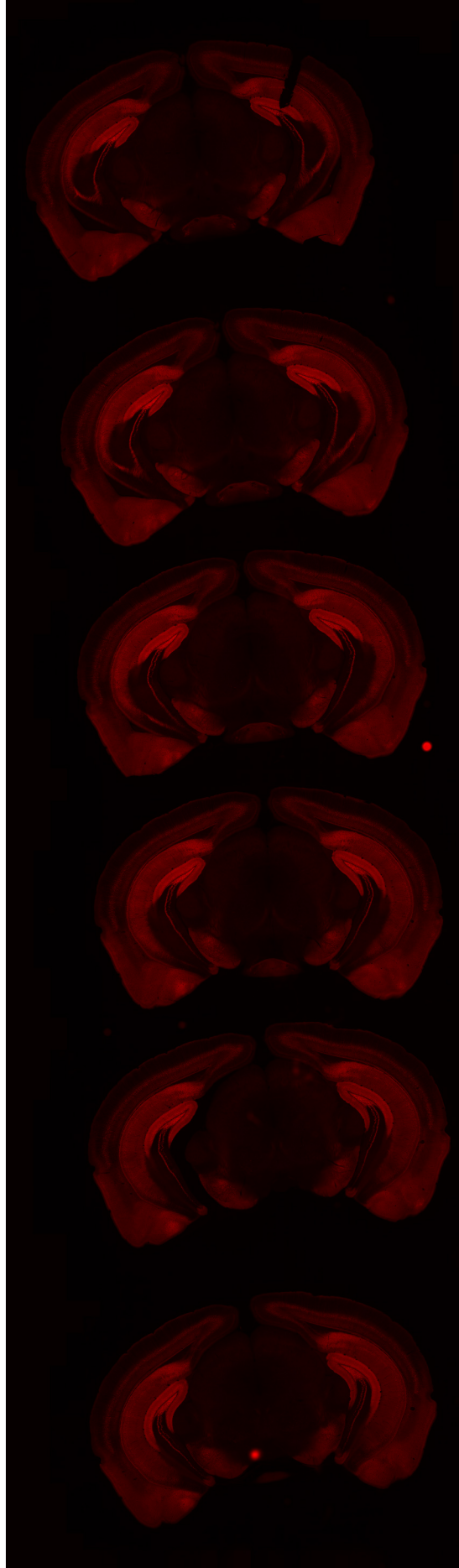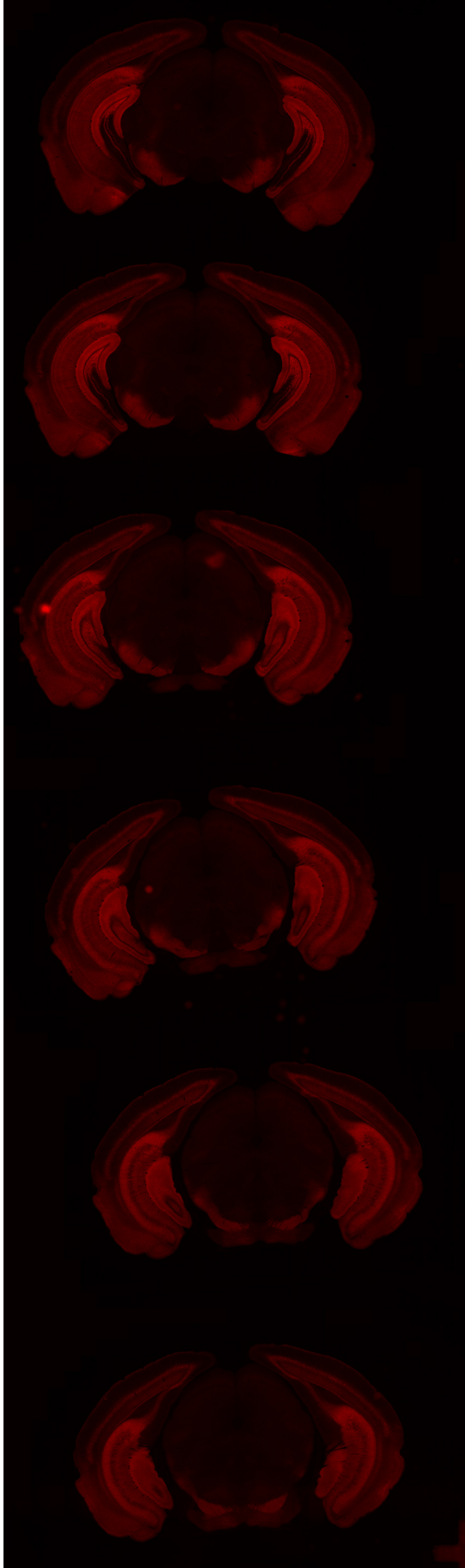

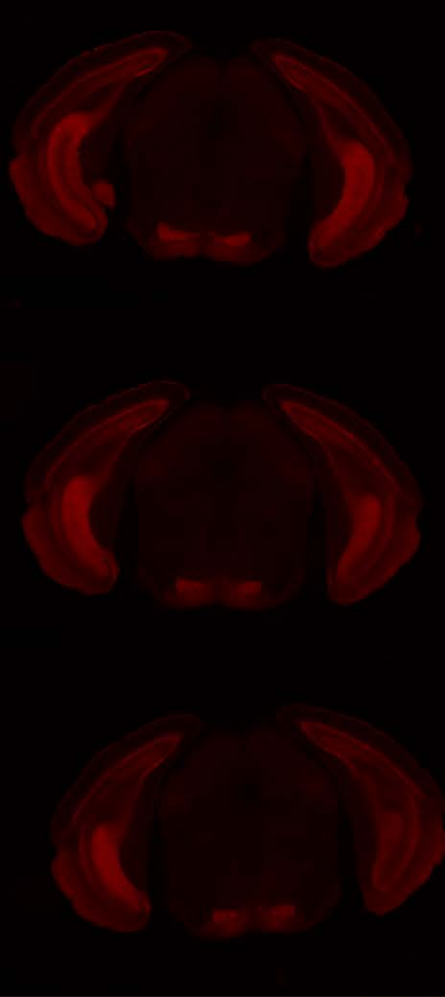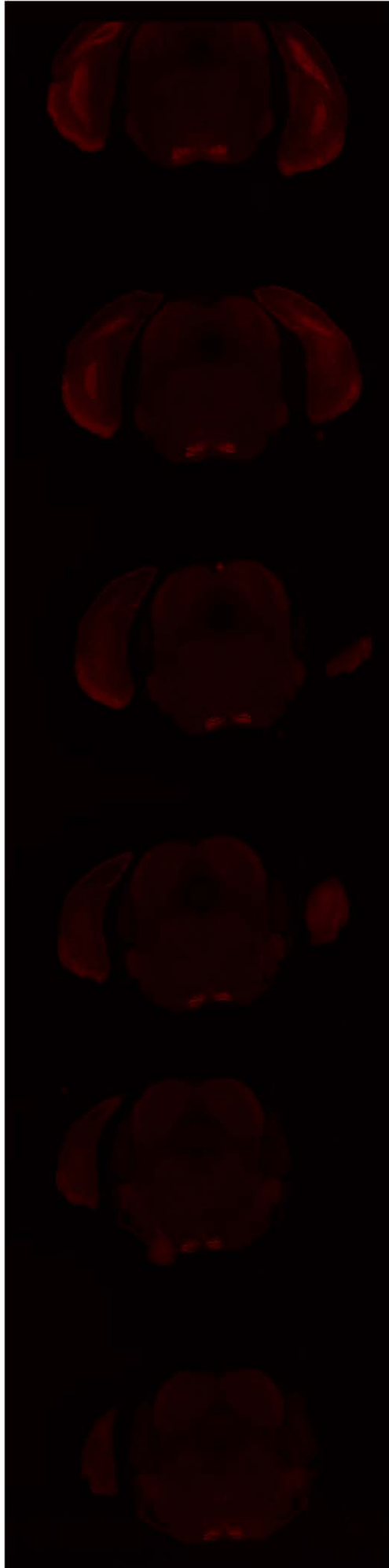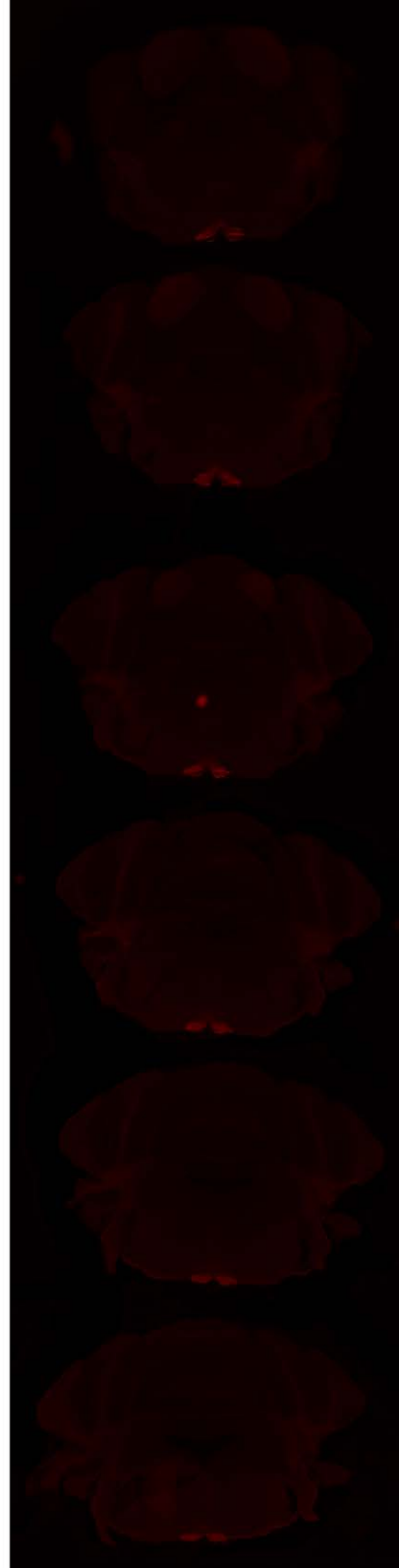

**GP 8.66**

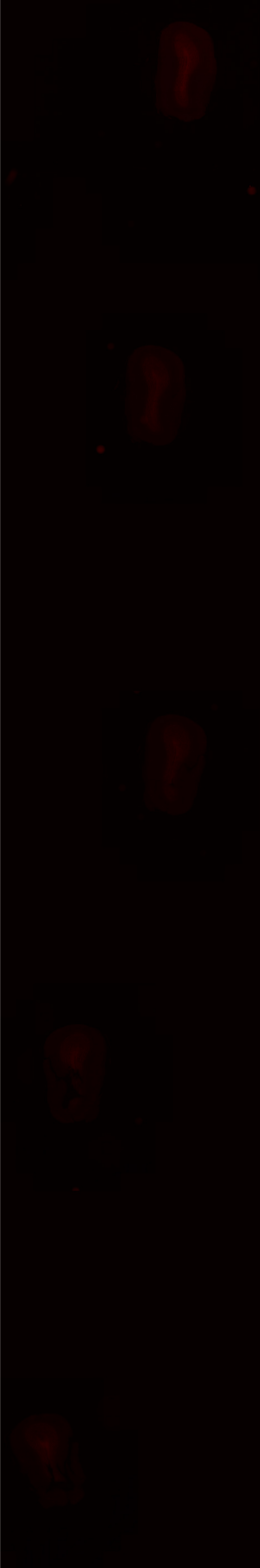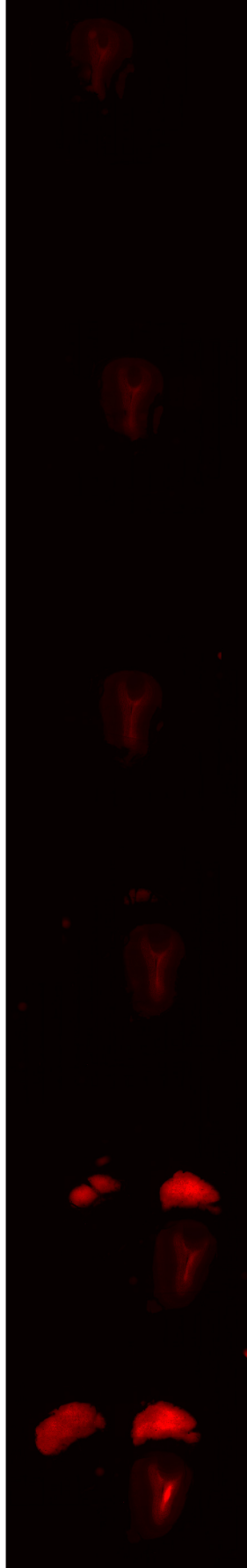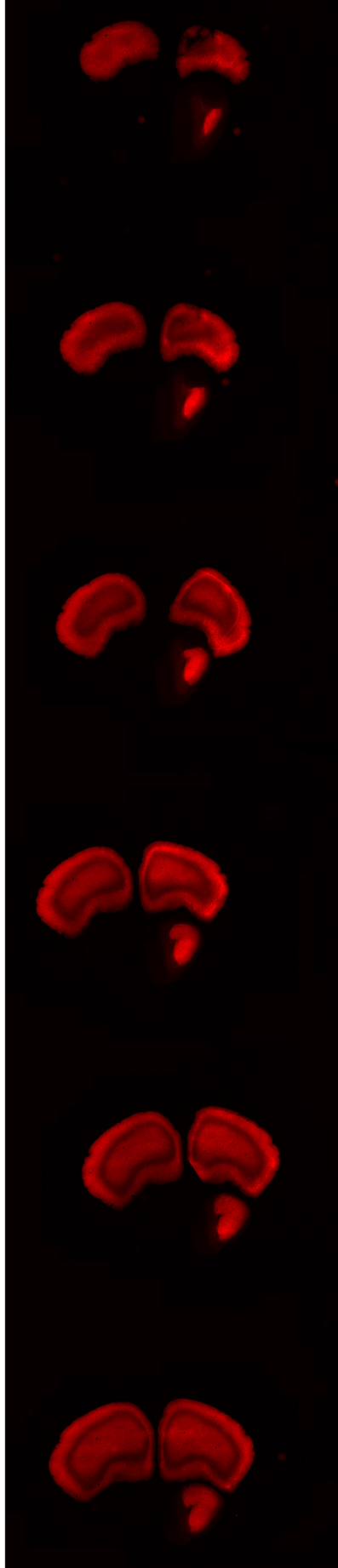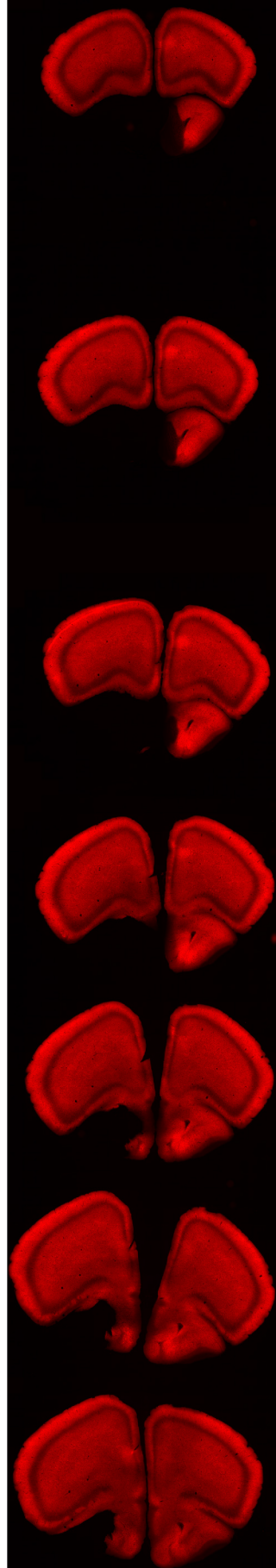

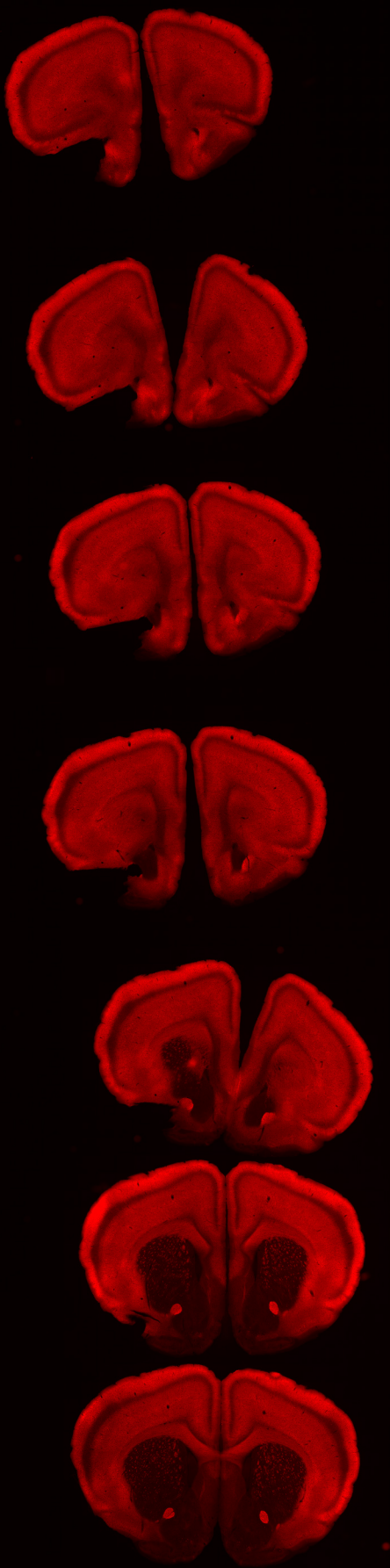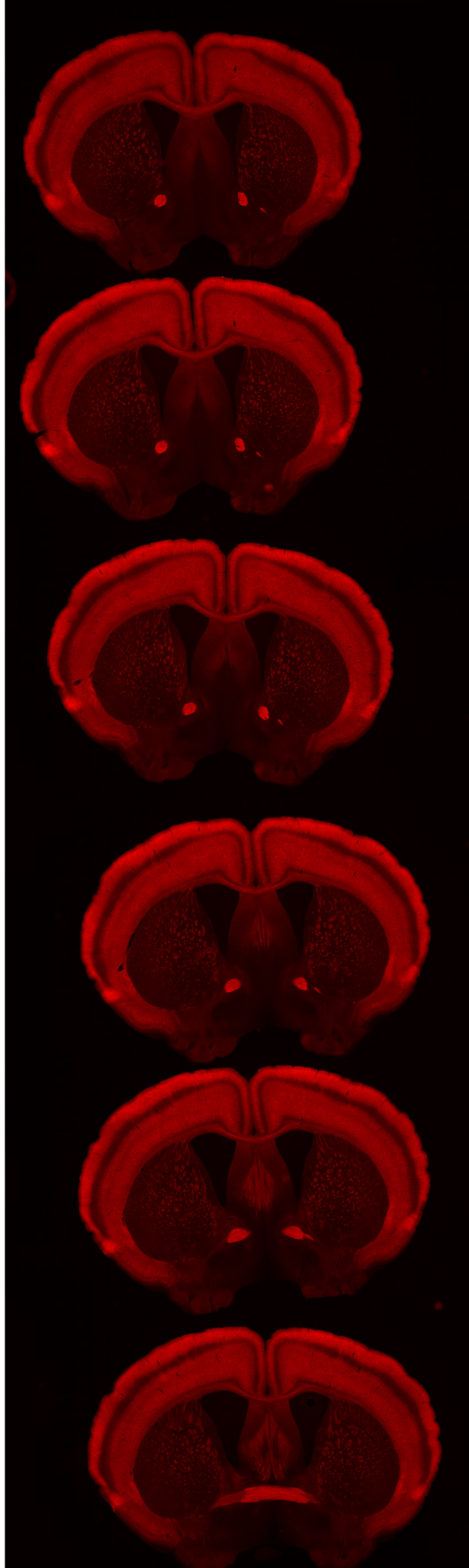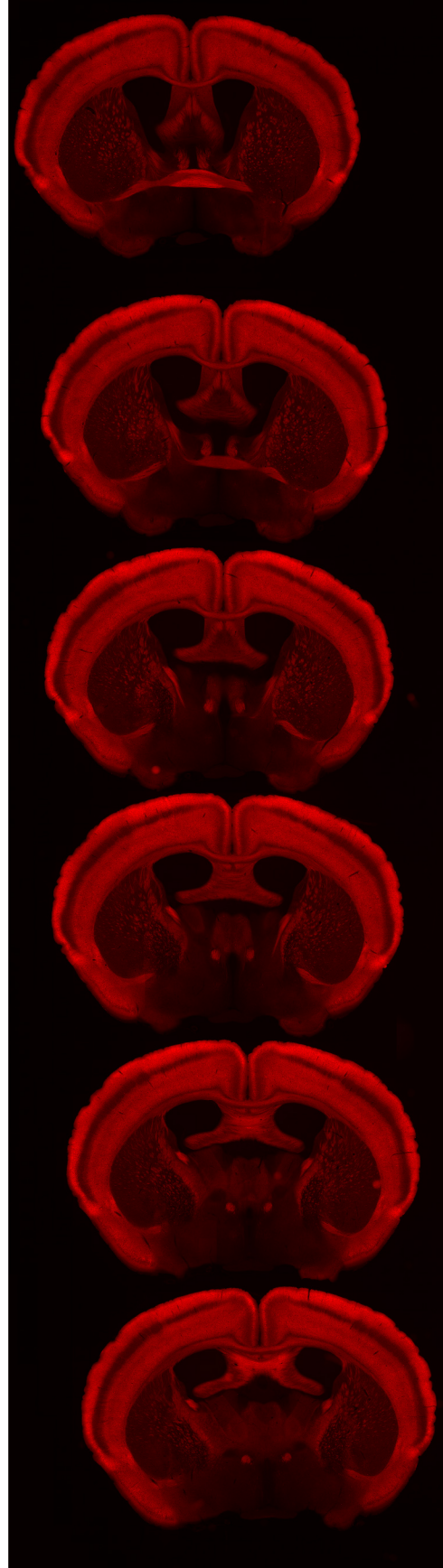

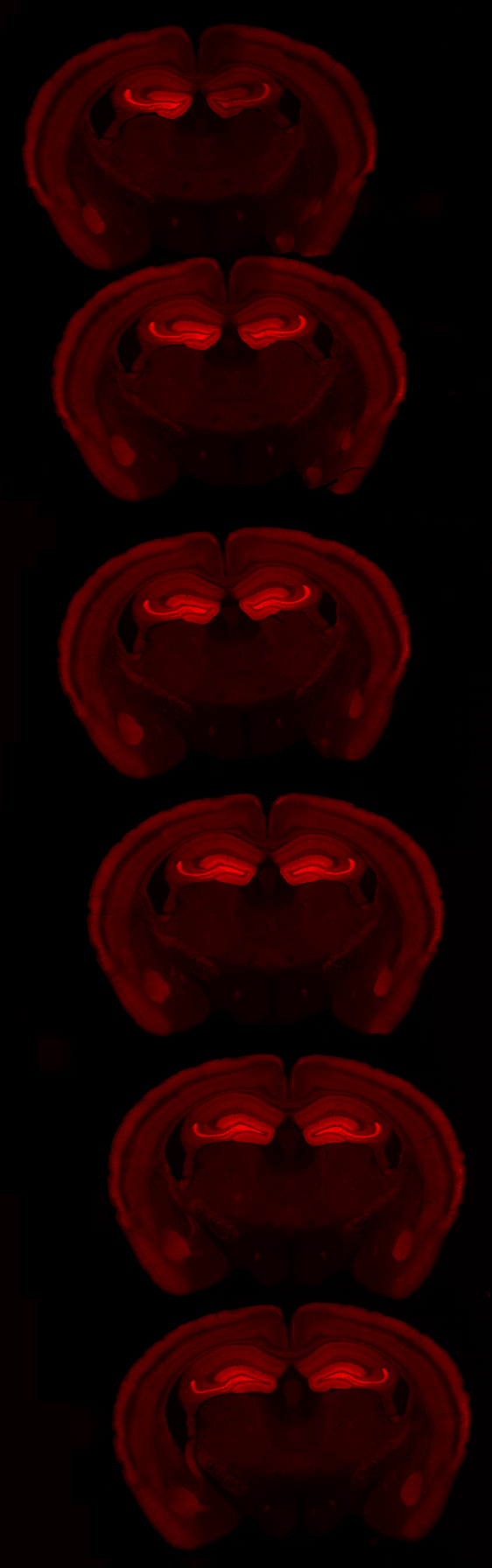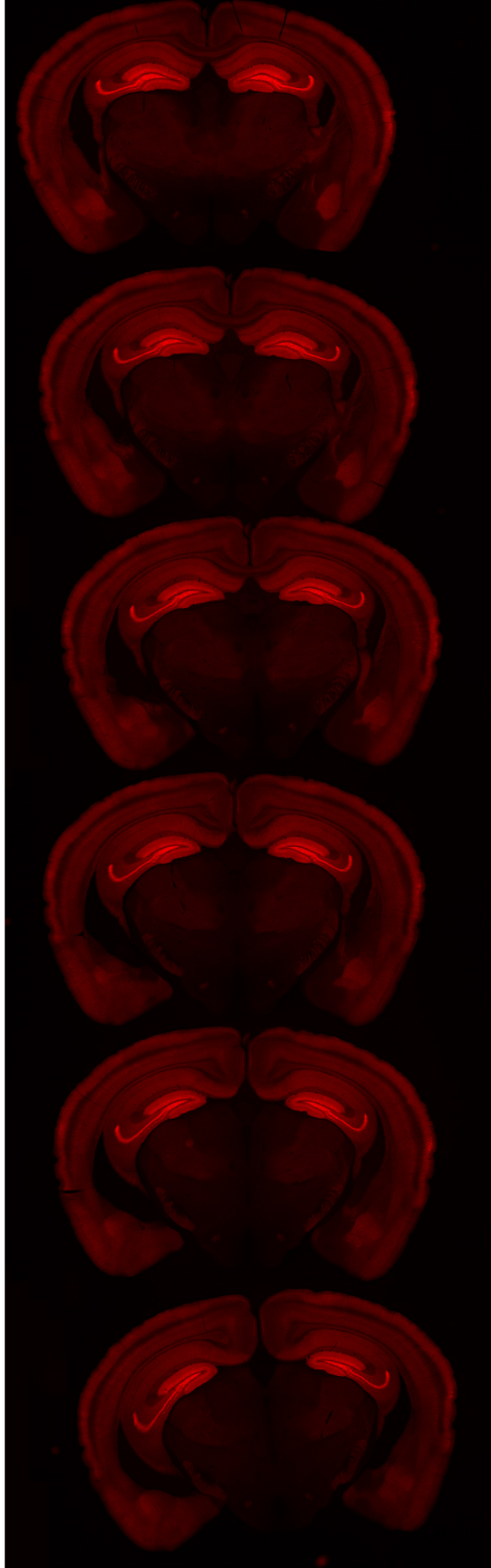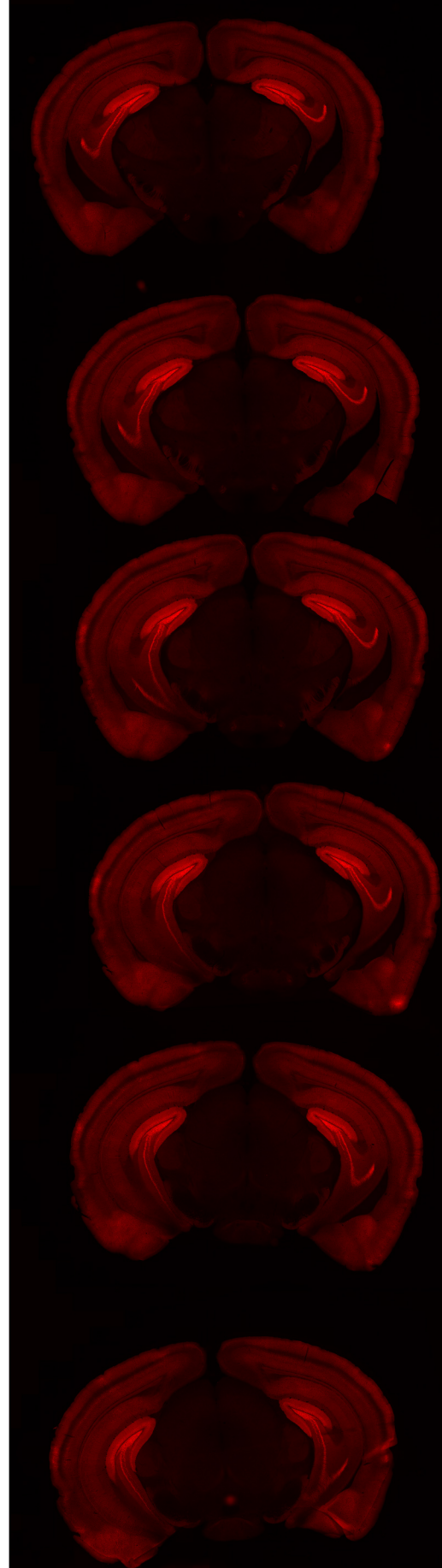

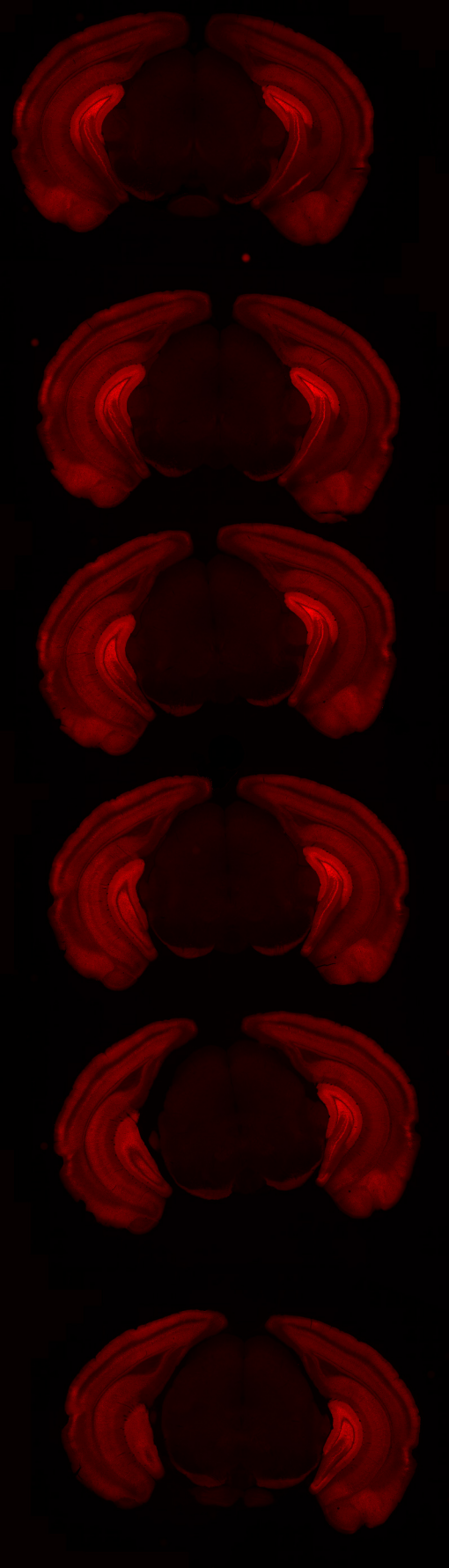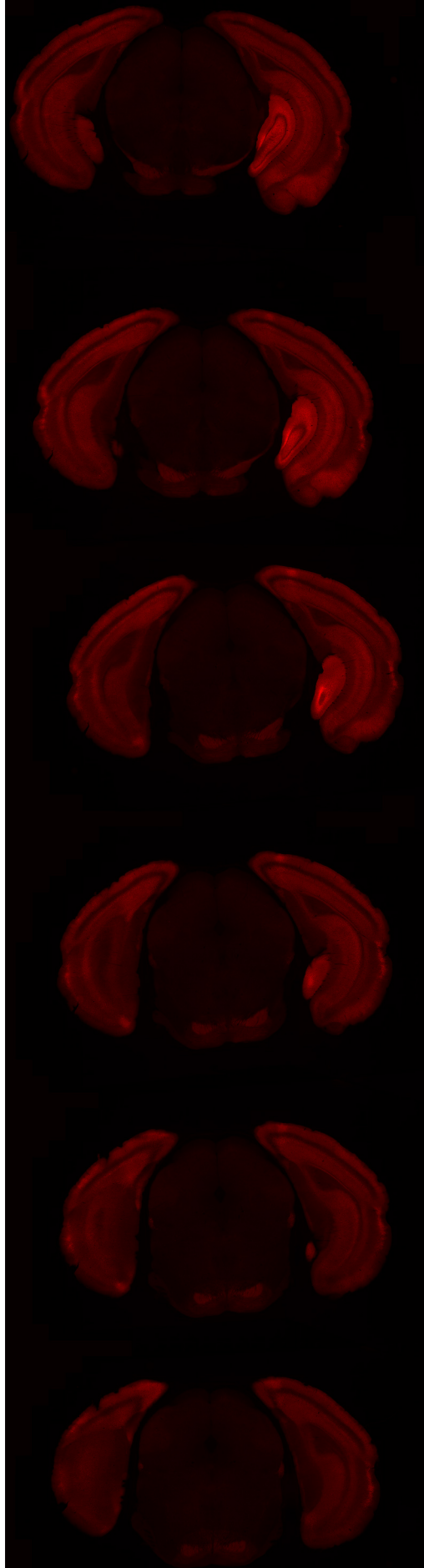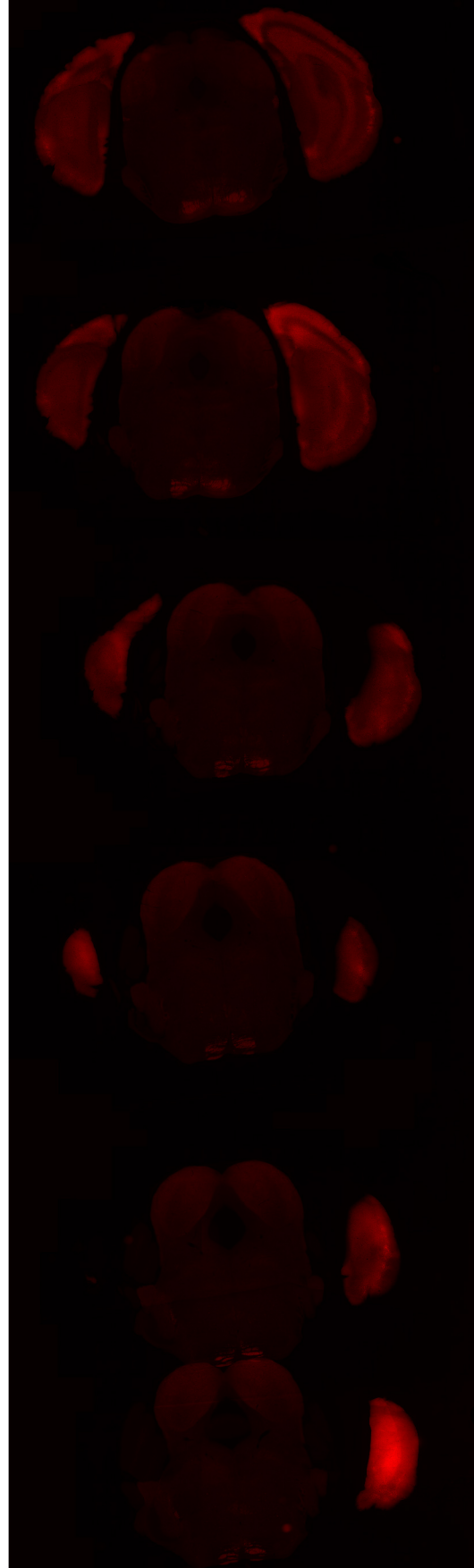

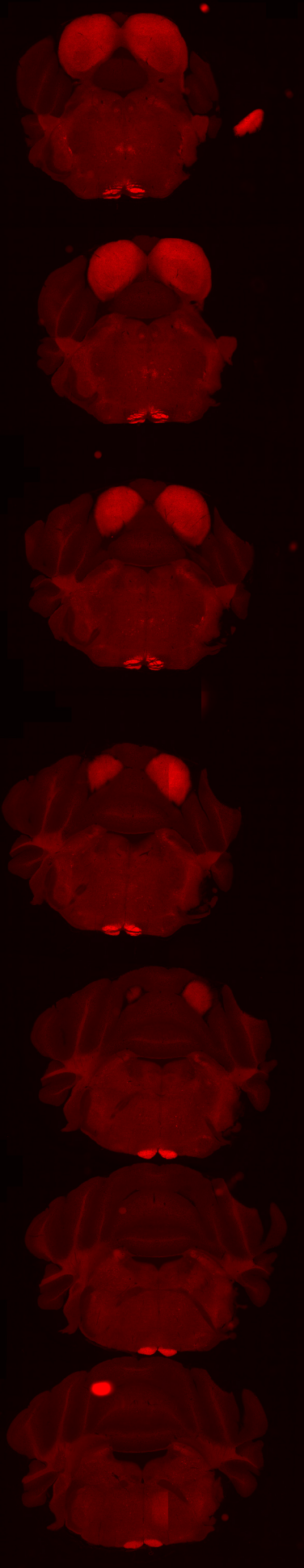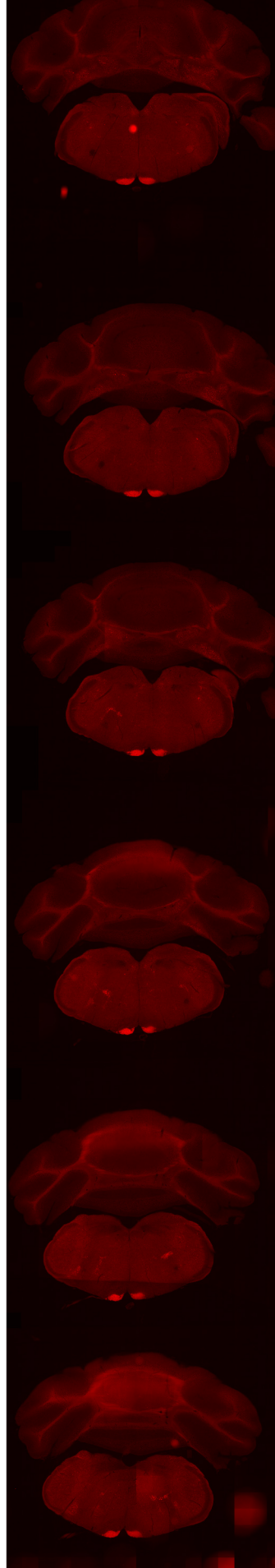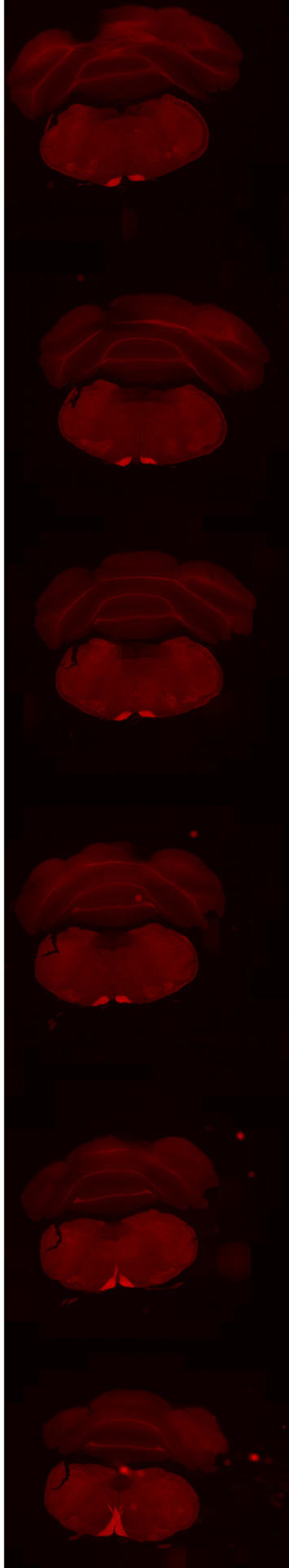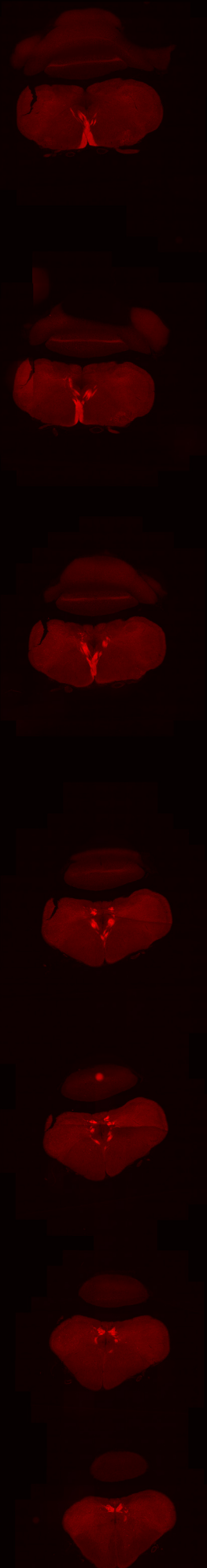

Supplement: S1 Data — Images of all characterized GP lines were taken using a slide scanner (Methods). All imaging conditions were kept identical, except for lines GP8.20, GP8.31, GP8.58, and GP.8.62, where the light intensity was reduced to 1/3 of its level with the other lines. Sections are organized from rostral to caudal on the slides, each slide direction is from top to bottom, and the slides are organized from left to right. (PDF) [file pone.0205444.s004.pdf]
